# Palladium/norbornene-catalyzed C–H bond activation and annulation to construct polycyclic aromatic hydrocarbon-based fluorescent materials†

**DOI:** 10.1039/d5sc00617a

**Published:** 2025-03-18

**Authors:** Chunlin Zhou, Xianhui Yang, Lian Gou, Bijin Li

**Affiliations:** a Chongqing Key Laboratory of Natural Product Synthesis and Drug Research, School of Pharmaceutical Sciences, Chongqing University Chongqing 401331 P. R. China bijinli@cqu.edu.cn

## Abstract

Reported herein is the first example of NBE-CO_2_Me-mediated palladium-catalyzed C–H bond activation and annulation of bromo(hetero)aromatics to construct structurally diverse polycyclic aromatic hydrocarbon (PAH)-based fluorescent materials. The approach shows a broad substrate scope and provides straightforward access to screening high-performance fluorescent materials. A novel organic single-molecule white-light material with anti-Kasha dual-emission characteristics has been developed herein. Furthermore, the anti-Kasha dual-emission material was fabricated as water-dispersed nanoparticles (NPs) to target the mitochondria of living cells. The corresponding NPs could be further applied in two-channel emission intensity ratio imaging to observe the cellular local imaging information and the intercellular structure.

## Abstract 2

This work reports the first example of a NBE-CO_2_Me-mediated palladium-catalyzed annulative π-extension reaction of bromoaromatics to construct structurally diverse polycyclic aromatic hydrocarbon (PAH)-based fluorescent materials.

## Graphical abstract

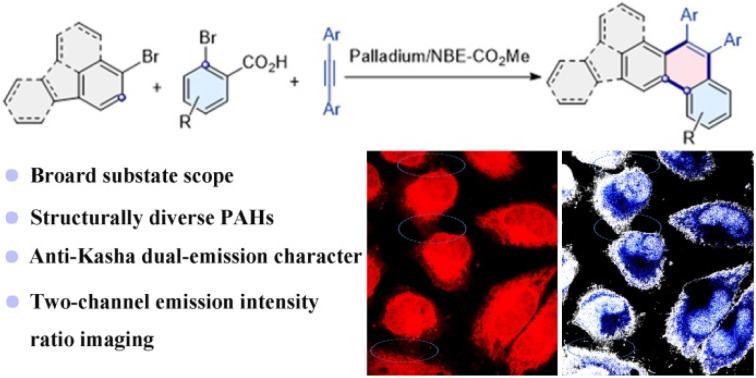

## Introduction

**Scheme 1:**
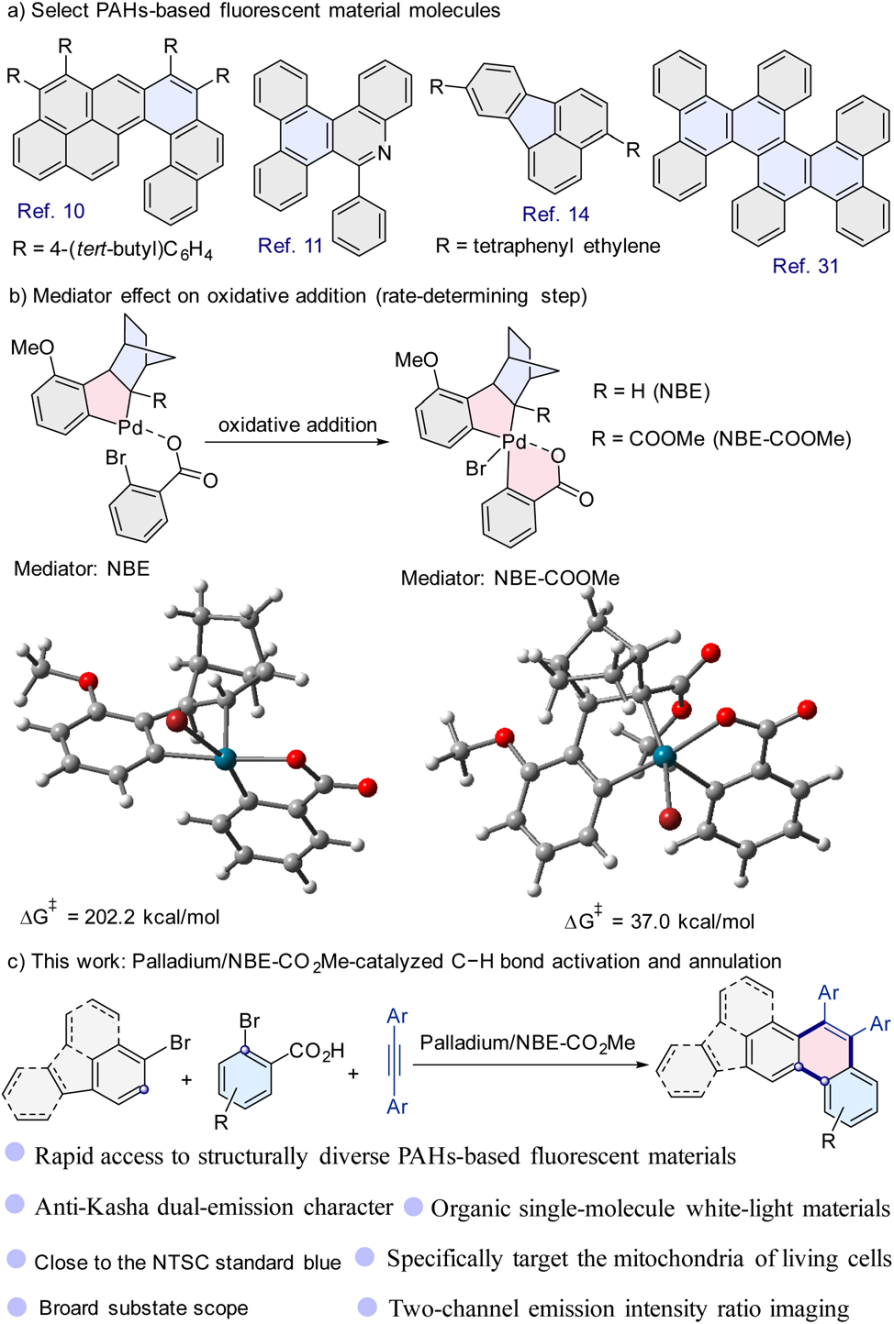
Polycyclic aromatic hydrocarbon (PAH)-based fluorescent materials and synthetic routes.

Fluorescent materials have been significantly affecting and increasing the quality of human life.^1–15^ Polycyclic aromatic hydrocarbon (PAH)-based fluorescent materials are of great interest in diverse scientific fields including biology, medicine, chemistry, and materials science due to their excellent physical and biological properties, such as long-wavelength absorption and emission, narrow HOMO–LUMO gaps, strong π–π interactions, and high mechanical strength.^5–8^ As such, PAH-based fluorescent materials have been widely used in various areas including sensors, fluorescent bioimaging probes, organic light-emitting diodes (OLEDs), memory devices, and security systems (Scheme 1a).^1–15^ Therefore, scientists have developed many methods to construct PAHs based on stepwise π-extension reactions of small arenes, the Scholl reaction, Diels–Alder cycloaddition, and Friedel–Crafts-type reactions.^16–26^ Unfortunately, most of these methods suffer from low synthetic efficiency, chemoselectivity, and product diversity.

In recent years, transition-metal-catalyzed cycloaddition reactions to construct PAHs have made great progress.^9,27–39^ It should be mentioned that Itami and co-workers disclosed annulative π-extension reactions of diiodobiaryls with aromatics.^28^ Larock and co-workers developed a palladium-catalyzed three-component cross-coupling of aryl iodide, arynes, and acetylenes.^27^ Kwong and co-workers discovered the regioselective aromatic π-extension reaction of iodoaromatics.^29,33^ Despite significant progress, these cycloaddition approaches typically require iodoaromatics, leading to limited types of products. In particular, many aryl (heteroaryl) iodides are difficult to prepare or not available (under current technology), resulting in limited PAH diversity. Thus, innovative strategies to address such a challenge are needed. One wise strategy is to use bromoaromatics instead of aryl (heteroaromatic) iodides based on the innovative catalytic system.

Density functional theory (DFT) calculations have become an effective means of assisting catalytic reactions.^40–42^ In the norbornene-mediated palladium-catalyzed aromatic π-extension reaction to construct PAHs, DFT calculations show that oxidative addition is the rate-determining step.^33^ Based on previous work,^33^ our further DFT calculations indicated that the reaction was incompatible with bromoaromatics using norbornene (NBE) as a mediator due to the high free-energy barrier (202.2 kcal mol^−1^) (Scheme 1b). In the methyl bicyclo[2.2.1]hept-2-ene-2-carboxylate (NBE-CO_2_Me), the CO_2_Me group through both the steric hindrance and electronic effect could favor the oxidative addition process of the reaction. Further calculations indicate that the free energy barrier was reduced to 37.0 kcal mol^−1^ when NBE-CO_2_Me was used as a mediator in the palladium-catalyzed aromatic π-extension reaction (Scheme 1b).

## Results and discussion

### Reaction optimization & substrate scope

To verify this strategy, NBE-CO_2_Me-mediated palladium-catalyzed C–H bond activation and annulation of bromobenzene (1a) and 2-bromobenzoic acid (2a) with 1,2-diphenylethyne (3a) was performed (Table S1†). Screening of several reaction parameters led us to the standard reaction conditions [Pd(OAc)_2_ (10 mol%), NBE-CO_2_Me (1.5 equiv.), P(2-MeC_6_H_4_)_3_ (25 mol%), K_2_CO_3_ (4.5 equiv.), and toluene (1.0 mL) at 130 °C for 72 h], affording the desired product 1-methoxy-9,10-diphenylphenanthrene (4a) in 71% yield (entry 39, Table S1†). In addition, the reaction did not work with the mediator NBE (entry 20, Table S1†), thus highlighting the indispensable role of the electron-deficient norbornene-2-carboxylate.

**Scheme 2:**
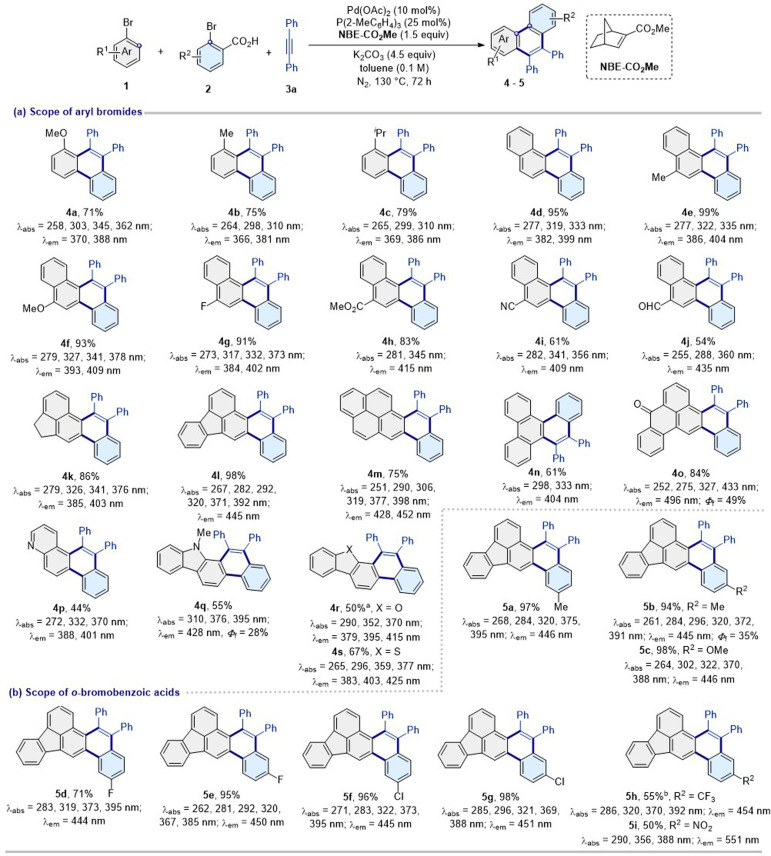
Substrate scope of bromides. Reaction conditions: 1 (0.1 mmol), 2 (0.2 mmol), 3a (0.15 mmol), Pd(OAc)_2_ (10 mol%), P(2-MeC_6_H_4_)_3_ (25 mol%), NBE-CO_2_Me (1.5 equiv.), K_2_CO_3_ (4.5 equiv.), toluene (0.1 M), N_2_, 130 °C, 72 h. ^*a*^PhDavePhos (25 mol%). ^*b*^Pd(OAc)_2_ (20 mol%), P(2-MeC_6_H_4_)_3_ (40 mol%). Test conditions for absorption and emission: absorption maximum in CH_2_Cl_2_ (1 × 10^−6^ M). Emission maximum in CH_2_Cl_2_ (5.0 × 10^−5^ M). Excitation slit: 2.5 nm, emission slit: 1.0 nm. *λ*_ex_ = 370 nm, PMT voltage = 700 V. Test parameters for quantum yield: scan slit: 0.55, fixed/offset slit: 2.5, detector: PMT-900.

**Scheme 3:**
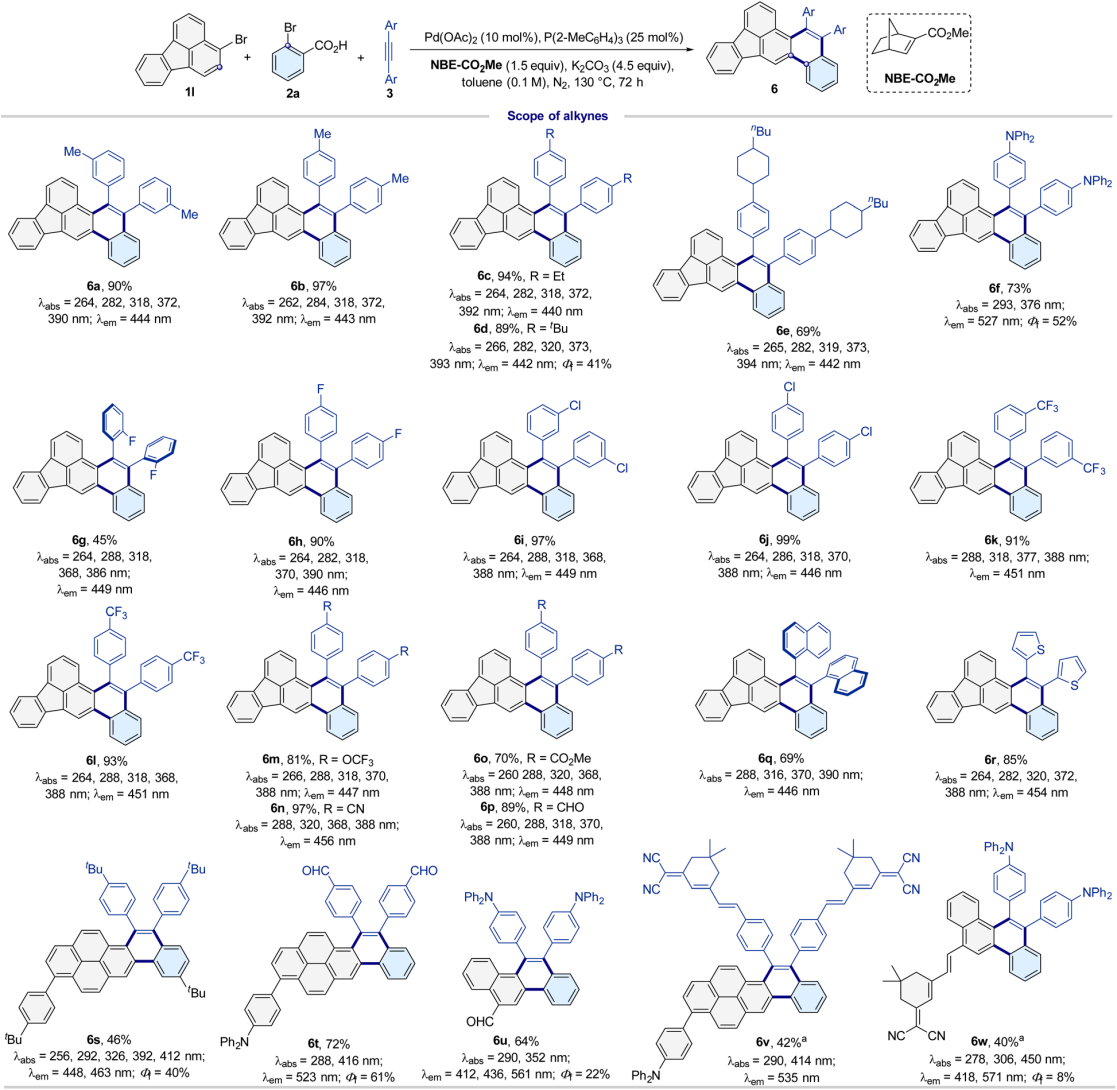
Substrate scope of alkynes. Reaction conditions: 1 (0.1 mmol), 2 (0.2 mmol), 3 (0.15 mmol), Pd(OAc)_2_ (10 mol%), P(2-MeC_6_H_4_)_3_ (25 mol%), NBE-CO_2_Me (1.5 equiv.), K_2_CO_3_ (4.5 equiv.), toluene (0.1 M), N_2_, 130 °C, 72 h. ^*a*^0.05 mmol. Test conditions for absorption and emission: absorption maximum in CH_2_Cl_2_ (1 × 10^−6^ M). Emission maximum in CH_2_Cl_2_ (5.0 × 10^−5^ M). Excitation slit: 2.5 nm, emission slit: 1.0 nm. *λ*_ex_ = 370 nm, PMT voltage = 700 V. Test parameters for quantum yield: scan slit: 0.55 nm, fixed/offset slit: 2.5 nm, detector: PMT-900.

Subsequently, the optimal catalytic system evaluated the scope for aryl bromides, *ortho*-bromide carboxylic acids, and alkynes (Schemes 2 and 3). Aryl(heterocycle) bromides with different electronic properties and steric profiles provided the corresponding PAH products in moderate to excellent yields with excellent site selectivities (4a–6w). Various functional groups such as methoxy (4a, 4f, and 5c), methyl (4b, 4e, 5a, 5b, 6a, and 6b), isopropyl (4c), *tert* butyl (6d, 6e, and 6s), naphthyl (4d–4j, 6u, and 6w), ester (4h and 6o), cyano (4i and 6n), fluoranthyl (4l and 5a–6r), 1,2-dihydroacenaphthylene (4k), pyrenyl (4m, 6s, 6t, and 6v), phenanthryl (4n), 7*H*-benzoanthracene-7-one group (4o), carbazole group (4q), dibenzofuran group (4r), dibenzothiophene group (4s), quinoline group (4p), nitro (5i), trifluoromethyl (5h, 6k and 6l), triphenylamine group (6f and 6t–6w), trifluoromethoxy (6m), thienyl (6r), and dicyanoisophorone (6v and 6w) were well tolerated in the reaction. Furthermore, the aldehyde functional group (4j, 6p, 6t,and 6u) and halides (4g, 5d–5g, and 6g–6j) were well tolerated and could be useful for further synthetic transformations. It is worth noting that π-extended PAHs 4h, 4l, 4o, 4q, and 5a–6w are difficult to obtain by other current technologies because the corresponding aryl (heteroaryl) iodides are unavailable.

**Scheme 4:**
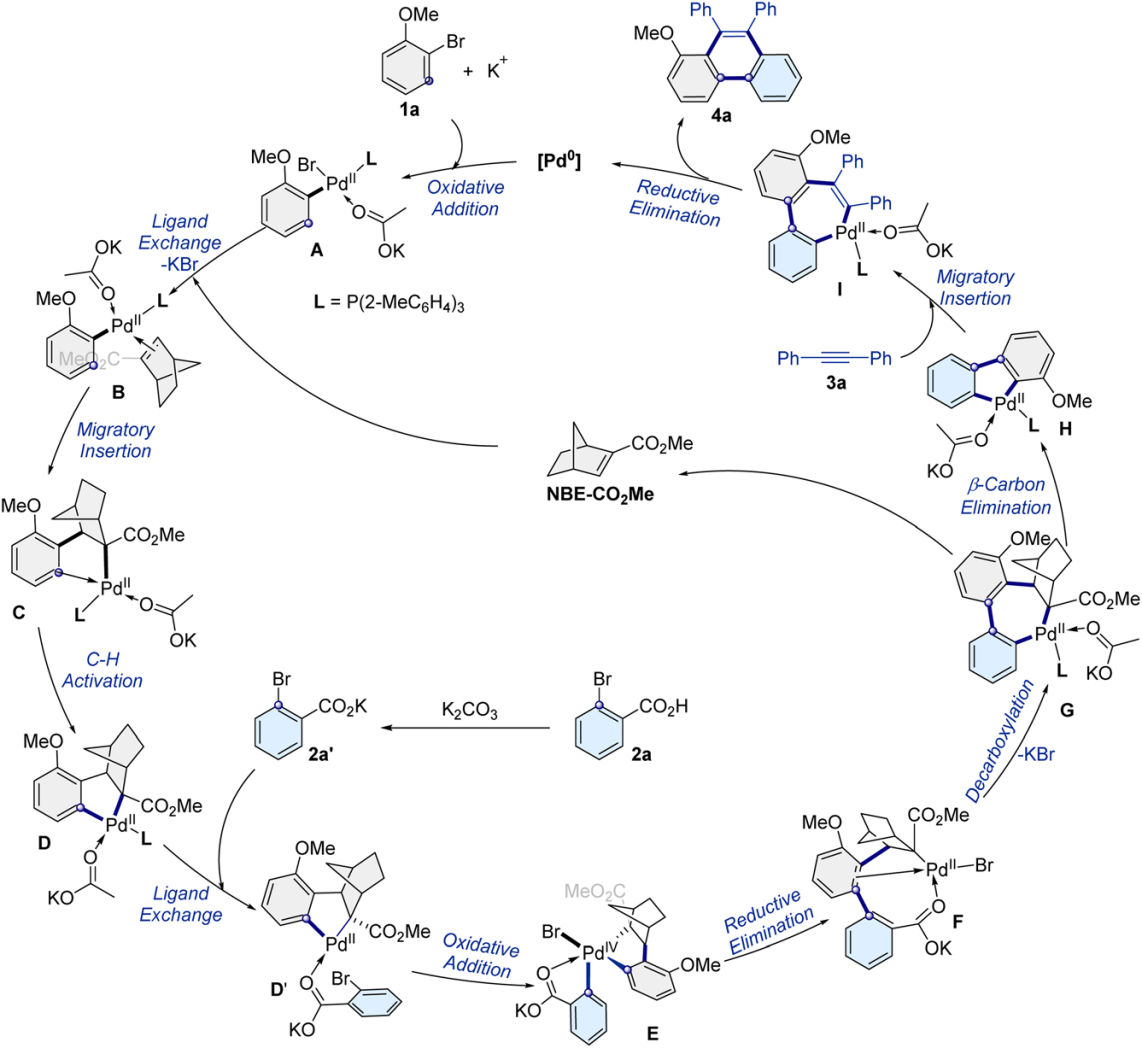
Proposed reaction mechanism.

Based on the result and previous reports,^40–48^ a possible mechanism pathway is proposed (Scheme 4). Initially, the Pd(ii) intermediate A forms through the aryl bromide oxidative addition with palladium. Subsequently, the intermediate A undergoes ligand exchange, migratory insertion, C–H bond activation, and subsequent oxidative addition to provide the intermediates B, C, D, D′, and E, respectively. Next, the Pd^IV^ species E generates the biaryl motif intermediate F by a reductive elimination process. The following decarboxylation produces intermediate G, and β-carbon elimination to afford the palladacycle intermediate H and release ligand NBE-CO_2_Me. Finally, the alkyne 3a coordinates with the palladacycle intermediate H and subsequently undergoes migratory insertion to form intermediate I, followed by a reductive elimination process to give the desired product 4a and formed palladium(0) species. Moreover, the formation of intermediates D and G was supported by high-resolution electrospray ionization mass spectrometry (HRMS) analysis (Fig. S4 and S5†). In addition, NBE-CO_2_Me plays a key role in regulating the site selectivity, with higher reactivity than alkynes in the system, and could react with palladium preferentially to form the intermediate D. Thus, the C–H insertion between alkynes and intermediate A is unfavorable and the corresponding byproduct was not detected in the protocol.

### Photophysical properties

The novel structurally diverse PAHs could possess unexpectedly excellent fluorescence properties. In particular, single-molecule dual-emission fluorescence materials are of great interest in materials science, biology, and medicine because dual-emission molecules are a candidate for single-molecule white light materials and can be used for highly accurate analysis in basic life science research.^49–51^ Subsequently, we further tested the photophysical properties of the constructed library of PAHs, and the corresponding absorption and emission maxima are summarized in Schemes 2, 3 and Table S2.† Their absorption and emission spectra are shown in Fig. S7–S13.† All PAHs (4a–6w) exhibited bright fluorescent emission and their emission wavelengths are located in the range of ultraviolet to yellow (369 nm to 571 nm) in dichloromethane (Schemes 2, 3 and Table S2†). In particular, 4o, 6f, 6s and 6t exhibit high quantum yields of 49%, 52%, 40% and 61% in CH_2_Cl_2_ solution (5.0 × 10^−5^ M). In addition, PAHs 4l, 5b–5d, and 6a display an excellent color purity with a narrow full-width at half-maximum and CIE coordinates of (0.15, 0.08), which are very close to the CIE (0.14, 0.08) of the National Television System Committee (NTSC) standard blue (Table S2†).

To our surprise, the novel PAHs 6u and 6w show distinct dual emission behavior involving a blue-light component and the orange-light component, which completely covers the whole visible range (400–700 nm) in solution (Fig. S12d and S13b†). They display bright white-light emissions with CIE_1931_ coordinates of 6u (0.31, 0.34) and 6w (0.33, 0.33), and the CIE of 6w is identical to pure white light (CIE: 0.33, 0.33) (Table S2 and Fig. S14†). Moreover, 6w also exhibited aggregation-induced emission (AIE) characteristics (Fig. S13c†). At present, the examples of PAHs describing single-molecular dual-emission materials are very rare.^8^ Furthermore, the time-resolved PL measurement demonstrates that the excited-state lifetimes of 6u and 6w are short-lived components with nanosecond order (Fig. S16–S19†), which ruled out the phosphorescence or delayed fluorescence emission.

**Fig. 1:**
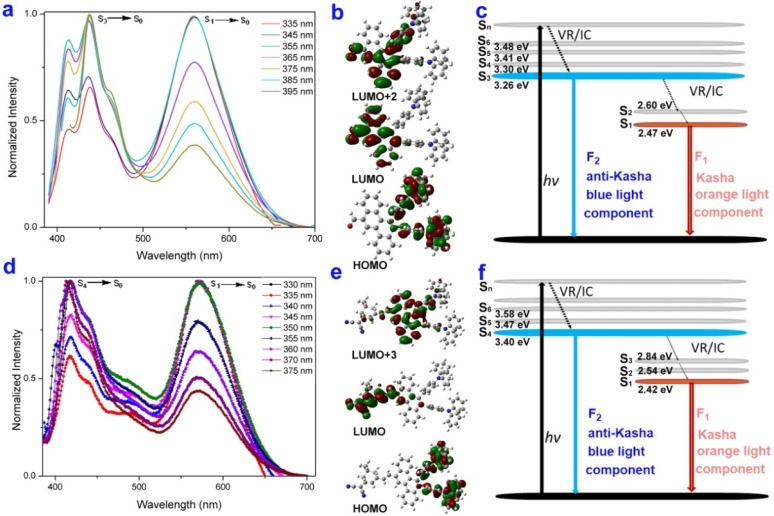
Anti-Kasha dual-emission characteristics of 6u and 6w. (a), (d) Excitation-wavelength-dependent fluorescence spectra of 6u (0.25 μM in DCM) and 6w (2.4 μM in toluene). (b) and (e) Molecular orbitals of the S_0_, S_1_, S_3_, or S_4_ states of 6u and 6w; (c) and (f) Jablonski diagram illustrating the anti-Kasha dual-emission mechanism of 6u and 6w.

To gain insight into the single-molecular white-light emissions mechanism, the anti-Kasha dual-emission characteristics of 6u and 6w were further demonstrated by the emission-wavelength-dependent excitation experiments, excitation-wavelength-dependent fluorescence experiments, and time-dependent density functional theory (TD-DFT) calculations (Fig. 1 and S15†).^52–64^ In the emission-wavelength-dependent excitation experiments of 6u, the shorter excitation wavelength peaks at ∼300 nm displayed enhanced, and the longer excitation wavelengths at ∼375 nm exhibited a slight blue shift and the intensity weakened as the emission wavelength increased (Fig. S15a†). For 6w, the shorter excitation wavelength peaks at ∼310 nm exhibited slight enhancement, the longer excitation wavelengths at ∼375 nm gradually disappeared, and a new peak appeared at 450 nm as the emission wavelength increased (Fig. S15b†). Furthermore, the relative intensity of dual emission of 6u and 6w depends to some extent on the excitation wavelength. The lower energy excitations result in the shorter wavelengths of 6u in a slight enhancement, and the intensity gradually decreases of the longer wavelengths (Fig. 1a). For 6w, lower energy excitations resulted in the shoulder peak of the shorter wavelengths a slight redshift, a decrease in intensity, and the intensity gradually enhancement of the longer wavelengths (Fig. 1d). The experimental result implied that the dual emission was from different excited states.

In addition, the theoretical calculations reveal the white light emission of 6u and 6w with the shorter wavelengths from high-lying singlet state emission (S_3_ → S_0_ for 6u, Fig. 1c; S_4_ → S_0_ for 6w, Fig. 1f), and the longer wavelengths from low-lying singlet state emission (S_1_ → S_0_). The internal conversion (IC) from the S_*n*_ to the S_*n*−1_ state is comparatively slow because of the large energy gap Δ*E* (S_*n*_ → S_*n*−1_) values (6u: 0.66 eV, 6w: 0.56 eV), which prompt S_*n*_ (6u: *n* = 3, 6w: *n* = 4) state fluorescence emission to compete with IC (Fig. 1c and f).

### Applications

Dual-emission fluorescence imaging is of great interest in biology and medical science because it can provide two-channel information and then favorable for observing cellular local imaging information, and the intercellular structure, improves the detection sensitivity, and excavates high-performance imaging reagents.^54,55,60^ The anti-Kasha dual emission molecule 6w is different from other anti-Kasha molecules in that it belongs to the dicyanoisophorone (DCI)-based fluorescent material. DCI-based fluorescent materials have excellent photophysical properties and ultra-fast intramolecular charge transfer.^65^ They could be applied in diagnosing live cell markers and have produced wonderful results in early diagnosis and treatment, promising to be used in optical imaging *in situ* and *in vivo* detection.^65^ Subsequently, the anti-Kasha dual-emission molecule 6w was fabricated as water-dispersed nanoparticles (NPs) based on Poloxamer 188 as a matrix through a thin-film hydration method (Section 4.2, ESI†). The 6w NPs exhibited apparent anti-Kasha dual-emission (*λ*_em_ = 475 nm, *λ*_em_ = 625 nm) with CIE coordinates of (0.33, 0.28) (Fig. S20c and d†), and have diameters of approximately about 145.8 nm by dynamic light scattering (DLS) measurement (Fig. S20a†). Furthermore, the cytotoxicity experiments of 6w NPs were executed and have almost no toxicity in HeLa cells (Fig. S21, Section 4.3, SI†).

**Fig. 2:**
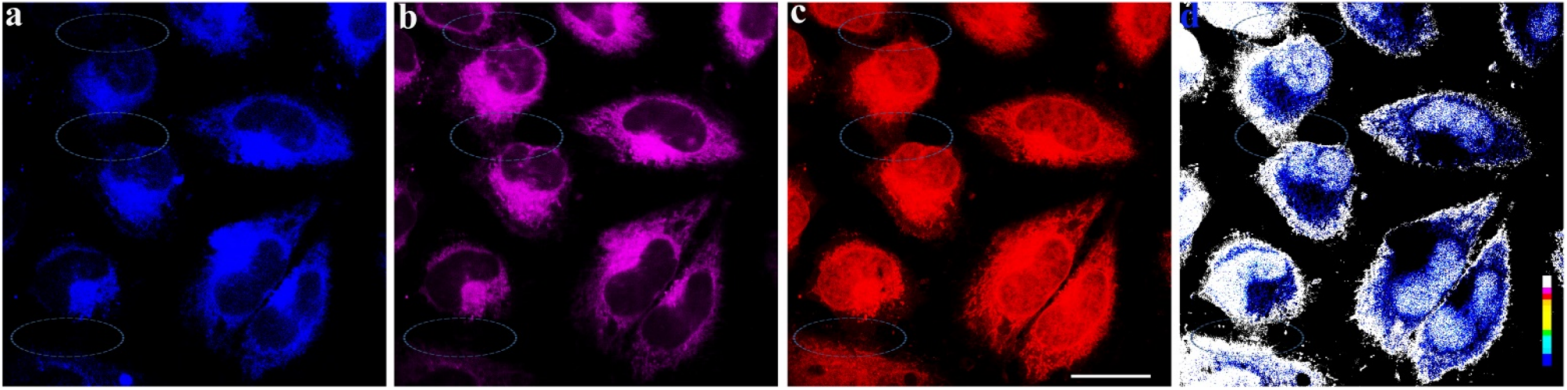
Fluorescence microscopy images of HeLa cells incubated with 6w NPs (10 μM) for 2 h at 37 °C. (a) Fluorescence microscope image from channel 1 at 425–500 nm (excitation 405 nm). (b) Fluorescence microscope image from channel 2 at 500–700 nm (excitation 405 nm). (c) Fluorescence microscope image from channel 2 at 508–700 nm (excitation 488 nm). (d) The emission intensity ratio of (a) and (c) of HeLa cells (image generation by Image J software). Elliptic: mark out the intercellular structure and cellular local imaging information. The scale bar is 25.0 μm.

In addition, the two-channel emission intensity ratio imaging of 6w NPs was carried out in HeLa cells (Fig. 2 and S22†). Confocal laser scanning microscopy displayed signal distributions in the blue, purple, and red regions when excited at 405 nm (for S_4_ and S_1_) and 488 nm (for S_1_) (Fig. 2a–c and S22a–c†). Then, the emission intensity ratio images were prepared based on the fluorescence imaging from the S_1_ and S_4_ emission channels by the software Image J (Fig. 2d and S22d†). The cellular local imaging information and the intercellular structure can be observed in Fig. 2d and S22d.† Nevertheless, these structures could not be watched constantly on both S_1_ and S_4_ emission channels (Fig. 2a–c and S22a–c†). These imaging results indicate that the emission intensity ratio image obtained from two channels involves more comprehensive information and features of HeLa cells than one-channel images with only reflecting the cellular textures (Fig. 2 and S22†).

**Fig. 3:**
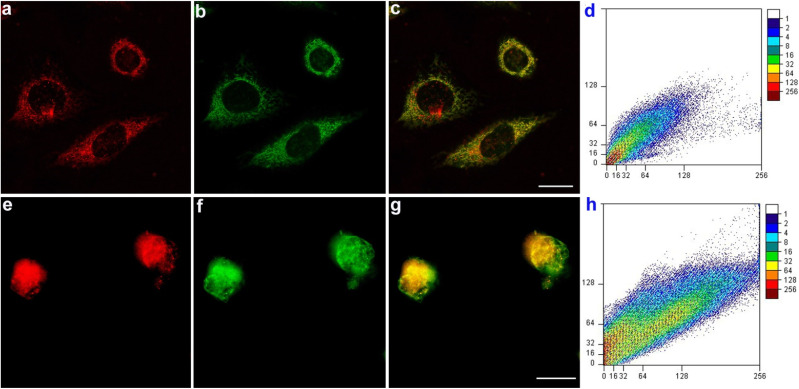
The co-staining experiments. (a) Fluorescent image of HeLa cells cultured with 6w NPs (10 μM) (*λ*_ex_ = 552 nm, *λ*_em_ = 575–700 nm). (b) Fluorescent image of HeLa cells with Mito-Tracker Green (*λ*_ex_ = 488 nm, *λ*_em_ = 508–540 nm). (c) Merged image of (a) and (b). (d) The Pearson correlation coefficient *r* = 0.87. (e) Fluorescent image of HeLa cells cultured with 6w NPs (5 μM) (*λ*_ex_ = 552 nm, *λ*_em_ = 570–600 nm). (f) Fluorescent image of HeLa cells with Mito-Tracker Red (*λ*_ex_ = 633 nm, *λ*_em_ = 650–700 nm). (g) Merged image of (e) and (f). (h) The Pearson correlation coefficient *r* = 0.85; the scale bar is 25.0 μm.

Furthermore, the co-staining experiments of HeLa cells were executed with 6w NPs and the commercially available 3,3′-dioctadecyloxacarbocyanine perchlorate (DiO) (cell membrane-specific tracker), Lyso-Tracker green (lysosome-specific tracker), Mito-Tracker green, and Mito-Tracker Deep Red 633, (mitochondria-specific tracker), respectively (Fig. 3, S23 and S24†). The experimental results reveal that 6w NPs have selectively accumulated in mitochondria with high Pearson's correlation coefficients (*R*) (0.87/0.85), and show relatively low Pearson's coefficient with other probes (lysosome, and cell membrane, 0.44/0.57).

## Conclusions

In conclusion, palladium/norbornene catalyzed C–H bond activation and annulation of bromo(hetero)aromatics has been developed to construct a library of structurally diverse polycyclic aromatic hydrocarbons using NBE-CO_2_Me as the cooperative catalyst for the first time. This approach shows a broad substrate scope and provides straightforward access to PAHs, which opens up a new route for the rapid screening of high-performance fluorescent functional materials. Novel organic single-molecule white-light materials based on PAHs with anti-Kasha dual-emission characteristics have been developed for the first time herein. Furthermore, the anti-Kasha dual-emission molecules could provide two-channel information and are favorable for observing cellular local imaging information, which involves more comprehensive information and cell image features than one-channel images that only reflect the cellular textures. This work represents the first example of a palladium/norbornene catalyzed C–H bond activation and annulation of bromo(hetero)aromatics to construct PAHs with fluorescent emission, unlocking an opportunity to discover organic single-molecular white-light-emitting materials with anti-Kasha dual-emission characteristics and two-channel emission intensity ratio imaging materials.

## Data availability

All experimental data associated with this work are provided in the ESI.†

## Author contributions

C. Z., X. Y. and L. G. performed the experiments and analyzed the data. B. L. designed and directed the project and wrote the manuscript. All authors contributed to discussions.

## Conflicts of interest

There are no conflicts to declare.

## Supplementary Material

SC-016-D5SC00617A-s001
