# Supplementary material for: Palladium/norbornene-catalyzed C–H bond activation and annulation to construct polycyclic aromatic hydrocarbon-based fluorescent materials
Source: Chem Sci. 2025 Mar 18;16(16):6755–62. doi: 10.1039/d5sc00617a (PMC11934061; doi:10.1039/d5sc00617a)
Supplement: SC-016-D5SC00617A-s001 [file SC-016-D5SC00617A-s001.pdf]

Supplementary Information (SI) for Chemical Science.
This journal is © The Royal Society of Chemistry 2025

Supporting Information

Palladium/Norbornenes-Catalyzed C−H Bond Activation and Annulation to Construct Polycyclic Aromatic Hydrocarbons-based Fluorescent Materials

Chunlin Zhou, Xianhui Yang, Lian Gou, Bijin Li*

Chongqing Key Laboratory of Natural Product Synthesis and Drug Research, School of Pharmaceutical Sciences, Chongqing University, Chongqing 401331, P. R. China.

E-mail: bijinli@cqu.edu.cn

Table of contents

1. General Information S3

2. DFT Calculation S3

3. Experimental Section S14

3.1 Optimization of the reaction conditions S14

3.2 General procedures for synthesis of products..S16

3.3 Characterization of products...S17

3.4 Mechanistic Studies...S41

3.5 General procedures for the synthesis of substrates.......................................S43

4. Photophysical properties and applications of products. S45

4.1. The absorption and fluorescence properties of products S45

4.2. Preparation and characterization of **6w** NPs...S54

4.3. Cell imaging experiments...S55

5. References S57

6. NMR spectrum of compounds S59

1. General Information

Unless otherwise noted, commercial available reagents were purchased from commercial suppliers (such as Energy Chemical, Bide Pharmatech Ltd. and Adamas) and used as received. Solvents were generally dried over 4Å molecular sieves. Purification of products was performed by flash column chromatography using silica gel. ^{1}H and ^{13}C NMR spectra were recorded on a Agilent 400MR DD2 spectrometer (400 MHz and 101 MHz, respectively). Chemical shifts are reported parts per million (ppm) referenced to $CDCl_3$ (δ 7.26 ppm), tetramethylsilane (TMS, δ 0.00 ppm) for ^{1}H NMR; $CDCl_3$ (δ 77.16 ppm) for ^{13}C NMR. The following abbreviations (or combinations thereof) were used to explain multiplicities: s = singlet, d = doublet, t = triplet, q = quartet, dd = doublet of doublet, td = triplet of doublet and m = multiplet. To distinguish, some ^{13}C NMR chemical shifts retain two decimal places. High-resolution mass spectra (HRMS) were obtained on an Agilent 6546 Q-TOF LC/MS system with an ESI source at the School of Pharmaceutical Sciences of Chongqing University. UV−vis spectra were recorded on Agilent Cary60 spectrometer. Fluorescence spectra were collected on HITACHI F-700003040428 Fluorescence Spectrometer or Agilent Technologies Cary Eclipse Fluorescence Spectrometer. The absolute quantum yields were taken using Edinburgh Instruments FLS1000 fluorescence spectrometer with a calibrated integrating sphere system. The excited-state lifetimes were performed using Edinburgh Instruments FLS1000.

2. DFT Calculation

All calculations were performed using the Gaussian 16, Revision A.03.[S1] Geometries were optimized in toluene with the SMD solvation model by the B3-LYP-D3 functional and a basis set of 6-31G(d). Vibrational frequencies were calculated at the same level and check the optimized structure. Solvation energies were computed through a self-consistent reaction field using the SMD model. Calculations were performed at the M06/SDD-6-31+G(d,p)-SMD(toluene) level of

theory.

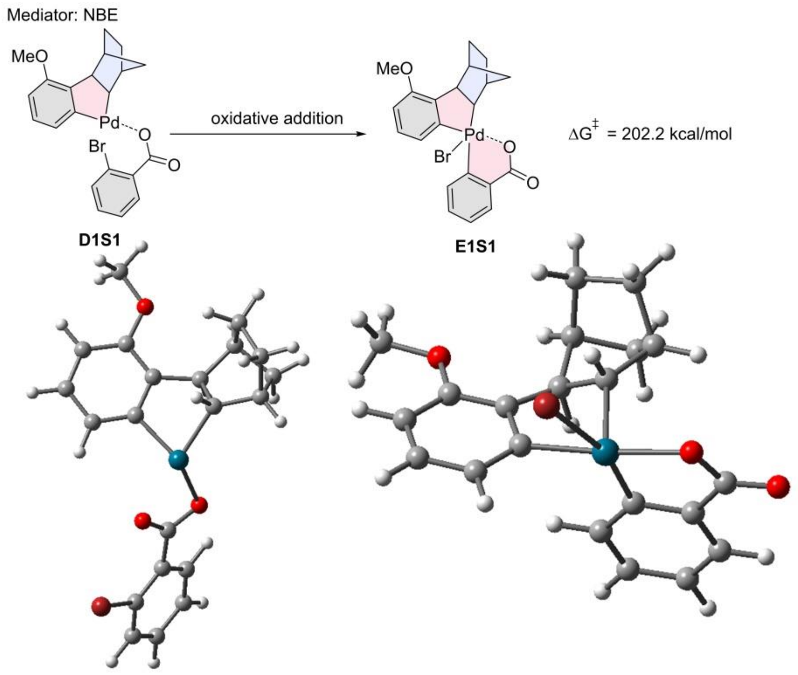

Fig. S1. DFT calculates the free-energy barrier of the reaction of the rate-determining step using norbornene (NBE) as a mediator.

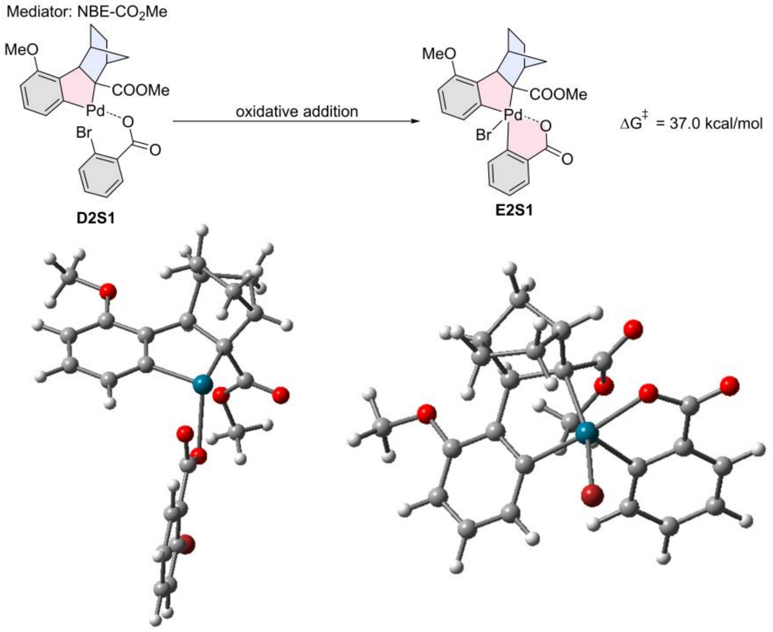

Fig. S2. DFT calculates the free-energy barrier of the reaction of the rate-determining step using methyl bicyclo[2.2.1]hept-2-ene-2-carboxylate (NBE-CO_2Me) as a mediator.

Cartesian coordinates of the structures

D1S1

C	-2.00716	-1.35636	-0.32571
C	-2.62379	3.31508	1.17352
C	-1.44274	3.57769	0.45362
C	-0.78516	2.55658	-0.25536
C	-1.3515	1.27305	-0.23643
C	-2.52727	0.97071	0.46851
C	-3.16388	2.01622	1.18084
C	-4.26955	-0.09583	-1.79398
C	-4.36042	-0.71481	-0.37102
C	-3.0324	-0.44225	0.42772
C	-3.22945	-1.01968	-2.52092
C	-2.83758	-2.05852	-1.42299
C	-4.20128	-2.2309	-0.67786
Pd	-0.46398	-0.24275	-1.22047
C	5.80371	0.58778	0.25377
C	6.3287	1.33408	-0.81154
C	5.49278	1.7191	-1.87301
C	4.1441	1.35166	-1.85307
C	3.58989	0.59818	-0.78825
C	4.44786	0.22077	0.26925
Br	3.88601	-0.8049	1.81801
C	2.13653	0.27648	-0.89713
O	1.5118	-0.42133	0.00047
O	1.46732	0.71723	-1.92283
O	-4.32008	1.658	1.86568
C	-5.02381	2.64445	2.66166
C	-1.14855	-2.26722	0.47886
O	-0.59014	-3.28981	0.03156
O	-0.98005	-1.85739	1.77622
C	-0.00882	-2.58372	2.59904
H	-3.10802	4.12514	1.72141
H	-1.03041	4.59153	0.44866
H	0.13457	2.76111	-0.80917
H	-3.95512	0.96144	-1.7699
H	-5.25475	-0.13325	-2.29298
H	-5.25731	-0.41761	0.19181
H	-3.18025	-0.80996	1.45888
H	-3.67358	-1.53589	-3.39151
H	-2.35469	-0.45733	-2.90028
H	-2.36452	-2.97384	-1.80549
H	-4.99541	-2.63423	-1.3326
H	-4.12848	-2.86048	0.22702
H	6.44959	0.28661	1.08199
H	7.38694	1.61061	-0.80635
H	5.88987	2.30095	-2.70906
H	3.47111	1.63703	-2.66385

H	-5.87751	2.11178	3.10652
H	-4.37785	3.04563	3.4655
H	-5.39403	3.47696	2.03365
H	-0.07017	-2.11984	3.59367
H	-0.26983	-3.65424	2.64583
H	1.00048	-2.46321	2.17172

E1S1

C	0.60361	1.62205	-0.00889
C	3.48631	-2.36522	0.80719
C	2.38377	-3.16964	0.46346
C	1.17873	-2.59879	0.02566
C	1.0719	-1.19731	-0.07192
C	2.17037	-0.37298	0.25384
C	3.37544	-0.97004	0.70856
C	2.38703	1.13015	-2.39376
C	2.79602	1.81789	-1.06553
C	2.06851	1.12735	0.1535
C	0.88558	1.53913	-2.54396
C	0.60569	2.45328	-1.29897
C	2.00396	3.15085	-1.14219
Pd	-0.59934	-0.13198	-0.63962
C	-1.60308	-1.48091	1.92641
C	-2.57248	-1.60902	2.94384
C	-3.72629	-0.80482	2.94145
C	-3.93008	0.13051	1.9148
C	-2.97841	0.27221	0.89065
C	-1.81784	-0.51882	0.93768
Br	-1.4884	-2.0978	-2.03715
C	-3.17227	1.21657	-0.26042
O	-2.1743	1.18151	-1.14107
O	-4.18487	1.93994	-0.37743
O	4.38637	-0.08247	1.04782
C	5.65905	-0.58078	1.53792
C	-0.12069	2.27689	1.15194
O	-0.82756	3.27517	0.95114
O	-0.00058	1.85672	2.44496
C	0.81624	0.78412	3.00694
H	4.41278	-2.83284	1.14485
H	2.46791	-4.25817	0.54055
H	0.33888	-3.24361	-0.23699
H	2.99597	1.51954	-3.22872
H	2.53184	0.03692	-2.36651
H	3.8807	1.89145	-0.90506
H	2.5145	1.56997	1.06391
H	0.2063	0.66473	-2.63031
H	0.69442	2.12375	-3.46051
H	-0.27463	3.09478	-1.39812
H	2.25141	3.76804	-2.02447
H	2.08253	3.77537	-0.23482

H	-0.71996	-2.12124	1.93069
H	-2.41787	-2.35063	3.73362
H	-4.47239	-0.91882	3.73301
H	-4.82445	0.7597	1.87173
H	6.26806	0.31355	1.74015
H	5.53091	-1.15817	2.47345
H	6.16455	-1.20602	0.77785
H	0.35185	0.55982	3.97921
H	0.81245	-0.11562	2.3773
H	1.8489	1.13891	3.16932

D2S2

C	2.48081	-1.28104	0.65082
C	2.142	3.34026	-0.2073
C	0.94322	3.27496	0.49893
C	0.38519	2.04918	0.86589
C	1.08443	0.91196	0.50708
C	2.26263	0.94621	-0.22676
C	2.80714	2.17248	-0.58641
C	5.13023	-0.79989	0.10779
C	4.11563	-0.91813	-1.06012
C	2.72982	-0.42708	-0.598
C	4.75448	-1.99407	1.05818
C	3.41186	-2.50478	0.45114
C	3.78835	-2.44234	-1.05736
Pd	0.47828	-0.95681	1.01287
C	-5.36954	0.04562	-1.22582
C	-5.38847	-0.58213	-2.46163
C	-4.25903	-1.25886	-2.90984
C	-3.13227	-1.29576	-2.10991
C	-3.10488	-0.6712	-0.85892
C	-4.23824	0.00658	-0.42251
Br	-4.36262	0.93044	1.24632
C	-1.83234	-0.79909	-0.10721
O	-0.83895	-1.40762	-0.69211
O	-1.66822	-0.35285	1.07866
O	3.98831	2.12572	-1.30167
C	4.59442	3.38028	-1.69928
H	2.80343	-0.73151	1.54812
H	2.54416	4.30833	-0.47033
H	0.43302	4.19363	0.76139
H	-0.56393	1.97988	1.37985
H	5.03555	0.17277	0.59551
H	6.15519	-0.9028	-0.25684
H	4.45105	-0.48471	-1.99898
H	2.02567	-0.76711	-1.37403
H	5.51064	-2.78367	1.00429
H	4.64559	-1.68334	2.10008
H	3.07436	-3.46627	0.83579
H	2.96172	-2.69515	-1.72566

H	4.65552	-3.06859	-1.28172
H	-6.2388	0.57867	-0.868
H	-6.28247	-0.53917	-3.07052
H	-4.25994	-1.75025	-3.87373
H	-2.23337	-1.80702	-2.42236
H	5.49738	3.10349	-2.24138
H	3.93369	3.95685	-2.35554
H	4.86043	3.99253	-0.83079

E2S2

C	0.06398	0.40018	1.20071
C	-3.74184	0.00752	-1.68353
C	-3.14362	-1.12643	-2.21022
C	-1.85103	-1.48933	-1.83168
C	-1.14377	-0.68022	-0.91686
C	-1.76417	0.47488	-0.39466
C	-3.04449	0.82885	-0.77884
C	-1.58029	1.87853	2.76817
C	-1.05833	2.46479	1.43001
C	-0.82109	1.32984	0.39648
C	-0.30759	1.21225	3.40637
C	0.74143	1.29098	2.24932
C	0.44619	2.73358	1.75276
Pd	0.78811	-0.99752	-0.14916
C	1.35702	1.21011	-2.17676
C	2.25308	2.1125	-2.75451
C	3.56002	2.22059	-2.28181
C	3.98704	1.41407	-1.23414
C	3.10443	0.50994	-0.66463
C	1.79958	0.42194	-1.12559
Br	-0.93973	-2.93402	0.62152
C	3.5287	-0.39743	0.44622
O	2.55794	-1.25744	0.80134
O	4.64384	-0.3582	0.9534
O	-3.54174	1.99852	-0.25507
C	-4.87827	2.42454	-0.63205
H	-0.48836	-0.40932	1.68043
H	-4.74873	0.26186	-1.98295
H	-3.69209	-1.74117	-2.91259
H	-1.39402	-2.3778	-2.24597
H	-2.38159	1.15733	2.59044
H	-1.96779	2.671	3.4125
H	-1.64015	3.29099	1.03111
H	-0.18857	1.80855	-0.36042
H	0.05337	1.79763	4.25649
H	-0.48563	0.18667	3.73691
H	1.75861	1.03355	2.53179
H	1.02514	3.01807	0.87124
H	0.5903	3.46844	2.54774
H	0.34225	1.13239	-2.54714

H	1.92453	2.73197	-3.58032
H	4.24018	2.92947	-2.73657
H	4.9945	1.45324	-0.83633
H	-5.03838	3.35965	-0.09848
H	-4.9554	2.59799	-1.71019
H	-5.63277	1.69316	-0.32564

The time-dependent density functional theory (TDDFT) calculations of the excitation energies were calculated at the optimized geometries of the ground states. Calculations were performed at the B3LYP-6-31+G(d) (**6u**) and MPWLPW91-6-31+G(d) (**6w**) level of theory.

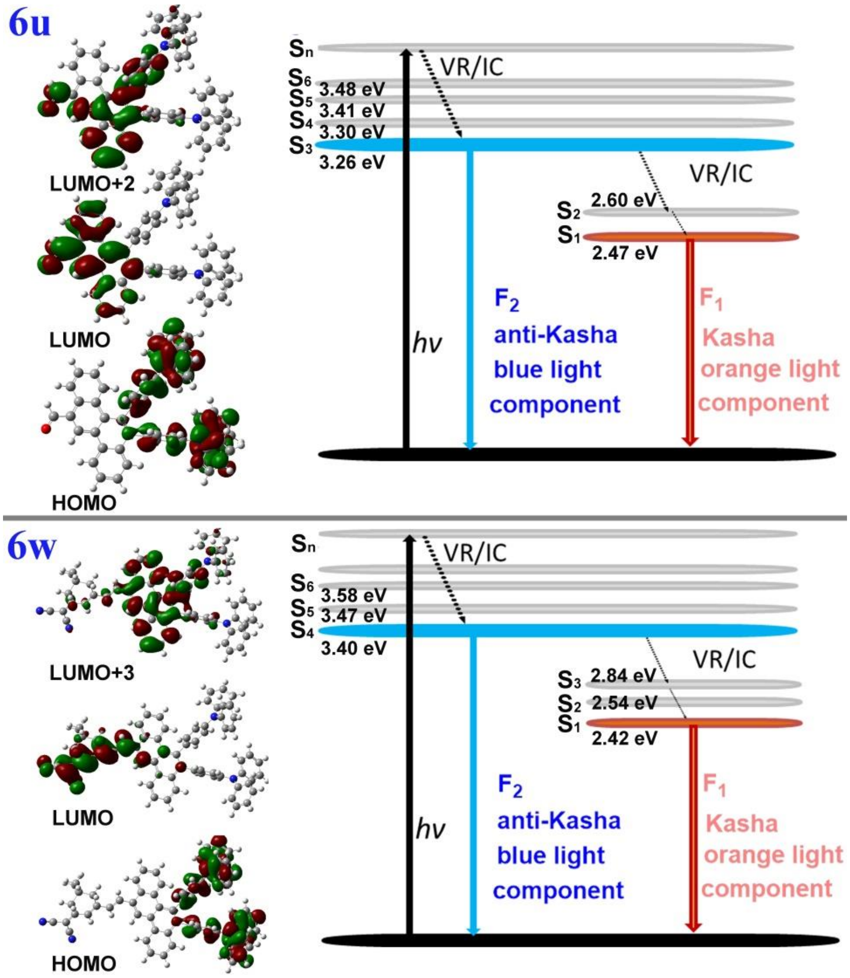

Fig. S3. Molecular orbitals of the S_0, S_1, S_3, or S_4 states of **6u** and **6w**; Jablonski diagram illustrating the anti-Kasha dual-emission mechanism of **6u** and **6w**.

Cartesian coordinates of the structures

6u

C	-6.74854	-0.45937	0.38766
C	-6.06282	0.74942	-0.00307
C	-6.09785	-1.66765	0.29141
C	-4.72465	-1.78524	-0.0533
C	-3.95104	-0.60275	-0.23434
C	-4.66898	0.67204	-0.35517
C	-2.50155	-0.72984	-0.28322
C	-4.11452	-3.09744	-0.16862
C	-4.87573	-4.29631	-0.13281
C	-4.27338	-5.53575	-0.25993
C	-2.87512	-5.63039	-0.42192
C	-2.10752	-4.4812	-0.4634
C	-2.70239	-3.19379	-0.35011
C	-1.90697	-1.98796	-0.34839
C	-1.59638	0.45695	-0.11741
C	-0.41248	-2.13113	-0.35468
C	-4.07113	1.84667	-0.89147
C	-4.76469	3.03921	-1.00534
C	-6.10599	3.12397	-0.58704
C	-6.74103	1.9932	-0.10847
C	-1.57773	1.16175	1.10019
C	-0.70918	2.2337	1.30415
C	0.18438	2.63185	0.29221
C	0.17122	1.93359	-0.93
C	-0.70677	0.8674	-1.12682
C	0.28551	-2.42114	-1.54085
C	1.67444	-2.55842	-1.54864
C	2.41345	-2.39877	-0.36175
C	1.7207	-2.11991	0.83133
C	0.33091	-1.99833	0.83123
N	1.08884	3.71395	0.50534
C	1.35171	4.64878	-0.54328
C	2.66226	5.10345	-0.78253
C	2.91249	6.02465	-1.80268
C	1.8692	6.49454	-2.60992
C	0.56572	6.0379	-2.37877
C	0.30327	5.12975	-1.35014
C	1.75306	3.84969	1.76527
C	2.27722	2.71959	2.42028
C	2.91889	2.85701	3.65378
C	3.06335	4.11846	4.24444
C	2.55005	5.24497	3.58988
C	1.89219	5.11545	2.36412
N	3.83531	-2.51319	-0.37077
C	4.50961	-3.17672	0.70101
C	5.69958	-2.64577	1.2333
C	6.35957	-3.30311	2.27458

C	5.83932	-4.48645	2.81305
C	4.65128	-5.01223	2.29052
C	3.99338	-4.37113	1.23793
C	4.58914	-1.94198	-1.444
C	5.67474	-2.64136	-2.00323
C	6.41452	-2.07563	-3.04473
C	6.07646	-0.81638	-3.55556
C	4.9916	-0.12227	-3.00571
C	4.25615	-0.67258	-1.95291
C	-8.14139	-0.4775	0.87018
O	-8.77053	-1.52159	1.1327
H	-6.67609	-2.54595	0.54836
H	-5.95193	-4.25237	-0.02595
H	-4.87992	-6.43528	-0.23738
H	-2.40339	-6.60323	-0.51489
H	-1.03428	-4.55382	-0.58313
H	-3.05527	1.81147	-1.24815
H	-4.27188	3.90711	-1.43147
H	-6.64741	4.06103	-0.66699
H	-7.78837	2.05811	0.15863
H	-2.26106	0.87344	1.89276
H	-0.71682	2.76496	2.24895
H	0.85308	2.22633	-1.72029
H	-0.69342	0.33886	-2.07372
H	-0.26647	-2.54626	-2.46775
H	2.19204	-2.78553	-2.47359
H	2.27506	-1.99736	1.75478
H	-0.18351	-1.7747	1.75997
H	3.47544	4.73291	-0.16885
H	3.92873	6.3658	-1.97428
H	2.06833	7.20523	-3.40513
H	-0.25384	6.40009	-2.99178
H	-0.70983	4.79142	-1.16424
H	2.17981	1.74277	1.95981
H	3.31887	1.97659	4.14711
H	3.56775	4.22191	5.19943
H	2.64927	6.22783	4.04002
H	1.48406	5.98814	1.86691
H	6.09938	-1.72278	0.82901
H	7.27642	-2.88089	2.67416
H	6.35127	-4.99068	3.62591
H	4.24044	-5.93278	2.69342
H	3.08194	-4.79034	0.82697
H	5.93147	-3.62224	-1.61942
H	7.2491	-2.62736	-3.46613
H	6.64921	-0.38292	-4.36871
H	4.72373	0.85824	-3.38701
H	3.42564	-0.1253	-1.52103
H	-8.63759	0.49209	1.02219

6w

C	-11.32954	-1.65787	-0.19484
C	-9.02175	-0.83752	-0.10565
C	-10.4112	-0.79448	0.30746
C	-10.77766	0.22029	1.36132
C	-9.98681	1.53624	1.21941
C	-8.47945	1.20879	1.21012
C	-8.10616	0.06015	0.30123
C	-6.71677	-0.1045	-0.13436
C	-5.68308	0.6538	0.23614
C	2.47195	-2.03257	0.80061
C	3.82193	-2.33078	0.89196
C	4.55434	-2.64839	-0.24852
C	3.9013	-2.66531	-1.47802
C	2.55545	-2.34851	-1.56235
C	1.91514	1.0101	-1.27217
C	2.88645	1.9653	-1.02236
C	2.91794	2.63966	0.19537
C	1.95319	2.33577	1.1511
C	0.99244	1.37117	0.89706
C	-3.95334	2.80042	-0.72385
C	-3.17899	3.82985	-1.17855
C	-1.8347	3.58787	-1.48482
C	-1.29294	2.34588	-1.29231
C	1.8195	-2.02013	-0.42763
C	0.95382	0.69264	-0.31749
C	0.35899	-1.6842	-0.52342
C	-0.57947	-2.77976	-0.54842
C	-0.14575	-4.12753	-0.59583
C	-1.04018	-5.1599	-0.59176
C	-2.41655	-4.89011	-0.53968
C	-2.85902	-3.59855	-0.47976
C	-1.95522	-2.50773	-0.47262
C	-0.07115	-0.38527	-0.53301
C	-2.04822	1.265	-0.7745
C	-1.49726	-0.0817	-0.58539
C	-2.40186	-1.13175	-0.42901
C	-3.77841	-0.84363	-0.16936
C	-3.4208	1.50601	-0.53004
C	-4.28083	0.4112	-0.15648
C	-10.30282	2.44042	2.42148
C	-10.39023	2.26625	-0.07579
C	-10.98352	-2.65168	-1.16343
N	-10.68715	-3.44359	-1.9402
C	-12.69679	-1.63551	0.22104
N	-13.79236	-1.60664	0.56404
N	5.94024	-2.94567	-0.16277
C	6.84977	-2.361	-1.08698
C	7.83728	-3.13647	-1.68995

C	8.72919	-2.56295	-2.58291
C	8.63908	-1.21503	-2.89979
C	7.65063	-0.4424	-2.30701
C	6.76601	-1.00623	-1.40061
C	6.4233	-3.80248	0.86431
C	7.54138	-3.446	1.61465
C	8.01375	-4.28811	2.60954
C	7.36979	-5.48671	2.88158
C	6.25095	-5.84033	2.14086
C	5.78411	-5.01047	1.13333
N	3.91824	3.61284	0.46028
C	4.62244	3.58859	1.69538
C	4.85204	4.76859	2.39928
C	5.54737	4.74407	3.59825
C	6.00809	3.54392	4.12053
C	5.77255	2.36621	3.42573
C	5.09224	2.38553	2.21793
C	4.23342	4.59189	-0.52135
C	3.21822	5.29055	-1.17056
C	3.52612	6.23734	-2.13522
C	4.84827	6.51299	-2.45365
C	5.86175	5.82504	-1.80171
C	5.55967	4.865	-0.84825
H	-8.72435	-1.6296	-0.76429
H	-10.55589	-0.22291	2.3307
H	-11.83962	0.42402	1.3474
H	-8.15465	0.96466	2.21997
H	-7.93191	2.09791	0.91975
H	-6.54418	-0.92156	-0.81135
H	-5.8449	1.49559	0.88307
H	1.92737	-1.78654	1.69155
H	4.30895	-2.31742	1.84678
H	4.44892	-2.91912	-2.36387
H	2.07164	-2.36357	-2.52092
H	1.91329	0.50178	-2.21613
H	3.62268	2.18729	-1.7693
H	1.96097	2.84989	2.09165
H	0.25817	1.15198	1.64851
H	-4.99658	2.96868	-0.54857
H	-3.60285	4.80375	-1.32954
H	-1.22643	4.37346	-1.88963
H	-0.27755	2.19759	-1.56976
H	0.90195	-4.33724	-0.63635
H	-0.69337	-6.17417	-0.63327
H	-3.12235	-5.69797	-0.55292
H	-3.91392	-3.42544	-0.46382
H	-4.4242	-1.64714	0.10978
H	-10.05759	1.95208	3.35858
H	-9.73605	3.36371	2.36613
H	-11.35572	2.69883	2.44328

H	-9.86896	3.21419	-0.15323
H	-10.15971	1.68761	-0.96151
H	-11.45483	2.47257	-0.07943
H	7.90331	-4.18069	-1.45671
H	9.48584	-3.17286	-3.03806
H	9.32633	-0.77492	-3.59596
H	7.57284	0.6024	-2.53962
H	6.01007	-0.40231	-0.93873
H	8.03596	-2.51613	1.41485
H	8.87786	-4.00114	3.17758
H	7.73346	-6.13364	3.65609
H	5.74691	-6.76712	2.33758
H	4.92715	-5.29339	0.55471
H	4.48784	5.69685	2.00587
H	5.71805	5.66171	4.1279
H	6.54043	3.52675	5.05164
H	6.12729	1.43157	3.81601
H	4.92127	1.47301	1.6817
H	2.19606	5.08995	-0.91665
H	2.73328	6.7669	-2.62794
H	5.08445	7.25083	-3.19555
H	6.88831	6.02636	-2.0412
H	6.3456	4.32889	-0.35412

3. Experimental Section

3.1 Optimization of the reaction conditions

The oven-dried Schlenk tube (10 mL) equipped with a stirring bar was charged with substrates **1a** (0.1 mmol, 1.0 equiv), **2a** (0.2 mmol, 2.0 equiv), **3a** (0.15 mmol, 1.5 equiv), $Pd(OAc)_2$ (10 mol%), ligand (25 mol%) and base (2.5-4.5 equiv) in the air. Followed by the mediator smNBE (1.5 equiv) and the anhydrous solvent (0.1 M) was added via syringe under nitrogen flow. Then the tube was evacuated and back-filled with N_2 for three times. Finally, the reaction mixture was stirred at 130 ℃ for 24-72 h and then cooled to room temperature. Upon completion of the reaction, the mixture was diluted with 5 mL EtOAc and filtered through a short pad of Celite and the Celite pad were washed with an additional EtOAc (10 mL × 5). The filtrate was concentrated *in vacuo* to give the crude product. The crude ^1H NMR spectrum was taken using CH_2Br_2 (0.1 mmol) as internal standard.

Table S1. Screening of the reaction conditions.

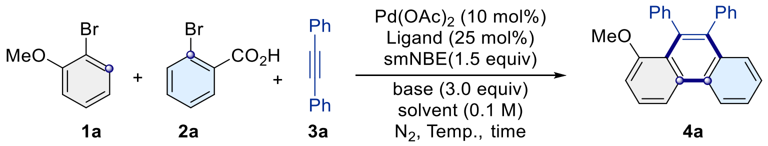

NO.	Ligand (25 mol%)	smNBE (1.5 eq.)	Base (3.0 eq.)	Solvent (0.1M)	Temp.	time	Yield[a] (%)
1	L1	N5	K_2CO_3	DMF	130 °C	24 h	n.d.
2	L1	N1	K_2CO_3	toluene	130 °C	24 h	33
3	L2	N1	K_2CO_3	toluene	130 °C	24 h	7
4	L3	N1	K_2CO_3	toluene	130 °C	24 h	trace
5	L4	N1	K_2CO_3	toluene	130 °C	24 h	4
[c]6	L5	N1	K_2CO_3	toluene	130 °C	24 h	11
[c]7	L6	N1	K_2CO_3	toluene	130 °C	24 h	4
[c]8	L7	N1	K_2CO_3	toluene	130 °C	24 h	2
[c]9	L8	N1	K_2CO_3	toluene	130 °C	24 h	2
10	L1	N1	Na_2CO_3	toluene	130 °C	24 h	13
11	L1	N1	Cs_2CO_3	toluene	130 °C	24 h	8
12	L1	N1	^{t}BuONa	toluene	130 °C	24 h	n.d.
13	L1	N1	KOAc	toluene	130 °C	24 h	trace
14	L1	N1	CsOAc	toluene	130 °C	24 h	5
15	L1	N1	K_3PO_4	toluene	130 °C	24 h	36
16	L1	N2	K_3PO_4	toluene	130 °C	24 h	21
17	L1	N3	K_3PO_4	toluene	130 °C	24 h	35
18	L1	N4	K_3PO_4	toluene	130 °C	24 h	trace
19	L1	N5	K_3PO_4	toluene	130 °C	24 h	n.d.
20	L1	-	K_3PO_4	toluene	130 °C	24 h	n.d.
21	L1 (20 mol%)	N1	K_3PO_4	toluene	130 °C	24 h	30
22	L1 (15 mol%)	N1	K_3PO_4	toluene	130 °C	24 h	29
23	L1	N1	K_3PO_4	xylene	130 °C	24 h	34
24	L1	N1	K_3PO_4	DMF	130 °C	24 h	n.d.
25	L1	N1	K_3PO_4 (2.5 eq.)	toluene	130 °C	24 h	16
26	L1	N1	K_3PO_4 (3.5 eq.)	toluene	130 °C	24 h	50
27	L1	N1	K_3PO_4 (4.0 eq.)	toluene	130 °C	24 h	40
28	L1	N1	K_2CO_3 (3.5 eq.)	toluene	130 °C	24 h	38
29	L1	N1	K_2CO_3 (4.0 eq.)	toluene	130 °C	24 h	42
30	L1	N1	K_2CO_3 (4.5 eq.)	toluene	130 °C	24 h	51
31	L1	N1	K_2CO_3 (5.0 eq.)	toluene	130 °C	24 h	29
32	L1	N1	K_2CO_3 (4.5 eq.)	toluene	90 °C	24 h	5

**33**	**L1**	**N1**	K_2CO_3 (4.5 eq.)	toluene	110 °C	24 h	13
**34**	**L1**	**N1**	K_2CO_3 (4.5 eq.)	toluene	120 °C	24 h	26
**35**	**L1**	**N1**	K_2CO_3 (4.5 eq.)	toluene	140 °C	24 h	40
**36**	**L1**	**N1**	K_2CO_3 (4.5 eq.)	dioxane	130 °C	24 h	7
**37**	**L1**	**N1**	K_2CO_3 (4.5 eq.)	*t*-Amyl OH	130 °C	24 h	4
**38**	**L1**	**N1**	K_2CO_3 (4.5 eq.)	toluene	130 °C	48 h	63
**39**	**L1**	**N1**	K_2CO_3 (4.5 eq.)	toluene	130 °C	72 h	71 [b]

[a]Yield was determined by ^{1}H NMR with CH_2Br_2 (0.1mmol) as internal standard. [b]Isolated yield. [c]12 mol% ligand was used. n.d. = not detected.

NBE mediators and ligands:

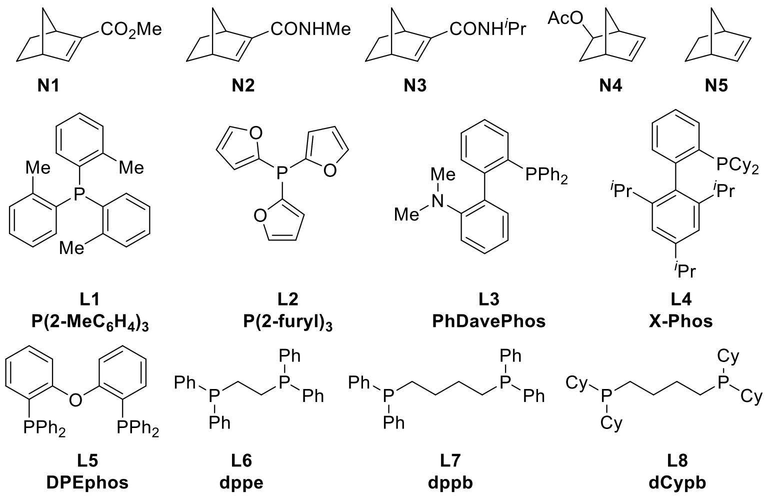

3.2 General procedures for synthesis of products

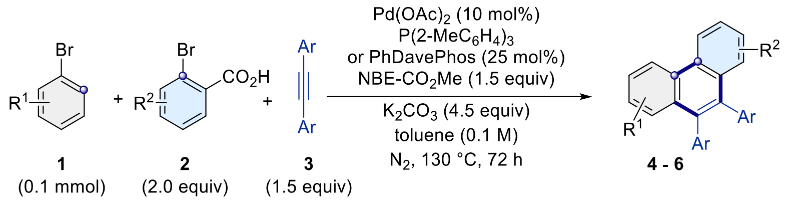

General procedure A:

The oven-dried Schlenk tube (10 mL) equipped with a stirring bar was charged with substrates **1** (0.1 mmol, 1.0 equiv), **2** (0.2 mmol, 2.0 equiv), **3** (0.15 mmol, 1.5 equiv), $Pd(OAc)_2$ (10 mol%), $P(2\text{-}MeC_6H_4)_3$ (25 mol%) and K_2CO_3 (4.5 equiv) in the air. Followed by the mediator NBE-CO_2Me (1.5 equiv) and the anhydrous toluene (1.0 mL) were added via syringe under nitrogen flow. Then the tube was evacuated and back-filled with N_2 for three times. Finally, the reaction mixture was stirred at 130 ℃ for 72 h and then cooled to room temperature. Upon completion of the

reaction, the mixture was diluted with 5 mL EtOAc and filtered through a short pad of Celite and the Celite pad were washed with an additional EtOAc (10 mL × 5). The filtrate was concentrated *in vacuo* to give the crude product. The crude ^{1}H NMR spectrum was taken using CH_2Br_2 (0.1 mmol) as internal standard. Finally, the crude product was purified via silica gel column chromatography using PE/EtOAc as the eluent.

General procedure B:

The oven-dried Schlenk tube (10 mL) equipped with a stirring bar was charged with substrates **1** (0.1 mmol, 1.0 equiv), **2** (0.2 mmol, 2.0 equiv), **3** (0.15 mmol, 1.5 equiv), $Pd(OAc)_2$ (10 mol%), PhDavePhos (25 mol%) and K_2CO_3 (4.5 equiv) in the air. Followed by the mediator NBE-CO_2Me (1.5 equiv) and the anhydrous toluene (1.0 mL) were added via syringe under nitrogen flow. Then the tube was evacuated and back-filled with N_2 for three times. Finally, the reaction mixture was stirred at 130 ℃ for 72 h and then cooled to room temperature. Upon completion of the reaction, the mixture was diluted with 5 mL EtOAc and filtered through a short pad of Celite and the Celite pad were washed with an additional EtOAc (10 mL × 5). The filtrate was concentrated *in vacuo* to give the crude product. The crude ^{1}H NMR spectrum was taken using CH_2Br_2 (0.1 mmol) as internal standard. Finally, the crude product was purified via silica gel column chromatography using PE/EtOAc as the eluent.

3.3 Characterization of products

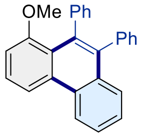

1-methoxy-9,10-diphenylphenanthrene (4a)

The general procedure A was followed. Yield: 71%, 25.6 mg. ^{1}H NMR (400 MHz, Chloroform-*d*) δ 8.76 (d, *J* = 8.4 Hz, 1H), 8.45 (d, *J* = 8.4 Hz, 1H), 7.66 – 7.55 (m, 2H), 7.48 – 7.39 (m, 2H), 7.26 – 7.11 (m, 3H), 7.12 – 6.99 (m, 7H), 6.96 (d, *J* = 8.0 Hz, 1H), 3.33 (s, 3H). ^{13}C NMR (101 MHz, Chloroform-*d*) δ 157.9, 144.2, 139.9,

138.2, 135.2, 132.3, 132.1, 131.3, 129.7, 129.6, 127.9, 127.4, 127.0, 126.8, 126.4, 126.3, 126.2, 124.8, 123.1, 122.6, 115.6, 109.3, 56.0. HRMS (m/z, ESI): calcd for $C_{27}H_{21}O^+$ $(M+H)^+$ 361.1587, found 361.1551.

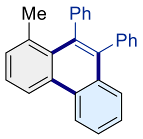

1-methyl-9,10-diphenylphenanthrene (4b)

The general procedure A was followed. Yield: 75%, 26.0 mg. ^{1}H NMR (400 MHz, Chloroform-*d*) δ 8.79 (d, *J* = 8.8 Hz, 1H), 8.75 (d, *J* = 8.8 Hz, 1H), 7.61 (t, *J* = 6.8 Hz, 1H), 7.54 (t, *J* = 7.6 Hz, 1H), 7.46 – 7.37 (m, 2H), 7.33 (d, *J* = 6.8 Hz, 1H), 7.22 – 7.13 (m, 3H), 7.12 – 6.99 (m, 7H), 1.91 (s, 3H). ^{13}C NMR (101 MHz, Chloroform-*d*) δ 143.2, 140.1, 139.0, 137.0, 136.96, 131.6, 131.59, 131.5, 131.3, 130.6, 130.5, 127.8, 127.5, 127.1, 126.6, 126.5, 126.3, 126.2, 123.0, 121.4, 25.5. HRMS (m/z, ESI): calcd for $C_{27}H_{21}^+$ $(M+H)^+$ 345.1565, found 345.1574.

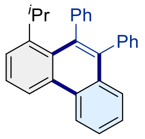

1-isopropyl-9,10-diphenylphenanthrene (4c)

The general procedure A was followed. Yield: 79%, 29.4 mg. ^{1}H NMR (400 MHz, Chloroform-*d*) δ 8.77 (d, *J* = 8.4 Hz, 1H), 8.71 (d, *J* = 7.6 Hz, 1H), 7.69 – 7.56 (m, 3H), 7.45 – 7.36 (m, 2H), 7.24 – 7.12 (m, 3H), 7.12 – 6.98 (m, 7H), 2.88 (p, *J* = 6.8 Hz, 1H), 0.89 (d, *J* = 6.8 Hz, 6H). ^{13}C NMR (101 MHz, Chloroform-*d*) δ 148.7, 143.6, 140.3, 138.9, 136.1, 131.7, 131.5, 131.3, 130.7, 130.6, 129.8, 127.8, 127.5, 127.3, 126.6, 126.5, 126.4, 126.3, 126.2, 126.0, 123.0, 120.7, 29.4, 24.8. HRMS (m/z, ESI): calcd for $C_{29}H_{25}^+$ $(M+H)^+$ 373.1878, found 373.1888.

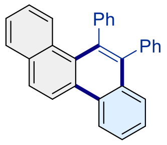

5,6-diphenylchrysene (4d)

The general procedure A was followed. Yield: 95%, 36.2 mg. ^{1}H NMR (400 MHz, Chloroform-*d*) δ 8.86 (d, *J* = 8.4 Hz, 1H), 8.82 (d, *J* = 9.2 Hz, 1H), 8.01 (d, *J* = 8.8 Hz,

1H), 7.89 (d, *J* = 7.6 Hz, 1H), 7.68 (t, *J* = 6.8 Hz, 1H), 7.61 – 7.52 (m, 2H), 7.48 (t, *J* = 7.2 Hz, 1H), 7.39 (t, *J* = 7.2 Hz, 1H), 7.28 – 7.19 (m, 3H), 7.19 – 7.12 (m, 3H), 7.12 – 7.05 (m, 4H), 7.01 (t, *J* = 7.6 Hz, 1H). ^{13}C NMR (101 MHz, Chloroform-*d*) δ 143.7, 140.2, 139.5, 136.9, 133.9, 131.9, 131.4, 131.2, 130.2, 129.7, 128.8, 128.4, 128.2, 127.92, 127.9, 127.6, 126.7, 126.6, 126.4, 126.35, 125.7, 124.8, 123.3, 121.2. HRMS (m/z, ESI): calcd for $C_{30}H_{21}^+$ $(M+H)^+$ 381.1565, found 381.1578.

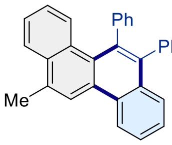

12-methyl-5,6-diphenylchrysene (4e)

The general procedure A was followed. Yield: 99%, 39.1 mg. ^{1}H NMR (400 MHz, Chloroform-*d*) δ 8.85 (d, *J* = 8.4 Hz, 1H), 8.66 (s, 1H), 8.06 (d, *J* = 8.4 Hz, 1H), 7.73 – 7.62 (m, 2H), 7.55 (d, *J* = 8.0 Hz, 1H), 7.45 (dt, *J* = 12.5, 7.4 Hz, 2H), 7.22 (m, 3H), 7.16 – 7.11 (m, 3H), 7.10 – 6.97 (m, 5H), 2.89 (s, 3H). ^{13}C NMR (101 MHz, Chloroform-*d*) δ 143.7, 140.2, 138.6, 136.6, 133.7, 133.3, 131.9, 131.4, 131.37, 131.2, 129.7, 129.4, 129.3, 128.1, 127.8, 127.6, 127.0, 126.5, 126.4, 126.3, 126.2, 125.6, 124.3, 124.1, 123.2, 121.7, 20.8. HRMS (m/z, ESI): calcd for $C_{31}H_{23}^+$ $(M+H)^+$ 395.1722, found 395.1717.

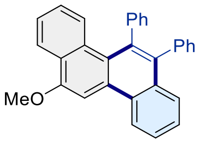

12-methoxy-5,6-diphenylchrysene (4f)

The general procedure A was followed. Yield: 93%, 38.1 mg. ^{1}H NMR (400 MHz, Chloroform-*d*) δ 8.75 (d, *J* = 8.4 Hz, 1H), 8.39 (d, *J* = 8.0 Hz, 1H), 8.04 (s, 1H), 7.64 (t, *J* = 7.6 Hz, 1H), 7.56 (t, *J* = 9.6 Hz, 2H), 7.46 (t, *J* = 7.6 Hz, 1H), 7.40 (t, *J* = 7.6 Hz, 1H), 7.28 – 7.17 (m, 3H), 7.17 – 6.96 (m, 8H), 4.24 (s, 3H). ^{13}C NMR (101 MHz, Chloroform-*d*) δ 154.5, 143.7, 140.3, 137.1, 136.7, 132.4, 132.1, 131.6, 131.2, 130.5, 129.6, 128.8, 128.1, 127.9, 127.5, 127.3, 126.6, 126.3, 126.2, 126.1, 125.4, 125.3, 123.2, 123.18, 121.9, 97.9, 55.7. HRMS (m/z, ESI): calcd for $C_{31}H_{23}O^+$ $(M+H)^+$ 411.1671, found 411.1692.

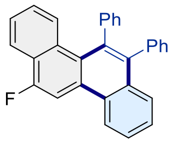

12-fluoro-5,6-diphenylchrysene (4g)

The general procedure A was followed. Yield: 91%, 36.5 mg. ^{1}H NMR (400 MHz, Chloroform-*d*) δ 8.68 (d, J = 8.4 Hz, 1H), 8.45 (d, J = 12.8 Hz, 1H), 8.21 (d, J = 8.0 Hz, 1H), 7.67 (t, J = 7.6 Hz, 1H), 7.60 – 7.52 (m, 2H), 7.52 – 7.43 (m, 2H), 7.26 – 7.19 (m, 3H), 7.18 – 7.12 (m, 3H), 7.10 – 7.02 (m, 5H). ^{13}C NMR (101 MHz, Chloroform-*d*) δ 158.1 (d, $J_{C\text{-}F}$ = 250.8 Hz), 143.3, 139.9, 138.8 (d, $J_{C\text{-}F}$ = 2.1 Hz), 136.6, 132.9 (d, $J_{C\text{-}F}$ = 4.9 Hz), 131.9, 131.3, 131.1, 130.0 (d, $J_{C\text{-}F}$ = 8.8 Hz), 129.6 (d, $J_{C\text{-}F}$ = 4.4 Hz), 128.8 (d, $J_{C\text{-}F}$ = 2.7 Hz), 128.3, 127.9, 127.6, 127.0, 126.6, 126.5, 126.4, 126.0 (d, $J_{C\text{-}F}$ = 1.6 Hz), 125.9, 125.1 (d, $J_{C\text{-}F}$ = 2.3 Hz), 124.9 (d, $J_{C\text{-}F}$ = 16.8 Hz), 123.3, 120.6 (d, $J_{C\text{-}F}$ = 6.9 Hz), 103.9 (d, $J_{C\text{-}F}$ = 21.8 Hz). HRMS (m/z, ESI): calcd for $C_{30}H_{20}F^+$ $(M+H)^+$ 399.1471, found 399.1473.

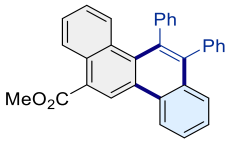

methyl 11,12-diphenylchrysene-6-carboxylate (4h)

The general procedure A was followed. Yield: 83%, 36.2 mg. ^{1}H NMR (400 MHz, Chloroform-*d*) δ 9.48 (s, 1H), 8.86 (t, J = 10.0 Hz, 2H), 7.79 – 7.67 (m, 2H), 7.59 (d, J = 8.4 Hz, 1H), 7.54 – 7.42 (m, 2H), 7.28 – 7.18 (m, 3H), 7.17 – 7.05 (m, 5H), 7.05 – 6.95 (m, 3H), 4.12 (s, 3H). ^{13}C NMR (101 MHz, Chloroform-*d*) δ 168.5, 143.1, 141.9, 139.7, 136.4, 132.0, 131.8, 131.3, 131.1, 130.8, 130.75, 130.3, 129.3, 128.2, 128.0, 127.9, 127.7, 127.2, 127.1, 126.8, 126.7, 126.6, 126.4, 126.1, 125.8, 124.8, 123.2, 52.6. HRMS (m/z, ESI): calcd for $C_{32}H_{23}O_2^+$ $(M+H)^+$ 439.1693, found 439.1661.

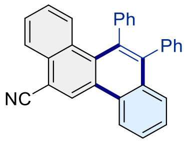

11,12-diphenylchrysene-6-carbonitrile (4i)

The general procedure A was followed. Yield: 61%, 25.0 mg. ^{1}H NMR (400 MHz, Chloroform-*d*) δ 9.30 (s, 1H), 8.79 (d, J = 8.4 Hz, 1H), 8.33 (d, J = 8.4 Hz, 1H), 7.77

(t, *J* = 6.8 Hz, 1H), 7.68 (d, *J* = 8.8 Hz, 1H), 7.64 – 7.51 (m, 3H), 7.32 – 7.21 (m, 3H), 7.21 – 7.14 (m, 3H), 7.15 – 7.11 (m, 1H), 7.11 – 7.06 (m, 2H), 7.06 – 6.99 (m, 2H). ^{13}C NMR (101 MHz, Chloroform-*d*) δ 143.0, 142.6, 139.3, 136.5, 132.0, 131.1, 131.0, 130.9, 130.7, 129.6, 129.5, 129.3, 128.4, 128.2, 127.9, 127.8, 127.76, 127.6, 127.4, 126.8, 126.79, 126.2, 125.6, 122.9, 118.7, 110.0. HRMS (m/z, ESI): calcd for $C_{31}H_{20}N^+$ $(M+H)^+$ 406.1517, found 406.1558.

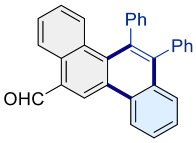

11,12-diphenylchrysene-6-carbaldehyde (4j)

The general procedure A was followed. Yield: 54%, 22.2 mg. ^{1}H NMR (400 MHz, Chloroform-*d*) δ 10.58 (s, 1H), 9.32 (d, *J* = 8.4 Hz, 1H), 9.25 (s, 1H), 8.89 (d, *J* = 8.4 Hz, 1H), 7.77 (t, *J* = 7.6 Hz, 1H), 7.72 (d, *J* = 8.8 Hz, 1H), 7.61 (d, *J* = 8.4 Hz, 1H), 7.54 (q, *J* = 7.6 Hz, 2H), 7.31 – 7.21 (m, 3H), 7.19 – 7.11 (m, 3H), 7.11 – 7.04 (m, 3H), 7.04 – 6.96 (m, 2H). ^{13}C NMR (101 MHz, Chloroform-*d*) δ 193.6, 143.3, 142.8, 139.4, 136.5, 134.3, 132.0, 131.9, 131.8, 131.1, 130.9, 130.3, 130.2, 130.0, 129.2, 128.3, 128.2, 128.0, 127.7, 127.64, 127.6, 127.3, 126.8, 126.6, 125.4, 124.8, 122.9. HRMS (m/z, ESI): calcd for $C_{31}H_{21}O^+$ $(M+H)^+$ 409.1514, found 409.1559.

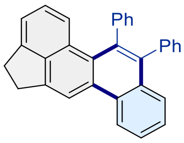

11,12-diphenyl-4,5-dihydrocyclopenta[*hi*]chrysene (4k)

The general procedure A was followed. Yield: 86%, 35.0 mg. ^{1}H NMR (400 MHz, Chloroform-*d*) δ 8.89 (d, *J* = 8.4 Hz, 1H), 8.70 (s, 1H), 7.65 (t, *J* = 7.6 Hz, 1H), 7.53 (d, *J* = 8.4 Hz, 1H), 7.47 (t, *J* = 7.6 Hz, 1H), 7.27 (d, *J* = 6.8 Hz, 1H), 7.26 – 7.15 (m, 6H), 7.15 – 7.07 (m, 4H), 7.03 (t, *J* = 8.0 Hz, 1H), 6.80 (d, *J* = 8.8 Hz, 1H), 3.54 (dd, *J* = 9.6, 5.6 Hz, 2H), 3.45 (dd, *J* = 8.8, 4.4 Hz, 2H). ^{13}C NMR (101 MHz, Chloroform-*d*) δ 145.6, 145.5, 143.4, 140.2, 139.2, 137.9, 137.5, 132.0, 131.7, 131.5, 131.1, 130.4, 130.0, 128.2, 127.8, 127.5, 127.0, 126.6, 126.4, 126.3, 126.2, 125.5, 124.5, 123.6, 120.4, 114.5, 30.6, 29.9. HRMS (m/z, ESI): calcd for $C_{32}H_{23}^+$ $(M+H)^+$

407.1722, found 407.1722.

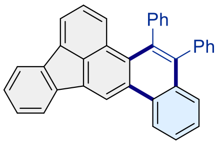

4,5-diphenylindeno[1,2,3-*hi*]chrysene (4l)

The general procedure A was followed. Yield: 98%, 45.1 mg. ^{1}H NMR (400 MHz, Chloroform-*d*) δ 9.36 (s, 1H), 9.02 (d, *J* = 8.4 Hz, 1H), 8.11 (d, *J* = 6.4 Hz, 1H), 7.87 (d, *J* = 6.8 Hz, 1H), 7.79 (d, *J* = 6.8 Hz, 1H), 7.73 (t, *J* = 7.2 Hz, 1H), 7.63 – 7.48 (m, 2H), 7.47 – 7.36 (m, 2H), 7.32 – 7.02 (m, 11H), 6.92 (d, *J* = 8.4 Hz, 1H). ^{13}C NMR (101 MHz, Chloroform-*d*) δ 143.0, 140.7, 139.9, 139.8, 138.5, 137.7, 136.9, 135.8, 133.0, 132.0, 131.23, 131.2, 131.19, 131.1, 129.0, 128.5, 128.4, 128.2, 128.0, 127.8, 127.6, 127.5, 127.1, 126.9, 126.74, 126.7, 126.5, 123.5, 121.7, 121.3, 119.4, 116.0. HRMS (m/z, ESI): calcd for $C_{36}H_{23}^{+}$ $(M+H)^{+}$ 455.1722, found 455.1714.

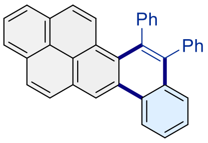

11,12-diphenylbenzo[*pqr*]picene (4m)

The general procedure A was followed. Yield: 75%, 34.1 mg. ^{1}H NMR (400 MHz, Chloroform-*d*) δ 9.48 (s, 1H), 9.10 (d, *J* = 8.4 Hz, 1H), 8.16 (d, *J* = 9.2 Hz, 1H), 8.08 (d, *J* = 7.6 Hz, 1H), 8.01 (t, *J* = 6.4 Hz, 2H), 7.90 (dd, *J* = 14.4, 7.2 Hz, 2H), 7.75 (t, *J* = 7.6 Hz, 1H), 7.65 – 7.51 (m, 3H), 7.30 – 7.21 (m, 3H), 7.19 – 7.09 (m, 5H), 7.09 – 7.02 (m, 2H). ^{13}C NMR (101 MHz, Chloroform-*d*) δ 143.9, 140.2, 139.1, 136.7, 131.8, 131.6, 131.4, 131.3, 130.5, 130.2, 130.1, 129.2, 128.7, 128.3, 128.2, 128.1, 128.0, 127.64, 127.6, 127.0, 126.8, 126.4, 126.3, 126.2, 126.18, 125.2, 125.1, 125.0, 124.9, 124.8, 123.5, 119.9. HRMS (m/z, ESI): calcd for $C_{36}H_{23}^{+}$ $(M+H)^{+}$ 455.1722, found 455.1718.

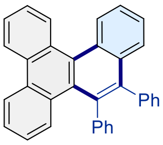

5,6-diphenylbenzo[*g*]chrysene (4n)

The general procedure A was followed. Yield: 61%, 26.1 mg. ^{1}H NMR (400 MHz,

Chloroform-*d*) δ 8.80 (t, *J* = 8.8 Hz, 2H), 8.65 (d, *J* = 7.6 Hz, 1H), 8.55 (d, *J* = 8.4 Hz, 1H), 7.77 (d, *J* = 8.4 Hz, 1H), 7.66 (p, *J* = 6.4 Hz, 2H), 7.62 – 7.50 (m, 2H), 7.46 (t, *J* = 7.6 Hz, 1H), 7.42 (t, *J* = 7.2 Hz, 1H), 7.37 – 7.21 (m, 3H), 7.17 (d, *J* = 7.2 Hz, 2H), 7.12 – 7.01 (m, 6H). ^{13}C NMR (101 MHz, Chloroform-*d*) δ 142.8, 139.5, 138.4, 135.9, 132.4, 132.0, 131.7, 131.5, 131.2, 130.8, 130.7, 129.94, 129.9, 129.6, 129.4, 128.9, 128.6, 128.5, 128.4, 127.9, 127.7, 127.0, 126.9, 126.6, 126.3, 126.2, 125.7, 125.6, 123.7, 123.2. HRMS (m/z, ESI): calcd for $C_{34}H_{23}^+$ (M+H)$^+$ 431.1722, found 431.1724.

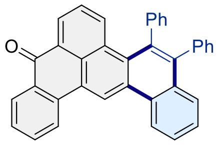

5,6-diphenyl-10H-dibenzo[*gh,m*]tetraphen-10-one (4o)

The general procedure A was followed. Yield: 84%, 40.4 mg. ^{1}H NMR (400 MHz, Chloroform-*d*) δ 9.75 (s, 1H), 8.95 (d, *J* = 8.4 Hz, 1H), 8.60 (dd, *J* = 7.2, 4.8 Hz, 2H), 8.52 (d, *J* = 7.6 Hz, 1H), 8.03 (d, *J* = 8.8 Hz, 1H), 7.82 (t, *J* = 7.2 Hz, 1H), 7.77 (t, *J* = 7.6 Hz, 1H), 7.66 – 7.51 (m, 3H), 7.33 – 7.19 (m, 4H), 7.19 – 7.14 (m, 3H), 7.10 (d, *J* = 7.6 Hz, 2H), 7.07 – 6.99 (m, 2H). ^{13}C NMR (101 MHz, Chloroform-*d*) δ 184.2, 143.0, 141.5, 139.6, 136.5, 136.4, 135.2, 133.5, 132.2, 131.2, 131.16, 131.1, 131.0, 130.2, 129.4, 129.3, 129.0, 128.6, 128.54, 128.5, 128.4, 128.3, 128.2, 127.8, 127.3, 127.28, 126.72, 126.7, 125.3, 125.1, 123.3, 122.9, 120.5. HRMS (m/z, ESI): calcd for $C_{37}H_{23}O^+$ (M+H)$^+$ 483.1743, found 483.1706.

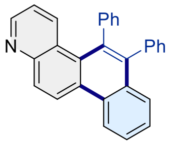

5,6-diphenylnaphtho[2,1-*f*]quinolone (4p)

The general procedure A was followed. Yield: 44%, 17.0 mg. ^{1}H NMR (400 MHz, Chloroform-*d*) δ 9.08 (d, *J* = 9.2 Hz, 1H), 8.89 (d, *J* = 8.8 Hz, 1H), 8.77 (s, 1H), 8.28 (d, *J* = 9.6 Hz, 1H), 7.79 (d, *J* = 8.8 Hz, 1H), 7.73 (t, *J* = 7.2 Hz, 1H), 7.58 (d, *J* = 8.4 Hz, 1H), 7.56 – 7.49 (m, 1H), 7.30 – 7.16 (m, 6H), 7.15 – 7.03 (m, 4H), 6.92 (dd, *J* = 9.2, 4.0 Hz, 1H). ^{13}C NMR (101 MHz, Chloroform-*d*) δ 149.2, 149.0, 142.9, 140.2,

139.7, 136.6, 136.1, 132.1, 131.2, 131.0, 130.0, 129.5, 129.4, 128.6, 128.0, 127.7, 127.6, 127.1, 127.0, 126.8, 126.6, 125.1, 123.3, 119.3. HRMS (m/z, ESI): calcd for $C_{29}H_{20}N^+$ $(M+H)^+$ 382.1590, found 382.1567.

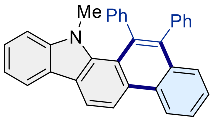

13-methyl-1,2-diphenyl-13*H*-naphtho[2,1-*a*]carbazole (4q)

The general procedure A was followed. Yield: 55%, 23.8 mg. ^{1}H NMR (400 MHz, Chloroform-*d*) δ 8.84 (d, *J* = 8.4 Hz, 1H), 8.66 (d, *J* = 8.8 Hz, 1H), 8.37 (d, *J* = 8.4 Hz, 1H), 8.18 (d, *J* = 8.0 Hz, 1H), 7.73 – 7.61 (m, 2H), 7.44 (dt, *J* = 16.8, 7.2 Hz, 2H), 7.38 – 7.27 (m, 4H), 7.21 – 7.05 (m, 4H), 7.05 – 6.91 (m, 4H), 2.89 (s, 3H). ^{13}C NMR (101 MHz, Chloroform-*d*) δ 143.5, 141.4, 140.4, 139.6, 137.5, 133.9, 132.8, 131.7, 131.6, 131.4, 130.8, 127.92, 127.87, 127.0, 126.7, 126.6, 126.3, 126.2, 125.6, 123.7, 123.6, 123.0, 120.0, 119.8, 119.6, 118.3, 115.6, 110.3, 36.6. HRMS (m/z, ESI): calcd for $C_{33}H_{24}N^+$ $(M+H)^+$ 434.1830, found 434.1866.

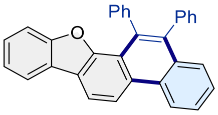

1,2-diphenylphenanthro[1,2-*b*]benzofuran (4r)

The general procedure B was followed. Yield: 50%, 21.1 mg. ^{1}H NMR (400 MHz, Chloroform-*d*) δ 8.89 (d, *J* = 8.4 Hz, 1H), 8.83 (d, *J* = 8.8 Hz, 1H), 8.22 (d, *J* = 8.8 Hz, 1H), 8.03 – 7.95 (m, 1H), 7.70 (t, *J* = 7.2 Hz, 1H), 7.63 (q, *J* = 8.4 Hz, 1H), 7.57 (d, *J* = 8.4 Hz, 1H), 7.50 (t, *J* = 7.2 Hz, 1H), 7.35 – 7.14 (m, 11H), 7.07 – 7.01 (m, 1H). ^{13}C NMR (101 MHz, Chloroform-*d*) δ 155.7, 153.3, 141.5, 139.4, 138.8, 134.2, 132.4, 132.3, 132.0, 131.3, 130.9, 130.2, 129.9, 128.3, 127.6, 127.1, 126.9, 126.8, 126.6, 126.5, 126.3, 124.0, 123.2, 122.7, 122.3, 120.2, 119.2, 119.1, 118.2, 111.7. HRMS (m/z, ESI): calcd for $C_{32}H_{21}O^+$ $(M+H)^+$ 421.1587, found 421.1635.

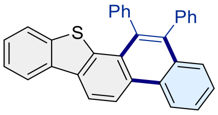

1,2-diphenylbenzo[*b*]phenanthro[2,1-*d*]thiophene (4s)

The general procedure A was followed. Yield: 67%, 29.5 mg. ^{1}H NMR (400 MHz, Chloroform-*d*) δ 8.95 (d, *J* = 8.8 Hz, 1H), 8.90 (d, *J* = 8.4 Hz, 1H), 8.42 (d, *J* = 8.8 Hz,

1H), 8.21 (d, J = 7.6 Hz, 1H), 7.77 – 7.61 (m, 2H), 7.57 – 7.47 (m, 2H), 7.42 (t, J = 7.6 Hz, 1H), 7.40 – 7.31 (m, 2H), 7.31 – 7.16 (m, 7H), 7.13 (d, J = 6.8 Hz, 2H). ^{13}C NMR (101 MHz, Chloroform-*d*) δ 140.9, 139.7, 139.3, 139.2, 138.6, 136.0, 135.2, 134.6, 132.8, 131.7, 131.1, 130.6, 129.6, 128.14, 128.1, 128.07, 127.6, 127.0, 126.9, 126.8, 126.6, 126.3, 124.1, 123.3, 122.1, 121.3, 120.4, 120.2. HRMS (m/z, ESI): calcd for $C_{32}H_{21}S^+$ $(M+H)^+$ 437.1286, found 437.1288.

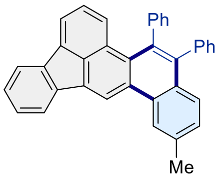

8-methyl-4,5-diphenylindeno[1,2,3-*hi*]chrysene (5a)

The general procedure A was followed. Yield: 97%, 45.5 mg. ^{1}H NMR (400 MHz, Chloroform-*d*) δ 9.33 (s, 1H), 8.77 (s, 1H), 8.13 (d, J = 6.8 Hz, 1H), 7.86 (d, J = 6.4 Hz, 1H), 7.77 (d, J = 6.4 Hz, 1H), 7.49 – 7.37 (m, 3H), 7.34 (d, J = 8.4 Hz, 1H), 7.28 – 7.04 (m, 11H), 6.92 (d, J = 8.8 Hz, 1H), 2.69 (s, 3H). ^{13}C NMR (101 MHz, Chloroform-*d*) δ 143.1, 140.7, 140.0, 139.8, 138.6, 136.9, 136.8, 136.4, 135.6, 133.0, 131.4, 131.24, 131.2, 130.8, 130.1, 129.0, 128.7, 128.6, 128.3, 128.1, 128.0, 127.8, 127.6, 127.4, 127.0, 126.8, 126.4, 123.0, 121.7, 121.3, 119.3, 116.1, 22.4. HRMS (m/z, ESI): calcd for $C_{37}H_{25}^+$ $(M+H)^+$ 469.1878, found 469.1871.

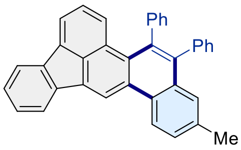

7-methyl-4,5-diphenylindeno[1,2,3-*hi*]chrysene (5b)

The general procedure A was followed. Yield: 94%, 44.2 mg. ^{1}H NMR (400 MHz, Chloroform-*d*) δ 9.35 (s, 1H), 8.91 (d, J = 8.8 Hz, 1H), 8.12 (d, J = 7.6 Hz, 1H), 7.88 (d, J = 6.4 Hz, 1H), 7.80 (d, J = 6.8 Hz, 1H), 7.56 (d, J = 8.8 Hz, 1H), 7.48 – 7.38 (m, 2H), 7.32 (s, 1H), 7.28 – 7.19 (m, 6H), 7.19 – 7.13 (m, 3H), 7.10 (d, J = 7.6 Hz, 2H), 6.91 (d, J = 8.4 Hz, 1H), 2.44 (s, 3H). ^{13}C NMR (101 MHz, Chloroform-*d*) δ 143.2, 140.8, 139.9, 139.6, 138.6, 137.8, 136.9, 136.5, 135.8, 132.9, 132.1, 131.3, 131.2, 129.3, 129.1, 128.8, 128.3, 128.1, 128.0, 127.8, 127.6, 127.5, 127.3, 127.1, 126.8, 126.4, 123.5, 121.7, 121.3, 119.2, 116.1, 21.9. HRMS (m/z, ESI): calcd for $C_{37}H_{25}^+$

(M+H)$^+$ 469.1878, found 469.1882.

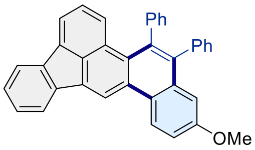

7-methoxy-4,5-diphenylindeno[1,2,3-*hi*]chrysene (5c)

The general procedure A was followed. Yield: 98%, 47.5 mg. ^{1}H NMR (400 MHz, Chloroform-*d*) δ 9.26 (s, 1H), 8.90 (d, *J* = 9.2 Hz, 1H), 8.09 (dd, *J* = 6.0, 2.4 Hz, 1H), 7.91 – 7.83 (m, 1H), 7.77 (d, *J* = 6.8 Hz, 1H), 7.46 – 7.39 (m, 2H), 7.36 (dd, *J* = 9.6, 2.8 Hz, 1H), 7.25 – 7.20 (m, 5H), 7.20 – 7.12 (m, 4H), 7.11 – 7.06 (m, 2H), 6.91 – 6.83 (m, 2H), 3.69 (s, 3H). ^{13}C NMR (101 MHz, Chloroform-*d*) δ 158.2, 143.1, 140.8, 139.9, 139.3, 138.6, 138.3, 136.9, 135.9, 133.5, 132.6, 131.4, 131.2, 131.1, 129.1, 128.3, 128.1, 127.7, 127.6, 127.4, 127.1, 127.06, 126.8, 126.5, 126.1, 125.3, 121.7, 121.2, 119.0, 117.7, 115.9, 107.7, 55.2. HRMS (m/z, ESI): calcd for $C_{37}H_{25}O^+$ (M+H)$^+$ 485.1827, found 485.1837.

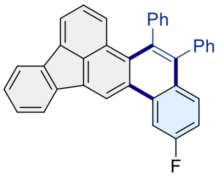

8-fluoro-4,5-diphenylindeno[1,2,3-*hi*]chrysene (5d)

The general procedure A was followed. Yield: 71%, 34.0 mg. ^{1}H NMR (400 MHz, Chloroform-*d*) δ 9.07 (s, 1H), 8.53 (d, *J* = 11.6 Hz, 1H), 8.06 (d, *J* = 6.0 Hz, 1H), 7.84 (d, *J* = 6.8 Hz, 1H), 7.76 (d, *J* = 6.8 Hz, 1H), 7.57 – 7.47 (m, 1H), 7.42 (q, *J* = 8.0, 4.0 Hz, 2H), 7.29 – 7.16 (m, 7H), 7.16 – 7.00 (m, 5H), 6.88 (d, *J* = 8.8 Hz, 1H). ^{13}C NMR (101 MHz, Chloroform-*d*) δ 161.6 (d, $J_{C\text{-}F}$ = 246.1 Hz), 142.8, 140.7, 139.6, 138.3, 136.9, 136.0, 133.2, 132.7 (d, $J_{C\text{-}F}$ = 8.3 Hz), 131.1, 130.6, 130.52, 130.5, 129.2, 128.9, 128.4, 128.3, 127.8, 127.7, 127.6, 127.2, 126.9, 126.6, 121.8, 121.3, 119.6, 116.0 (d, $J_{C\text{-}F}$ = 23.8 Hz), 115.8, 108.1 (d, $J_{C\text{-}F}$ = 22.5 Hz). HRMS (m/z, ESI): calcd for $C_{36}H_{22}F^+$ (M+H)$^+$ 473.1627, found 473.1615.

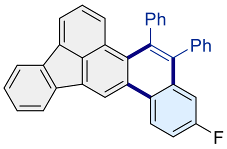

7-fluoro-4,5-diphenylindeno[1,2,3-*hi*]chrysene (5e)

The general procedure A was followed. Yield: 95%, 44.9 mg. ^{1}H NMR (400 MHz, Chloroform-*d*) δ 9.13 (s, 1H), 8.86 (dd, *J* = 9.2, 5.6 Hz, 1H), 8.07 – 7.97 (m, 1H), 7.82 (m, 1H), 7.73 (d, *J* = 6.8 Hz, 1H), 7.45 – 7.34 (m, 3H), 7.25 – 7.15 (m, 6H), 7.15 – 7.05 (m, 4H), 7.04 – 6.98 (m, 2H), 6.83 (d, *J* = 8.4 Hz, 1H). ^{13}C NMR (101 MHz, Chloroform-*d*) δ 161.3 (d, J_{C-F} = 246.3 Hz), 142.7, 140.7, 139.3, 139.2 (d, J_{C-F} = 4.3 Hz), 138.8, 138.4, 136.9, 136.2, 133.5 (d, J_{C-F} = 8.7 Hz), 132.8, 131.13, 131.1, 131.0, 128.9, 128.4, 128.3, 127.94, 127.9, 127.8, 127.7, 127.5, 127.2, 127.0, 126.7, 126.0 (d, J_{C-F} = 8.6 Hz), 121.7, 121.3, 119.4, 116.0 (d, J_{C-F} = 24.1 Hz), 115.7, 111.8 (d, J_{C-F} = 22.0 Hz). HRMS (m/z, ESI): calcd for $C_{36}H_{22}F^{+}$ $(M+H)^{+}$ 473.1627, found 473.1623.

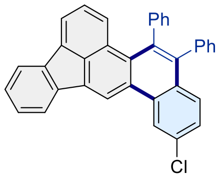

8-chloro-4,5-diphenylindeno[1,2,3-*hi*]chrysene (5f)

The general procedure A was followed. Yield: 96%, 47.1 mg. ^{1}H NMR (400 MHz, Chloroform-*d*) δ 9.06 (s, 1H), 8.87 (s, 1H), 8.06 (d, *J* = 6.4 Hz, 1H), 7.82 (d, *J* = 6.4 Hz, 1H), 7.72 (d, *J* = 6.8 Hz, 1H), 7.49 – 7.35 (m, 4H), 7.29 – 7.16 (m, 6H), 7.15 – 6.96 (m, 5H), 6.84 (d, *J* = 8.8 Hz, 1H). ^{13}C NMR (101 MHz, Chloroform-*d*) δ 142.7, 140.6, 139.44, 139.36, 138.3, 137.9, 136.9, 136.1, 133.1, 132.9, 132.2, 131.1, 131.0, 130.3, 130.2, 129.7, 129.1, 128.7, 128.4, 128.3, 127.8, 127.7, 127.6, 127.23, 127.2, 127.0, 126.7, 123.0, 121.9, 121.3, 119.6, 115.6. HRMS (m/z, ESI): calcd for $C_{36}H_{22}Cl^{+}$ $(M+H)^{+}$ 489.1332, found 489.1330.

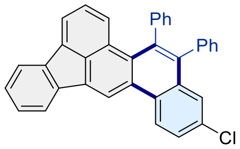

7-chloro-4,5-diphenylindeno[1,2,3-*hi*]chrysene (5g)

The general procedure A was followed. Yield: 98%, 48.0 mg. ^{1}H NMR (400 MHz, Chloroform-*d*) δ 9.29 (s, 1H), 8.95 (d, *J* = 9.2 Hz, 1H), 8.17 – 8.09 (m, 1H), 7.92 – 7.85 (m, 1H), 7.82 (d, *J* = 7.2 Hz, 1H), 7.67 (dd, *J* = 8.8, 2.4 Hz, 1H), 7.53 (s, 1H), 7.49 – 7.40 (m, 2H), 7.30 – 7.21 (m, 6H), 7.19 – 7.13 (m, 3H), 7.13 – 7.06 (m, 2H),

6.91 (d, *J* = 8.8 Hz, 1H). ^{13}C NMR (101 MHz, Chloroform-*d*) δ 148.2, 147.4, 142.7, 140.8, 139.1, 139.0, 138.4, 137.0, 136.4, 133.1, 132.8, 131.2, 131.1, 131.0, 129.6, 128.9, 128.6, 128.4, 127.9, 127.8, 127.6, 127.4, 127.3, 127.1, 126.9, 126.8, 125.3, 121.8, 121.4, 119.6, 115.7. HRMS (m/z, ESI): calcd for $C_{36}H_{22}Cl^+$ $(M+H)^+$ 489.1332, found 489.1319.

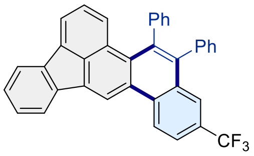

4,5-diphenyl-7-(trifluoromethyl)indeno[1,2,3-*hi*]chrysene (5h)

The general procedure A was followed (note: 20 mol% of $Pd(OAc)_2$ and 40 mol% of $P(2\text{-}MeC_6H_4)_3$ were used). Yield: 55%, 28.7 mg. ^{1}H NMR (400 MHz, Chloroform-*d*) δ 9.30 (s, 1H), 9.08 (d, *J* = 8.8 Hz, 1H), 8.18 – 8.08 (m, 1H), 7.94 – 7.86 (m, 2H), 7.84 (s, 1H), 7.81 (d, *J* = 7.2 Hz, 1H), 7.50 – 7.40 (m, 2H), 7.31 – 7.20 (m, 6H), 7.20 – 7.11 (m, 3H), 7.11 – 7.03 (m, 2H), 6.91 (d, *J* = 8.8 Hz, 1H). ^{13}C NMR (101 MHz, Chloroform-*d*) δ 142.5, 140.7, 140.1, 139.1, 138.7, 138.3, 137.0, 136.5, 133.3, 132.9, 131.3, 131.1, 131.0, 130.8, 129.8, 128.8, 128.54, 128.48, 128.36 (q, $J_{C\text{-}F}$ = 31.9 Hz), 127.93, 127.9, 127.7, 127.5, 127.2, 127.0, 125.4 (q, $J_{C\text{-}F}$ = 4.8 Hz), 124.5 (q, $J_{C\text{-}F}$ = 262.2 Hz), 124.6, 122.3 (q, $J_{C\text{-}F}$ = 4.8 Hz), 121.9, 121.4, 119.9, 115.8. HRMS (m/z, ESI): calcd for $C_{37}H_{22}F_3^+$ $(M+H)^+$ 523.1668, found 523.1793.

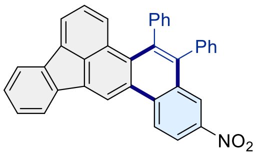

7-nitro-4,5-diphenylindeno[1,2,3-*hi*]chrysene (5i)

The general procedure A was followed. Yield: 50%, 24.9 mg. ^{1}H NMR (400 MHz, Chloroform-*d*) δ 9.89 (s, 1H), 9.26 (s, 1H), 8.23 (dd, *J* = 9.2, 2.0 Hz, 1H), 8.17 (d, *J* = 6.8 Hz, 1H), 7.85 (d, *J* = 6.4 Hz, 1H), 7.81 (d, *J* = 6.8 Hz, 1H), 7.67 (d, *J* = 9.2 Hz, 1H), 7.46 (p, *J* = 7.2 Hz, 2H), 7.32 – 7.23 (m, 6H), 7.20 – 7.11 (m, 3H), 7.11 – 7.05 (m, 2H), 6.88 (d, *J* = 8.8 Hz, 1H). ^{13}C NMR (101 MHz, Chloroform-*d*) δ 145.9, 142.2, 141.7, 140.7, 139.3, 138.7, 138.0, 137.4, 137.2, 135.1, 134.2, 133.3, 132.0, 131.1, 130.7, 130.5, 129.6, 128.8, 128.7, 128.5, 128.0, 127.94, 127.9, 127.7, 127.4, 127.1,

122.2, 121.4, 120.2, 120.1, 120.0, 115.3. HRMS (m/z, ESI): calcd for $C_{36}H_{22}NO_2^+$ $(M+H)^+$ 500.1572, found 500.1548.

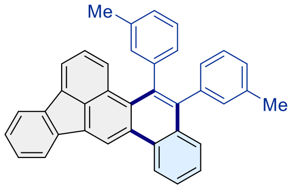

4,5-di-*m*-tolylindeno[1,2,3-*hi*]chrysene (6a)

The general procedure A was followed (note: the products were isolated as an inseparable mixture of diastereoisomers, *d.r.* = 1:1). Yield: 90%, 43.5 mg. ^{1}H NMR (400 MHz, Chloroform-*d*) δ 9.37 (s, 1H), 9.01 (d, *J* = 8.8 Hz, 1H), 8.16 – 8.07 (m, 1H), 7.91 – 7.84 (m, 1H), 7.81 (d, *J* = 6.8 Hz, 1H), 7.72 (t, *J* = 7.2 Hz, 1H), 7.60 (d, *J* = 8.0 Hz, 1H), 7.51 (t, *J* = 7.2 Hz, 1H), 7.46 – 7.36 (m, 2H), 7.21 – 7.08 (m, 3H), 7.07 – 6.84 (m, 7H), 2.26 (s, 1.50H), 2.25 (s, 1.44H), 2.23 (s, 1.47H), 2.21 (s, 1.53H). ^{13}C NMR (101 MHz, Chloroform-*d*) δ 142.8, 140.7, 140.1, 140.06, 139.7, 138.6, 137.8, 137.76, 137.7, 137.0, 136.9, 136.8, 135.7, 133.0, 132.1, 132.0, 131.9, 131.8, 131.2, 131.1, 129.1, 128.7, 128.6, 128.3, 128.24, 128.2, 128.16, 128.1, 128.05, 127.5, 127.4, 127.39, 127.3, 127.1, 127.08, 127.06, 126.6, 123.4, 121.7, 121.3, 119.4, 116.1, 21.5. HRMS (m/z, ESI): calcd for $C_{38}H_{27}^+$ $(M+H)^+$ 483.2035, found 483.2025.

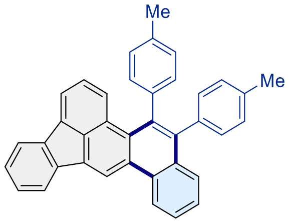

4,5-di-*p*-tolylindeno[1,2,3-*hi*]chrysene (6b)

The general procedure A was followed. Yield: 97%, 47.2 mg. ^{1}H NMR (400 MHz, Chloroform-*d*) δ 9.33 (s, 1H), 8.98 (d, *J* = 8.4 Hz, 1H), 8.08 (d, *J* = 6.4 Hz, 1H), 7.85 (d, *J* = 6.4 Hz, 1H), 7.78 (d, *J* = 6.8 Hz, 1H), 7.69 (t, *J* = 7.6 Hz, 1H), 7.55 (d, *J* = 8.4 Hz, 1H), 7.48 (t, *J* = 6.8 Hz, 1H), 7.44 – 7.35 (m, 2H), 7.16 (t, *J* = 7.6 Hz, 1H), 7.10 – 6.91 (m, 9H), 2.34 (s, 3H), 2.32 (s, 3H). ^{13}C NMR (101 MHz, Chloroform-*d*) δ 140.7, 140.1, 140.05, 138.6, 137.8, 136.9, 136.8, 136.2, 135.8, 135.7, 133.0, 132.3, 131.2, 131.17, 131.0, 130.9, 129.1, 128.9, 128.4, 128.1, 128.0, 127.4, 127.0, 126.6, 123.4,

121.7, 121.2, 119.3, 116.0, 21.5, 21.4. HRMS (m/z, ESI): calcd for $C_{38}H_{27}^{+}$ (M+H)$^{+}$ 483.2035, found 483.2026.

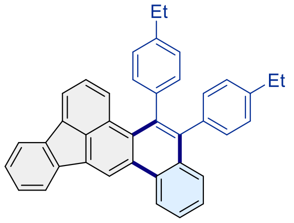

4,5-bis(4-ethylphenyl)indeno[1,2,3-*hi*]chrysene (6c)

The general procedure A was followed. Yield: 94%, 48.2 mg. ^{1}H NMR (400 MHz, Chloroform-*d*) δ 9.34 (s, 1H), 8.98 (d, *J* = 8.8 Hz, 1H), 8.13 – 8.05 (m, 1H), 7.85 (dd, *J* = 6.0, 2.0 Hz, 1H), 7.77 (d, *J* = 7.2 Hz, 1H), 7.69 (t, *J* = 7.2 Hz, 1H), 7.61 (d, *J* = 8.0 Hz, 1H), 7.49 (t, *J* = 7.2 Hz, 1H), 7.44 – 7.35 (m, 2H), 7.14 (dd, *J* = 8.4, 7.2 Hz, 1H), 7.08 – 6.94 (m, 9H), 2.61 (p, *J* = 7.4 Hz, 4H), 1.21 (t, *J* = 7.6 Hz, 6H). ^{13}C NMR (101 MHz, Chloroform-*d*) δ 142.8, 142.2, 140.7, 140.3, 140.2, 138.6, 137.9, 137.1, 136.8, 135.7, 133.0, 132.2, 131.2, 131.14, 131.1, 131.0, 129.1, 128.9, 128.14, 128.1, 128.0, 127.8, 127.4, 127.0, 126.6, 123.4, 121.7, 121.2, 119.3, 116.1, 28.8, 28.7, 16.1, 15.7. HRMS (m/z, ESI): calcd for $C_{40}H_{31}^{+}$ (M+H)$^{+}$ 511.2348, found 511.2340.

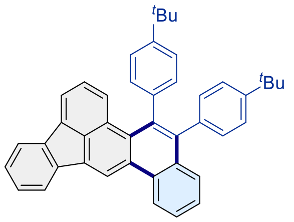

4,5-bis(4-(*tert*-butyl)phenyl)indeno[1,2,3-*hi*]chrysene (6d)

The general procedure A was followed. Yield: 89%, 50.8 mg. ^{1}H NMR (400 MHz, Chloroform-*d*) δ 9.38 (s, 1H), 9.02 (d, *J* = 8.4 Hz, 1H), 8.12 (d, *J* = 6.4 Hz, 1H), 7.87 (d, *J* = 6.8 Hz, 1H), 7.80 (d, *J* = 6.0 Hz, 1H), 7.75 – 7.67 (m, 2H), 7.51 (t, *J* = 7.6 Hz, 1H), 7.41 (p, *J* = 7.2 Hz, 2H), 7.21 – 7.08 (m, 6H), 7.01 (d, *J* = 7.6 Hz, 2H), 6.97 (d, *J* = 7.6 Hz, 2H), 1.29 (s, 9H), 1.28 (s, 9H). ^{13}C NMR (101 MHz, Chloroform-*d*) δ 149.6, 149.0, 140.8, 140.4, 140.1, 138.6, 138.1, 136.8, 136.7, 135.7, 133.0, 132.1, 131.2, 131.1, 130.9, 130.7, 129.1, 128.8, 128.2, 128.1, 128.07, 127.4, 127.0, 126.6, 124.9, 124.2, 123.4, 121.7, 121.2, 119.3, 116.1, 34.6, 34.5, 31.53, 31.49. HRMS (m/z, ESI): calcd for $C_{44}H_{39}^{+}$ (M+H)$^{+}$ 567.2974, found 567.2960.

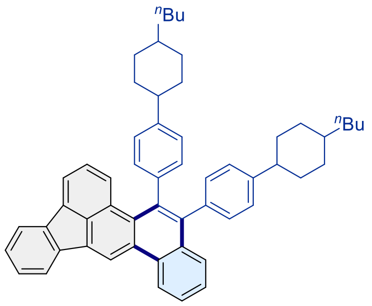

4,5-bis(4-(4-butylcyclohexyl)phenyl)indeno[1,2,3-*hi*]chrysene (6e)

The general procedure A was followed. Yield: 69%, 50.3 mg. ^{1}H NMR (400 MHz, Chloroform-*d*) δ 9.39 (s, 1H), 9.03 (d, *J* = 8.4 Hz, 1H), 8.13 (d, *J* = 6.4 Hz, 1H), 7.88 (d, *J* = 6.8 Hz, 1H), 7.80 (d, *J* = 6.8 Hz, 1H), 7.72 (t, *J* = 7.6 Hz, 1H), 7.66 (d, *J* = 8.4 Hz, 1H), 7.51 (t, *J* = 7.6 Hz, 1H), 7.46 – 7.37 (m, 2H), 7.14 (t, *J* = 8.0 Hz, 1H), 7.06 – 6.91 (m, 9H), 2.41 (q, *J* = 11.6 Hz, 2H), 1.86 (d, *J* = 11.2 Hz, 8H), 1.47 – 1.35 (m, 4H), 1.35 – 1.19 (m, 14H), 1.06 (t, *J* = 11.6 Hz, 4H), 0.92 (t, *J* = 6.8 Hz, 6H). ^{13}C NMR (101 MHz, Chloroform-*d*) δ 146.4, 145.8, 140.8, 140.4, 140.36, 138.6, 138.1, 137.1, 136.8, 135.7, 133.0, 132.2, 131.2, 131.1, 131.05, 130.9, 129.2, 128.9, 128.2, 128.1, 128.09, 127.4, 127.0, 126.6, 126.56, 125.8, 123.4, 121.7, 121.2, 119.3, 116.1, 44.5, 44.46, 37.5, 37.3, 34.7, 34.5, 33.8, 29.4, 23.2, 14.3. HRMS (m/z, ESI): calcd for $C_{56}H_{59}^{+}$ $(M+H)^{+}$ 731.4539, found 731.4511.

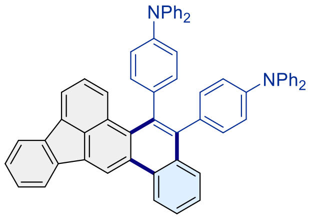

4,4'-(indeno[1,2,3-*hi*]chrysene-4,5-diyl)bis(*N*,*N*-diphenylaniline) (6f)

The general procedure A was followed. Yield: 73%, 57.5 mg. ^{1}H NMR (400 MHz, Chloroform-*d*) δ 9.39 (s, 1H), 9.04 (d, *J* = 8.8 Hz, 1H), 8.21 – 8.12 (m, 1H), 7.94 – 7.91 (m, 1H), 7.89 (d, *J* = 6.4 Hz, 1H), 7.82 (d, *J* = 8.0 Hz, 1H), 7.77 (t, *J* = 7.6 Hz, 1H), 7.60 (t, *J* = 7.6 Hz, 1H), 7.48 – 7.41 (m, 2H), 7.40 – 7.32 (m, 2H), 7.31 – 7.23 (m, 8H), 7.15 (t, *J* = 8.4 Hz, 8H), 7.07 – 6.99 (m, 12H). ^{13}C NMR (101 MHz, Chloroform-*d*) δ 148.04, 148.0, 146.5, 146.3, 140.8, 140.0, 138.6, 137.85, 137.8, 137.0, 135.9, 134.1, 133.1, 132.22, 132.2, 132.1, 131.3, 131.29, 129.5, 129.0, 128.8,

128.2, 128.1, 127.6, 126.9, 126.8, 126.77, 124.4, 124.3, 124.2, 123.6, 123.1, 122.9, 122.8, 121.7, 121.3, 119.4, 116.1. HRMS (m/z, ESI): calcd for $C_{60}H_{41}N_2^+$ $(M+H)^+$ 789.3191, found 789.3170.

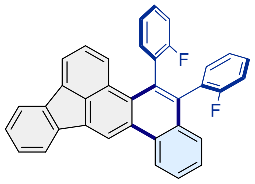

4,5-bis(2-fluorophenyl)indeno[1,2,3-*hi*]chrysene (6g)

The general procedure A was followed. Yield: 45%, 22.2 mg. ^{1}H NMR (400 MHz, Chloroform-*d*) δ 9.32 (s, 1H), 8.98 (d, *J* = 8.4 Hz, 1H), 8.05 (d, *J* = 6.4 Hz, 1H), 7.84 – 7.79 (m, 1H), 7.77 (d, *J* = 6.8 Hz, 1H), 7.73 – 7.65 (m, 1H), 7.53 – 7.41 (m, 2H), 7.40 – 7.31 (m, 2H), 7.31 – 7.13 (m, 5H), 7.06 – 6.96 (m, 3H), 6.95 – 6.86 (m, 2H). ^{13}C NMR (101 MHz, Chloroform-*d*) δ 159.9 (d, J_{C-F} = 245.5 Hz), 159.8 (d, J_{C-F} = 245.4 Hz), 140.7, 138.5, 137.2, 136.3, 134.9, 132.9, 132.2, 131.9, 131.6, 131.5, 131.0, 130.81, 129.8 (d, J_{C-F} = 34.7 Hz), 129.7 (d, J_{C-F} = 34.8 Hz), 128.5 (d, J_{C-F} = 56.6 Hz), 128.3, 127.9, 127.6, 127.4 (d, J_{C-F} = 17.9 Hz), 127.24, 127.22, 127.1, 126.2, 124.6 (d, J_{C-F} = 3.4 Hz), 123.9 (d, J_{C-F} = 3.7 Hz), 123.8, 121.8, 121.3, 119.6, 116.1, 115.6 (d, J_{C-F} = 22.0 Hz), 115.1 (d, J_{C-F} = 22.1 Hz). HRMS (m/z, ESI): calcd for $C_{36}H_{21}F_2^+$ $(M+H)^+$ 491.1533, found 491.1545.

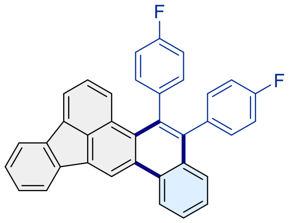

4,5-bis(4-fluorophenyl)indeno[1,2,3-*hi*]chrysene (6h)

The general procedure A was followed. Yield: 90%, 44.5 mg. ^{1}H NMR (400 MHz, Chloroform-*d*) δ 9.36 (s, 1H), 9.02 (d, *J* = 8.4 Hz, 1H), 8.12 (d, *J* = 6.0 Hz, 1H), 7.89 (d, *J* = 6.8 Hz, 1H), 7.83 (d, *J* = 7.2 Hz, 1H), 7.76 (t, *J* = 6.4 Hz, 1H), 7.59 – 7.50 (m, 2H), 7.48 – 7.39 (m, 2H), 7.22 (t, *J* = 7.6 Hz, 1H), 7.13 – 7.01 (m, 4H), 7.00 – 6.88 (m, 5H). ^{13}C NMR (101 MHz, Chloroform-*d*) δ 161.9 (d, J_{C-F} = 247.8 Hz), 161.6 (d, J_{C-F} = 246.9 Hz), 140.7, 139.2, 138.9 (d, J_{C-F} = 3.7 Hz), 138.5, 137.1, 137.0, 136.2, 135.6

(d, $J_{C\text{-}F}$ = 3.4 Hz), 133.0, 132.7, 132.64, 132.56, 132.0, 131.5, 131.3, 128.6 (d, $J_{C\text{-}F}$ = 39.9 Hz), 128.1 (d, $J_{C\text{-}F}$ = 54.3 Hz), 127.6, 127.5, 127.4, 127.0, 126.9, 123.6, 121.8, 121.4, 119.5, 116.0, 115.6 (d, $J_{C\text{-}F}$ = 21.1 Hz), 114.9 (d, $J_{C\text{-}F}$ = 21.4 Hz). HRMS (m/z, ESI): calcd for $C_{36}H_{21}F_2^+$ $(M+H)^+$ 491.1533, found 491.1520.

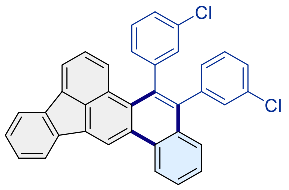

4,5-bis(3-chlorophenyl)indeno[1,2,3-*hi*]chrysene (6i)

The general procedure A was followed(note: the products were isolated as an inseparable mixture of diastereoisomers). Yield: 97%, 51.0 mg. ^{1}H NMR (400 MHz, Chloroform-*d*) δ 9.25 (s, 1H), 8.93 (d, *J* = 8.4 Hz, 1H), 8.09 – 7.97 (m, 1H), 7.87 – 7.78 (m, 1H), 7.75 (d, *J* = 6.8 Hz, 1H), 7.68 (t, *J* = 6.8 Hz, 1H), 7.51 – 7.42 (m, 2H), 7.40 – 7.31 (m, 2H), 7.19 – 7.01 (m, 7H), 7.00 – 6.84 (m, 3H). ^{13}C NMR (101 MHz, Chloroform-d) δ 144.5, 141.3, 140.7, 138.5, 138.4, 137.1, 136.3, 136.2, 134.5, 134.4, 133.9, 133.7, 133.0, 131.6, 131.4, 131.34, 131.26, 131.2, 131.1, 129.8, 129.7, 129.5, 129.4, 129.3, 129.25, 129.1, 129.0, 128.6, 128.4, 127.8, 127.6, 127.5, 127.4, 127.35, 127.2, 127.1, 127.0, 123.6, 121.8, 121.4, 119.6, 115.9. HRMS (m/z, ESI): calcd for $C_{36}H_{21}Cl_2^+$ $(M+H)^+$ 523.0942, found 523.0927.

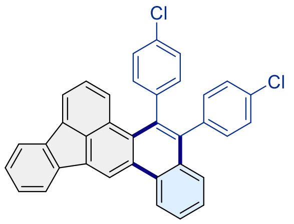

4,5-bis(4-chlorophenyl)indeno[1,2,3-*hi*]chrysene (6j)

The general procedure A was followed. Yield: 99%, 52.0 mg. ^{1}H NMR (400 MHz, Chloroform-*d*) δ 9.25 (s, 1H), 8.94 (d, *J* = 8.4 Hz, 1H), 8.05 (dd, *J* = 4.8, 2.0 Hz, 1H), 7.90 – 7.81 (m, 1H), 7.78 (d, *J* = 6.8 Hz, 1H), 7.72 (t, *J* = 7.2 Hz, 1H), 7.51 (t, *J* = 8.0 Hz, 1H), 7.48 – 7.36 (m, 3H), 7.29 – 7.13 (m, 5H), 7.05 – 6.92 (m, 4H), 6.89 (d, *J* = 8.8 Hz, 1H). ^{13}C NMR (101 MHz, Chloroform-*d*) δ 141.3, 140.6, 138.6, 138.4, 138.0, 137.1, 136.4, 136.2, 133.1, 132.9, 132.7, 132.4, 132.36, 131.6, 131.5, 131.3, 128.8,

128.6, 128.4, 128.1, 128.0, 127.7, 127.6, 127.4, 127.0, 126.9, 123.6, 121.8, 121.3, 119.5, 115.8. HRMS (m/z, ESI): calcd for $C_{36}H_{21}Cl_2^+$ $(M+H)^+$ 523.0942, found 523.0931.

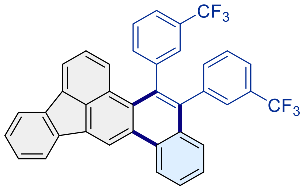

4,5-bis(3-(trifluoromethyl)phenyl)indeno[1,2,3-*hi*]chrysene (6k)

The general procedure A was followed (note: the products were isolated as an inseparable mixture of diastereoisomers, *d.r.* = 1:1). Yield: 91%, 53.9 mg. ^{1}H NMR (400 MHz, Chloroform-*d*) δ 9.21 (d, *J* = 3.2 Hz, 1H), 8.92 (d, *J* = 8.4 Hz, 1H), 8.05 – 7.95 (m, 1H), 7.84 – 7.75 (m, 1H), 7.75 – 7.65 (m, 2H), 7.48 (t, *J* = 8.0 Hz, 1H), 7.45 – 7.19 (m, 9H), 7.18 – 7.01 (m, 3H), 6.81 (d, *J* = 4.0 Hz, 0.52H), 6.78 (d, *J* = 4.4 Hz, 0.49H). ^{13}C NMR (101 MHz, Chloroform-d) δ 143.5, 143.46, 140.6, 140.3, 140.2, 138.4, 138.3, 137.1, 136.3, 134.6, 134.5, 134.2, 132.9, 131.6, 131.56, 131.33, 131.3, 131.2, 131.17, 128.9, 128.4, 128.31, 128.28, 128.1 (q, J_{C-F} = 3.6 Hz), 127.9 (q, J_{C-F} = 3.6 Hz), 127.6, 127.5, 127.44, 127.4, 127.2, 127.1, 127.06, 127.0, 125.5, 125.4, 124.1 (q, J_{C-F} = 273.4 Hz), 124.0 (q, J_{C-F} = 273.7 Hz), 123.8, 123.7, 123.63, 123.6, 121.8, 121.3, 119.5, 115.7. HRMS (m/z, ESI): calcd for $C_{38}H_{21}F_6^+$ $(M+H)^+$ 591.1469, found 591.1501.

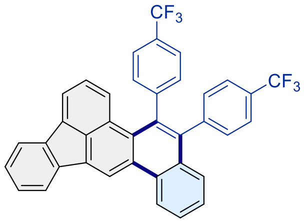

4,5-bis(4-(trifluoromethyl)phenyl)indeno[1,2,3-*hi*]chrysene (6l)

The general procedure A was followed. Yield: 93%, 55.0 mg. ^{1}H NMR (400 MHz, Chloroform-*d*) δ 9.29 (s, 1H), 8.99 (d, *J* = 8.4 Hz, 1H), 8.12 – 8.04 (m, 1H), 7.92 – 7.83 (m, 1H), 7.83 – 7.72 (m, 2H), 7.55 (t, *J* = 8.0 Hz, 1H), 7.49 (t, *J* = 7.6 Hz, 4H), 7.45 – 7.37 (m, 3H), 7.22 – 7.09 (m, 5H), 6.79 (d, *J* = 8.8 Hz, 1H). ^{13}C NMR (101 MHz, Chloroform-*d*) δ 146.5, 143.3, 140.7, 138.3, 137.2, 136.5, 136.1, 133.0, 131.7,

131.5, 131.46, 131.4, 131.2, 129.5 (q, $J_{C\text{-}F}$ = 32.7 Hz), 129.2 (q, $J_{C\text{-}F}$ = 32.6 Hz), 128.5, 128.4, 127.7, 127.6, 127.58, 127.5, 127.3, 127.2, 127.1, 125.44 (q, $J_{C\text{-}F}$ = 3.6 Hz), 124.82 (q, $J_{C\text{-}F}$ = 3.6 Hz), 124.23 (q, $J_{C\text{-}F}$ = 273.3 Hz), 124.2 (q, $J_{C\text{-}F}$ = 273.2 Hz), 123.7, 121.9, 121.4, 119.7, 115.8. HRMS (m/z, ESI): calcd for $C_{38}H_{21}F_6^+$ $(M+H)^+$ 591.1469, found 591.1454.

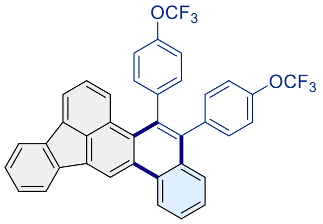

4,5-bis(4-(trifluoromethoxy)phenyl)indeno[1,2,3-*hi*]chrysene (6m)

The general procedure A was followed. Yield: 81%, 50.4 mg. ^{1}H NMR (400 MHz, Chloroform-*d*) δ 9.34 (s, 1H), 9.03 (d, *J* = 8.4 Hz, 1H), 8.18 – 8.05 (m, 1H), 7.94 – 7.86 (m, 1H), 7.82 (d, *J* = 6.8 Hz, 1H), 7.77 (t, *J* = 7.2 Hz, 1H), 7.61 – 7.51 (m, 2H), 7.48 – 7.39 (m, 2H), 7.19 (t, *J* = 8.0 Hz, 1H), 7.15 – 7.03 (m, 8H), 6.93 (d, *J* = 8.8 Hz, 1H). ^{13}C NMR (101 MHz, Chloroform-*d*) δ 148.3, 148.1, 141.7, 140.7, 138.7, 138.4, 138.2, 137.2, 136.5, 136.4, 133.0, 132.5, 132.4, 131.6, 131.5, 131.4, 128.6, 128.5, 128.0, 127.74, 127.7, 127.4, 127.3, 127.2, 127.1, 123.7, 121.8, 121.4, 121.2, 120.6 (q, $J_{C\text{-}F}$ = 258.1 Hz), 120.4, 119.6, 115.9. HRMS (m/z, ESI): calcd for $C_{38}H_{21}F_6O_2^+$ $(M+H)^+$ 623.1367, found 623.1337.

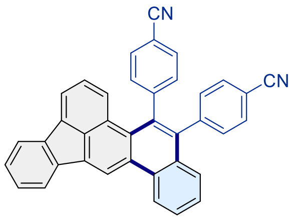

4,4'-(indeno[1,2,3-*hi*]chrysene-4,5-diyl)dibenzonitrile (6n)

The general procedure A was followed. Yield: 97%, 49.0 mg. ^{1}H NMR (400 MHz, Chloroform-*d*) δ 9.26 (s, 1H), 9.00 (d, *J* = 8.4 Hz, 1H), 8.13 – 8.04 (m, 1H), 7.94 – 7.85 (m, 1H), 7.84 – 7.76 (m, 2H), 7.60 – 7.48 (m, 5H), 7.48 – 7.41 (m, 2H), 7.34 (d, *J* = 8.4 Hz, 1H), 7.21 – 7.07 (m, 5H), 6.71 (d, *J* = 8.4 Hz, 1H). ^{13}C NMR (101 MHz, Chloroform-*d*) δ 147.5, 144.3, 140.6, 138.2, 137.6, 137.4, 136.8, 135.5, 132.9, 132.3, 132.26, 131.9, 131.85, 131.8, 131.4, 130.8, 128.7, 128.1, 127.9, 127.7, 127.6, 127.4,

127.3, 127.0, 126.8, 123.8, 121.9, 121.5, 119.8, 118.7, 118.6, 115.7, 111.4, 111.2. HRMS (m/z, ESI): calcd for $C_{38}H_{21}N_2^+$ $(M+H)^+$ 505.1699, found 505.1673.

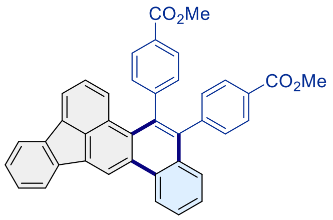

dimethyl 4,4'-(indeno[1,2,3-*hi*]chrysene-4,5-diyl)dibenzoate (6o)

The general procedure A was followed. Yield: 70%, 40.0 mg. ^{1}H NMR (400 MHz, Chloroform-*d*) δ 9.43 (s, 1H), 9.09 (d, *J* = 8.4 Hz, 1H), 8.21 – 8.14 (m, 1H), 8.00 – 7.90 (m, 5H), 7.86 (d, *J* = 6.8 Hz, 1H), 7.80 (t, *J* = 7.2 Hz, 1H), 7.55 (t, *J* = 7.6 Hz, 1H), 7.52 – 7.42 (m, 3H), 7.30 (d, *J* = 8.0 Hz, 2H), 7.22 (d, *J* = 8.2 Hz, 2H), 7.18 (d, *J* = 8.4 Hz, 1H), 6.93 (d, *J* = 8.4 Hz, 1H), 3.95 (s, 3H), 3.93 (s, 3H). ^{13}C NMR (101 MHz, Chloroform-*d*) δ 167.1, 147.8, 144.6, 140.8, 137.2, 136.5, 131.7, 131.4, 131.3, 131.28, 129.8, 129.2, 129.1, 128.9, 128.7, 128.6, 128.5, 127.8, 127.7, 127.6, 127.4, 127.3, 127.1, 123.7, 121.9, 121.4, 119.7, 118.6, 118.0, 115.9, 52.3, 52.3. HRMS (m/z, ESI): calcd for $C_{40}H_{27}O_4^+$ $(M+H)^+$ 571.1831, found 571.1847.

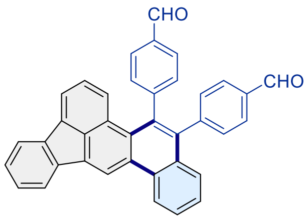

4,4'-(indeno[1,2,3-*hi*]chrysene-4,5-diyl)dibenzaldehyde (6p)

The general procedure A was followed. Yield: 89%, 45.6 mg. ^{1}H NMR (400 MHz, Chloroform-*d*) δ 9.99 (s, 1H), 9.97 (s, 1H), 9.31 (s, 1H), 9.01 (d, *J* = 8.4 Hz, 1H), 8.13 – 8.07 (m, 1H), 7.90 – 7.85 (m, 1H), 7.82 – 7.77 (m, 2H), 7.74 (dd, *J* = 8.0, 5.2 Hz, 4H), 7.54 (t, *J* = 8.0 Hz, 1H), 7.47 – 7.39 (m, 3H), 7.28 (d, *J* = 8.0 Hz, 2H), 7.23 (d, *J* = 8.0 Hz, 2H), 7.13 (dd, *J* = 8.4, 7.2 Hz, 1H), 6.83 (d, *J* = 8.4 Hz, 1H). ^{13}C NMR (101 MHz, Chloroform-*d*) δ 192.04, 191.99, 149.3, 146.1, 140.6, 138.3, 138.2, 137.2, 136.6, 136.1, 135.0, 134.9, 133.0, 131.9, 131.87, 131.7, 131.4, 131.0, 129.8, 129.2, 128.6, 128.4, 127.8, 127.5, 127.4, 127.2, 127.1, 123.8, 121.9, 121.4, 119.7, 115.8. HRMS (m/z, ESI): calcd for $C_{38}H_{23}O_2^+$ $(M+H)^+$ 511.1620, found 511.1632.

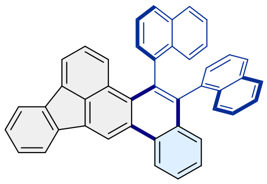

4,5-di(naphthalen-1-yl)indeno[1,2,3-*hi*]chrysene (6q)

The general procedure A was followed. Yield: 69%, 38.5 mg. ^{1}H NMR (400 MHz, Chloroform-*d*) δ 9.57 (s, 1H), 9.19 (d, *J* = 8.8 Hz, 1H), 8.20 (d, *J* = 7.2 Hz, 1H), 7.87 (d, *J* = 6.8 Hz, 1H), 7.83 – 7.70 (m, 4H), 7.62 (d, *J* = 8.4 Hz, 1H), 7.60 – 7.53 (m, 2H), 7.51 (d, *J* = 9.2 Hz, 1H), 7.49 – 7.38 (m, 5H), 7.37 – 7.26 (m, 3H), 7.15 (d, *J* = 7.2 Hz, 1H), 7.01 – 6.85 (m, 4H), 6.74 (d, *J* = 8.8 Hz, 1H). ^{13}C NMR (101 MHz, Chloroform-*d*) δ 140.8, 140.5, 139.1, 138.6, 137.3, 136.9, 136.8, 136.2, 133.4, 133.2, 133.1, 132.9, 132.5, 131.6, 130.1, 129.6, 129.2, 128.4, 128.3, 128.2, 128.1, 127.7, 127.66, 127.6, 127.3, 127.1, 127.05, 126.95, 126.7, 126.5, 126.1, 126.0, 125.8, 125.6, 125.4, 125.3, 124.8, 124.5, 123.7, 121.8, 121.3, 119.5, 116.3, 116.2. HRMS (m/z, ESI): calcd for $C_{44}H_{27}^+$ $(M+H)^+$ 555.2035, found 555.2013.

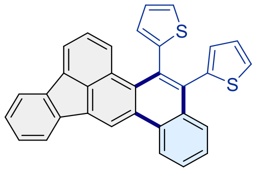

2,2'-(indeno[1,2,3-hi]chrysene-4,5-diyl)dithiophene (6r)

The general procedure A was followed. Yield: 85%, 39.8 mg. ^{1}H NMR (400 MHz, Chloroform-*d*) δ 9.33 (s, 1H), 9.00 (d, *J* = 8.4 Hz, 1H), 8.17 – 8.07 (m, 1H), 7.93 – 7.87 (m, 1H), 7.86 (d, *J* = 6.8 Hz, 1H), 7.83 – 7.72 (m, 2H), 7.58 (t, *J* = 8.0 Hz, 1H), 7.47 – 7.41 (m, 2H), 7.38 (dd, *J* = 10.4, 5.2 Hz, 2H), 7.31 (t, *J* = 8.4 Hz, 1H), 7.11 (d, *J* = 8.8 Hz, 1H), 7.04 (q, *J* = 4.0 Hz, 2H), 6.94 (dd, *J* = 7.2, 3.2 Hz, 2H). ^{13}C NMR (101 MHz, Chloroform-*d*) δ 143.9, 140.9, 139.9, 138.4, 137.0, 136.7, 134.7, 133.0, 132.7, 132.3, 131.9, 131.6, 129.7, 129.23, 129.2, 128.8, 128.5, 128.1, 127.64, 127.6, 127.4, 127.3, 127.2, 127.1, 126.9, 126.4, 126.3, 123.5, 121.8, 121.3, 119.7, 115.7. HRMS (m/z, ESI): calcd for $C_{32}H_{19}S_2^+$ $(M+H)^+$ 467.0850, found 467.0842.

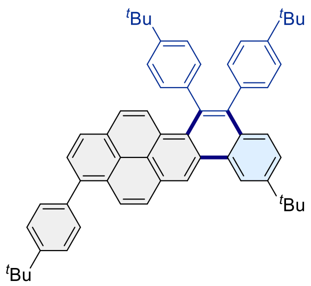

8-(*tert*-butyl)-3,11,12-tris(4-(*tert*-butyl)phenyl)benzo[*pqr*]picene (6s)

The general procedure A was followed. Yield: 46%, 34.6 mg. ^{1}H NMR (400 MHz, Chloroform-*d*) δ 9.56 (s, 1H), 9.16 (s, 1H), 8.29 – 8.21 (m, 2H), 8.17 (d, *J* = 9.6 Hz, 1H), 8.10 (d, *J* = 8.0 Hz, 1H), 7.95 (d, *J* = 8.0 Hz, 1H), 7.77 (d, *J* = 8.8 Hz, 1H), 7.70 (d, *J* = 8.4 Hz, 1H), 7.67 – 7.56 (m, 5H), 7.31 – 7.21 (m, 2H), 7.16 (d, *J* = 8.0 Hz, 2H), 7.05 (d, *J* = 8.0 Hz, 2H), 6.98 (d, *J* = 8.0 Hz, 2H), 1.61 (s, 9H), 1.49 (s, 9H), 1.35 (s, 9H), 1.33 (s, 9H). ^{13}C NMR (101 MHz, Chloroform-*d*) δ 150.2, 149.4, 148.94, 148.9, 141.2, 139.4, 138.6, 137.5, 137.3, 136.5, 131.0, 130.9, 130.5, 129.95, 129.9, 129.8, 129.5, 129.3, 128.9, 128.3, 128.2, 128.0, 127.9, 126.7, 126.0, 125.5, 125.4, 125.3, 125.2, 124.7, 124.2, 119.5, 119.2, 35.5, 34.8, 34.6, 34.5, 31.8, 31.7, 31.6, 31.5. HRMS (m/z, ESI): calcd for $C_{58}H_{59}^+$ $(M+H)^+$ 755.4539, found 755.4521.

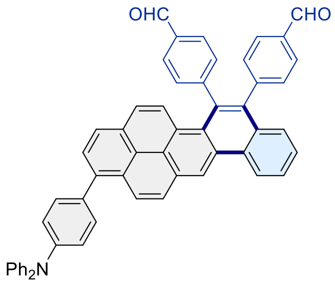

4,4'-(3-(4-(diphenylamino)phenyl)benzo[*pqr*]picene-11,12-diyl)dibenzaldehyde (6t)

The general procedure A was followed. Yield: 72%, 54.1 mg. ^{1}H NMR (400 MHz, Chloroform-*d*) δ 9.93 (s, 1H), 9.89 (s, 1H), 9.40 (s, 1H), 9.06 (d, *J* = 8.4 Hz, 1H), 8.23 (d, *J* = 9.2 Hz, 1H), 8.10 (d, *J* = 9.6 Hz, 1H), 8.00 (d, *J* = 8.0 Hz, 1H), 7.89 (d, *J* = 8.0 Hz, 1H), 7.77 – 7.65 (m, 4H), 7.59 (d, *J* = 7.6 Hz, 2H), 7.51 (t, *J* = 9.6 Hz, 2H), 7.41 (d, *J* = 8.4 Hz, 3H), 7.25 (t, *J* = 8.0 Hz, 4H), 7.22 – 7.11 (m, 10H), 7.00 (t, *J* = 7.2 Hz, 2H). ^{13}C NMR (101 MHz, Chloroform-*d*) δ 192.1, 192.0, 150.3, 147.9, 147.4, 146.7, 138.0, 137.6, 135.4, 135.0, 134.9, 134.5, 132.1, 132.0, 131.5, 130.8, 130.6, 130.4,

129.7, 129.6, 129.5, 129.3, 128.72, 128.69, 128.6, 128.0, 127.6, 127.55, 127.5, 127.1, 126.7, 125.6, 125.3, 125.1, 125.0, 124.8, 123.8, 123.3, 119.5. HRMS (m/z, ESI): calcd for $C_{56}H_{36}NO_2^+$ $(M+H)^+$ 754.2668, found 754.2647.

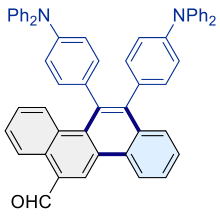

11,12-bis(4-(diphenylamino)phenyl)chrysene-6-carbaldehyde (6u)

The general procedure A was followed. Yield: 64%, 47.9 mg. ^{1}H NMR (400 MHz, Chloroform-*d*) δ 10.47 (s, 1H), 9.24 (d, *J* = 8.0 Hz, 1H), 9.12 (s, 1H), 8.76 (d, *J* = 8.4 Hz, 1H), 7.92 (d, *J* = 8.8 Hz, 1H), 7.74 (d, *J* = 8.0 Hz, 1H), 7.66 (t, *J* = 7.2 Hz, 1H), 7.50 (q, *J* = 7.6 Hz, 2H), 7.22 – 7.10 (m, 9H), 7.08 – 6.98 (m, 8H), 6.92 (t, *J* = 8.0 Hz, 6H), 6.88 – 6.80 (m, 4H), 6.75 (d, *J* = 8.0 Hz, 2H). ^{13}C NMR (101 MHz, Chloroform-d) δ 193.4, 147.9, 147.8, 146.5, 146.3, 143.4, 137.5, 136.7, 134.3, 133.6, 132.1, 132.0, 131.9, 131.8, 130.32, 130.3, 130.1, 129.5, 129.4, 129.3, 128.2, 127.9, 127.6, 127.3, 125.2, 124.9, 124.5, 124.2, 124.0, 123.0, 122.94, 122.87. HRMS (m/z, ESI): calcd for $C_{55}H_{39}N_2O^+$ $(M+H)^+$ 743.2984, found 743.2968.

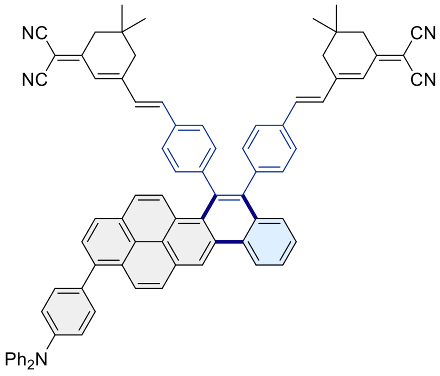

2,2'-(((1*E*,1'*E*)-((3-(4-(diphenylamino)phenyl)benzo[*pqr*]picene-11,12-diyl)bis(4,1-phenylene))bis(ethene-2,1-diyl))bis(5,5-dimethylcyclohex-2-en-3-yl-1-ylidene))di malononitrile (6v)

The general procedure A was followed (note: 0.05 mmol scale reaction was carried out). Yield: 42%, 22.9 mg. ^{1}H NMR (400 MHz, Chloroform-*d*) δ 9.38 (s, 1H), 9.04 (d, *J* = 8.4 Hz, 1H), 8.22 (d, *J* = 9.2 Hz, 1H), 8.08 (d, *J* = 9.6 Hz, 1H), 7.94 (d, *J* = 8.0 Hz, 1H), 7.87 (d, *J* = 8.0 Hz, 1H), 7.81 (d, *J* = 9.6 Hz, 1H), 7.76 – 7.68 (m, 1H), 7.56 –

7.48 (m, 3H), 7.40 (d, J = 8.4 Hz, 2H), 7.34 (d, J = 8.0 Hz, 2H), 7.29 – 7.20 (m, 5H), 7.21 – 7.11 (m, 7H), 7.10 (d, J = 8.0 Hz, 2H), 7.05 (d, J = 8.0 Hz, 2H), 6.99 (t, J = 7.2 Hz, 2H), 6.86 (d, J = 14.8 Hz, 2H), 6.82 (d, J = 13.6 Hz, 2H), 6.61 (s, 1H), 6.56 (s, 1H), 2.37 (s, 2H), 2.29 (s, 2H), 2.21 (s, 2H), 2.16 (s, 2H), 0.91 (s, 6H), 0.86 (s, 6H). ^{13}C NMR (101 MHz, Chloroform-*d*) δ 169.2, 169.1, 153.7, 153.6, 147.8, 147.3, 145.7, 141.9, 138.3, 137.8, 136.7, 135.9, 134.9, 134.1, 133.8, 132.1, 132.0, 131.5, 131.4, 130.3, 129.6, 129.55, 129.5, 129.45, 129.2, 129.1, 128.9, 128.7, 128.5, 128.2, 127.6, 127.32, 127.3, 127.1, 126.5, 125.6, 125.5, 125.4, 125.1, 125.0, 124.8, 123.7, 123.6, 123.5, 123.48, 123.3, 123.2, 119.6, 113.51, 113.49, 112.8, 112.7, 78.8, 78.7, 42.9, 42.8, 39.2, 39.1, 32.0, 31.9, 28.1, 28.0. HRMS (m/z, ESI): calcd for $C_{80}H_{60}N_5^+$ $(M+H)^+$ 1090.4770, found 1090.4719.

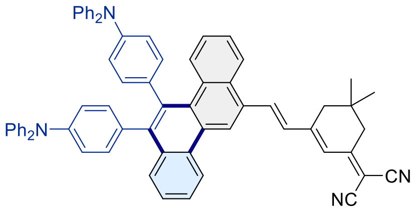

(*E*)-2-(3-(2-(11,12-bis(4-(diphenylamino)phenyl)chrysen-6-yl)vinyl)-5,5-dimethylcyclohex-2-en-1-ylidene)malononitrile (**6w**)

The general procedure A was followed (note: 0.05 mmol scale reaction was carried out). Yield: 40%, 18.0 mg. ^{1}H NMR (400 MHz, Chloroform-*d*) δ 8.98 (s, 1H), 8.84 (d, J = 8.4 Hz, 1H), 8.11 (d, J = 8.0 Hz, 1H), 7.96 (d, J = 8.8 Hz, 1H), 7.88 (d, J = 15.6 Hz, 1H), 7.74 (d, J = 8.4 Hz, 1H), 7.70 (t, J = 7.2 Hz, 1H), 7.52 (t, J = 7.6 Hz, 1H), 7.46 (t, J = 7.2 Hz, 1H), 7.24 – 7.13 (m, 10H), 7.04 (t, J = 8.4 Hz, 8H), 6.97 – 6.91 (m, 7H), 6.90 – 6.85 (m, 4H), 6.82 (d, J = 8.4 Hz, 2H), 2.56 (s, 2H), 2.55 (s, 2H), 1.08 (s, 6H). ^{13}C NMR (101 MHz, Chloroform-*d*) δ 169.4, 153.9, 148.0, 147.9, 146.4, 146.1, 140.8, 138.1, 136.6, 134.6, 134.0, 132.3, 132.2, 132.0, 131.7, 131.5, 130.1, 129.9, 129.45, 129.43, 129.2, 129.0, 128.1, 127.2, 126.2, 124.7, 124.4, 124.3, 124.2, 124.1, 123.4, 123.2, 123.1, 122.9, 122.8, 120.8, 113.6, 112.9, 79.2, 43.2, 39.5, 32.3, 28.3. HRMS (m/z, ESI): calcd for $C_{67}H_{51}N_4^+$ $(M+H)^+$ 911.4035, found 911.4030.

3.4 Mechanistic Studies

Control experiments:

(a) Reaction of **1a** with **NBE-CO$_2$Me** in the condition of quantitative Pd(OAc)$_2$:

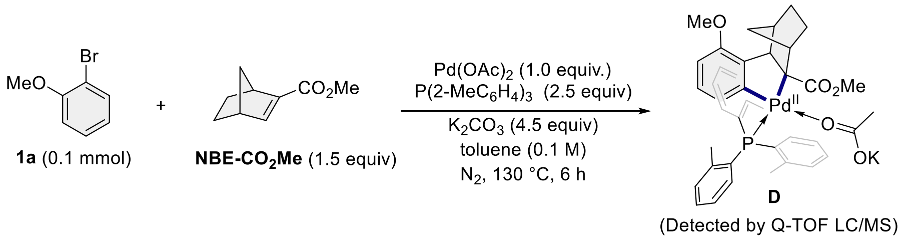

The oven-dried Schlenk tube (10 mL) equipped with a stirring bar was charged with substrates **1a** (0.1 mmol, 1.0 equiv), Pd(OAc)$_2$ (1.0 equiv), P(2-MeC$_6$H$_4$)$_3$ (2.5 equiv) and K$_2$CO$_3$ (4.5 equiv) in the air. Followed by the mediator NBE-CO$_2$Me (1.5 equiv) and the anhydrous toluene (1.0 mL) were added via syringe under nitrogen flow. Then the tube was evacuated and back-filled with N$_2$ for three times. Finally, the reaction mixture was stirred at 130 ℃ for 6 h and then cooled to room temperature. Upon completion of the reaction, the mixture was analyzed by the Agilent 6546 Q-TOF LC/MS system detection and intermediate **D** were detected.

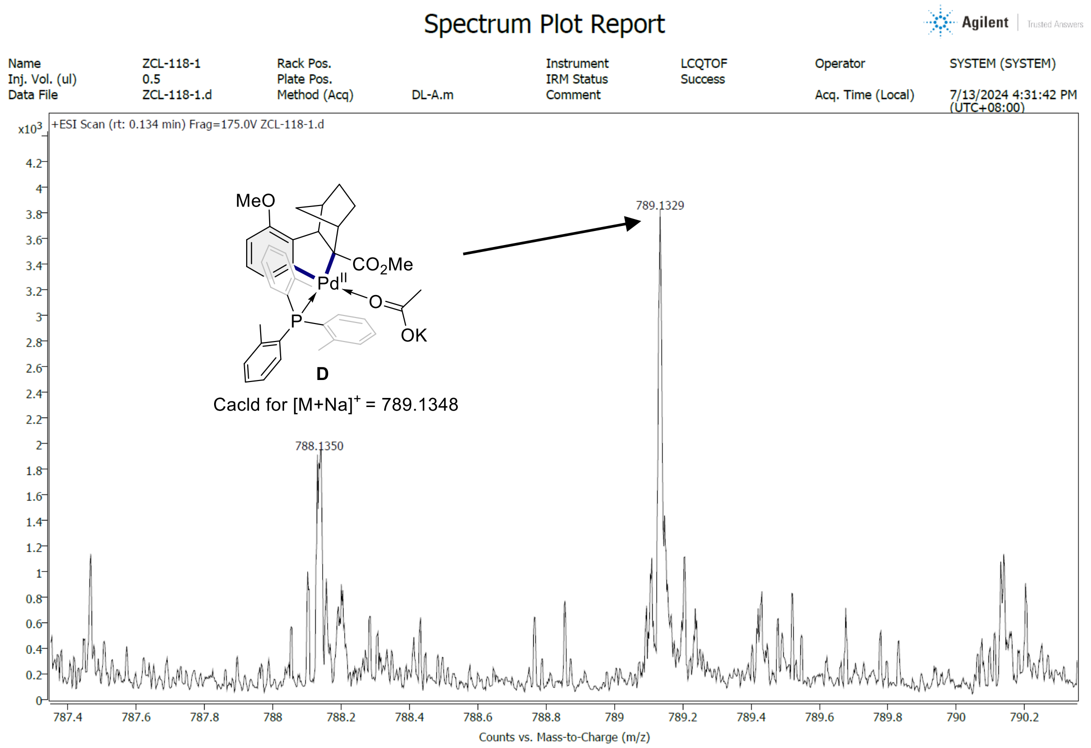

Fig. S4. MS spectrum of intermediate **D**.

(b) Reaction of **1a** with **NBE-CO_2Me** and **2a** in the condition of quantitative Pd$(OAc)_2$:

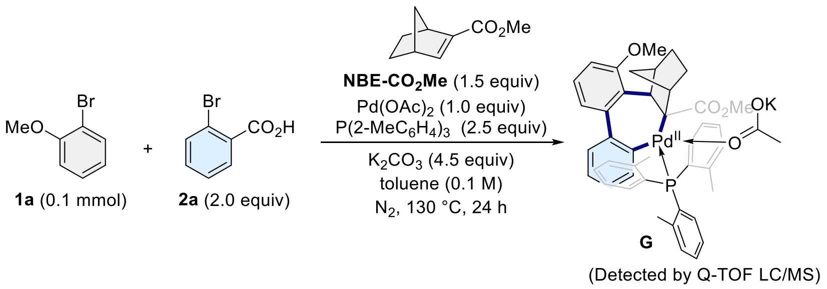

The oven-dried Schlenk tube (10 mL) equipped with a stirring bar was charged with substrates **1a** (0.1 mmol, 1.0 equiv), **2a** (2.0 equiv), Pd$(OAc)_2$ (1.0 equiv), P(2-Me$C_6H_4)_3$ (2.5 equiv) and K_2CO_3 (4.5 equiv) in the air. Followed by the mediator NBE-CO_2Me (1.5 equiv) and the anhydrous toluene (1.0 mL) were added via syringe under nitrogen flow. Then the tube was evacuated and back-filled with N_2 for three times. Finally, the reaction mixture was stirred at 130 ℃ for 24 h and then cooled to room temperature. Upon completion of the reaction, the mixture was analyzed by the Agilent 6546 Q-TOF LC/MS system detection and intermediate **G** were detected.

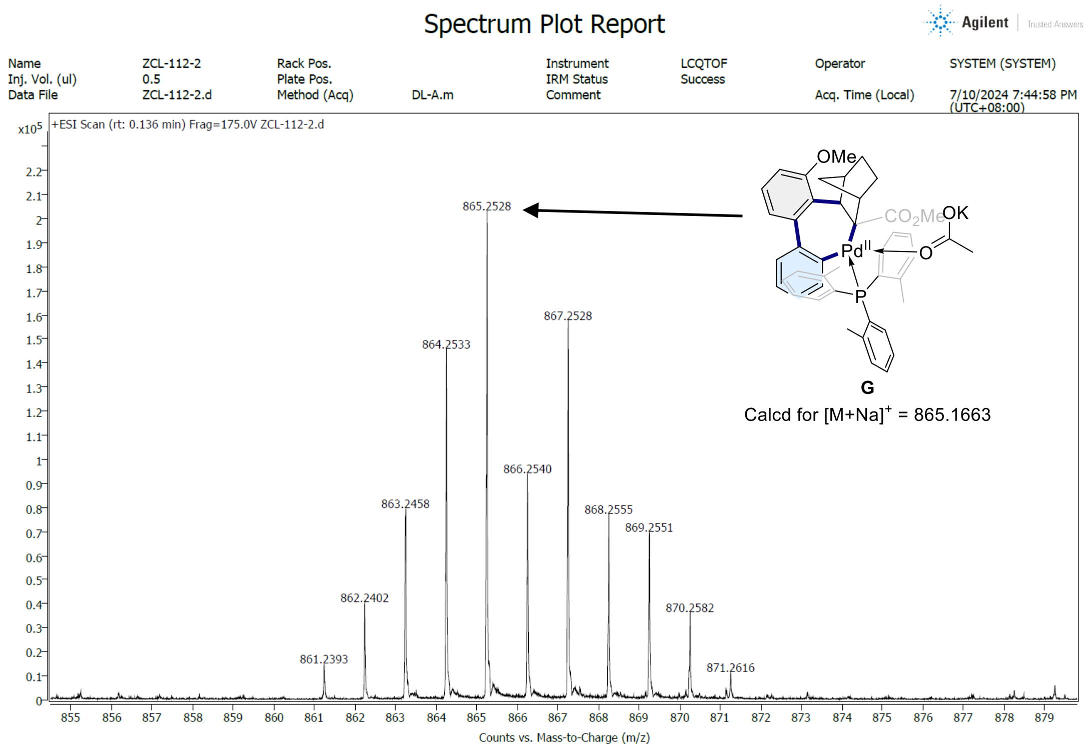

Fig. S5. MS spectrum of intermediate **G**.

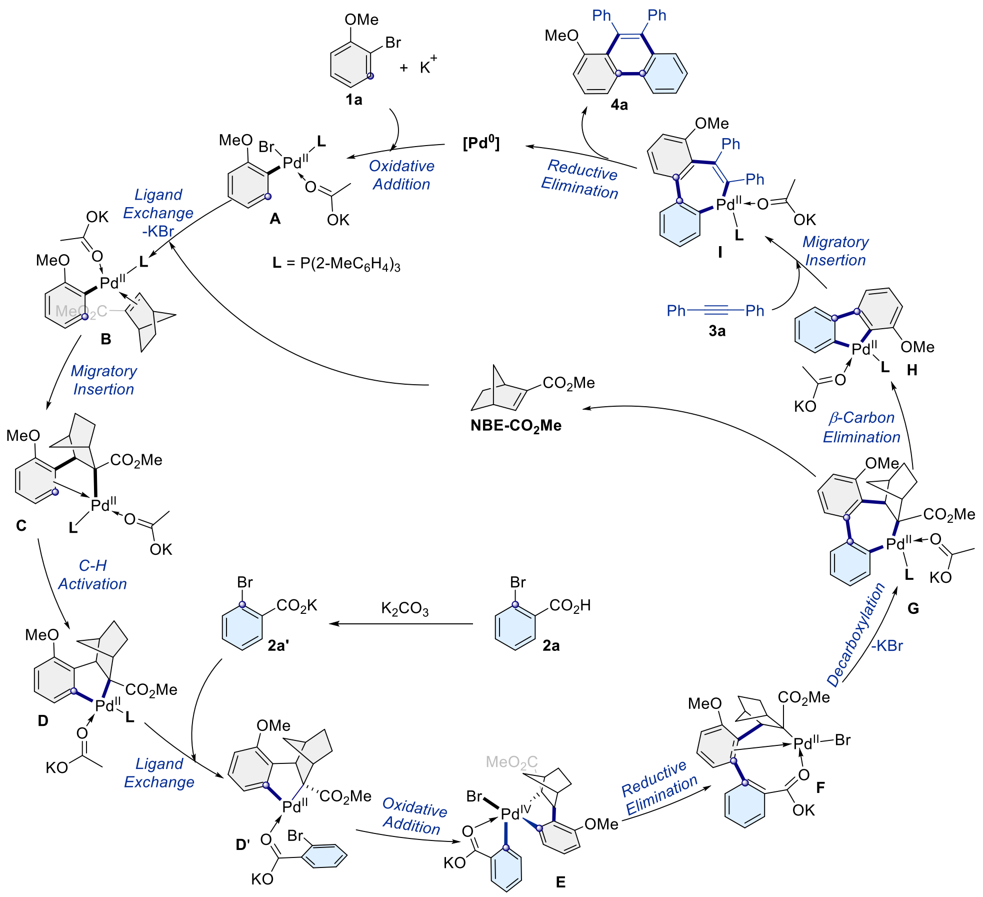

Fig. S6. Proposed reaction mechanism.

3.5 General procedures for the synthesis of substrates

(a)The synthesis of substrates 1t and 1u:

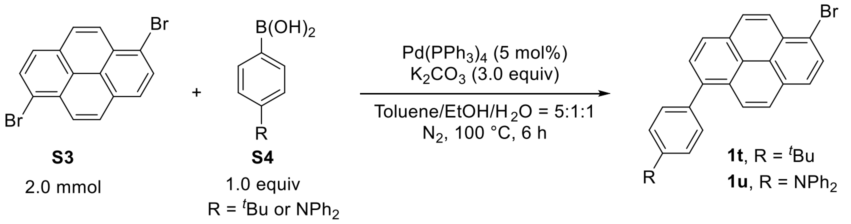

The oven-dried Schlenk tube (100 mL) equipped with a stirring bar was charged with 1,6-dibromopyrene (**S3**, 2.0 mmol, 1.0 equiv), 4-tert-butylphenylboronic acid or 4-(diphenylamino)phenyl boronic acid (**S4** 1.0 equiv), $Pd(PPh_3)_4$ (5 mol%), and K_2CO_3 (4.5 equiv) in the air. Followed by the toluene/EtOH/H_2O (5/1/1, 10 mL) were added via syringe under nitrogen flow. Then the tube was evacuated and back-filled with N_2 for three times. Finally, the reaction mixture was stirred at 100 ℃ for 6 h and then cooled to room temperature. Upon completion of the reaction, the mixture was

quenched by 2N HCl aqueous solution and ethyl acetate (EA). The organic phases were separated and the aqueous phase was extracted with EA three times. The combined organic phases were washed with brine, dried with anhydrous Na_2SO_4 and concentrated *in vacuo*. The resulting residue was purified via silica gel column chromatography using PE/EA (200/1 – 20/1) as the eluent.

Characterization of substrates 1t and 1u:

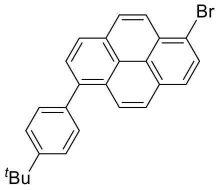

1-bromo-6-(4-(*tert*-butyl)phenyl)pyrene (1t): ^{1}H NMR (400 MHz, Chloroform-*d*) δ 8.45 (d, *J* = 9.2 Hz, 1H), 8.30 – 8.21 (m, 3H), 8.19 (d, *J* = 9.2 Hz, 1H), 8.02 (d, *J* = 8.0 Hz, 1H), 8.00 – 7.92 (m, 2H), 7.64 – 7.52 (m, 4H), 1.46 (s, 9H). ^{13}C NMR (101 MHz, Chloroform-*d*) δ 150.5, 138.7, 138.0, 130.6, 130.4, 130.35, 130.3, 130.1, 129.2, 128.7, 128.5, 127.1, 126.3, 126.0, 125.9, 125.5, 125.4, 125.3, 124.5, 120.0, 34.8, 31.6. HRMS (m/z, ESI): calcd for $C_{26}H_{24}Br^+$ $(M+H)^+$ 415.0983, found 415.0795.

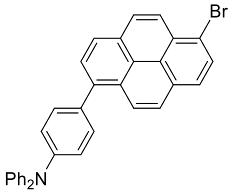

4-(6-bromopyren-1-yl)-*N,N*-diphenylaniline (1u): ^{1}H NMR (400 MHz, Chloroform-*d*) δ 8.44 (d, *J* = 9.2 Hz, 1H), 8.31 (d, *J* = 9.2 Hz, 1H), 8.24 (dd, *J* = 7.6, 6.0 Hz, 2H), 8.18 (d, *J* = 9.2 Hz, 1H), 8.03 (d, *J* = 8.0 Hz, 1H), 7.99 (d, *J* = 8.8 Hz, 2H), 7.50 (d, *J* = 8.4 Hz, 2H), 7.34 (t, *J* = 8.0 Hz, 4H), 7.30 – 7.18 (m, 6H), 7.09 (t, *J* = 7.2 Hz, 2H). ^{13}C NMR (101 MHz, Chloroform-*d*) δ 147.8, 147.4, 138.4, 134.7, 131.5, 130.6, 130.31, 130.29, 130.1, 129.5, 129.2, 128.6, 128.4, 127.1, 126.3, 125.9, 125.4, 125.36, 124.8, 124.6, 123.3, 120.0. HRMS (m/z, ESI): calcd for $C_{34}H_{25}BrN^+$ $(M+H)^+$ 526.1092, found 526.0902.

(b) The synthesis of symmetrical 1,2-diarylalkynes:

All the symmetrical 1,2-diarylalkynes were prepared according to the reported literatures[S2].

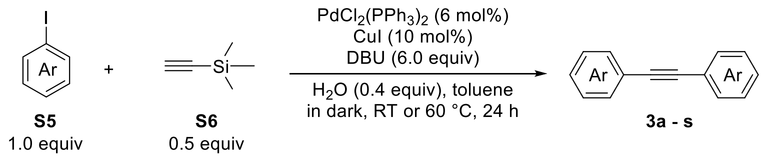

(c) The synthesis of norbornene mediators:

The structurally modified norbornenes were synthesized following the procedure as described in literatures[S3, S4].

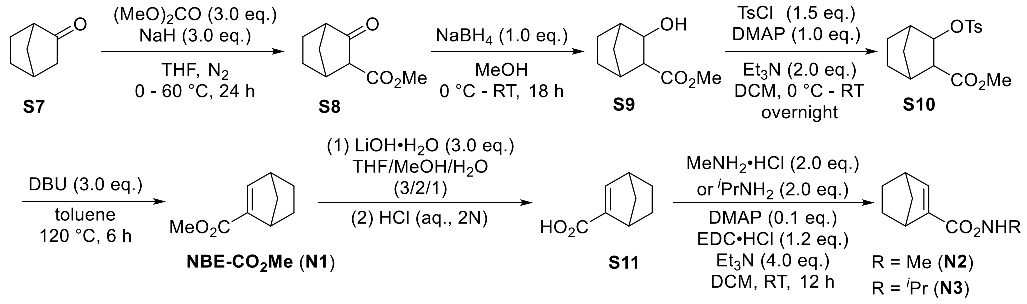

4. Photophysical properties and applications of products.

4.1. The absorption and fluorescence properties of products

Fluorescence spectra were collected on HITACHI F-700003040428 Fluorescence Spectrometer. UV−vis spectra were recorded on Agilent Cary60 spectrometer. Samples for the experiments were prepared in a 4.0 mL glass cuvette with a septum screw cap. In a typical experiment, a 5.0×10^{-5} M and 1.0×10^{-6} M solution of product in DCM for the collection of emission spectrum and UV-vis spectrum were prepared respectively.

Table S2. Absorption maxima and emission maxima of products.

Compunds	Absorb.(λ_{abs}, nm) [a]	Emission (λ_{em}, nm) [b]	CIE_{1931}
**4a**	258, 303, 345, 362	370, 388	(0.16, 0.02)
**4b**	264, 298, 310	366, 381	(0.16, 0.03)
**4c**	265, 299, 310	369, 386	(0.16, 0.04)
**4d**	277, 319, 333	382, 399	(0.16, 0.02)
**4e**	277, 322, 335	386, 404	(0.16, 0.02)
**4f**	279, 327, 341, 378	393, 409	(0.16, 0.02)
**4g**	273, 317, 332, 373	384, 402	(0.16, 0.02)
**4h**	281, 345	415	(0.16, 0.03)
**4i**	282, 341, 356	409	(0.16, 0.02)
**4j**	255, 288, 360	435	(0.15, 0.06)
**4k**	279, 326, 341, 376	385, 403	(0.16, 0.01)
**4l**	267, 282, 292, 320, 371, 392	445	(0.15, 0.08)
**4m**	251, 290, 306, 319, 377, 398	428, 452	(0.15, 0.04)
**4n**	298, 333	404	(0.16, 0.02)
**4o**	252, 275, 327, 433	496	(0.15, 0.49)
**4p**	272, 332, 370	388, 401	(0.16, 0.02)
**4q**	310, 376, 395	428	(0.16, 0.03)
**4r**	290, 352, 370	379, 395, 415	(0.17, 0.01)
**4s**	265, 296, 359, 377	383, 403, 425	(0.17, 0.01)
**5a**	268, 284, 320, 375, 395	446	(0.15, 0.07)
**5b**	261, 284, 296, 320, 372, 391	445	(0.15, 0.08)
**5c**	264, 302, 322, 370, 388	446	(0.15, 0.08)
**5d**	283, 319, 373, 395	444	(0.15, 0.08)
**5e**	262, 281, 292, 320, 367, 385	450	(0.15, 0.13)
**5f**	271, 283, 322, 373, 395	445	(0.15, 0.10)
**5g**	285, 296, 321, 369, 388	451	(0.15, 0.13)
**5h**	286, 320, 370, 392	454	(0.15, 0.14)
**5i**	290, 356, 388	551	(0.37, 0.58)
**6a**	264, 282, 318, 372, 390	444	(0.15, 0.08)
**6b**	262, 284, 318, 372, 392	443	(0.15, 0.07)
**6c**	264, 282, 318, 372, 392	440	(0.15, 0.07)
**6d**	266, 282, 320, 373, 393	442	(0.15, 0.07)

**6e**	265, 282, 319, 373, 394	442	(0.15, 0.07)
**6f**	293, 376	527	(0.26, 0.60)
**6g**	264, 288, 318, 368, 386	449	(0.15, 0.13)
**6h**	264, 282, 318, 370, 390	446	(0.15, 0.10)
**6i**	264, 288, 318, 368, 388	449	(0.15, 0.12)
**6j**	264, 286, 318, 370, 388	446	(0.15, 0.10)
**6k**	288, 318, 377, 388	451	(0.15, 0.13)
**6l**	264, 288, 318, 368, 388	451	(0.15, 0.13)
**6m**	266, 288, 318, 370, 388	447	(0.15, 0.11)
**6n**	288, 320, 368, 388	456	(0.15, 0.15)
**6o**	260, 288, 320, 368, 388	448	(0.15, 0.12)
**6p**	260, 288, 318, 370, 388	449	(0.15, 0.12)
**6q**	288, 316, 370, 390	446	(0.15, 0.10)
**6r**	264, 282, 320, 372, 388	454	(0.15, 0.13)
**6s**	256, 292, 326, 392, 412	448, 463	(0.14, 0.09)
**6t**	288, 416	523	(0.24, 0.61)
**6u** [c]	290, 352	412, 436, 561	(0.31, 0.34)
**6v**	290, 414	535	(0.32, 0.62)
**6w** [d]	278, 306, 450	394, 418, 571	(0.33, 0.33)
**6w NPs** [e]	281, 305, 459	426, 465, 610	(0.33, 0.28)

[a] Absorption maximum in CH_2Cl_2 (1×10^{-6} M). [b] Emission maximum in CH_2Cl_2 (5.0×10^{-5} M). [c] CH_2Cl_2 used as solvent (2.5×10^{-7} M). [d] Toluene used as solvent (2.4×10^{-6} M). [e] H_2O used as solvent (2.0×10^{-5} M).

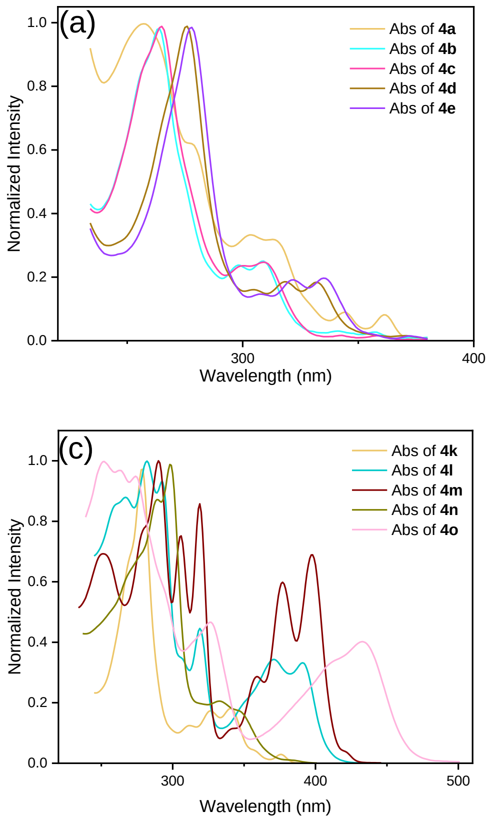

Fig. S7. Normalized UV-Vis absorption spectra of **4a-4s** in DCM (1×10^{-6} M).

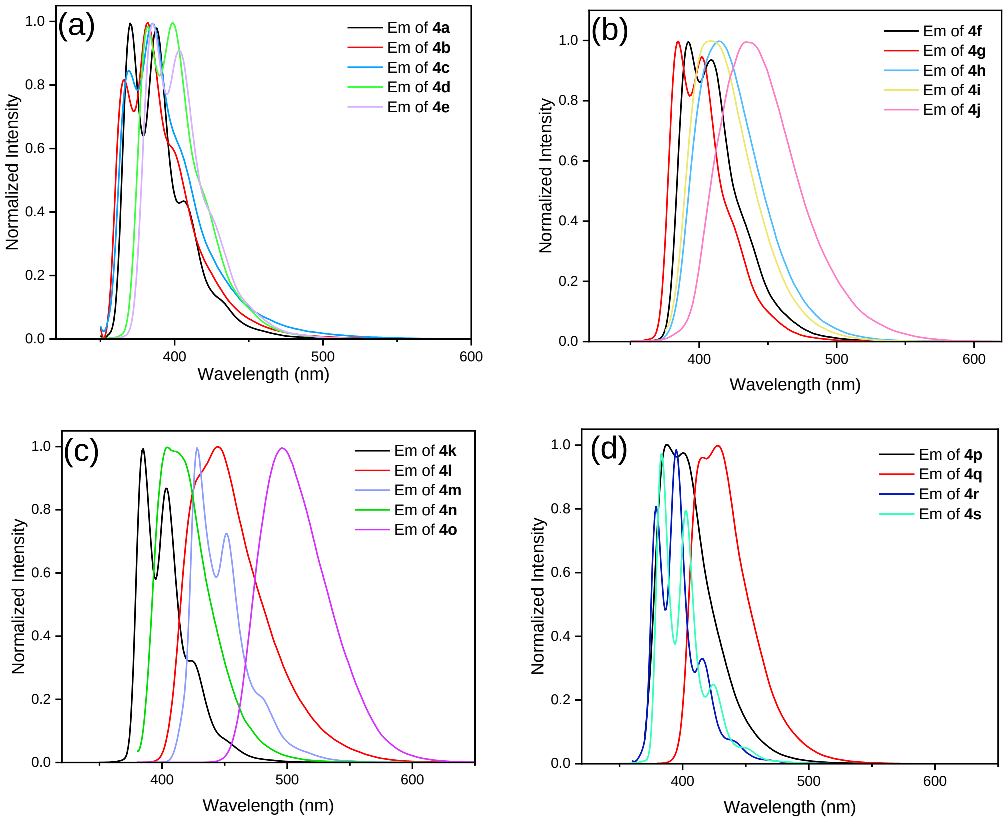

Fig. S8. Normalized fluorescence emission spectra of **4a-4s** in DCM (5.0×10^{-5} M).

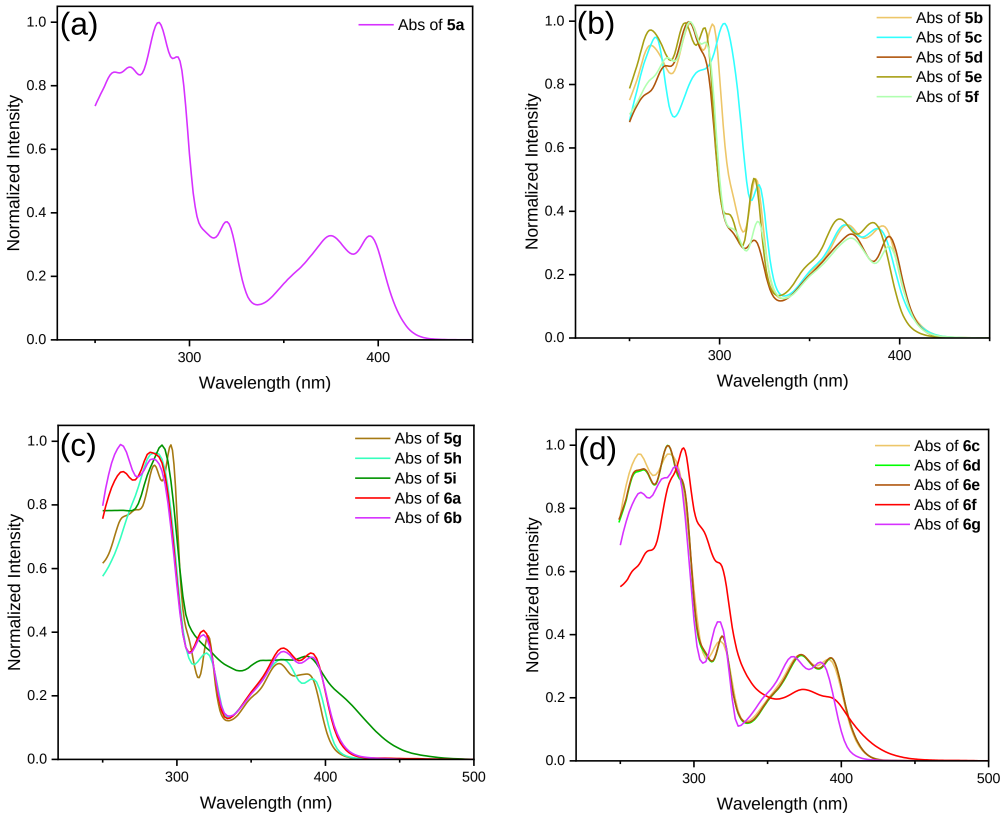

Fig. S9. Normalized UV-Vis absorption spectra of **5a-6g** in DCM (1×10^{-6} M).

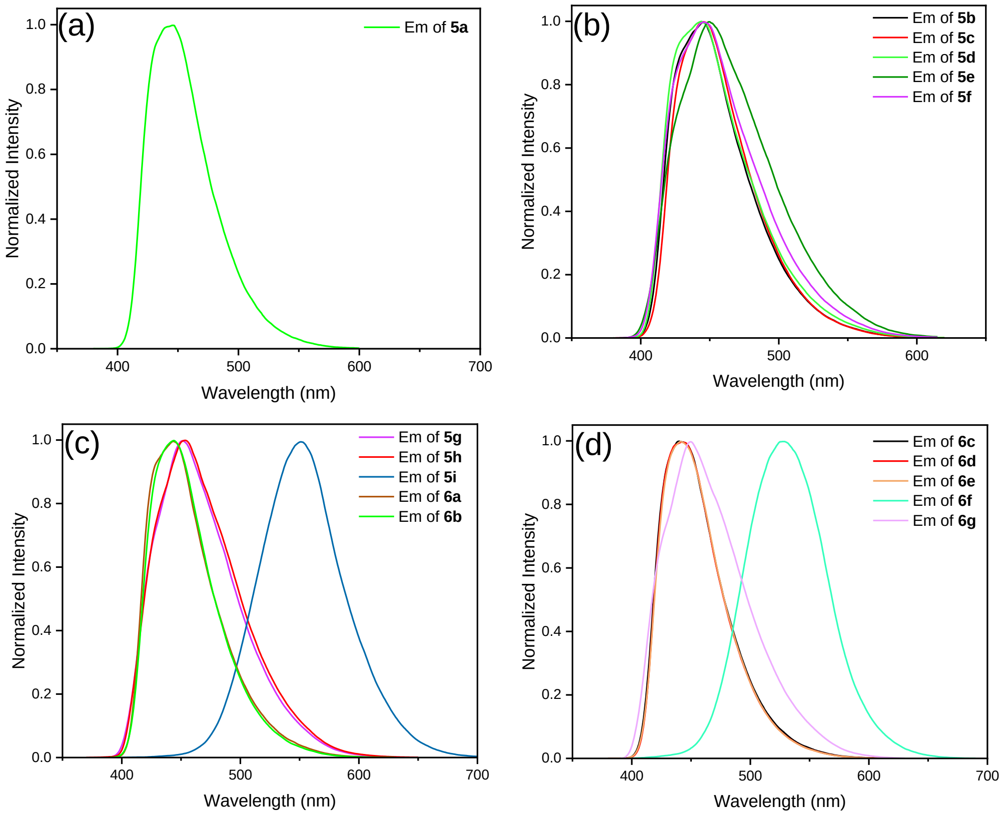

Fig. S10. Normalized fluorescence emission spectra of **5a-6g** in DCM (5.0×10^{-5} M).

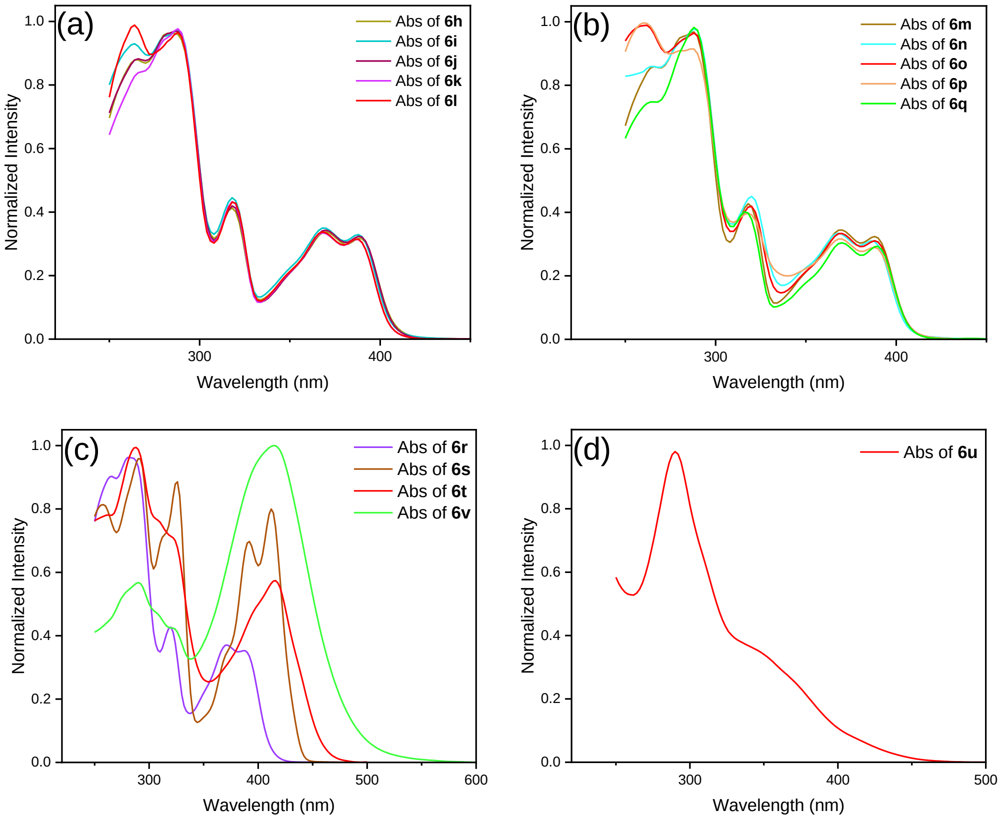

Fig. S11. Normalized UV-Vis absorption spectra of **6h-6v** in DCM (1×10^{-6} M).

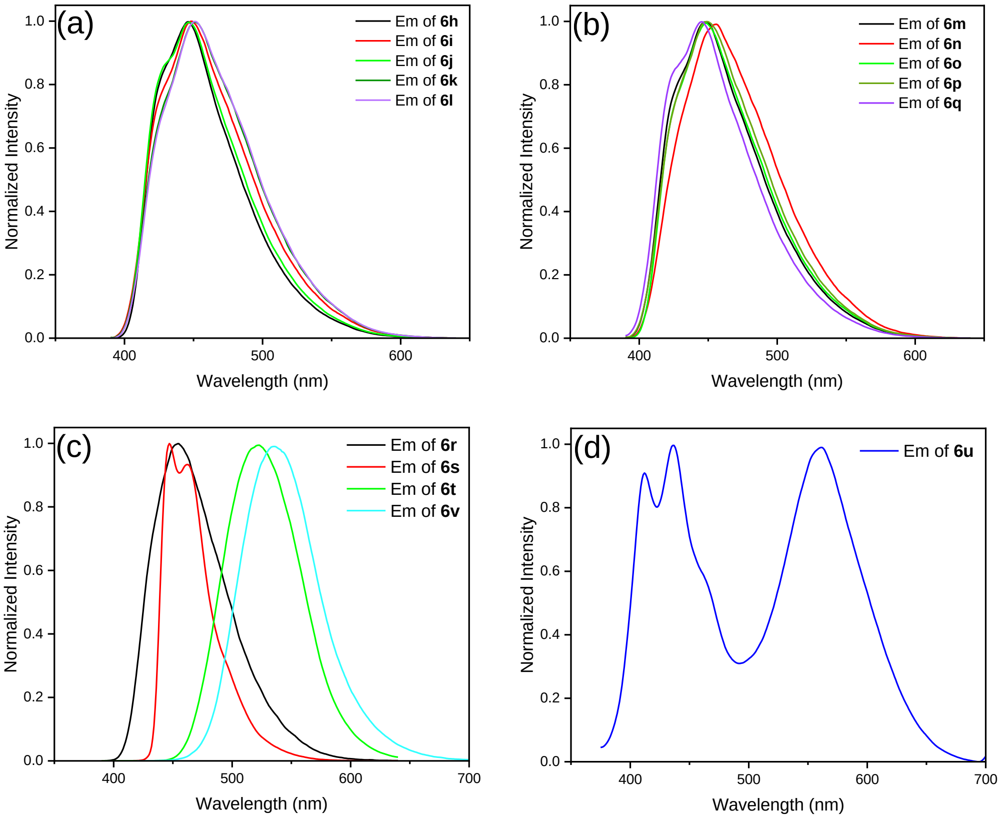

Fig. S12. (a - c) Normalized fluorescence emission spectra of **6h-6t** and **6v** in DCM

(5.0×10^{-5} M). (d) Normalized fluorescence emission spectra of **6u** in DCM (2.5×10^{-7} M).

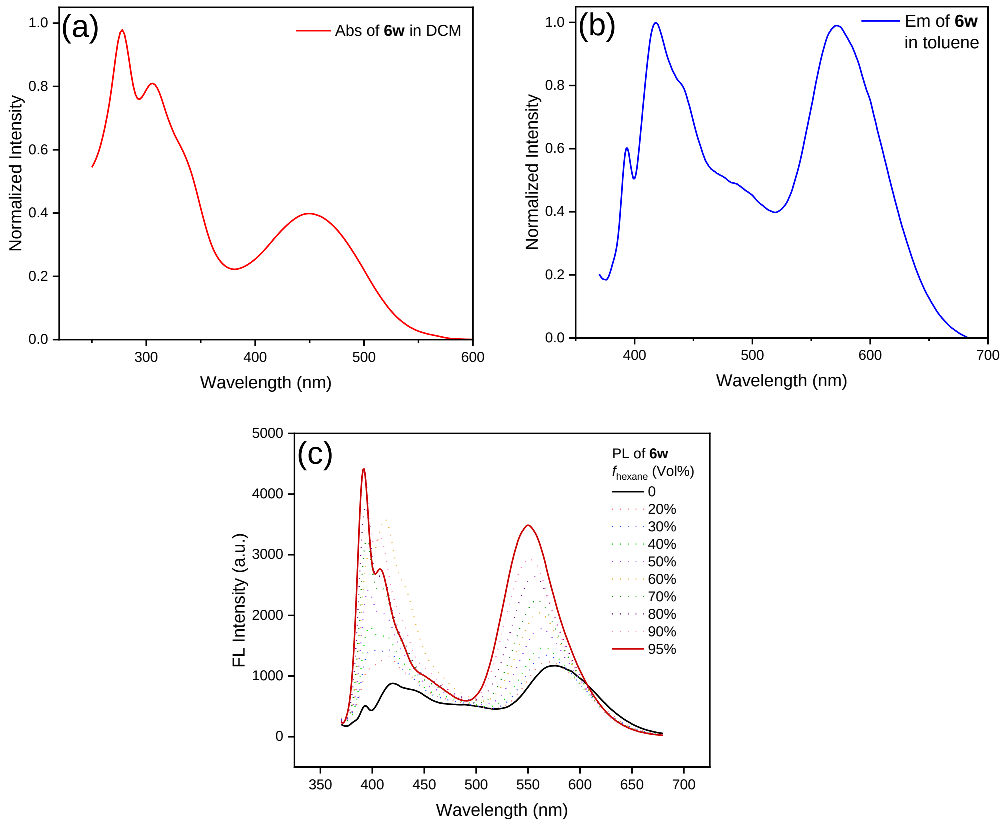

Fig. S13. (a) Normalized UV-Vis absorption spectra of **6w** in DCM (1×10^{-6} M); (b) Normalized fluorescence emission spectra of **6w** in toluene (2.4×10^{-6} M); (c) Fluorescence spectra of **6w** in toluene–hexane mixtures (2.4×10^{-6} M) with different fractions (f_{hexane}).

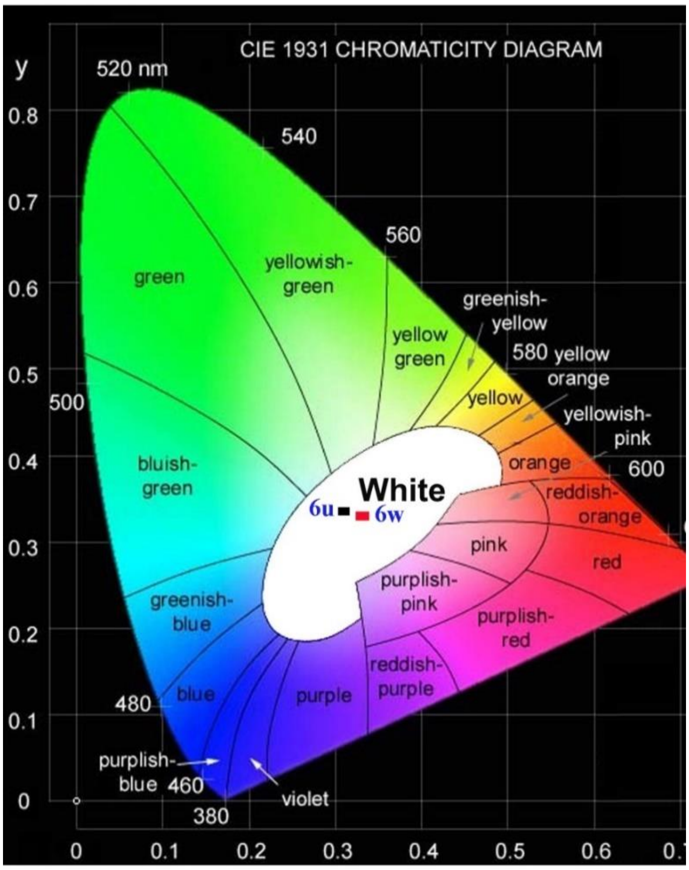

Fig. S14. The white-light emissions of **6u** and **6w**. CIE_{1931} chromaticity diagrams **6u** (0.31, 0.34) and **6w** (0.33, 0.33) in CH_2Cl_2 (Concentration: **6u**: 2.5×10^{-7} M; **6w**: 2.4×10^{-6} M).

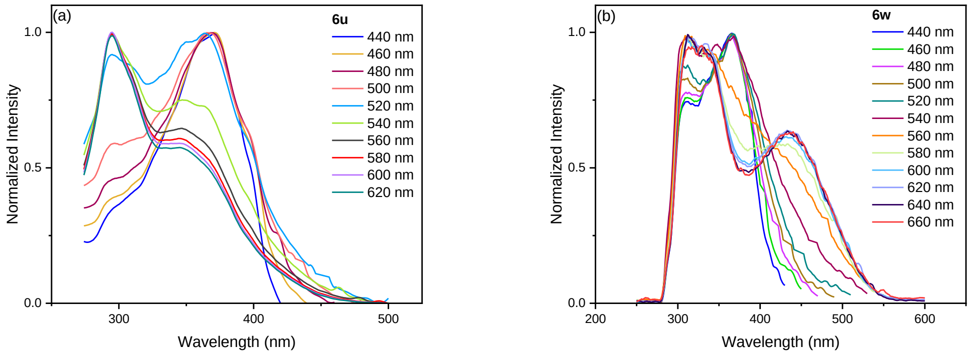

Fig. S15. (a) Emission-wavelength-dependent excitation spectra of **6u** (1.0×10^{-8} M in DCM). (b) Emission-wavelength-dependent excitation spectra of **6w** (1.3×10^{-7} M in toluene). Excitation spectra were collected by Edinburgh Instruments FLS1000.

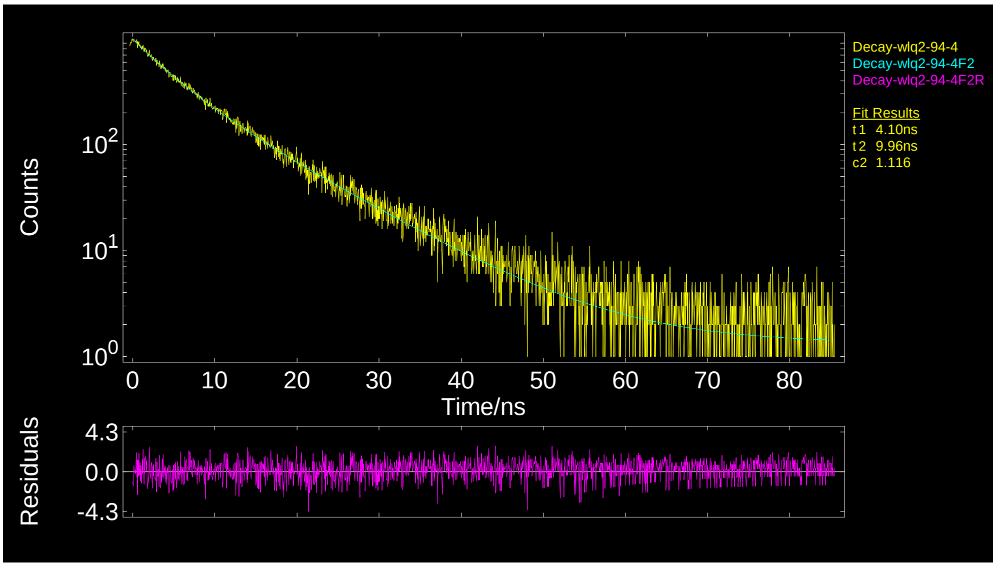

Fig. S16. Fluorescence lifetime decay curves of **6u** in TBCPF film (3 wt%).

	Fix	Value / ns	Std. Dev / ns		Fix	Value	Std. Dev	Rel %
τ_1	☐	4.1024	0.1554	B_1	☐	524.3182	21.0846	31.39
τ_2	☐	9.9586	0.1609	B_2	☐	472.1648	23.1554	68.61
τ_3	☐			B_3	☐			
τ_4	☐			B_4	☐			

	Value / ns	Std. Dev / ns
$\langle\tau\rangle_{amp}$	6.8773	0.1451
$\langle\tau\rangle_{int}$	8.1205	0.1533

A ☐ 1.3421

χ^2 : **1.1161**

Fig. S17. The excited state lifetimes of **6u** in TBCPF film.

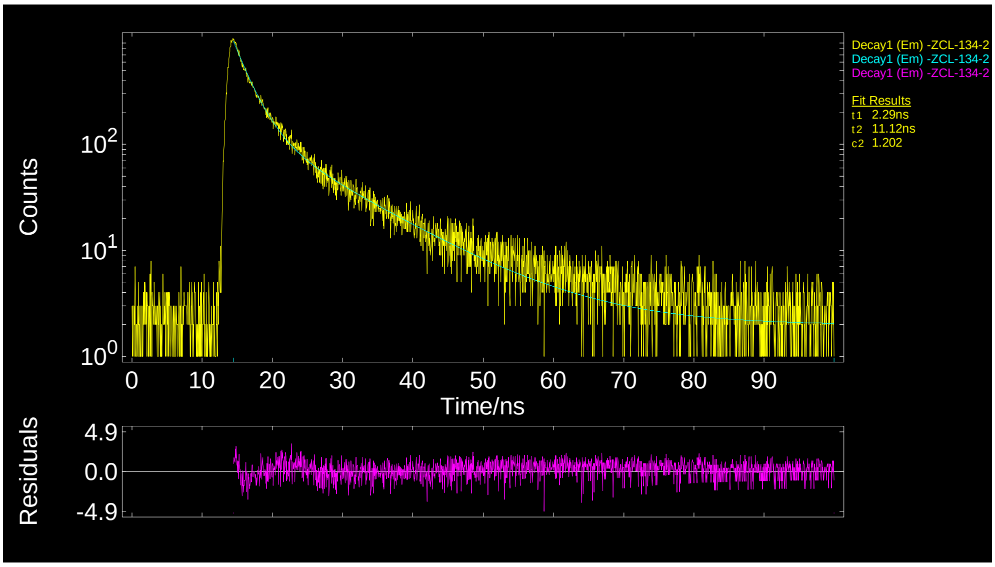

Fig. S18. Fluorescence lifetime decay curves of **6w** in TBCPF film (10 wt%).

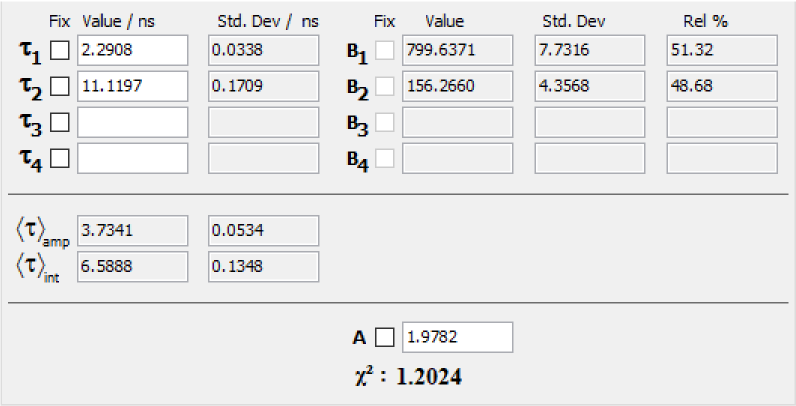

	Fix	Value / ns	Std. Dev / ns		Fix	Value	Std. Dev	Rel %
τ_1	☐	2.2908	0.0338	B_1	☐	799.6371	7.7316	51.32
τ_2	☐	11.1197	0.1709	B_2	☐	156.2660	4.3568	48.68
τ_3	☐			B_3	☐			
τ_4	☐			B_4	☐			
$\langle\tau\rangle_{amp}$		3.7341	0.0534					
$\langle\tau\rangle_{int}$		6.5888	0.1348					

A ☐ 1.9782

χ^2 : 1.2024

Fig. S19. The excited state lifetimes of **6w** in TBCPF film.

4.2. Preparation and characterization of 6w NPs.

The compound **6w** (2.0 mg) was dissolved in THF (2 mL) followed by ultrasound for 10 min. Poloxamer 188 (60 mg) was added to the solution of **6w** in THF and the mixture was treated by ultrasound for 10 min. The resulted mixture was stirred three hours at RT. Then the solvent was removed by reduced pressure. The residue was dissolved in deionized water (2 mL) and stirred overnight at RT to drive the formation of NPs. To remove the residual THF, the nanoparticle suspensions were dialyzed by three times. The concentrations of nanoparticles were calibrated via the UV-vis spectra. Finally, the NPs solution was collected and stored at 4 °C.

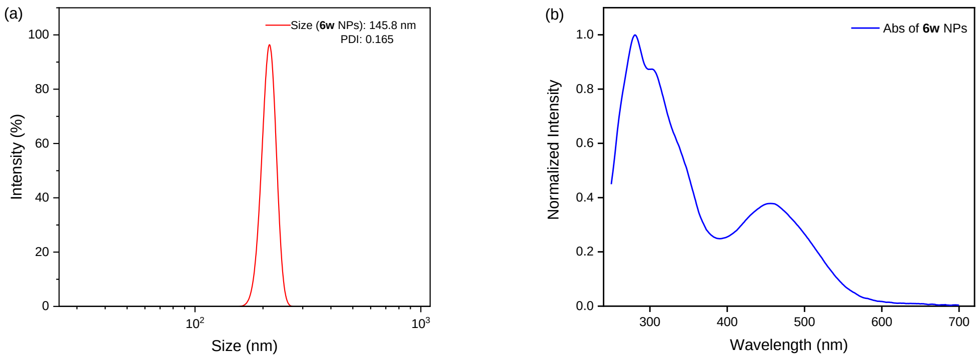

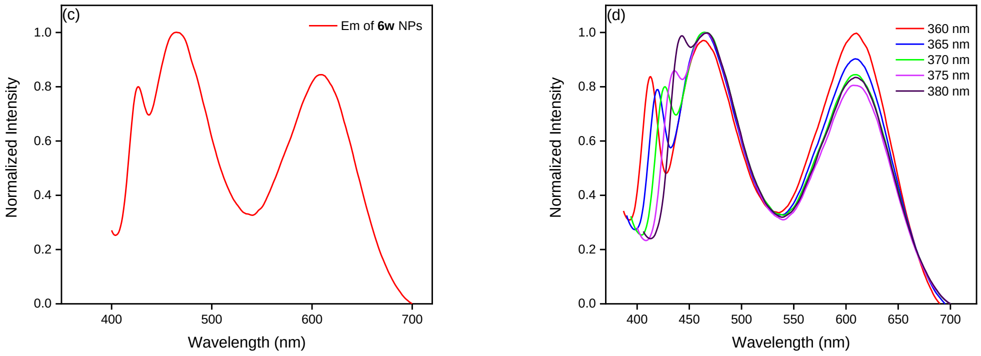

Fig. S20. (a) Hydrodynamic size distribution of **6w** NPs in aqueous solution. (b) Normalized UV-Vis absorption spectra emission spectra of **6w** NPs. in H_2O (1.0×10^{-6} M). (b) Normalized fluorescence emission spectra of **6w** NPs. in H_2O (2.0×10^{-5} M). (d) Excitation-wavelength-dependent fluorescence spectra of **6w** NPs in H_2O (2.0×10^{-5} M).

4.3. Cell imaging experiments

Cellular Experiments: The Hela cells were cultured in 10% FBS and 1% penicillin-streptomycin-containing DMEM medium at 37 ℃ with 5% CO_2.

Methyl thiazolyl tetrazolium (MTT) assay: To assess the safe usability of **6w** nanoparticles for biomedical applications, the cytotoxicity experiments in Hela cells were carried out using the standard methyl thiazolyl tetrazolium (MTT) assay.

Cell viability assay: The Hela cells were seeded into 96-well plates and incubated with a standard medium overnight. The cell suspensions were treated with different concentrations of **6w** NPs in a deionized water solution (0, 1, 5, 10, 20, 40 μM) and incubated for another 24 h. Live/dead cell staining was carried out after 20 min white light irradiation and without light irradiation, respectively.

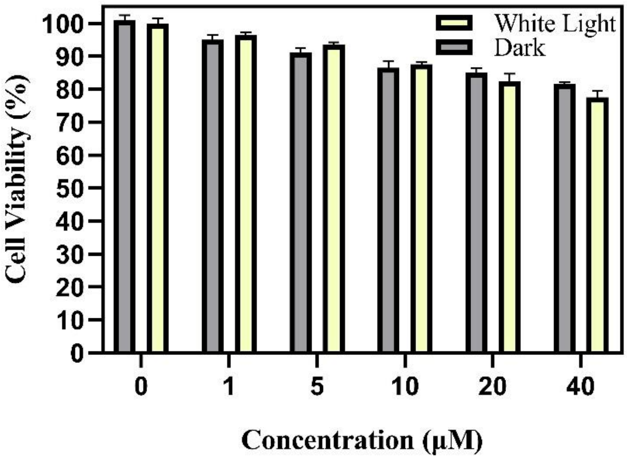

Fig. S21. In vitro cytotoxicity of the **6w** NPs. Cell viabilities of Hela cells incubated with **6w** NPs for 24 h.

Confocal imaging experiments: The confocal imaging experiments were performed on Leica TCS SP8. The nanoparticles of product **6w** (**6w** NPs) was used as fluorescent probe.

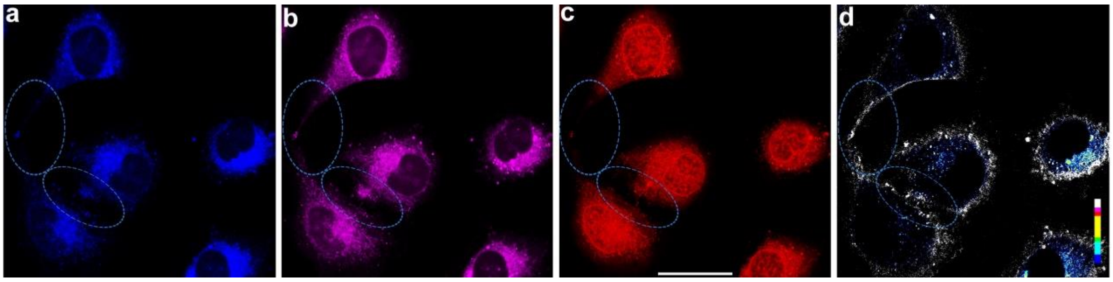

Fig. S22. Fluorescence microscopy images of HeLa cells incubated with **6w** NPs (10 μM) for 2 h at 37 ^{o}C. (a) Fluorescence microscope image from channel 1 at 425–500 nm (Excitation 405 nm). (b) Fluorescence microscope image from channel 1 at 500–700 nm (Excitation 405 nm). (c) Fluorescence microscope image from channel 2 at 500–700 nm (Excitation 488 nm). (d) The emission intensity ratio of (a) and (c) of HeLa cells (Image generation by Image J software). Elliptic: mark out the intercellular structure and cellular local imaging information. The scale bar is 25.0 μm.

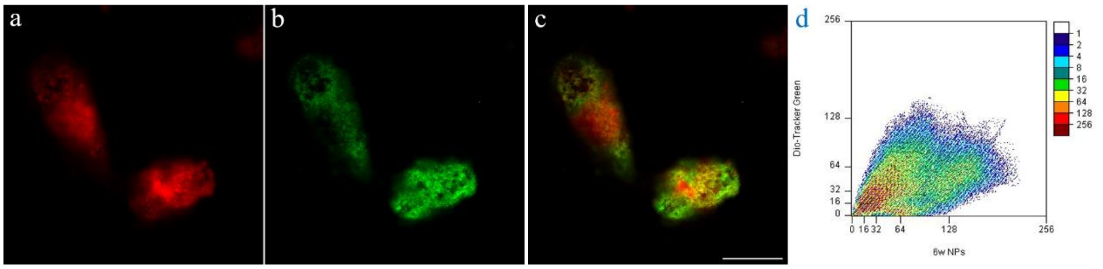

Fig. S23. The co-staining experiments. (a) Fluorescent image of HeLa cells cultured with **6w** NPs (10 μM) (λ_{ex} = 552 nm, λ_{em} = 575–750 nm). (b) Fluorescent image of HeLa cells with Dio-Tracker Green (λ_{ex} = 488 nm, λ_{em} = 508–540 nm). (c) Merged image of (a) and (b). (d) The Pearson correlation coefficient r = 0.57; The scale bar is 25.0 μm.

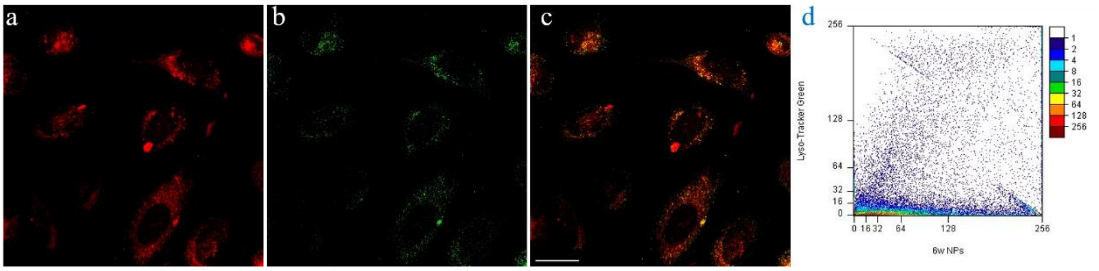

Fig. S24. The co-staining experiments. (a) Fluorescent image of HeLa cells cultured with **6w** NPs (10 μM) (λ_{ex} = 552 nm, λ_{em} = 575–750 nm). (b) Fluorescent image of HeLa cells with Lyso-Tracker Green (λ_{ex} = 488 nm, λ_{em} = 508–540 nm). (c) Merged image of (a) and (b). (d) The Pearson correlation coefficient r = 0.44; The scale bar is 25.0 μm.

5. References

[S1] Frisch, M. J., Trucks, G. W., Schlegel, H. B., Scuseria, G. E., Robb, M. A., Cheeseman, J. R., Scalmani, G., Barone, V., Petersson, G. A., Nakatsuji, H., Li, X., Caricato, M., Marenich, A. V., Bloino, J., Janesko, B. G., Gomperts, R., Mennucci, B., Hratchian, H. P., Ortiz, J. V., Izmaylov, A. F., Sonnenberg, J. L., Williams-Young, D., Ding, F., Lipparini, F., Egidi, F., Goings, J., Peng, B., Petrone, A., Henderson, T., Ranasinghe, D., Zakrzewski, V. G., Gao, J., Rega, N., Zheng, G., Liang, W., Hada, M., Ehara, M., Toyota, K., Fukuda, R., Hasegawa, J., Ishida, M., Nakajima, T., Honda, Y., Kitao, O., Nakai, H., Vreven, T. K. Throssell,

K., Montgomery, J. A., Peralta, Jr., J. E., Ogliaro, F., Bearpark, M. J., Heyd, J. J., Brothers, E. N., Kudin, K. N., Staroverov, V. N., Keith, T. A., Kobayashi, R., Normand, J., Raghavachari, K., Rendell, A. P., Burant, J. C., Iyengar, S. S., Tomasi, J., Cossi, M., Millam, J. M., Klene, M., Adamo, C., Cammi, R., Ochterski, J. W., Martin, R. L., Morokuma, K., Farkas, O., Foresman, J. B. & Fox, D. J., Gaussian 16, Revision A.03, Gaussian, Inc., Wallingford CT, **2016**.

[S2] J. Bai, N. Xu, H. Wang and X. Luan. *Org. Lett.* **2022**, *24*, 5099–5104.

[S3] J. Wang, Z. Dong, C. Yang and G. Dong. *Nat. Chem.* **2019**, *11*, 1106–1112.

[S4] V. Sukowski, M. Borselen, S. Mathew and M. Á. Fernández-Ibáñez. *Angew. Chem. Int. Ed.* **2022**, *61*, e202201750.

6. NMR spectrum of compounds

NMR spectrum of products 4a – 6w:

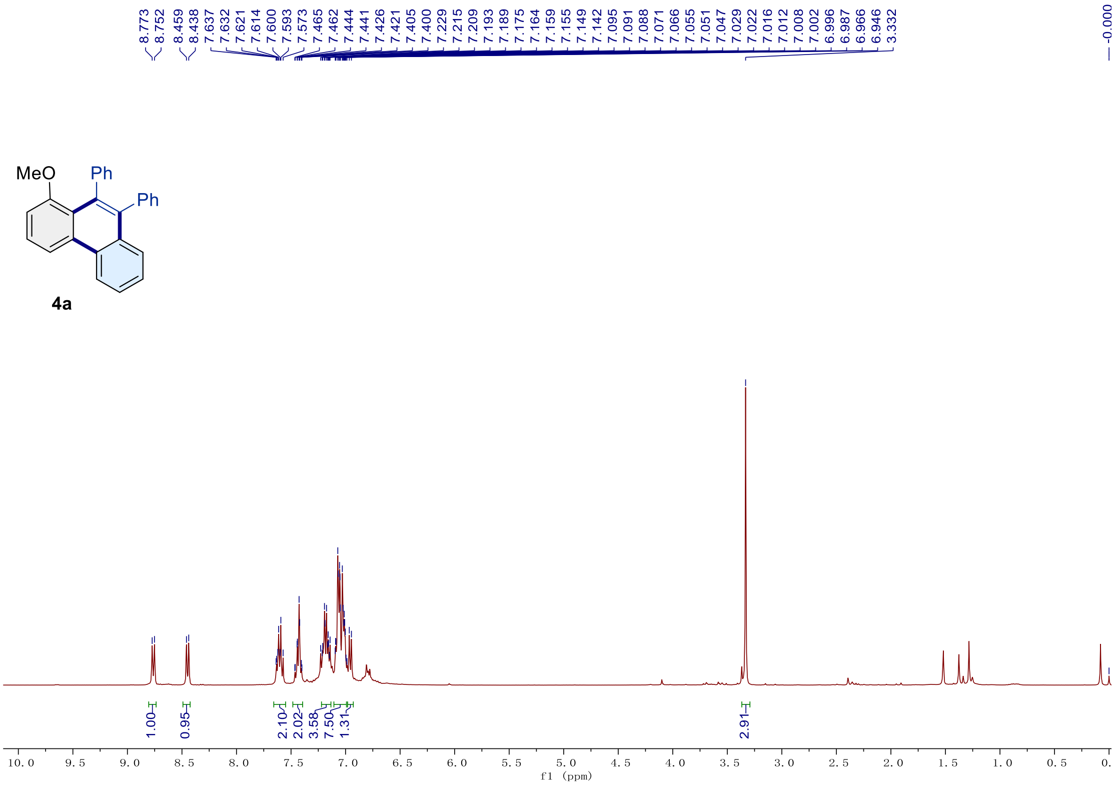

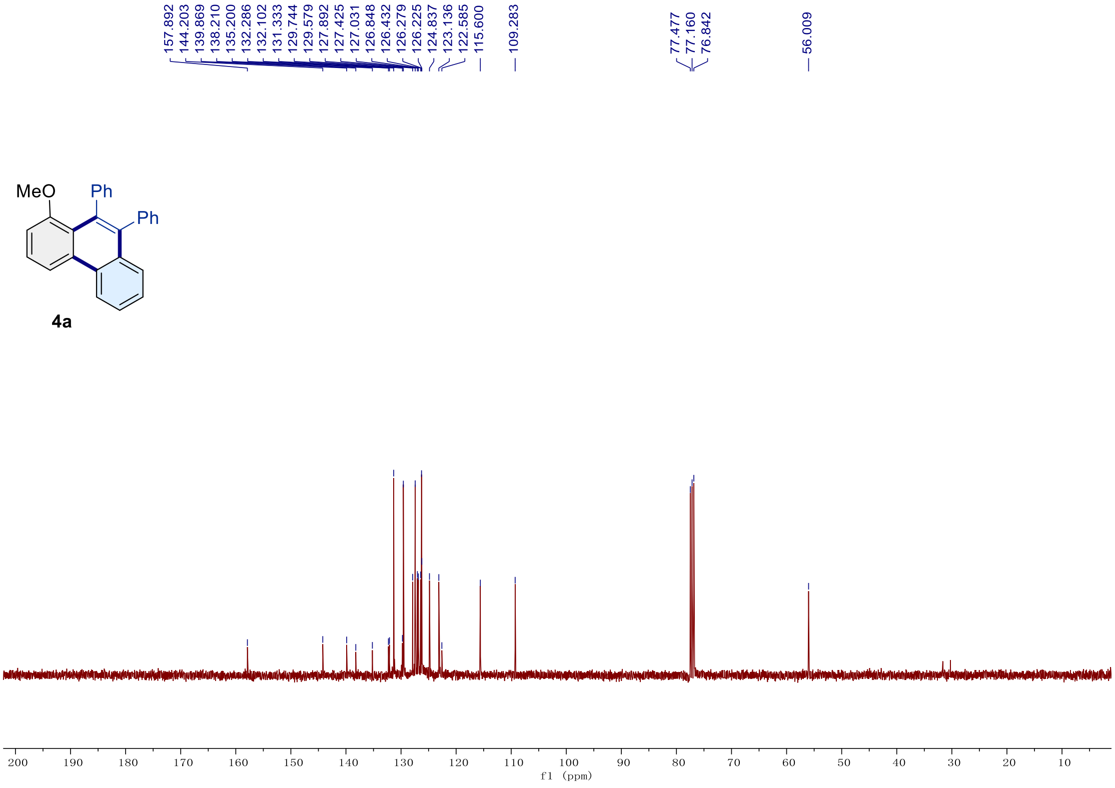

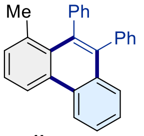
Me
Ph
Ph

4b

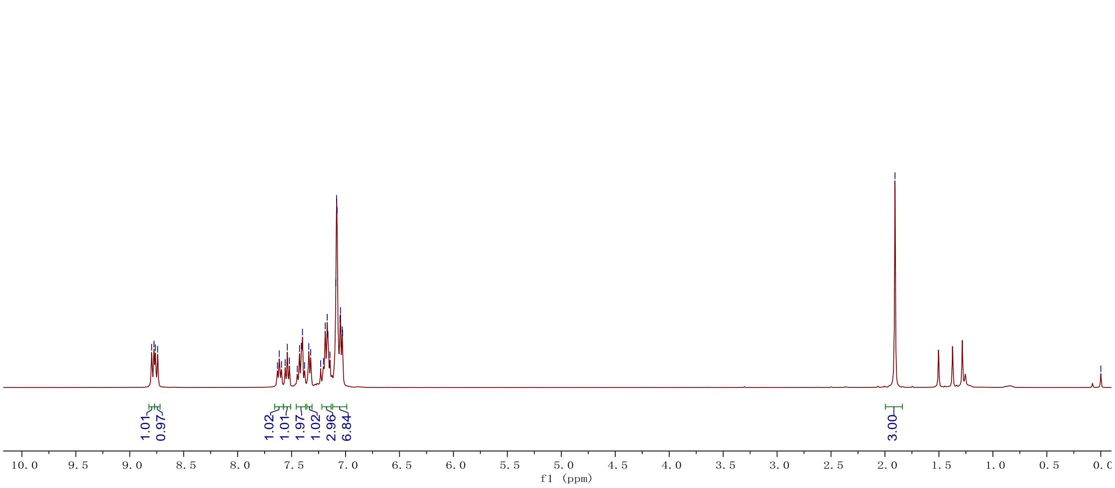
8.797
8.775
8.763
8.741
7.631
7.614
7.594
7.559
7.540
7.521
7.447
7.427
7.410
7.400
7.380
7.340
7.323
7.231
7.205
7.188
7.170
7.163
7.144
7.090
7.084
7.079
7.051
7.047
7.032
7.028
1.907
-0.001
1.01
0.97
1.02
1.01
1.97
1.02
2.96
6.84
3.00
10.0 9.5 9.0 8.5 8.0 7.5 7.0 6.5 6.0 5.5 5.0 4.5 4.0 3.5 3.0 2.5 2.0 1.5 1.0 0.5 0.0
f1 (ppm)

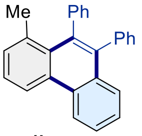
Me
Ph
Ph

4b

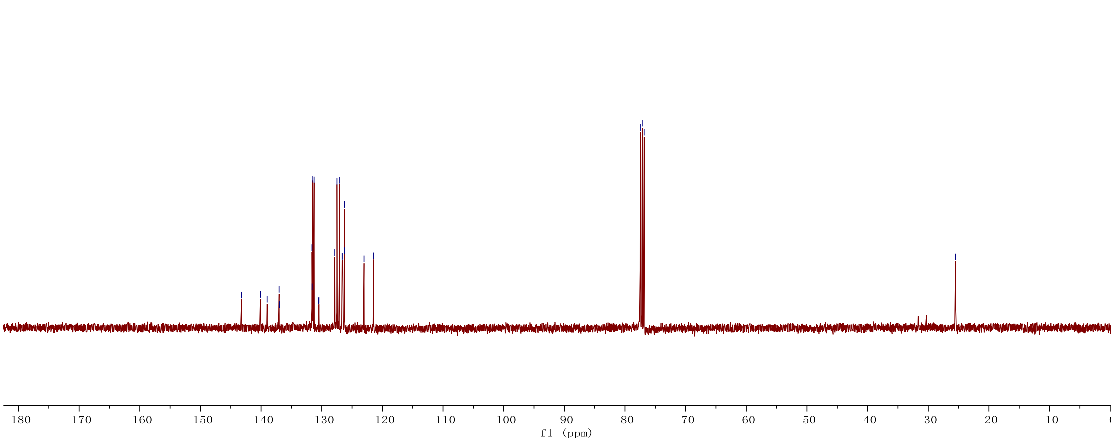
143.222
140.120
139.005
137.030
136.957
131.607
131.586
131.463
131.261
130.551
130.461
127.847
127.486
127.106
126.634
126.523
126.253
126.222
123.024
121.433
77.478
77.160
76.841
25.507
180 170 160 150 140 130 120 110 100 90 80 70 60 50 40 30 20 10
f1 (ppm)

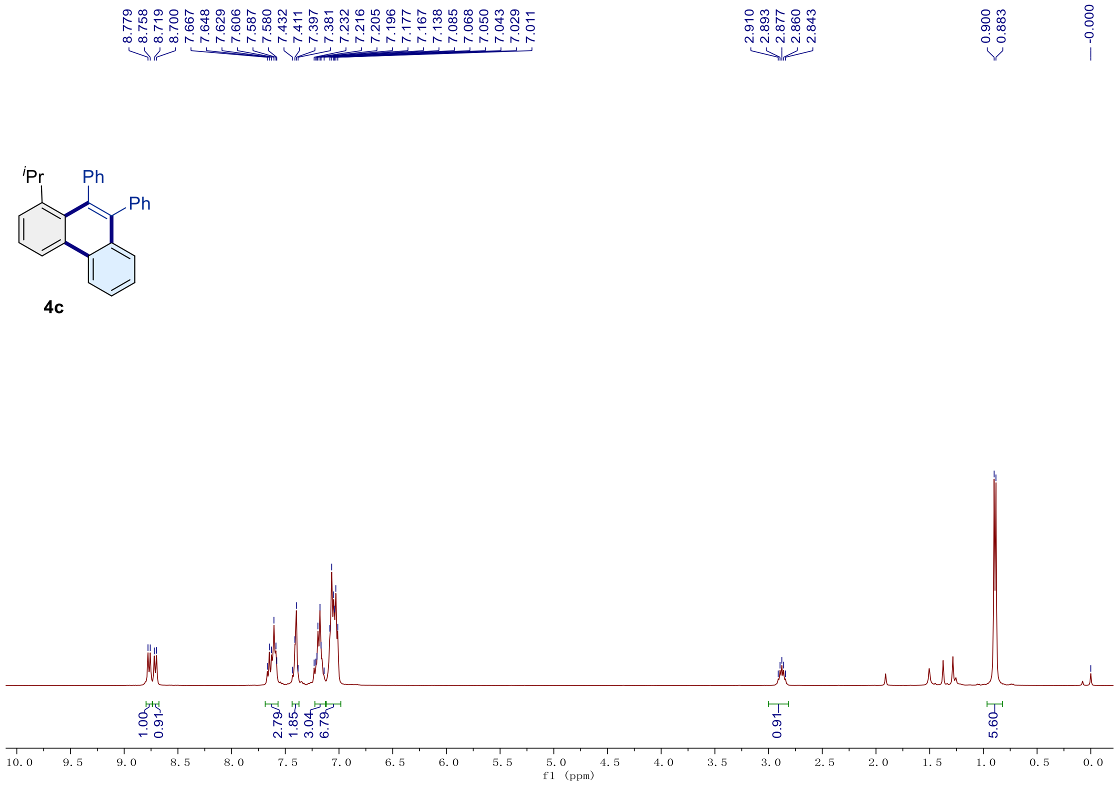
8.779
8.758
8.719
8.700
7.667
7.648
7.629
7.606
7.587
7.580
7.432
7.411
7.397
7.381
7.232
7.216
7.205
7.196
7.177
7.167
7.138
7.085
7.068
7.050
7.043
7.029
7.011
2.910
2.893
2.877
2.860
2.843
0.900
0.883
-0.000
iPr
Ph
Ph
4c
1.00
0.91
2.79
1.85
3.04
6.79
0.91
5.60
f1 (ppm)

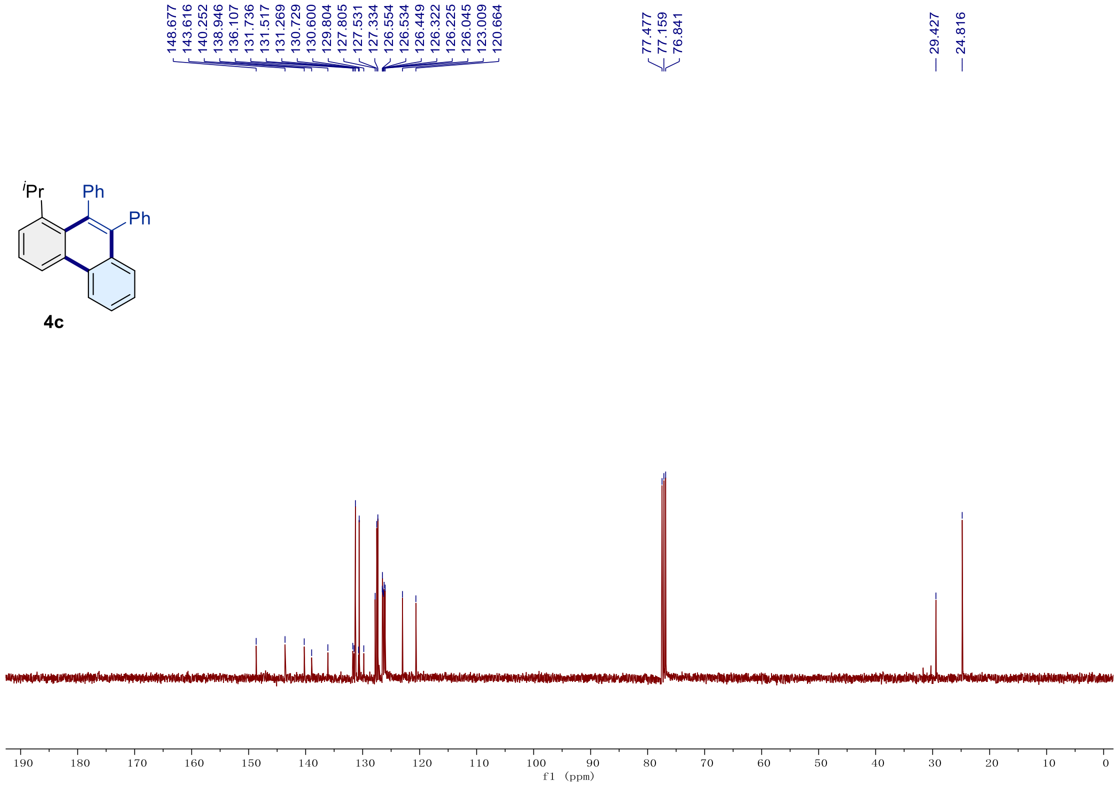
148.677
143.616
140.252
138.946
136.107
131.736
131.517
131.269
130.729
130.600
129.804
127.805
127.531
127.334
126.554
126.534
126.449
126.322
126.225
126.045
123.009
120.664
77.477
77.159
76.841
29.427
24.816
iPr
Ph
Ph
4c
f1 (ppm)

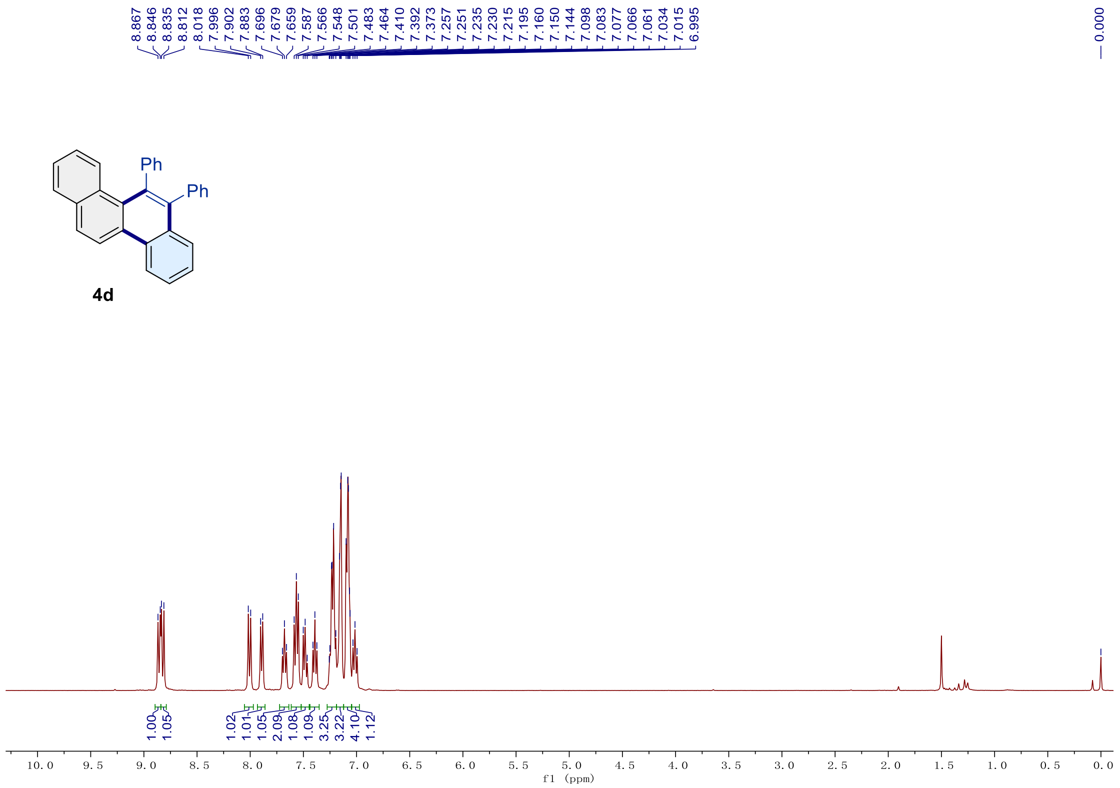
8.867
8.846
8.835
8.812
8.018
7.996
7.902
7.883
7.696
7.679
7.659
7.587
7.566
7.548
7.501
7.483
7.464
7.410
7.392
7.373
7.257
7.251
7.235
7.230
7.215
7.195
7.160
7.150
7.144
7.098
7.083
7.077
7.066
7.061
7.034
7.015
6.995
0.000
Ph
Ph
4d
1.00
1.05
1.02
1.01
1.05
2.09
1.08
1.09
3.25
3.22
4.10
1.12
10.0 9.5 9.0 8.5 8.0 7.5 7.0 6.5 6.0 5.5 5.0 4.5 4.0 3.5 3.0 2.5 2.0 1.5 1.0 0.5 0.0
f1 (ppm)

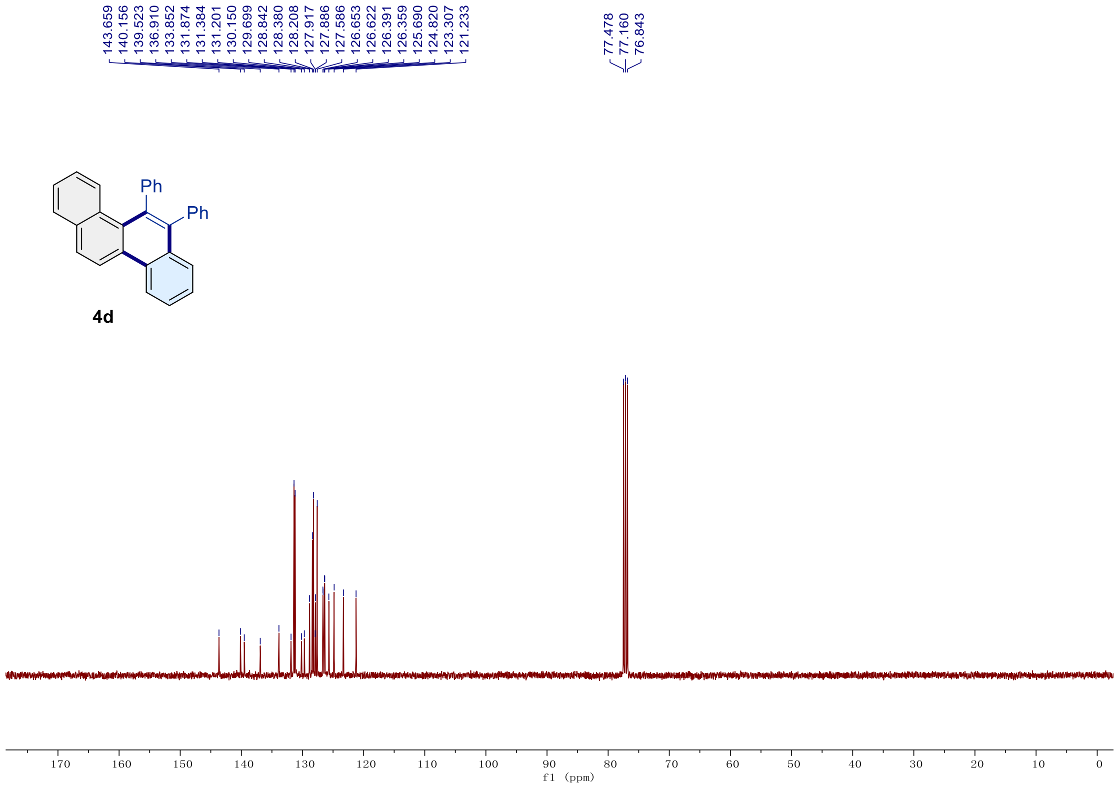
143.659
140.156
139.523
136.910
133.852
131.874
131.384
131.201
130.150
129.699
128.842
128.380
128.208
127.917
127.886
127.586
126.653
126.622
126.391
126.359
125.690
124.820
123.307
121.233
77.478
77.160
76.843
Ph
Ph
4d
170 160 150 140 130 120 110 100 90 80 70 60 50 40 30 20 10 0
f1 (ppm)

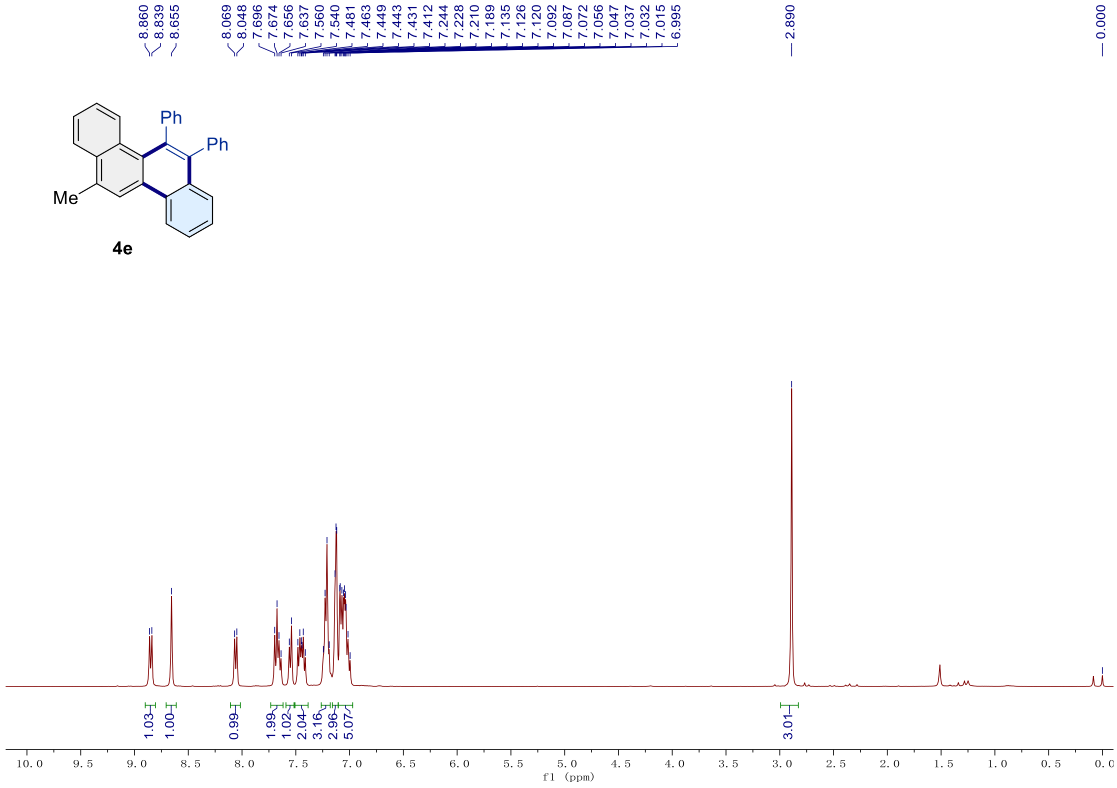

8.860
8.839
8.655
8.069
8.048
7.696
7.674
7.656
7.637
7.560
7.540
7.481
7.463
7.449
7.443
7.431
7.412
7.244
7.228
7.210
7.189
7.135
7.126
7.120
7.092
7.087
7.072
7.056
7.047
7.037
7.032
7.015
6.995
2.890
0.000
Ph
Ph
Me
4e
1.03
1.00
0.99
1.99
1.02
2.04
3.16
2.96
5.07
3.01
f1 (ppm)

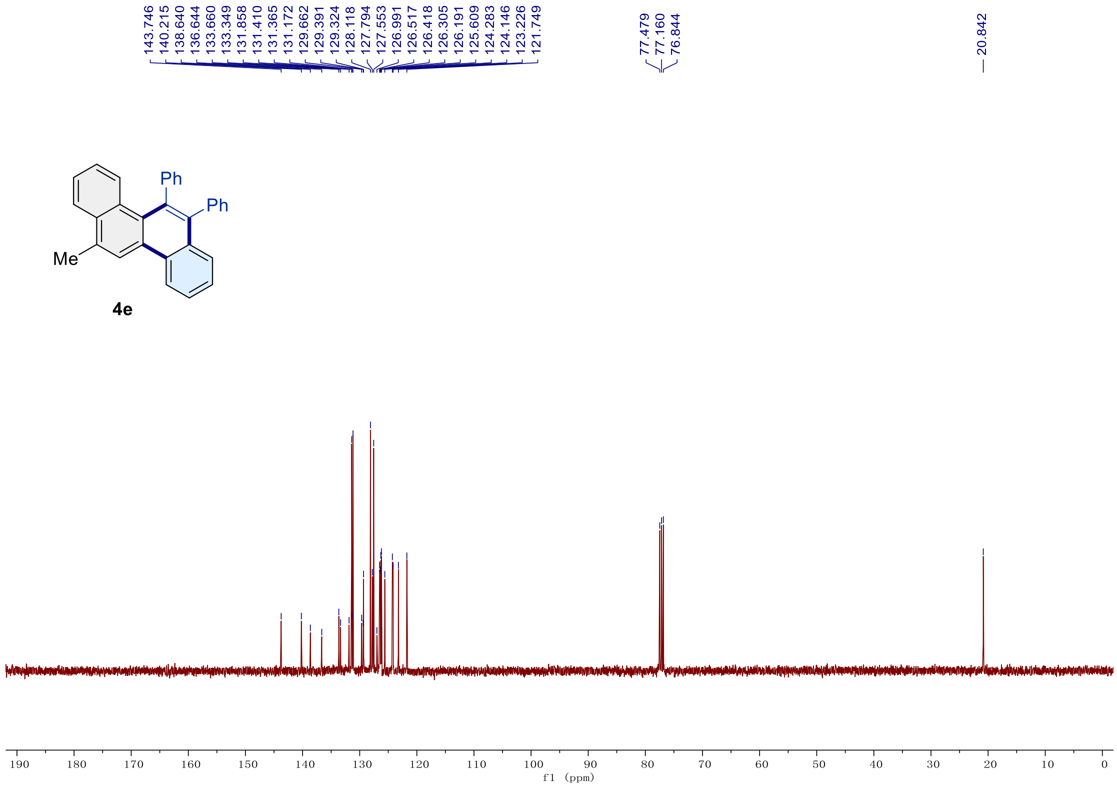

143.746
140.215
138.640
136.644
133.660
133.349
131.858
131.410
131.365
131.172
129.662
129.391
129.324
128.118
127.794
127.553
126.991
126.517
126.418
126.305
126.191
125.609
124.283
124.146
123.226
121.749
77.479
77.160
76.844
20.842
Ph
Ph
Me
4e
f1 (ppm)

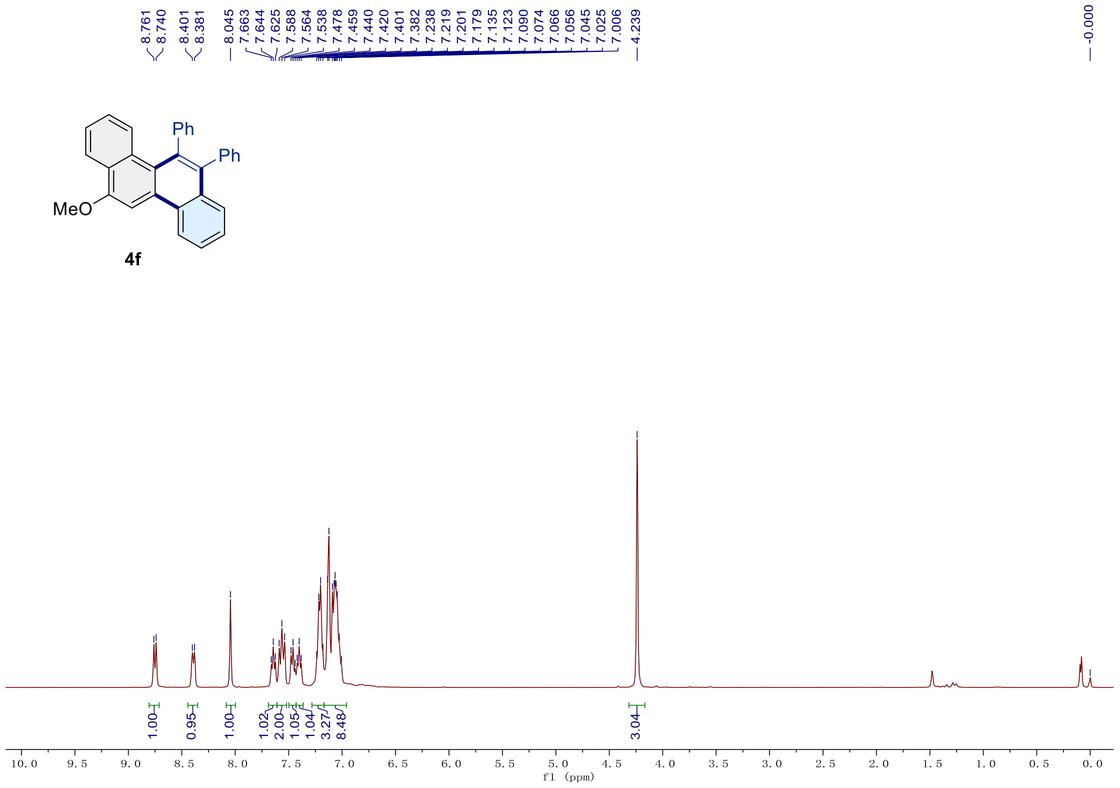
8.761
8.740
8.401
8.381
8.045
7.663
7.644
7.625
7.588
7.564
7.538
7.478
7.459
7.440
7.420
7.401
7.382
7.238
7.219
7.201
7.179
7.135
7.123
7.090
7.074
7.066
7.056
7.045
7.025
7.006
4.239
-0.000
Ph
Ph
MeO
4f
1.00
0.95
1.00
1.02
2.00
1.05
1.04
3.27
8.48
3.04
f1 (ppm)

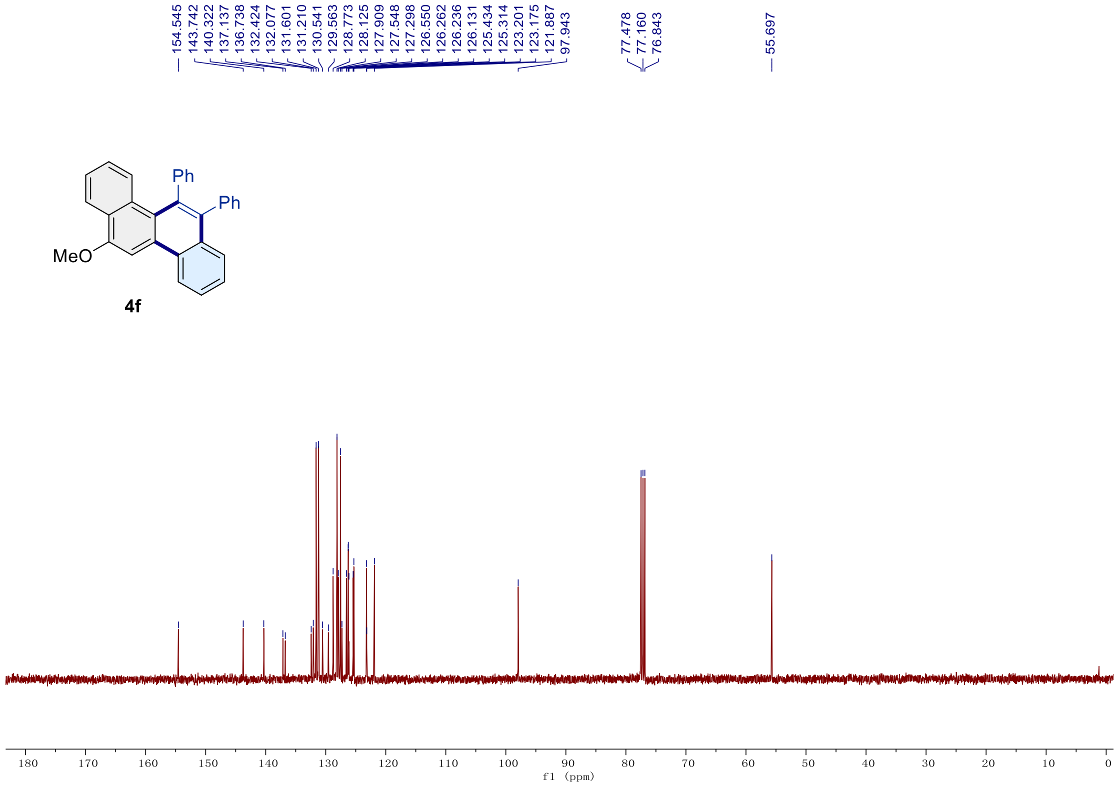
154.545
143.742
140.322
137.137
136.738
132.424
132.077
131.601
131.210
130.541
129.563
128.773
128.125
127.909
127.548
127.298
126.550
126.262
126.236
126.131
125.434
125.314
123.201
123.175
121.887
97.943
77.478
77.160
76.843
55.697
Ph
Ph
MeO
4f
f1 (ppm)

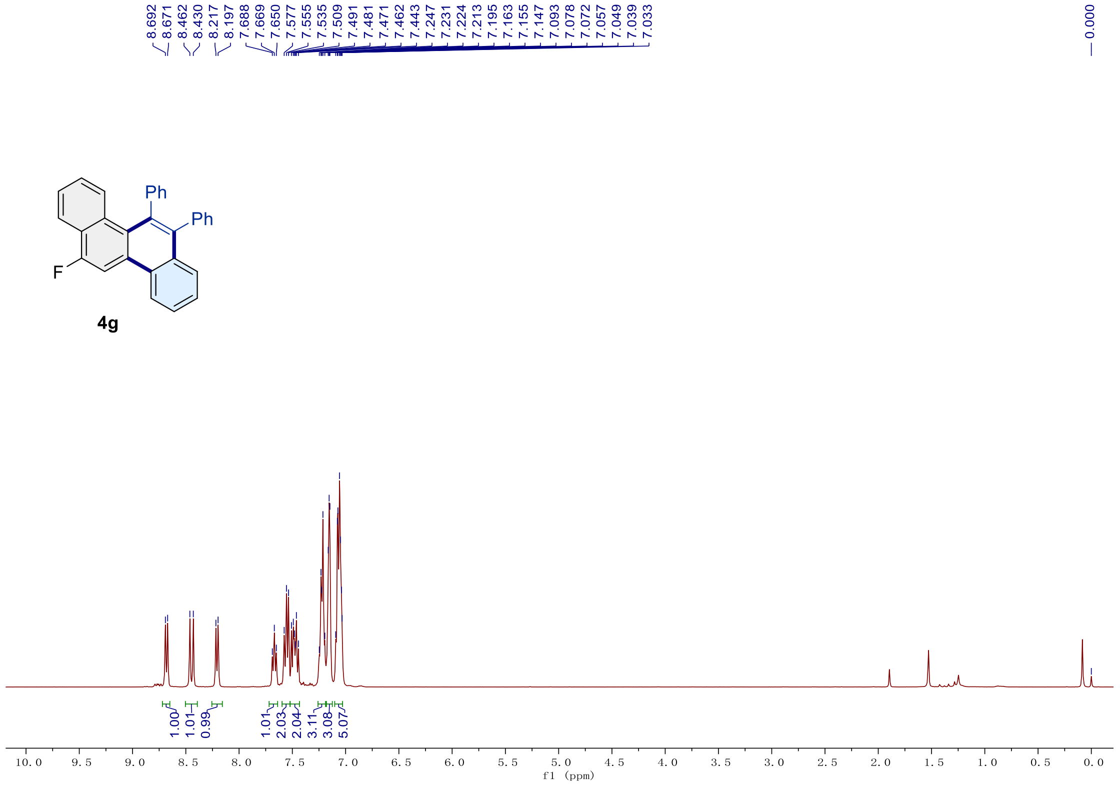
8.692
8.671
8.462
8.430
8.217
8.197
7.688
7.669
7.650
7.577
7.555
7.535
7.509
7.491
7.481
7.471
7.462
7.443
7.247
7.231
7.224
7.213
7.195
7.163
7.155
7.147
7.093
7.078
7.072
7.057
7.049
7.039
7.033
0.000
Ph
Ph
F
4g
1.00
1.01
0.99
1.01
2.03
2.04
3.11
3.08
5.07
f1 (ppm)

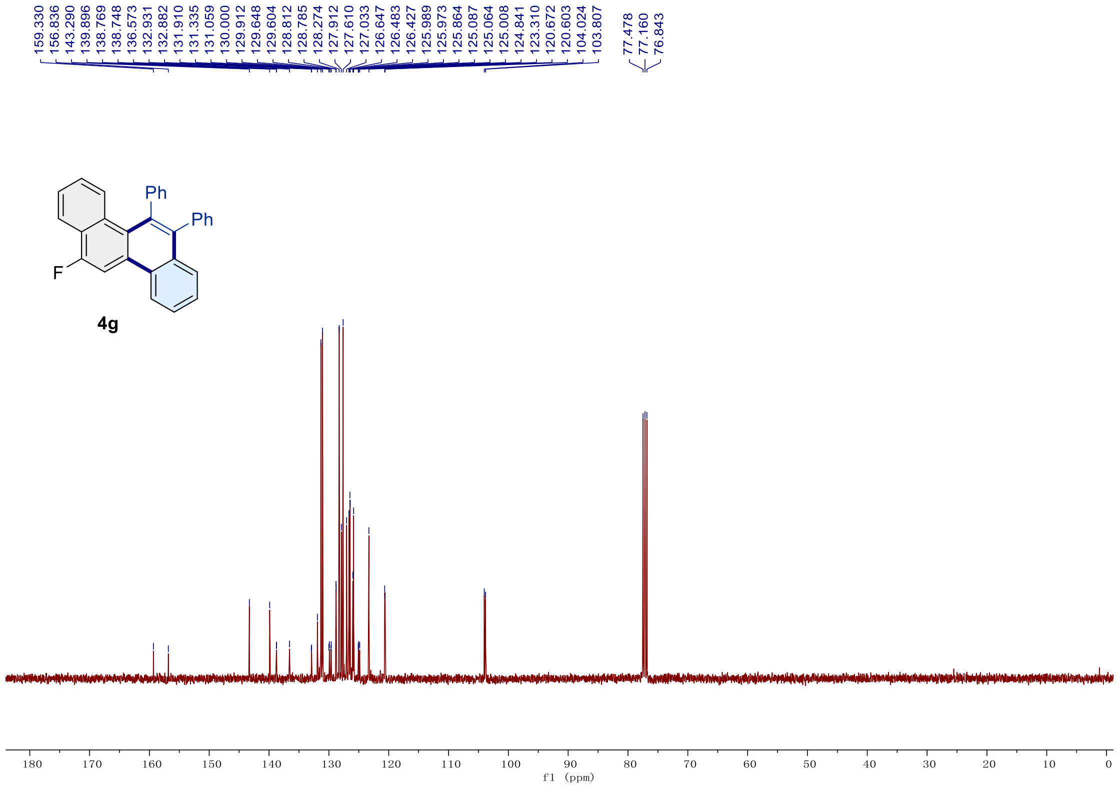
159.330
156.836
143.290
139.896
138.769
138.748
136.573
132.931
132.882
131.910
131.335
131.059
130.000
129.912
129.648
129.604
128.812
128.785
128.274
127.912
127.610
127.033
126.647
126.483
126.427
125.989
125.973
125.864
125.087
125.064
125.008
124.841
123.310
120.672
120.603
104.024
103.807
77.478
77.160
76.843
Ph
Ph
F
4g
f1 (ppm)

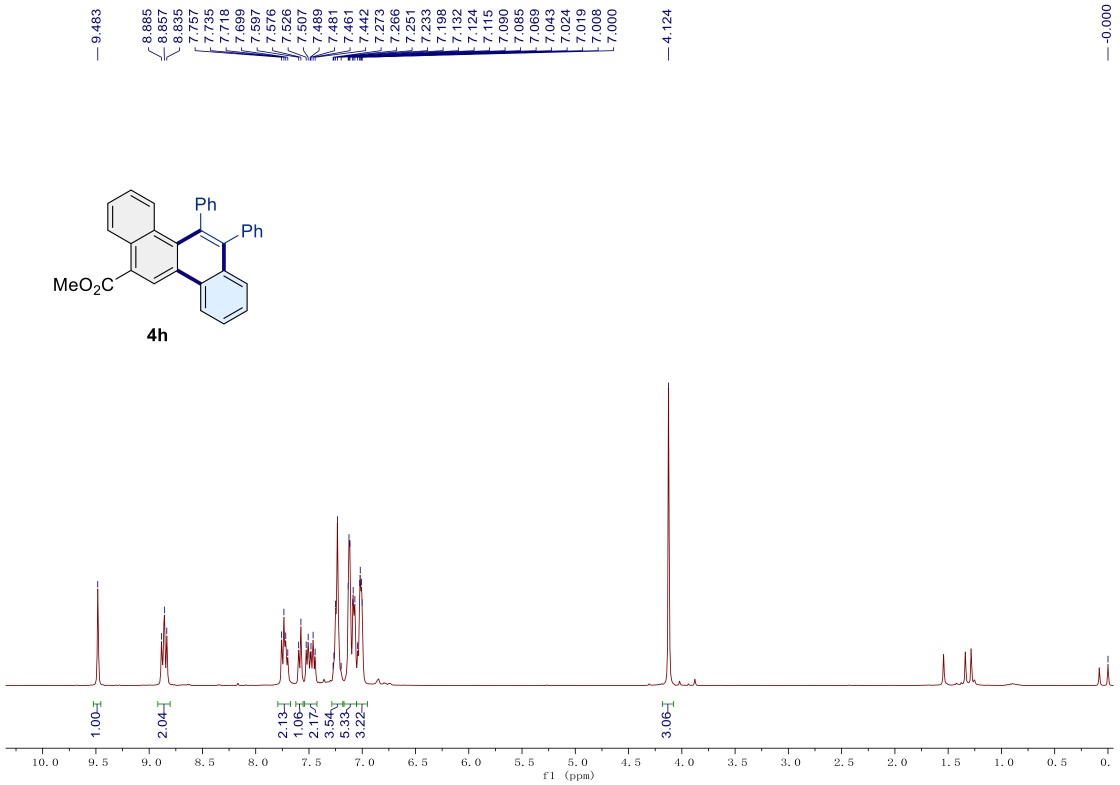
9.483
8.885
8.857
8.835
7.757
7.735
7.718
7.699
7.597
7.576
7.526
7.507
7.489
7.481
7.461
7.442
7.273
7.266
7.251
7.233
7.198
7.132
7.124
7.115
7.090
7.085
7.069
7.043
7.024
7.019
7.008
7.000
4.124
-0.000
Ph
Ph
MeO2C
4h
1.00
2.04
2.13
1.06
2.17
3.54
5.33
3.22
3.06
10.0 9.5 9.0 8.5 8.0 7.5 7.0 6.5 6.0 5.5 5.0 4.5 4.0 3.5 3.0 2.5 2.0 1.5 1.0 0.5 0.
f1 (ppm)

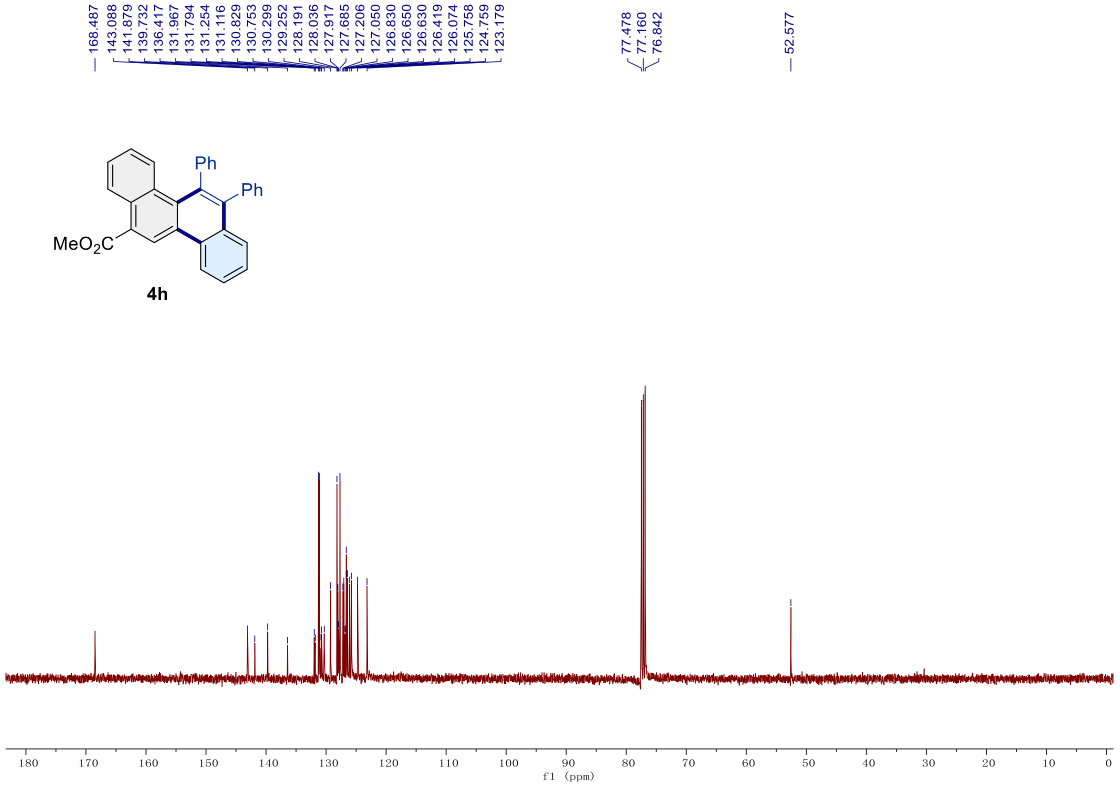
168.487
143.088
141.879
139.732
136.417
131.967
131.794
131.254
131.116
130.829
130.753
130.299
129.252
128.191
128.036
127.917
127.685
127.206
127.050
126.830
126.650
126.630
126.419
126.074
125.758
124.759
123.179
77.478
77.160
76.842
52.577
Ph
Ph
MeO2C
4h
180 170 160 150 140 130 120 110 100 90 80 70 60 50 40 30 20 10 0
f1 (ppm)

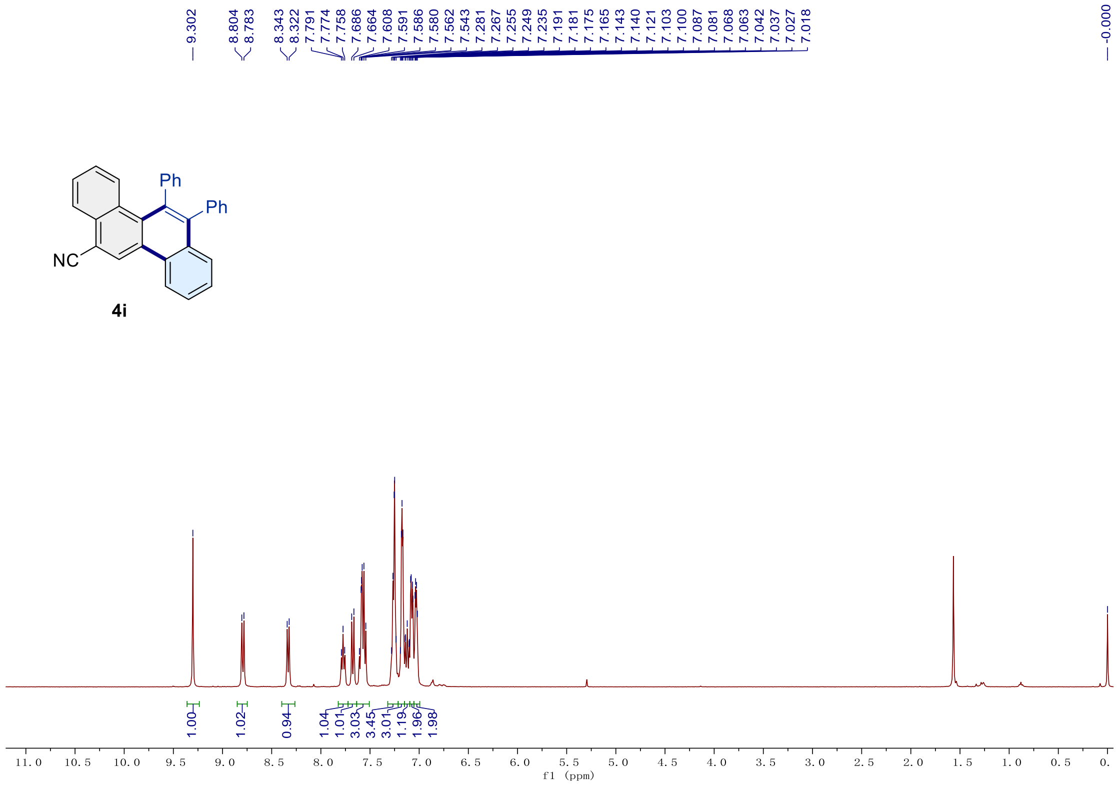
9.302
8.804
8.783
8.343
8.322
7.791
7.774
7.758
7.686
7.664
7.608
7.591
7.586
7.580
7.562
7.543
7.281
7.267
7.255
7.249
7.235
7.191
7.181
7.175
7.165
7.143
7.140
7.121
7.103
7.100
7.087
7.081
7.068
7.063
7.042
7.037
7.027
7.018
-0.000
Ph
Ph
NC
4i
1.00
1.02
0.94
1.04
1.01
3.03
3.45
3.01
1.19
1.96
1.98
f1 (ppm)

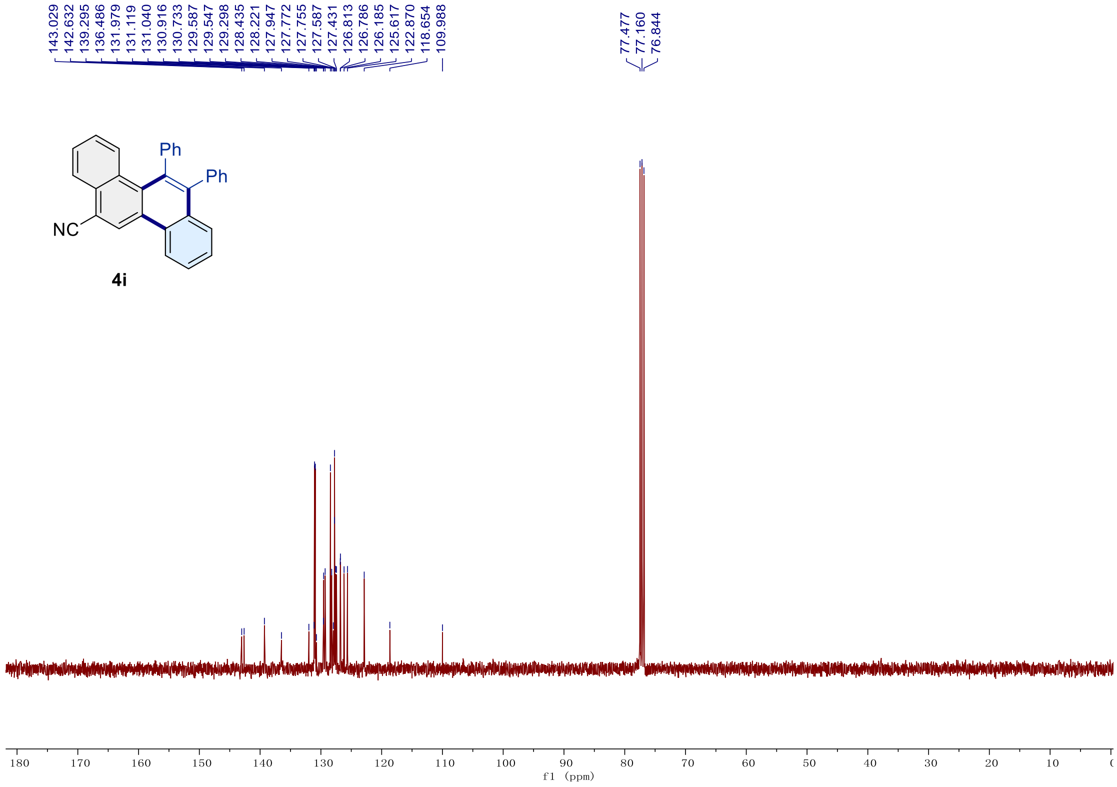
143.029
142.632
139.295
136.486
131.979
131.119
131.040
130.916
130.733
129.587
129.547
129.298
128.435
128.221
127.947
127.772
127.755
127.587
127.431
126.813
126.786
126.185
125.617
122.870
118.654
109.988
77.477
77.160
76.844
Ph
Ph
NC
4i
f1 (ppm)

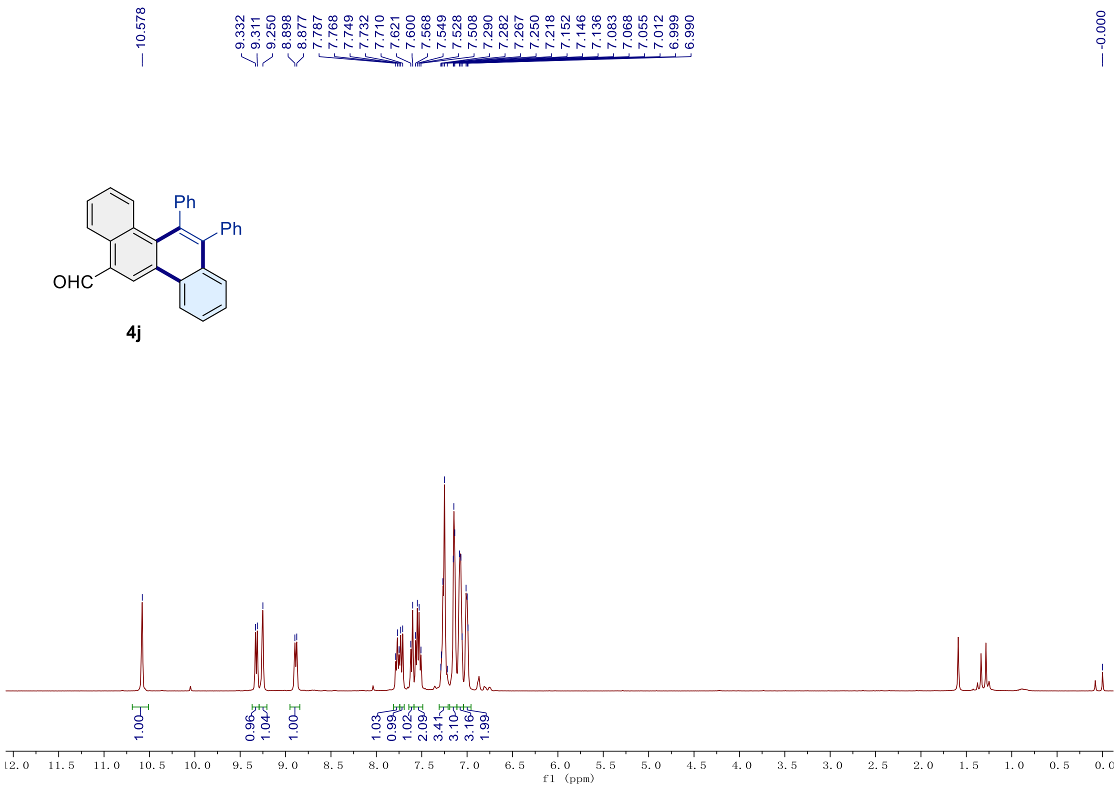
10.578
9.332
9.311
9.250
8.898
8.877
7.787
7.768
7.749
7.732
7.710
7.621
7.600
7.568
7.549
7.528
7.508
7.290
7.282
7.267
7.250
7.218
7.152
7.146
7.136
7.083
7.068
7.055
7.012
6.999
6.990
-0.000
Ph
Ph
OHC
4j
1.00
0.96
1.04
1.00
1.03
0.99
1.02
2.09
3.41
3.10
3.16
1.99
f1 (ppm)

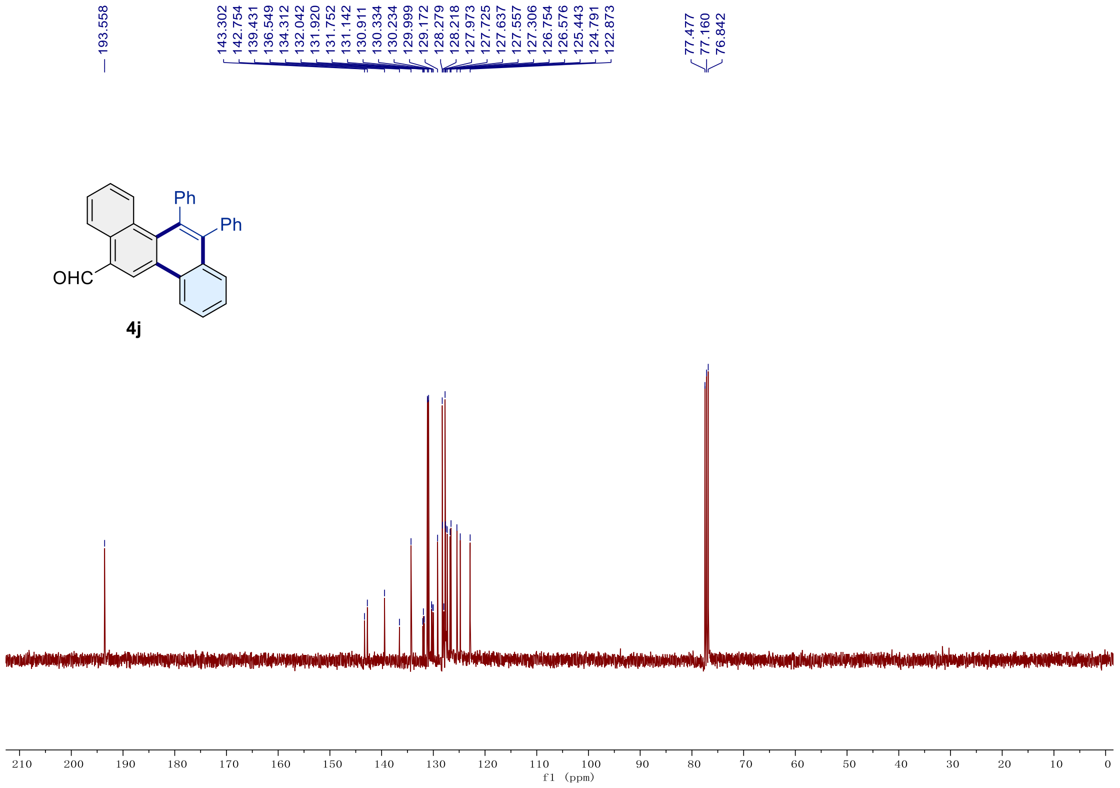
193.558
143.302
142.754
139.431
136.549
134.312
132.042
131.920
131.752
131.142
130.911
130.334
130.234
129.999
129.172
128.279
128.218
127.973
127.725
127.637
127.557
127.306
126.754
126.576
125.443
124.791
122.873
77.477
77.160
76.842
Ph
Ph
OHC
4j
f1 (ppm)

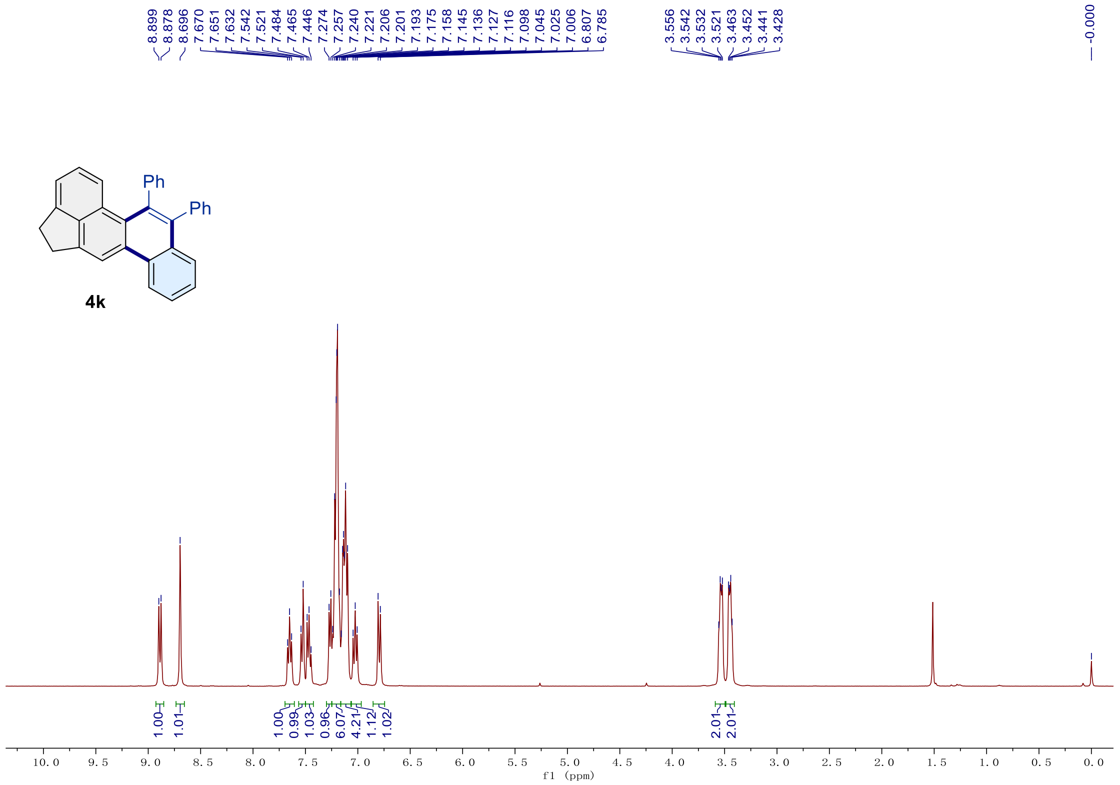
8.899
8.878
8.696
7.670
7.651
7.632
7.542
7.521
7.484
7.465
7.446
7.274
7.257
7.240
7.221
7.206
7.201
7.193
7.175
7.158
7.145
7.136
7.127
7.116
7.098
7.045
7.025
7.006
6.807
6.785
3.556
3.542
3.532
3.521
3.463
3.452
3.441
3.428
-0.000
Ph
Ph
4k
1.00
1.01
1.00
0.99
1.03
0.96
6.07
4.21
1.12
1.02
2.01
2.01
f1 (ppm)

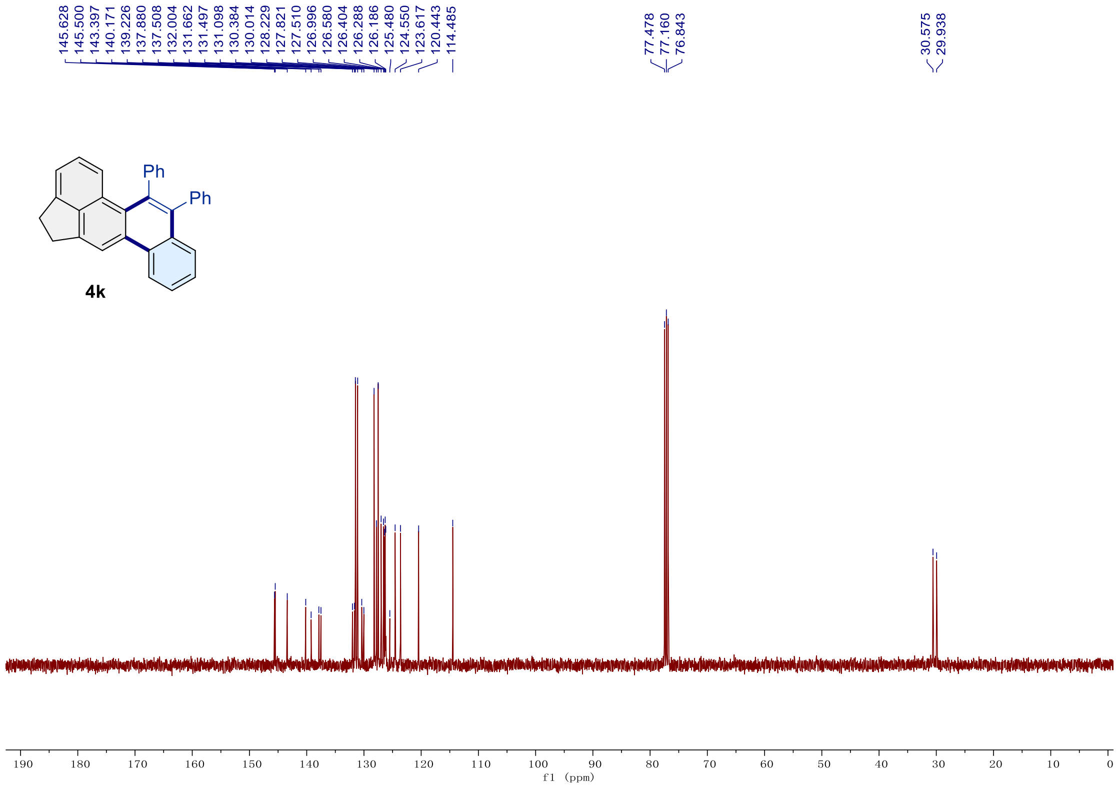
145.628
145.500
143.397
140.171
139.226
137.880
137.508
132.004
131.662
131.497
131.098
130.384
130.014
128.229
127.821
127.510
126.996
126.580
126.404
126.288
126.186
125.480
124.550
123.617
120.443
114.485
77.478
77.160
76.843
30.575
29.938
Ph
Ph
4k
f1 (ppm)

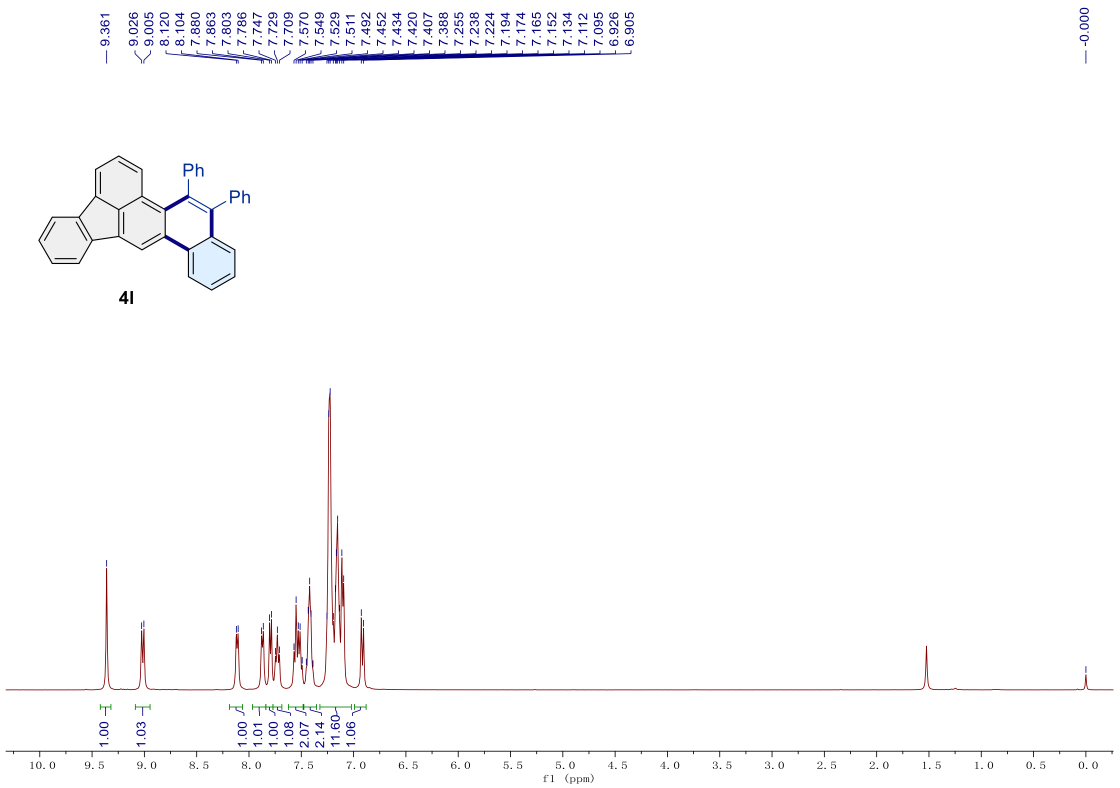

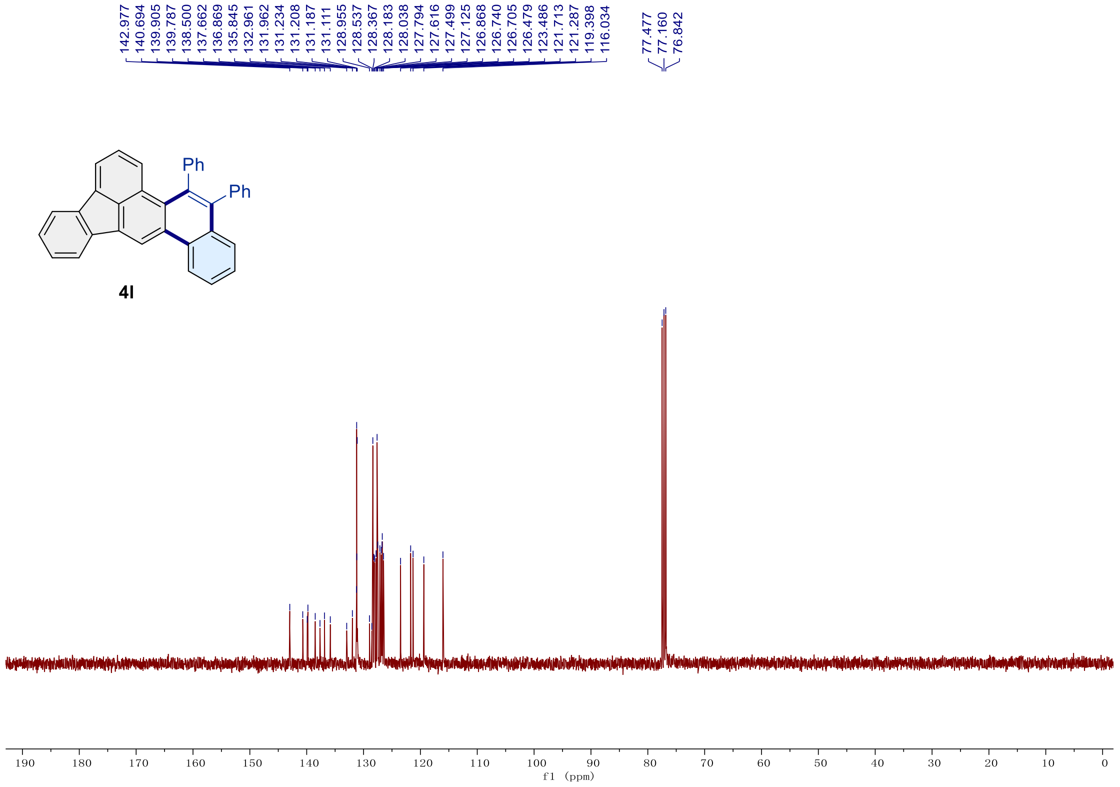

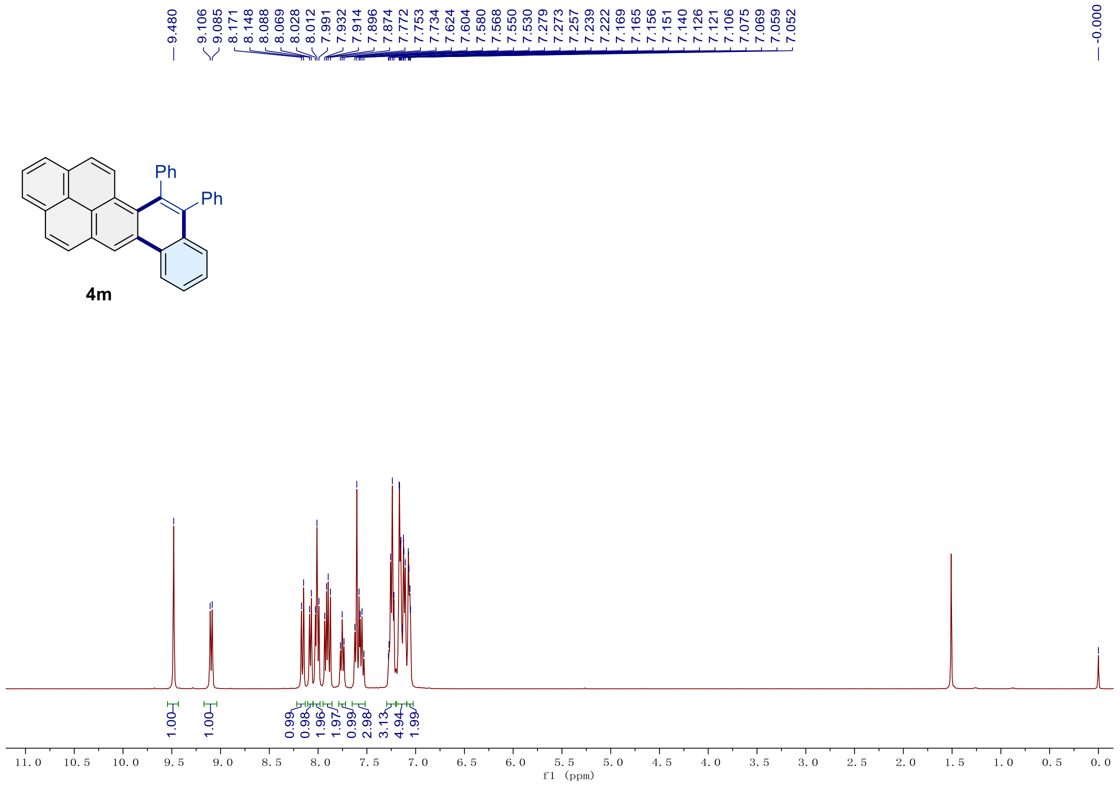
9.480
9.106
9.085
8.171
8.148
8.088
8.069
8.028
8.012
7.991
7.932
7.914
7.896
7.874
7.772
7.753
7.734
7.624
7.604
7.580
7.568
7.550
7.530
7.279
7.273
7.257
7.239
7.222
7.169
7.165
7.156
7.151
7.140
7.126
7.121
7.106
7.075
7.069
7.059
7.052
-0.000
Ph
Ph
4m
1.00
1.00
0.99
0.98
1.96
1.97
0.99
2.98
3.13
4.94
1.99
f1 (ppm)

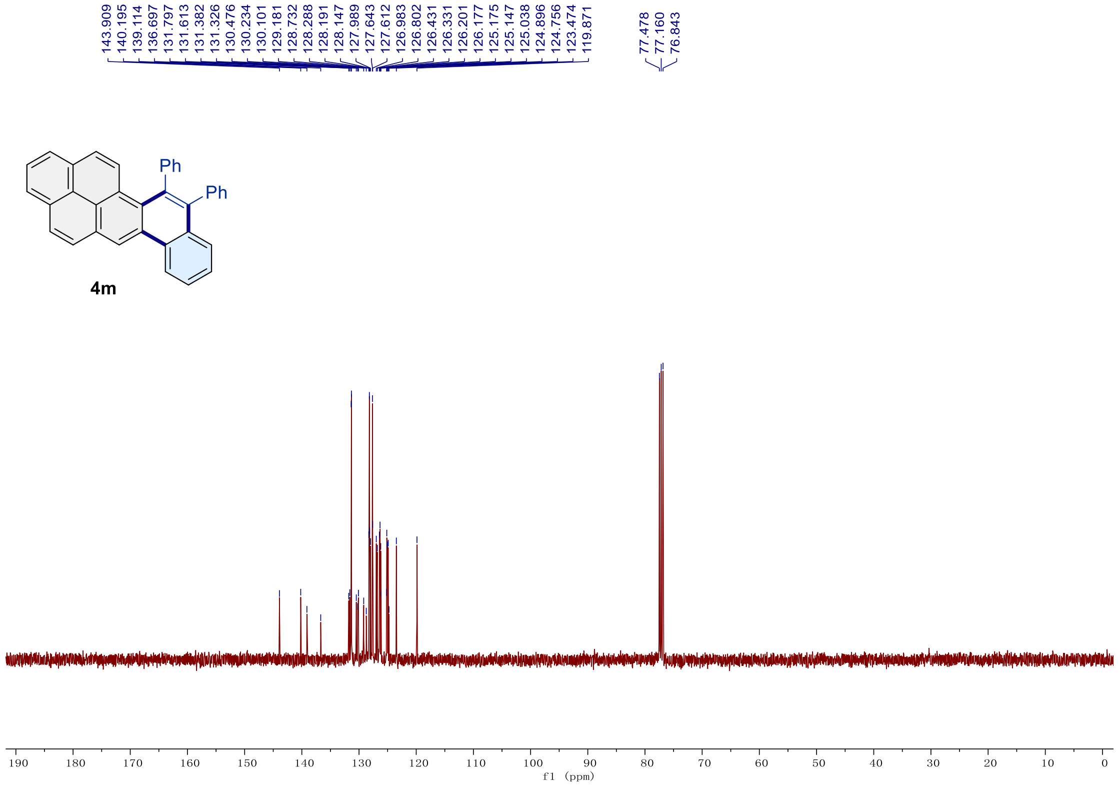
143.909
140.195
139.114
136.697
131.797
131.613
131.382
131.326
130.476
130.234
130.101
129.181
128.732
128.288
128.191
128.147
127.989
127.643
127.612
126.983
126.802
126.431
126.331
126.201
126.177
125.175
125.147
125.038
124.896
124.756
123.474
119.871
77.478
77.160
76.843
Ph
Ph
4m
f1 (ppm)

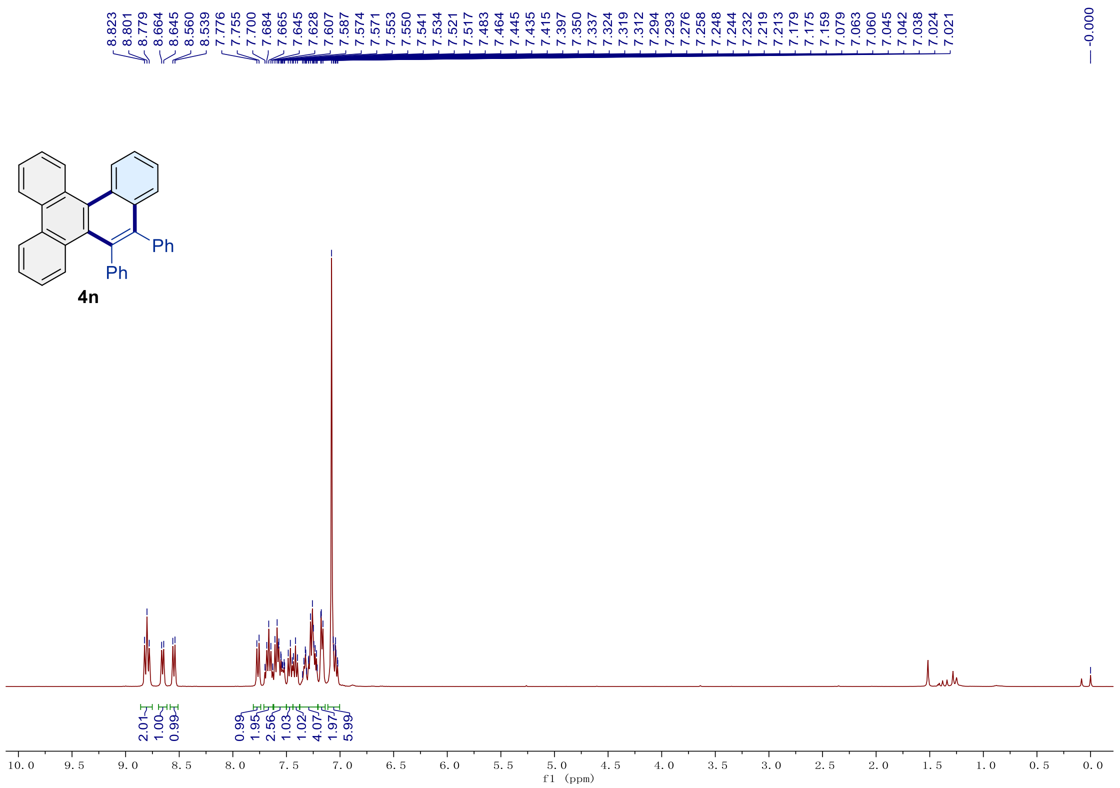
8.823
8.801
8.779
8.664
8.645
8.560
8.539
7.776
7.755
7.700
7.684
7.665
7.645
7.628
7.607
7.587
7.574
7.571
7.553
7.550
7.541
7.534
7.521
7.517
7.483
7.464
7.445
7.435
7.415
7.397
7.350
7.337
7.324
7.319
7.312
7.294
7.293
7.276
7.258
7.248
7.244
7.232
7.219
7.213
7.179
7.175
7.159
7.079
7.063
7.060
7.045
7.042
7.038
7.024
7.021
-0.000
Ph
Ph
4n
2.01
1.00
0.99
0.99
1.95
2.56
1.03
1.02
4.07
1.97
5.99
f1 (ppm)

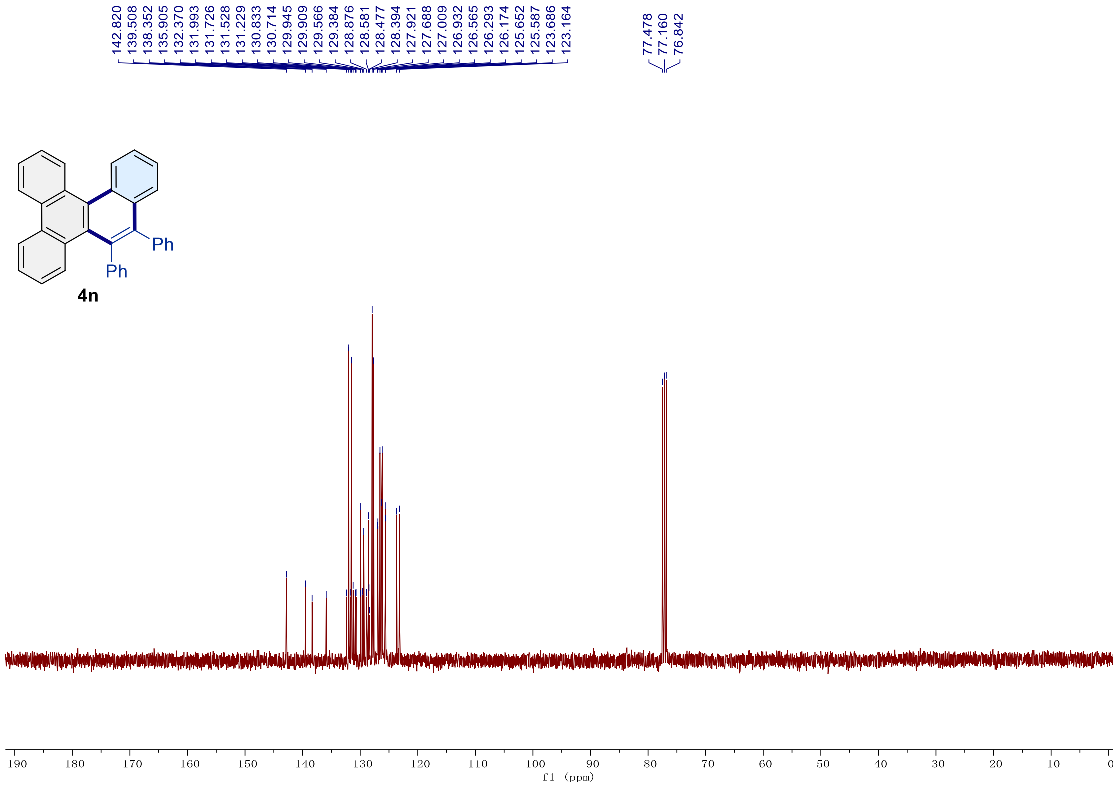
142.820
139.508
138.352
135.905
132.370
131.993
131.726
131.528
131.229
130.833
130.714
129.945
129.909
129.566
129.384
128.876
128.581
128.477
128.394
127.921
127.688
127.009
126.932
126.565
126.293
126.174
125.652
125.587
123.686
123.164
77.478
77.160
76.842
Ph
Ph
4n
f1 (ppm)

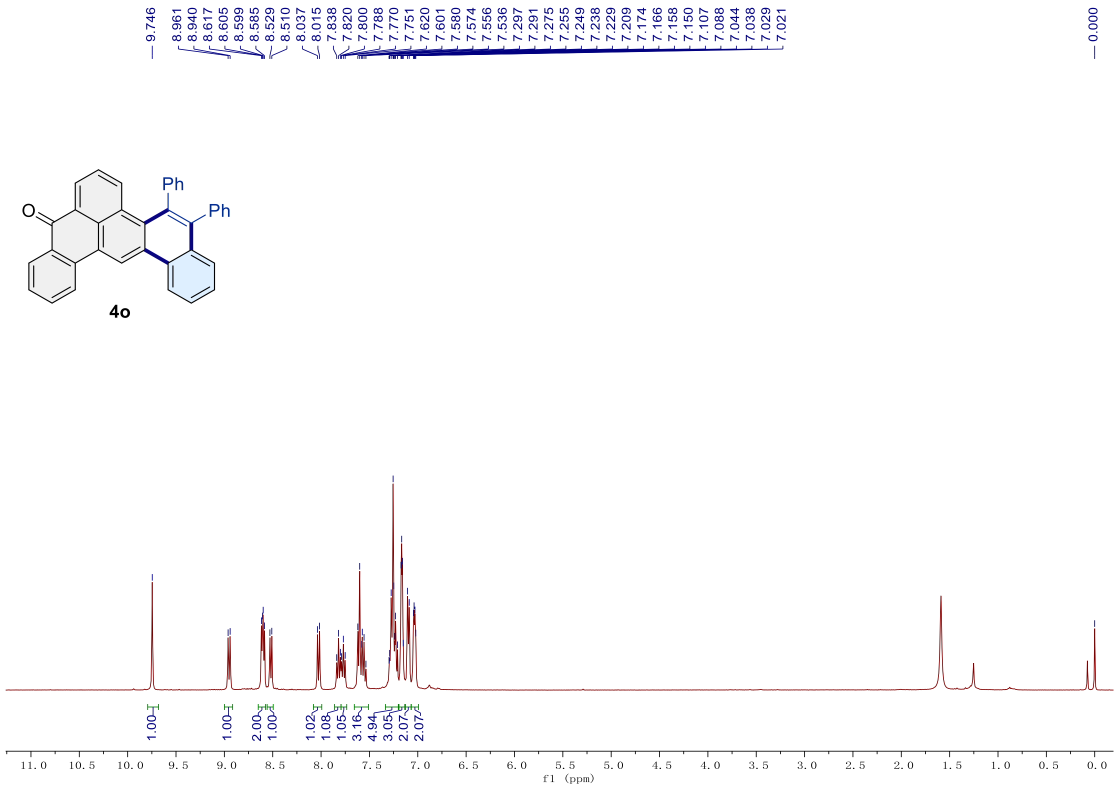
9.746
8.961
8.940
8.617
8.605
8.599
8.585
8.529
8.510
8.037
8.015
7.838
7.820
7.800
7.788
7.770
7.751
7.620
7.601
7.580
7.574
7.556
7.536
7.297
7.291
7.275
7.255
7.249
7.238
7.229
7.209
7.174
7.166
7.158
7.150
7.107
7.088
7.044
7.038
7.029
7.021
0.000
O
Ph
Ph
4o
1.00
1.00
2.00
1.00
1.02
1.08
1.05
3.16
4.94
3.05
2.07
2.07
11.0 10.5 10.0 9.5 9.0 8.5 8.0 7.5 7.0 6.5 6.0 5.5 5.0 4.5 4.0 3.5 3.0 2.5 2.0 1.5 1.0 0.5 0.0
f1 (ppm)

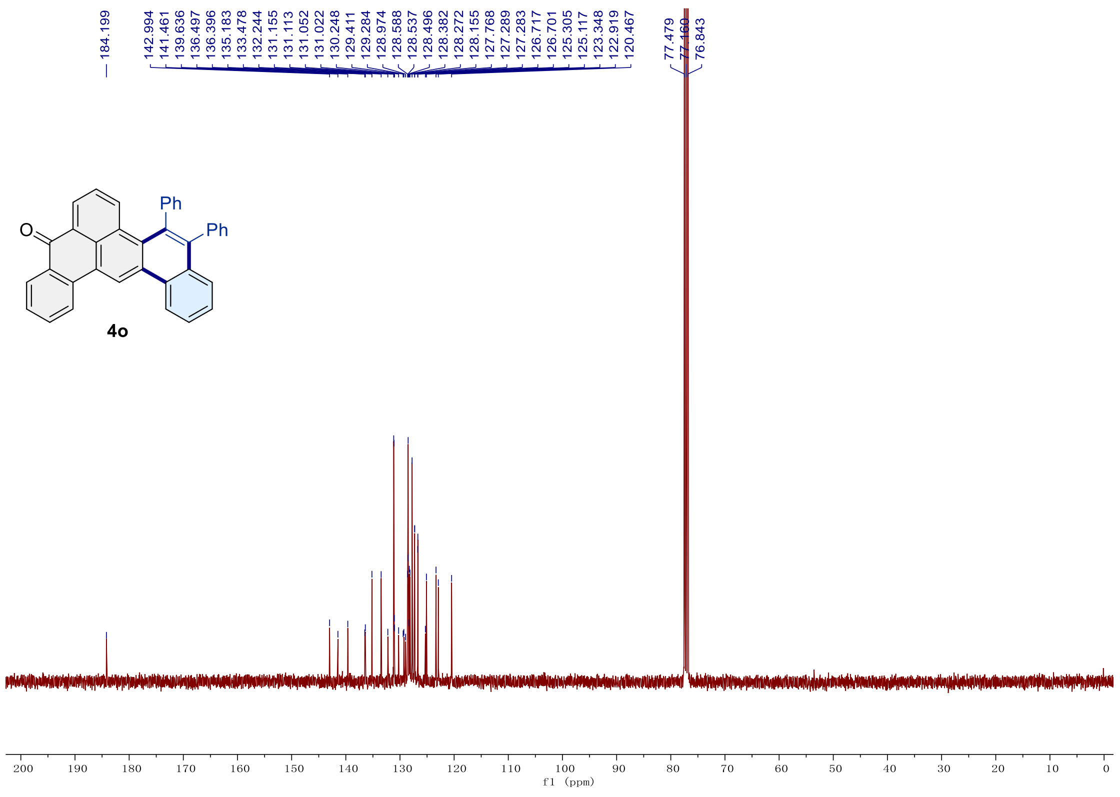
184.199
142.994
141.461
139.636
136.497
136.396
135.183
133.478
132.244
131.155
131.113
131.052
131.022
130.248
129.411
129.284
128.974
128.588
128.537
128.496
128.382
128.272
128.155
127.768
127.289
127.283
126.717
126.701
125.305
125.117
123.348
122.919
120.467
77.479
77.160
76.843
O
Ph
Ph
4o
200 190 180 170 160 150 140 130 120 110 100 90 80 70 60 50 40 30 20 10 0
f1 (ppm)

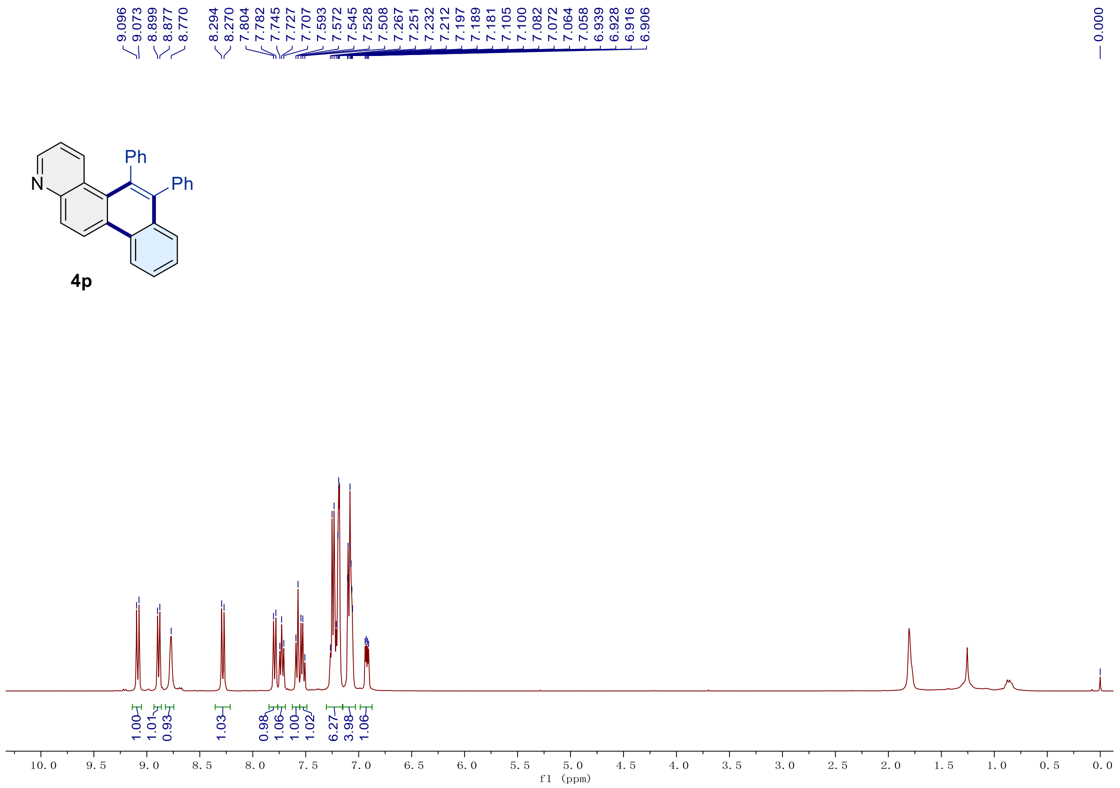
9.096
9.073
8.899
8.877
8.770
8.294
8.270
7.804
7.782
7.745
7.727
7.707
7.593
7.572
7.545
7.528
7.508
7.267
7.251
7.232
7.212
7.197
7.189
7.181
7.105
7.100
7.082
7.072
7.064
7.058
6.939
6.928
6.916
6.906
0.000
Ph
Ph
N
4p
1.00
1.01
0.93
1.03
0.98
1.06
1.00
1.02
6.27
3.98
1.06
f1 (ppm)

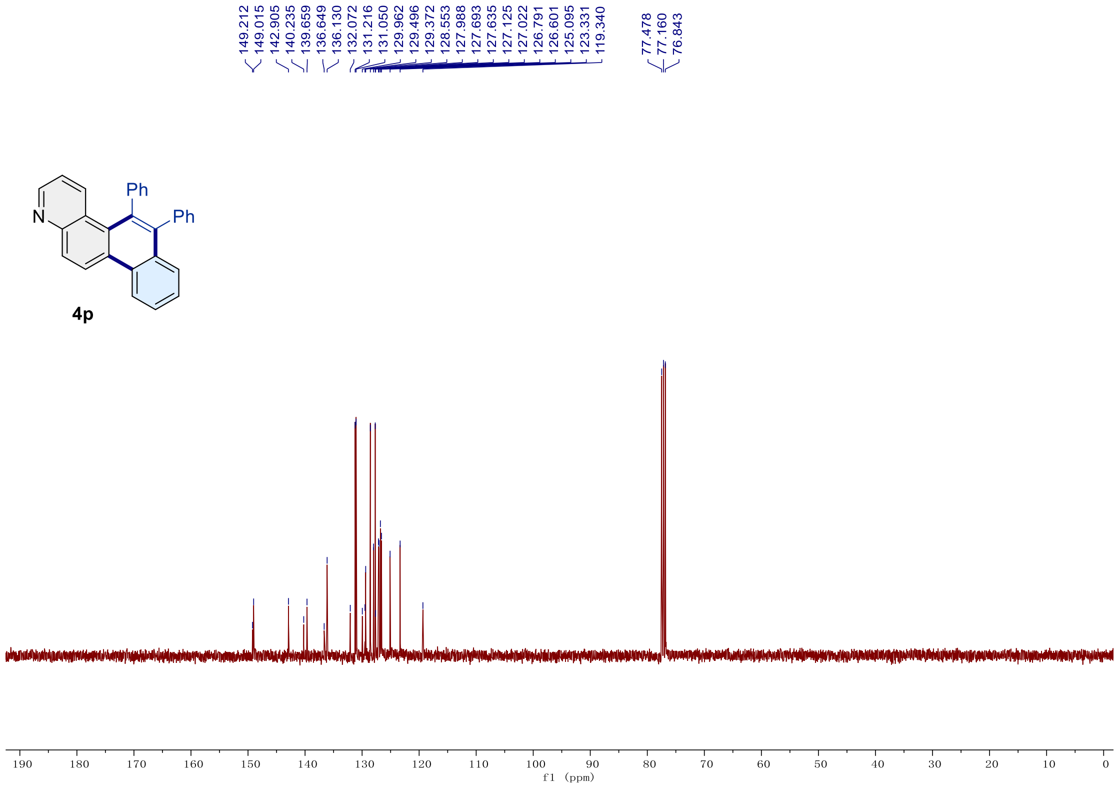
149.212
149.015
142.905
140.235
139.659
136.649
136.130
132.072
131.216
131.050
129.962
129.496
129.372
128.553
127.988
127.693
127.635
127.125
127.022
126.791
126.601
125.095
123.331
119.340
77.478
77.160
76.843
Ph
Ph
N
4p
f1 (ppm)

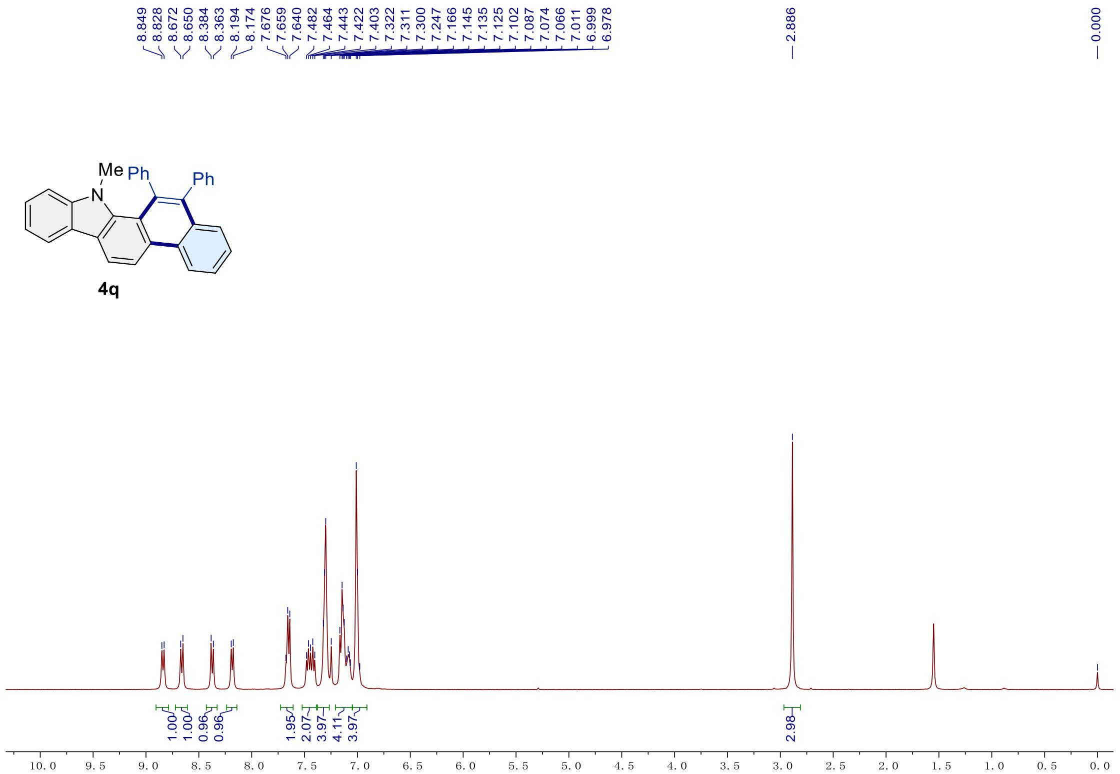
8.849
8.828
8.672
8.650
8.384
8.363
8.194
8.174
7.676
7.659
7.640
7.482
7.464
7.443
7.422
7.403
7.322
7.311
7.300
7.247
7.166
7.145
7.135
7.125
7.102
7.087
7.074
7.066
7.011
6.999
6.978
2.886
0.000
Me
N
Ph
Ph
4q
1.00
1.00
0.96
0.96
1.95
2.07
3.97
4.11
3.97
2.98
f1 (ppm)

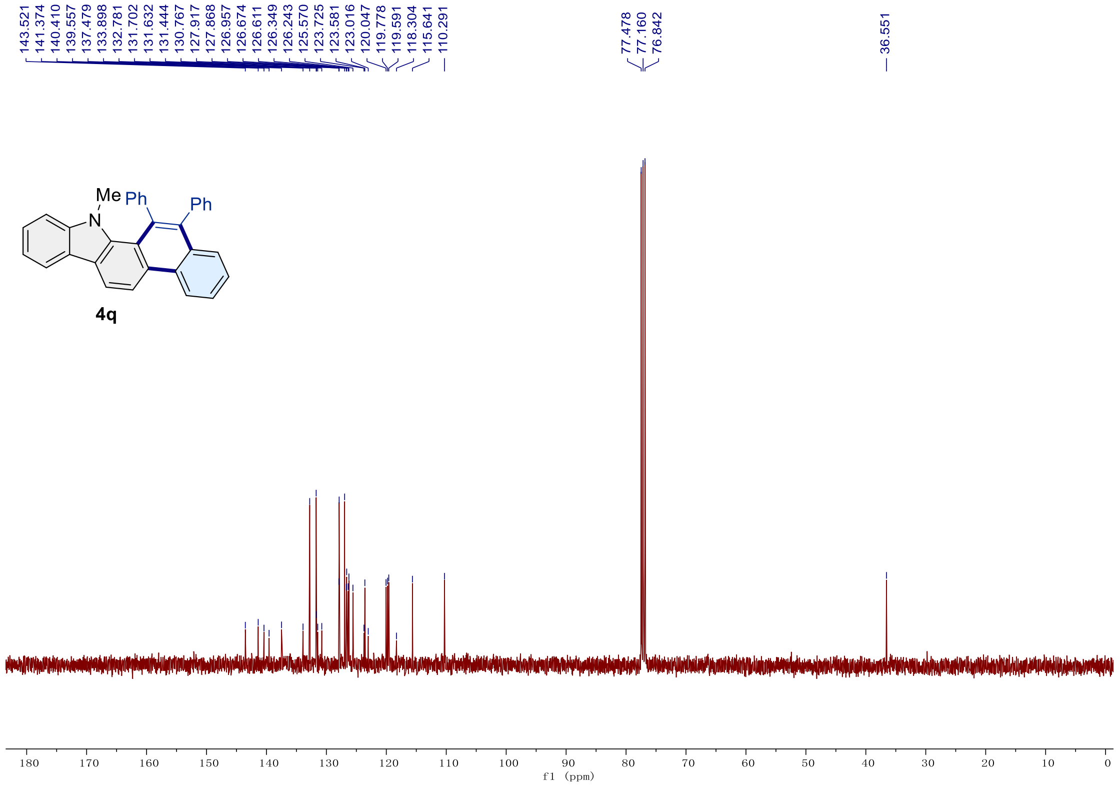
143.521
141.374
140.410
139.557
137.479
133.898
132.781
131.702
131.632
131.444
130.767
127.917
127.868
126.957
126.674
126.611
126.349
126.243
125.570
123.725
123.581
123.016
120.047
119.778
119.591
118.304
115.641
110.291
77.478
77.160
76.842
36.551
Me
N
Ph
Ph
4q
f1 (ppm)

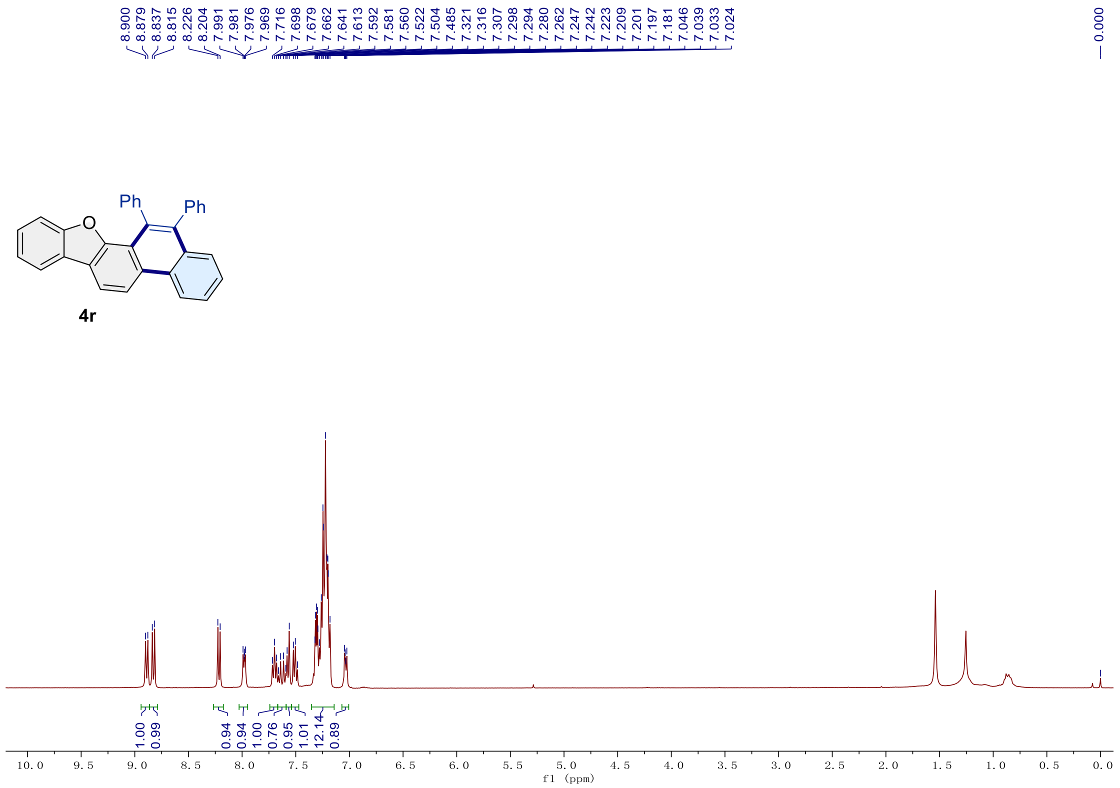
8.900
8.879
8.837
8.815
8.226
8.204
7.991
7.981
7.976
7.969
7.716
7.698
7.679
7.662
7.641
7.613
7.592
7.581
7.560
7.522
7.504
7.485
7.321
7.316
7.307
7.298
7.294
7.280
7.262
7.247
7.242
7.223
7.209
7.201
7.197
7.181
7.046
7.039
7.033
7.024
0.000
Ph
Ph
O
4r
1.00
0.99
0.94
0.94
1.00
0.76
0.95
1.01
12.14
0.89
f1 (ppm)

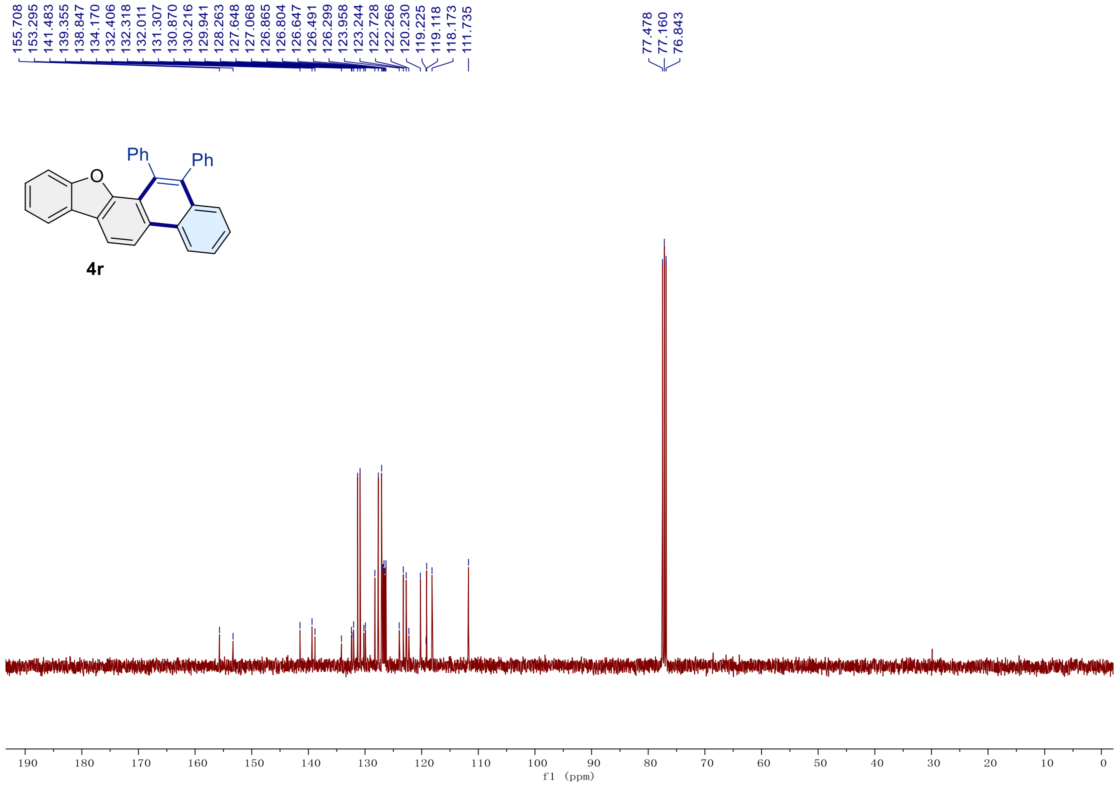
155.708
153.295
141.483
139.355
138.847
134.170
132.406
132.318
132.011
131.307
130.870
130.216
129.941
128.263
127.648
127.068
126.865
126.804
126.647
126.491
126.299
123.958
123.244
122.728
122.266
120.230
119.225
119.118
118.173
111.735
77.478
77.160
76.843
Ph
Ph
O
4r
f1 (ppm)

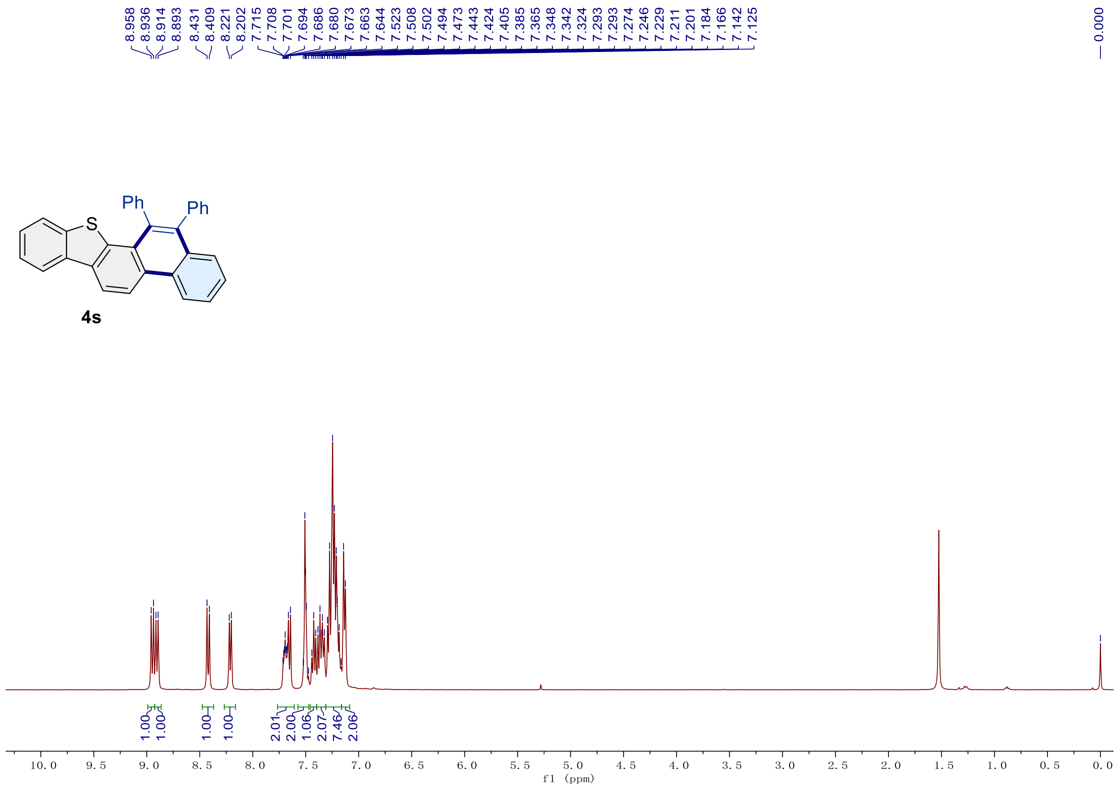
8.958
8.936
8.914
8.893
8.431
8.409
8.221
8.202
7.715
7.708
7.701
7.694
7.686
7.680
7.673
7.663
7.644
7.523
7.508
7.502
7.494
7.473
7.443
7.424
7.405
7.385
7.365
7.348
7.342
7.324
7.293
7.293
7.274
7.246
7.229
7.211
7.201
7.184
7.166
7.142
7.125
0.000
Ph
Ph
S
4s
1.00
1.00
1.00
1.00
2.01
2.00
1.06
2.07
7.46
2.06
10.0 9.5 9.0 8.5 8.0 7.5 7.0 6.5 6.0 5.5 5.0 4.5 4.0 3.5 3.0 2.5 2.0 1.5 1.0 0.5 0.0
f1 (ppm)

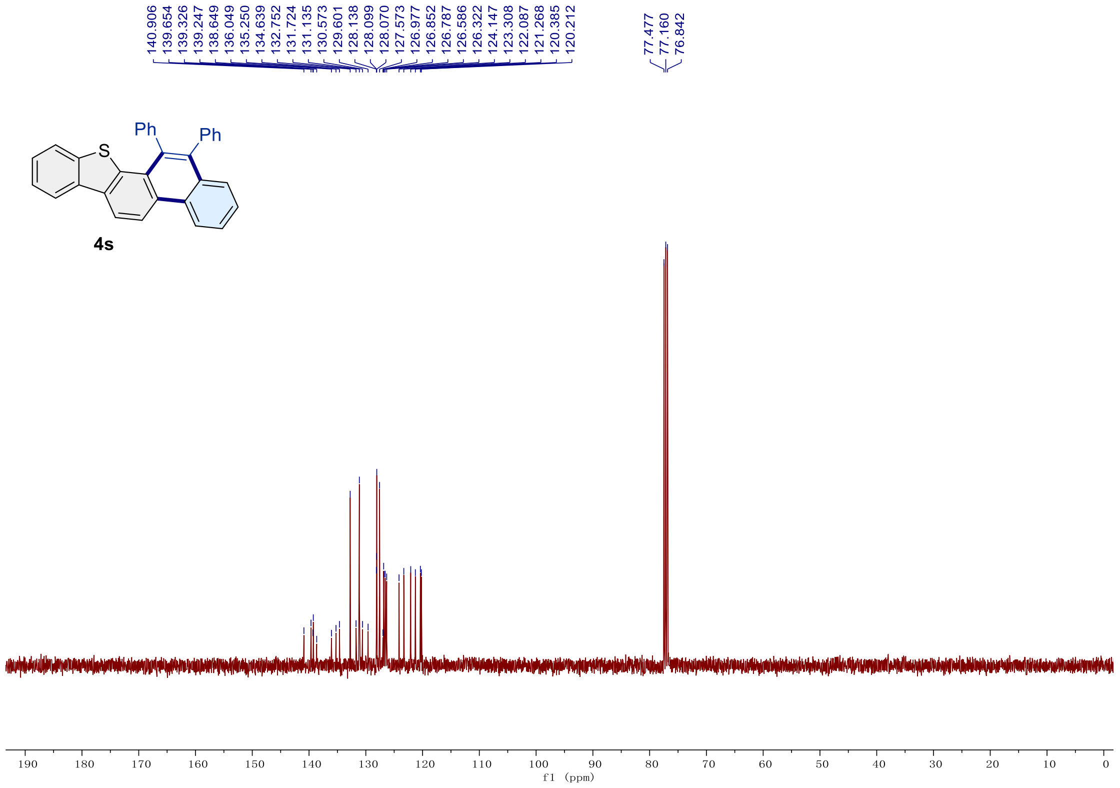
140.906
139.654
139.326
139.247
138.649
136.049
135.250
134.639
132.752
132.724
131.724
131.135
130.573
129.601
128.138
128.099
128.070
127.573
126.977
126.852
126.787
126.586
126.322
124.147
123.308
122.087
121.268
120.385
120.212
77.477
77.160
76.842
Ph
Ph
S
4s
190 180 170 160 150 140 130 120 110 100 90 80 70 60 50 40 30 20 10 0
f1 (ppm)

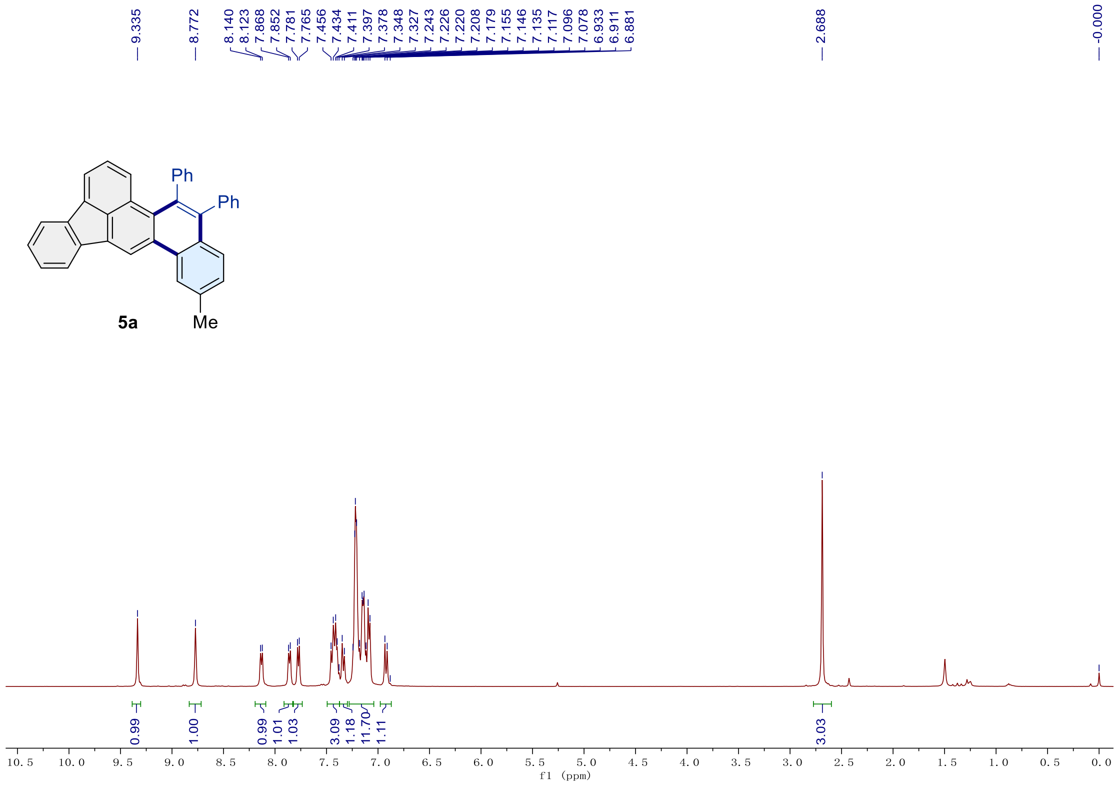

9.335
8.772
8.140
8.123
7.868
7.852
7.781
7.765
7.456
7.434
7.411
7.397
7.378
7.348
7.327
7.243
7.226
7.220
7.208
7.179
7.155
7.146
7.135
7.117
7.096
7.078
6.933
6.911
6.881
2.688
-0.000
Ph
Ph
5a
Me
0.99
1.00
0.99
1.01
1.03
3.09
1.18
11.70
1.11
3.03
f1 (ppm)

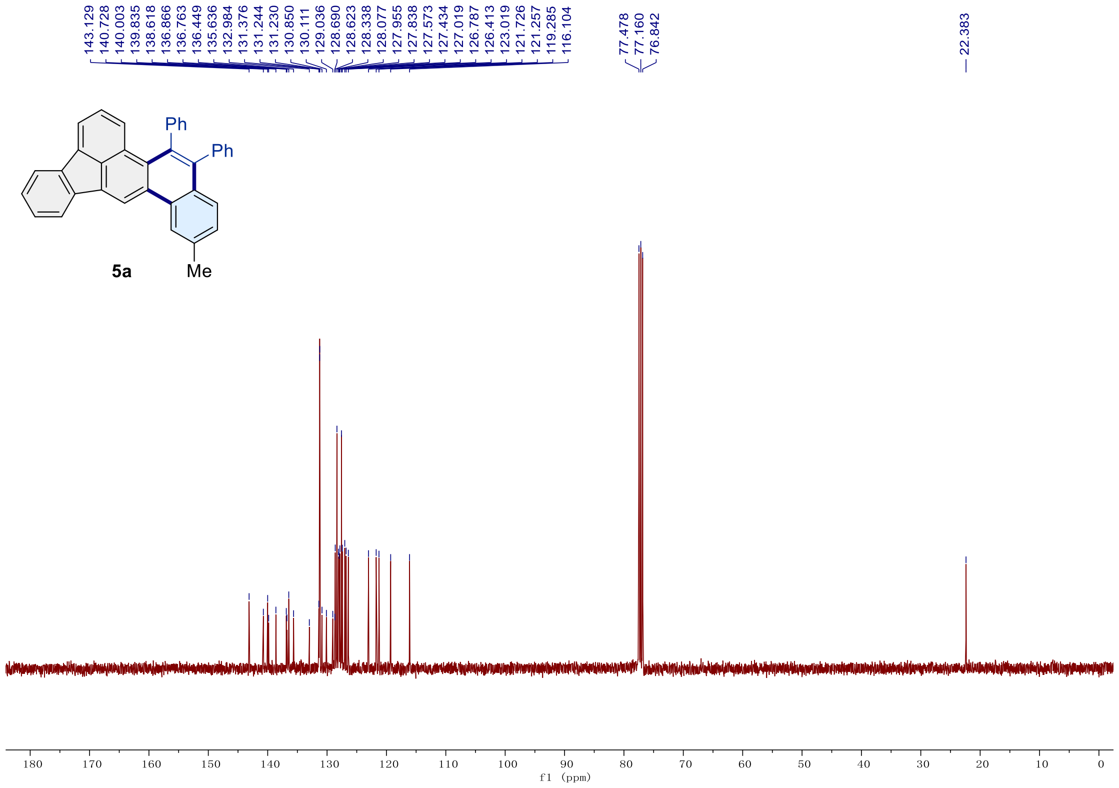

143.129
140.728
140.003
139.835
138.618
136.866
136.763
136.449
135.636
132.984
131.376
131.244
131.230
130.850
130.111
129.036
128.690
128.623
128.338
128.077
127.955
127.838
127.573
127.434
127.019
126.787
126.413
123.019
121.726
121.257
119.285
116.104
77.478
77.160
76.842
22.383
Ph
Ph
5a
Me
f1 (ppm)

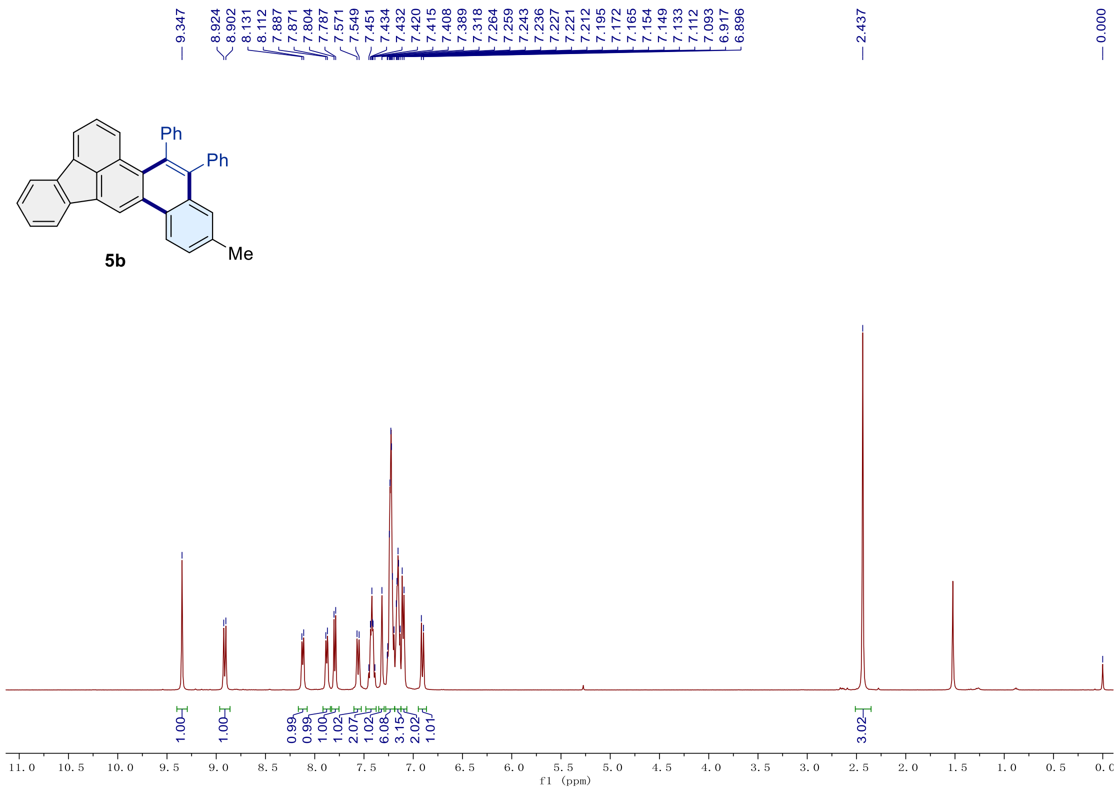

9.347
8.924
8.902
8.131
8.112
7.887
7.871
7.804
7.787
7.571
7.549
7.451
7.434
7.432
7.420
7.415
7.408
7.389
7.318
7.264
7.259
7.243
7.236
7.227
7.221
7.212
7.195
7.172
7.165
7.154
7.149
7.133
7.112
7.093
6.917
6.896
2.437
0.000
Ph
Ph
Me
5b
1.00
1.00
0.99
0.99
1.00
1.02
2.07
1.02
6.08
3.15
2.02
1.01
3.02
f1 (ppm)

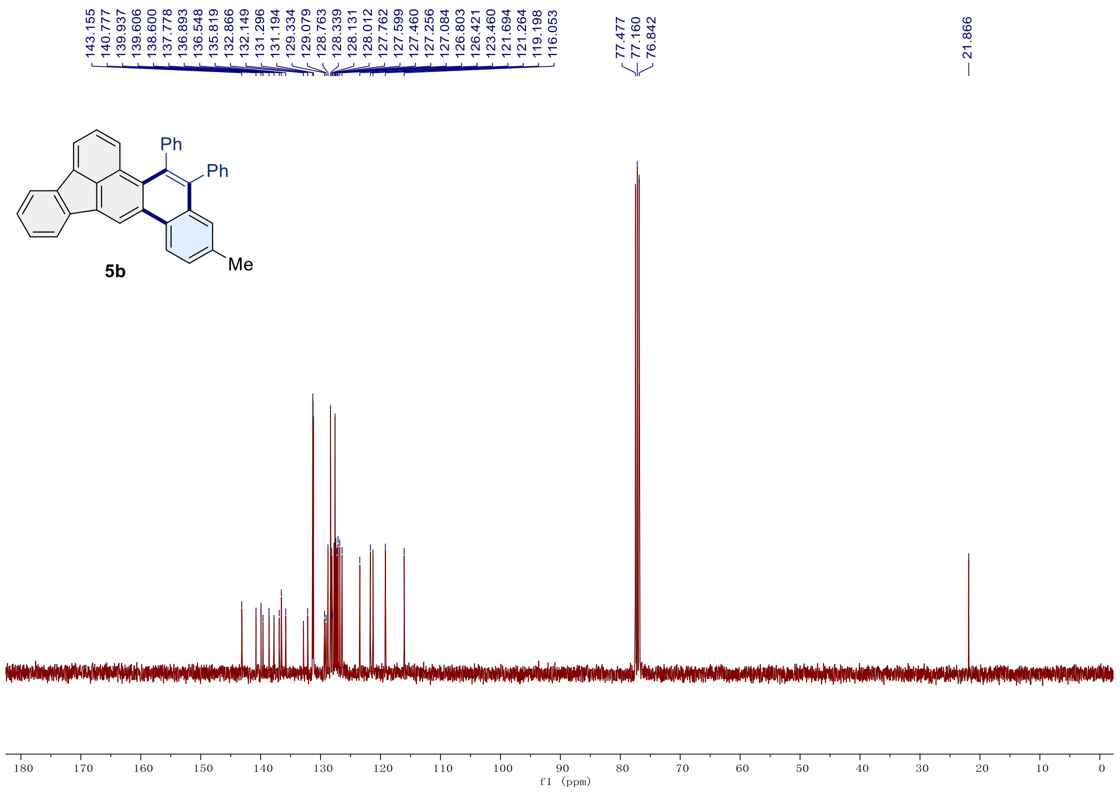

143.155
140.777
139.937
139.606
138.600
137.778
136.893
136.548
135.819
132.866
132.149
131.296
131.194
129.334
129.079
128.763
128.339
128.131
128.012
127.762
127.599
127.460
127.256
127.084
126.803
126.421
123.460
121.694
121.264
119.198
116.053
77.477
77.160
76.842
21.866
Ph
Ph
Me
5b
f1 (ppm)

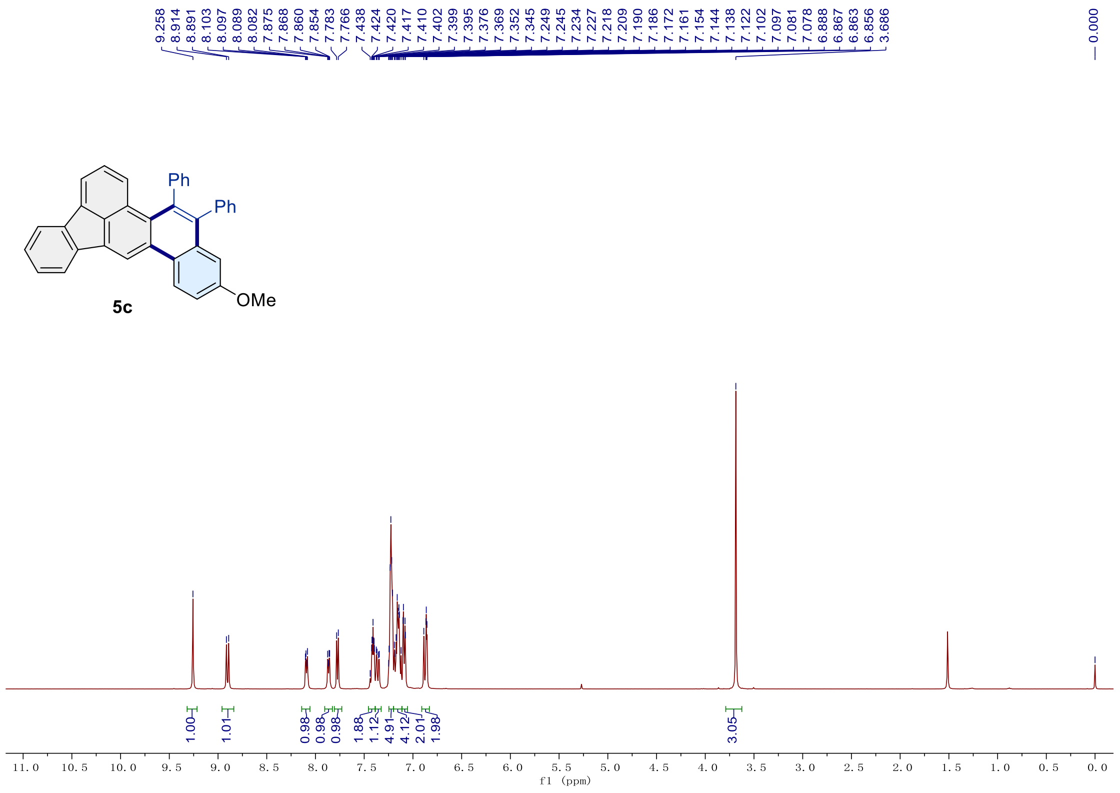
9.258
8.914
8.891
8.103
8.097
8.089
8.082
7.875
7.868
7.860
7.854
7.783
7.766
7.438
7.424
7.420
7.417
7.410
7.402
7.399
7.395
7.376
7.369
7.352
7.345
7.249
7.245
7.234
7.227
7.218
7.209
7.190
7.186
7.172
7.161
7.154
7.144
7.138
7.122
7.102
7.097
7.081
7.078
6.888
6.867
6.863
6.856
3.686
0.000
Ph
Ph
OMe
5c
1.00
1.01
0.98
0.98
0.98
1.88
1.12
4.91
4.12
2.01
1.98
3.05
f1 (ppm)

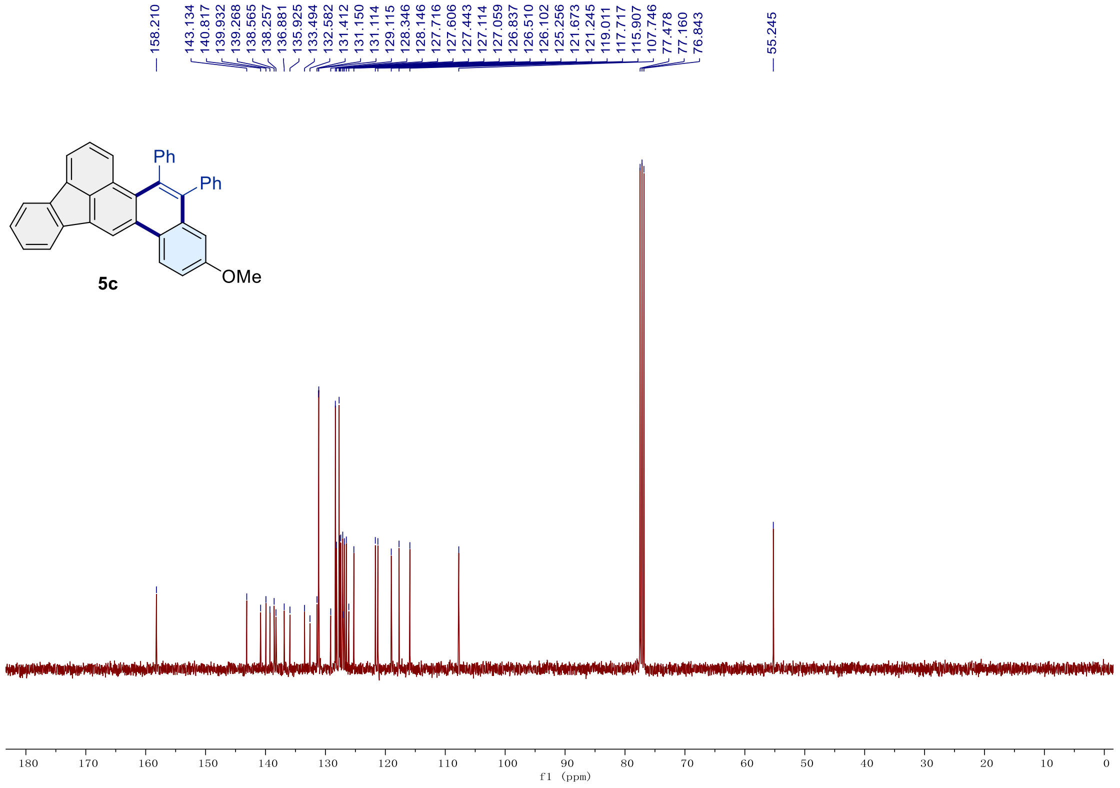
158.210
143.134
140.817
139.932
139.268
138.565
138.257
136.881
135.925
133.494
132.582
131.412
131.150
131.114
129.115
128.346
128.146
127.716
127.606
127.443
127.114
127.059
126.837
126.510
126.102
125.256
121.673
121.245
119.011
117.717
115.907
107.746
77.478
77.160
76.843
55.245
Ph
Ph
OMe
5c
f1 (ppm)

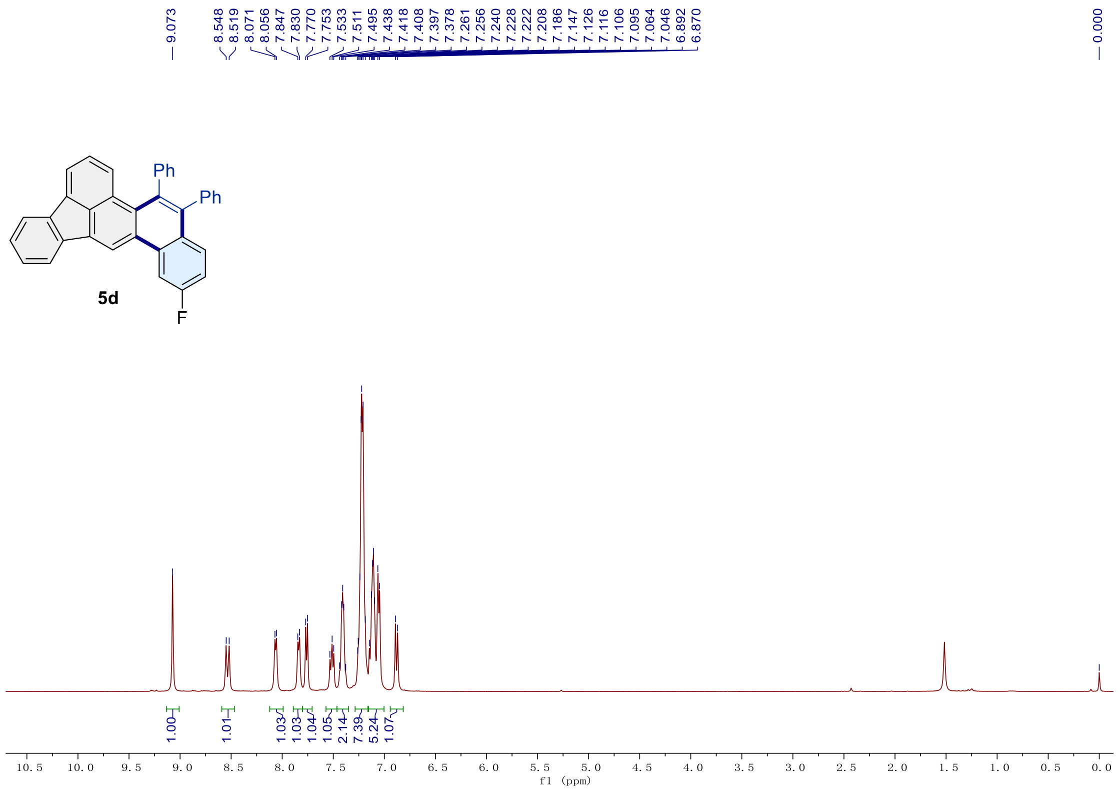
9.073
8.548
8.519
8.071
8.056
7.847
7.830
7.770
7.753
7.533
7.511
7.495
7.438
7.418
7.408
7.397
7.378
7.261
7.256
7.240
7.228
7.222
7.208
7.186
7.147
7.126
7.116
7.106
7.095
7.064
7.046
6.892
6.870
0.000
Ph
Ph
5d
F
1.00
1.01
1.03
1.03
1.04
1.05
2.14
7.39
5.24
1.07
f1 (ppm)

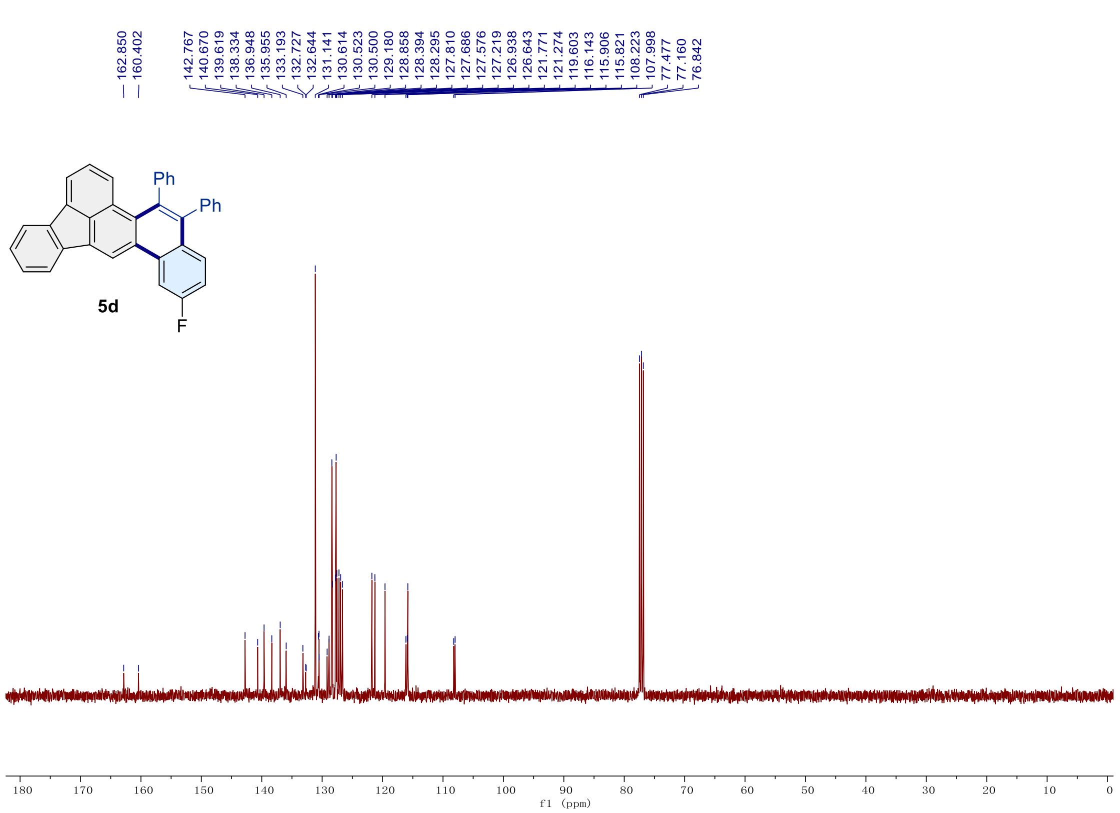
162.850
160.402
142.767
140.670
139.619
138.334
136.948
135.955
133.193
132.727
132.644
131.141
130.614
130.523
130.500
129.180
128.858
128.394
128.295
127.810
127.686
127.576
127.219
126.938
126.643
121.771
121.274
119.603
116.143
115.906
115.821
108.223
107.998
77.477
77.160
76.842
Ph
Ph
5d
F
f1 (ppm)

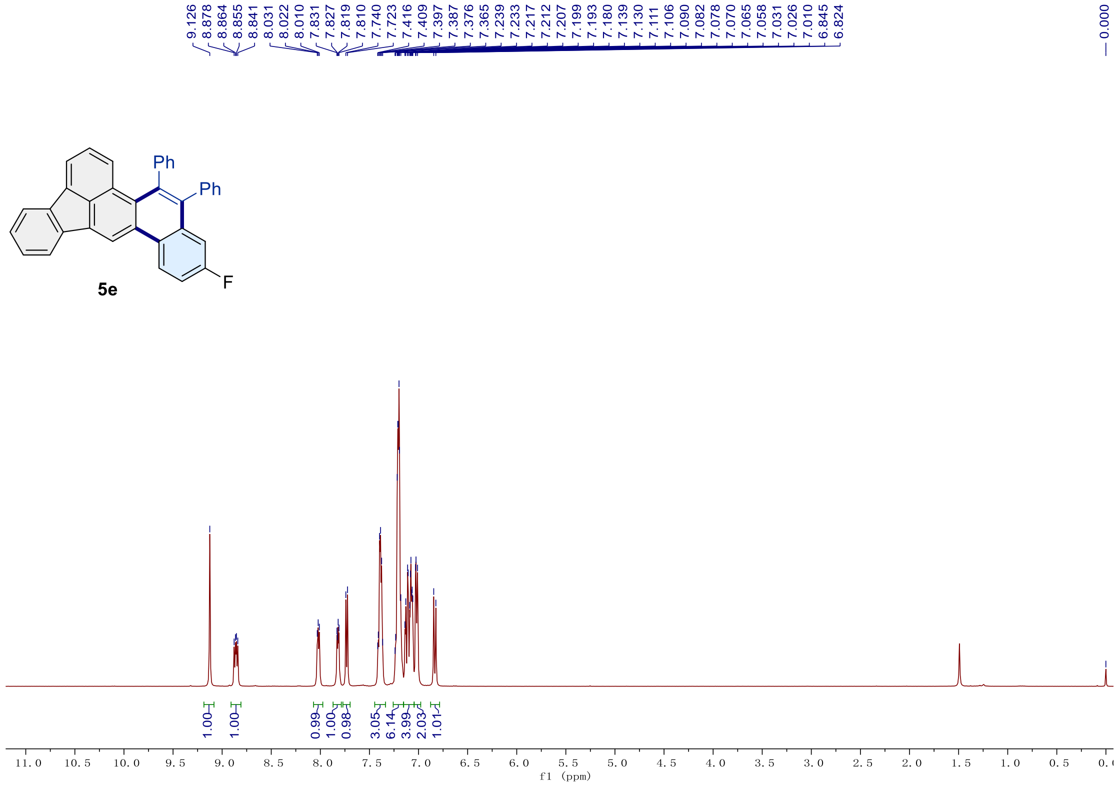
9.126
8.878
8.864
8.855
8.841
8.031
8.022
8.010
7.831
7.827
7.819
7.810
7.740
7.723
7.416
7.409
7.397
7.387
7.376
7.365
7.239
7.233
7.217
7.212
7.207
7.199
7.193
7.180
7.139
7.130
7.111
7.106
7.090
7.082
7.078
7.070
7.065
7.058
7.031
7.026
7.010
6.845
6.824
0.000
Ph
Ph
F
5e
1.00
1.00
0.99
1.00
0.98
3.05
6.14
3.99
2.03
1.01
f1 (ppm)

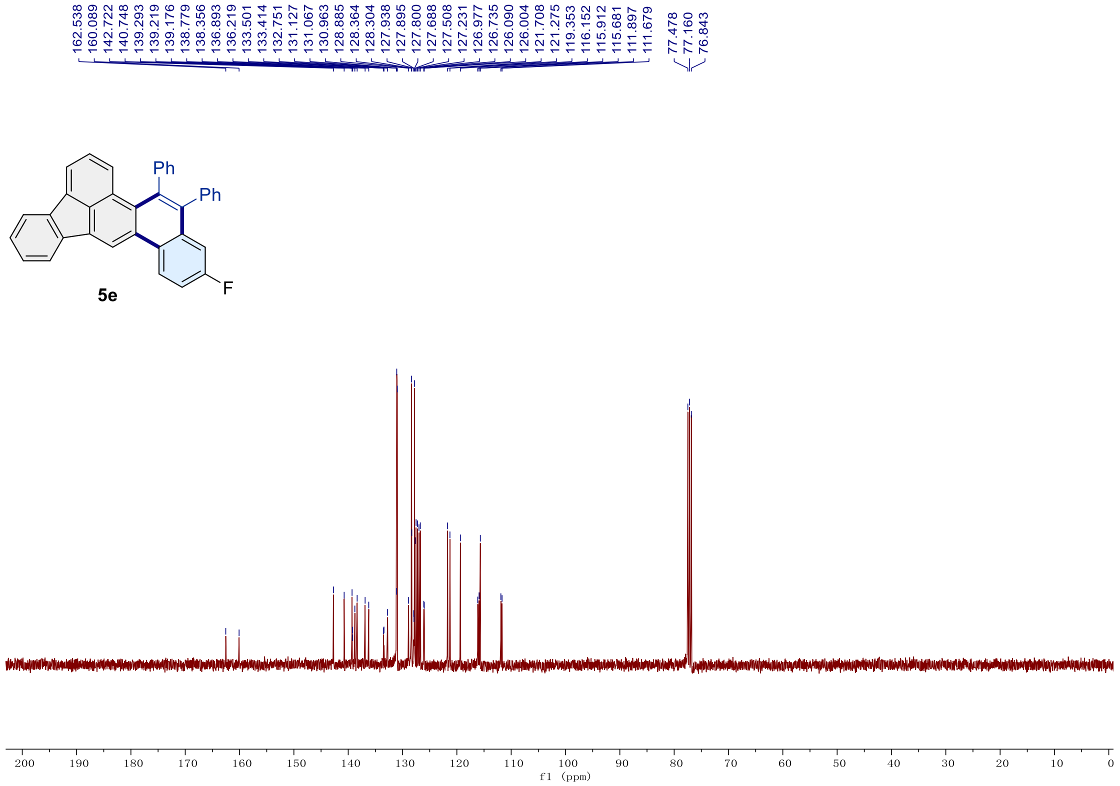
162.538
160.089
142.722
140.748
139.293
139.219
139.176
138.779
138.356
136.893
136.219
133.501
133.414
132.751
131.127
131.067
130.963
128.885
128.364
128.304
127.938
127.895
127.800
127.688
127.508
127.231
126.977
126.735
126.090
126.004
121.708
121.275
119.353
116.152
115.912
115.681
111.897
111.679
77.478
77.160
76.843
Ph
Ph
F
5e
f1 (ppm)

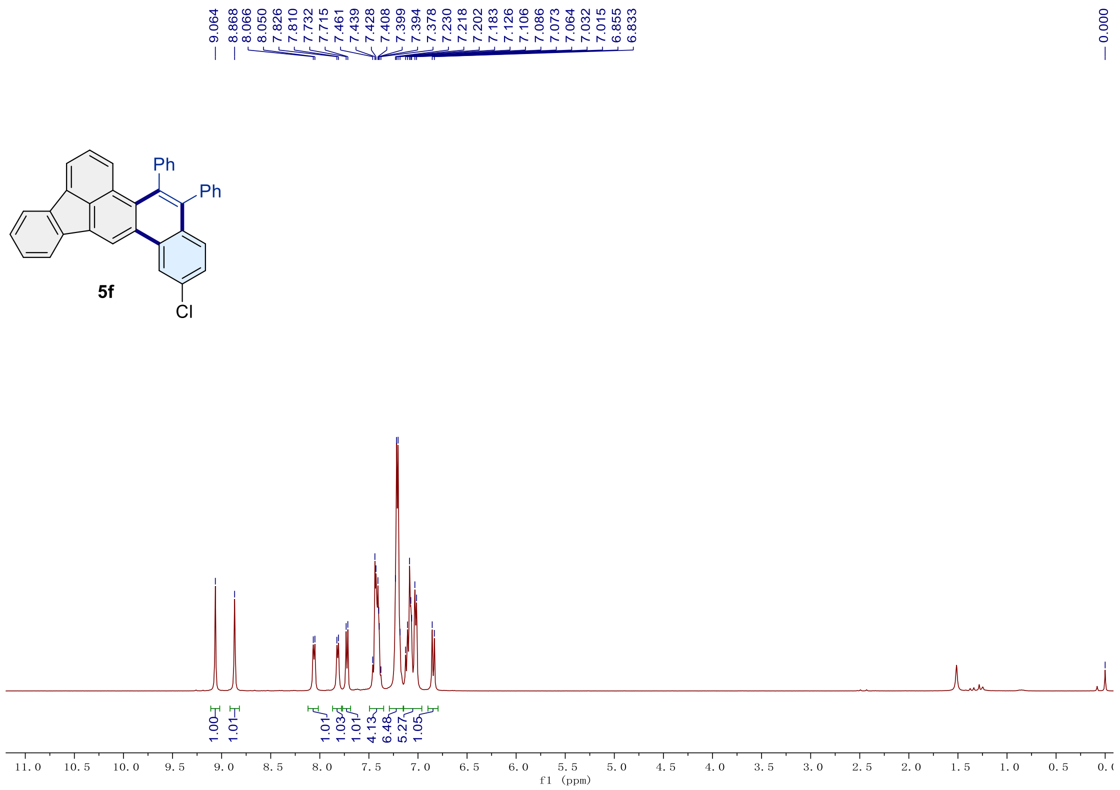
9.064
8.868
8.066
8.050
7.826
7.810
7.732
7.715
7.461
7.439
7.428
7.408
7.399
7.394
7.378
7.230
7.218
7.202
7.183
7.126
7.106
7.086
7.073
7.064
7.032
7.015
6.855
6.833
0.000
Ph
Ph
5f
Cl
1.00
1.01
1.01
1.03
1.01
4.13
6.48
5.27
1.05
f1 (ppm)

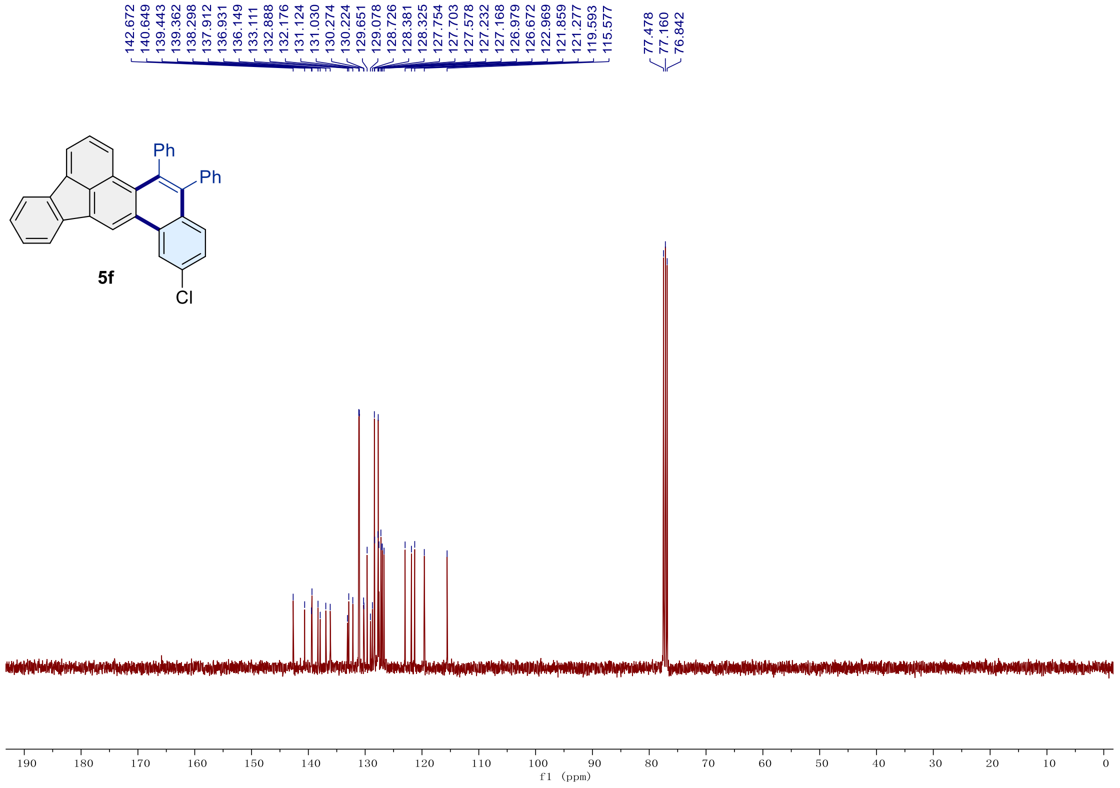
142.672
140.649
139.443
139.362
138.298
137.912
136.931
136.149
136.111
133.111
132.888
132.176
131.124
131.030
130.274
130.224
129.651
129.078
128.726
128.381
128.325
127.754
127.703
127.578
127.232
127.168
126.979
126.672
122.969
121.859
121.277
119.593
115.577
77.478
77.160
76.842
Ph
Ph
5f
Cl
f1 (ppm)

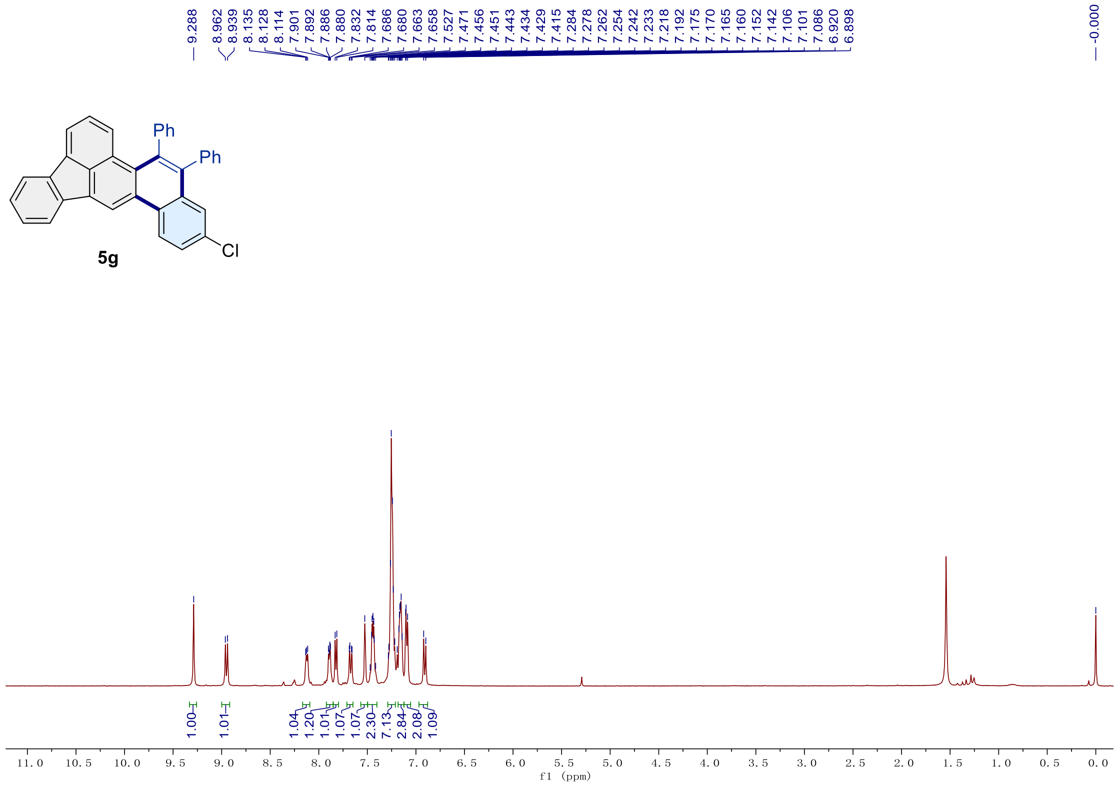
9.288
8.962
8.939
8.135
8.128
8.114
7.901
7.892
7.886
7.880
7.832
7.814
7.686
7.680
7.663
7.658
7.527
7.471
7.456
7.451
7.443
7.434
7.429
7.415
7.284
7.278
7.262
7.254
7.242
7.233
7.218
7.192
7.175
7.170
7.165
7.160
7.152
7.142
7.106
7.101
7.086
6.920
6.898
-0.000
Ph
Ph
Cl
5g
1.00
1.01
1.04
1.20
1.01
1.07
1.07
2.30
7.13
2.84
2.08
1.09
f1 (ppm)

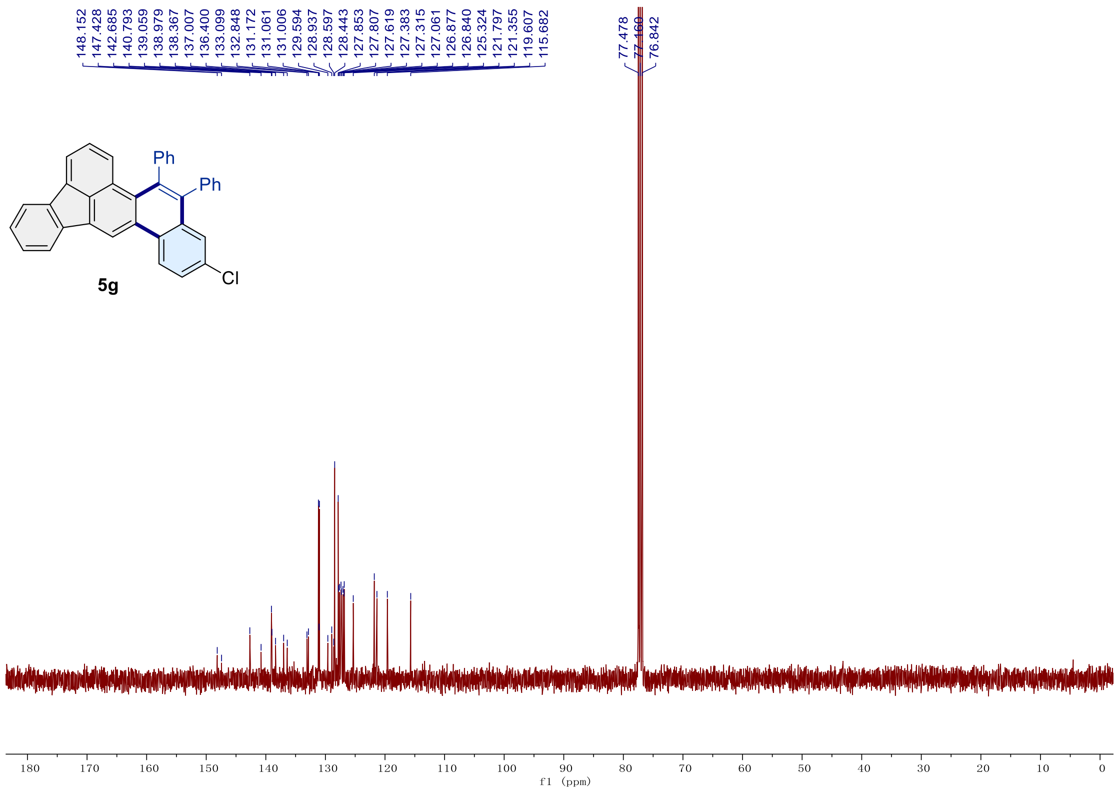
148.152
147.428
142.685
140.793
139.059
138.979
138.367
137.007
136.400
133.099
132.848
131.172
131.061
131.006
129.594
128.937
128.597
128.443
127.853
127.807
127.619
127.383
127.315
127.061
126.877
126.840
125.324
121.797
121.355
119.607
115.682
77.478
77.160
76.842
Ph
Ph
Cl
5g
f1 (ppm)

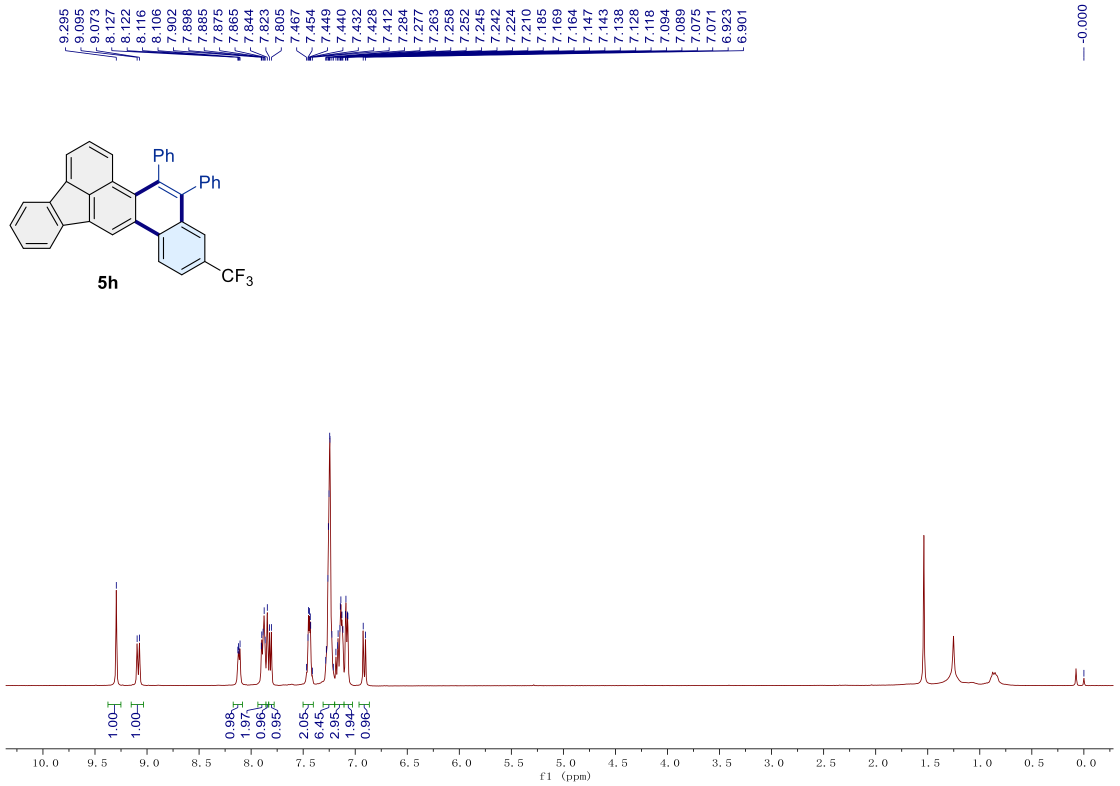
9.295
9.095
9.073
8.127
8.122
8.116
8.106
7.902
7.898
7.885
7.875
7.865
7.844
7.823
7.805
7.467
7.454
7.449
7.440
7.432
7.428
7.412
7.284
7.277
7.263
7.258
7.252
7.245
7.242
7.224
7.210
7.185
7.169
7.164
7.147
7.143
7.138
7.128
7.118
7.094
7.089
7.075
7.071
6.923
6.901
-0.000
Ph
Ph
CF3
5h
1.00
1.00
0.98
1.97
0.96
0.95
2.05
6.45
2.95
1.94
0.96
10.0 9.5 9.0 8.5 8.0 7.5 7.0 6.5 6.0 5.5 5.0 4.5 4.0 3.5 3.0 2.5 2.0 1.5 1.0 0.5 0.0
f1 (ppm)

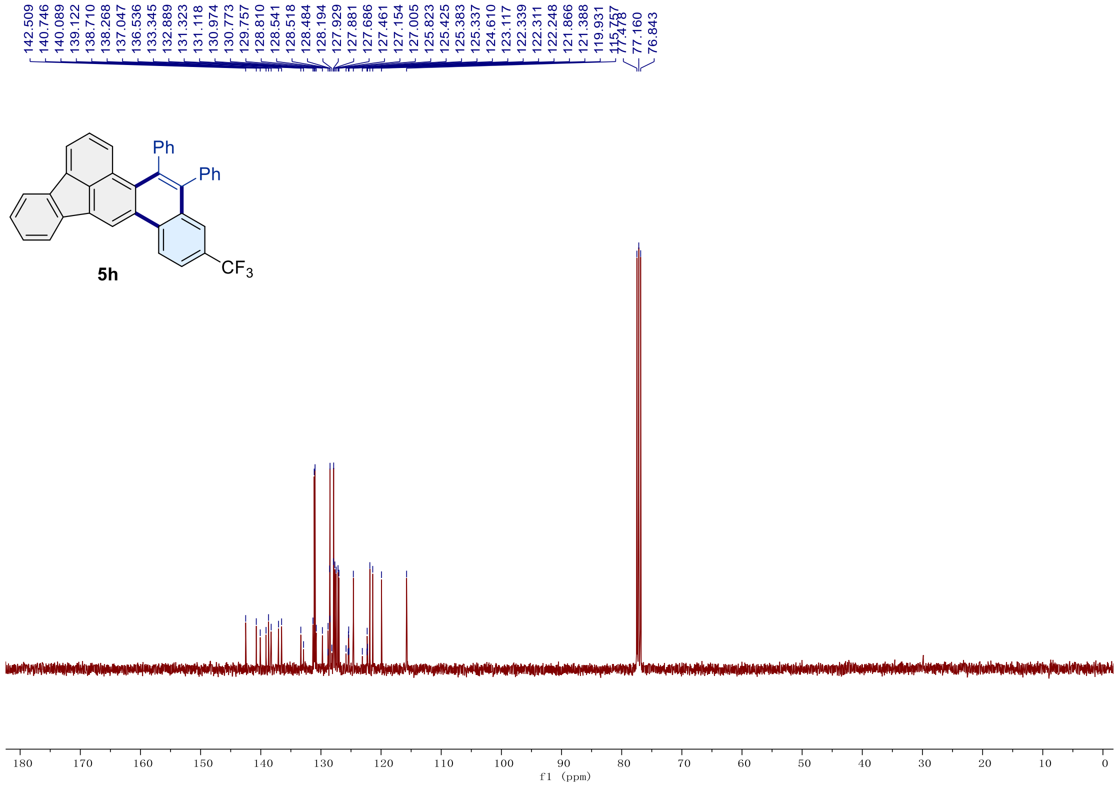
142.509
140.746
140.089
139.122
138.710
138.268
137.047
136.536
133.345
132.889
131.323
131.118
130.974
130.773
129.757
128.810
128.541
128.518
128.484
128.194
127.929
127.881
127.686
127.461
127.154
127.005
125.823
125.425
125.383
125.337
124.610
123.117
122.339
122.311
122.248
121.866
121.388
119.931
115.757
77.478
77.160
76.843
Ph
Ph
CF3
5h
180 170 160 150 140 130 120 110 100 90 80 70 60 50 40 30 20 10 0
f1 (ppm)

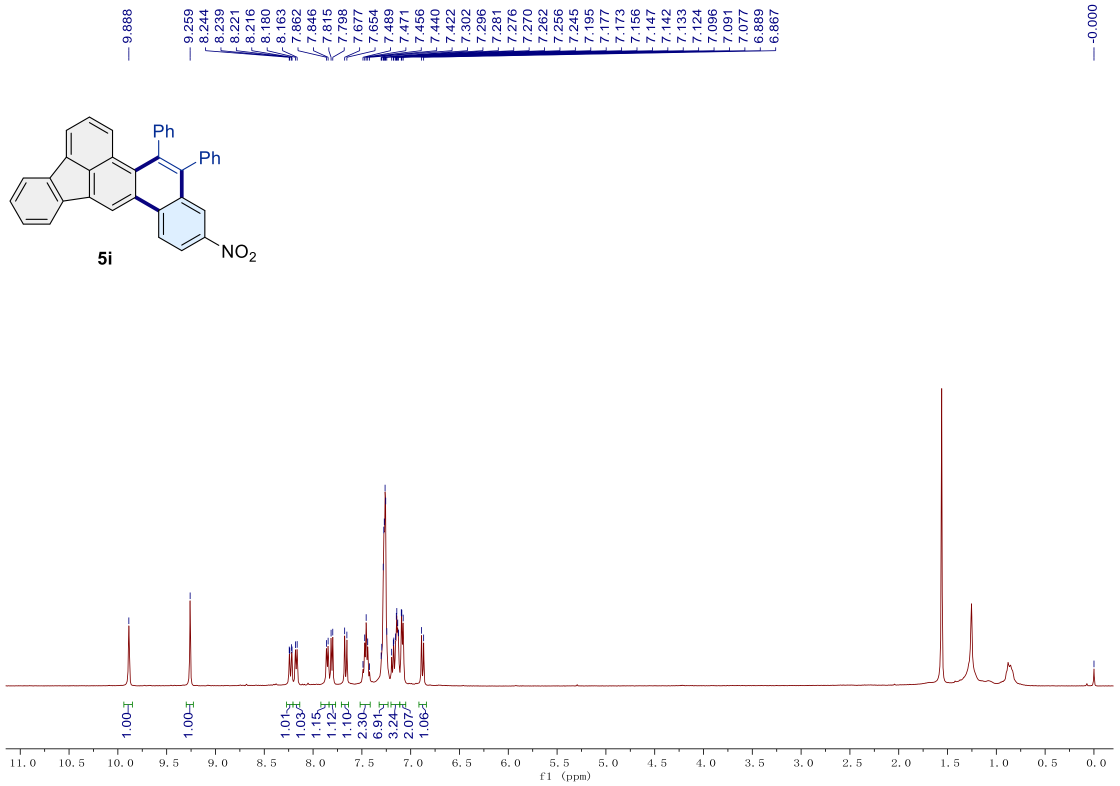
9.888
9.259
8.244
8.239
8.221
8.216
8.180
8.163
7.862
7.846
7.815
7.798
7.677
7.654
7.489
7.471
7.456
7.440
7.422
7.302
7.296
7.281
7.276
7.270
7.262
7.256
7.245
7.195
7.177
7.173
7.156
7.147
7.142
7.133
7.124
7.096
7.091
7.077
6.889
6.867
-0.000
Ph
Ph
NO2
5i
1.00
1.00
1.01
1.03
1.15
1.12
1.10
2.30
6.91
3.24
2.07
1.06
f1 (ppm)

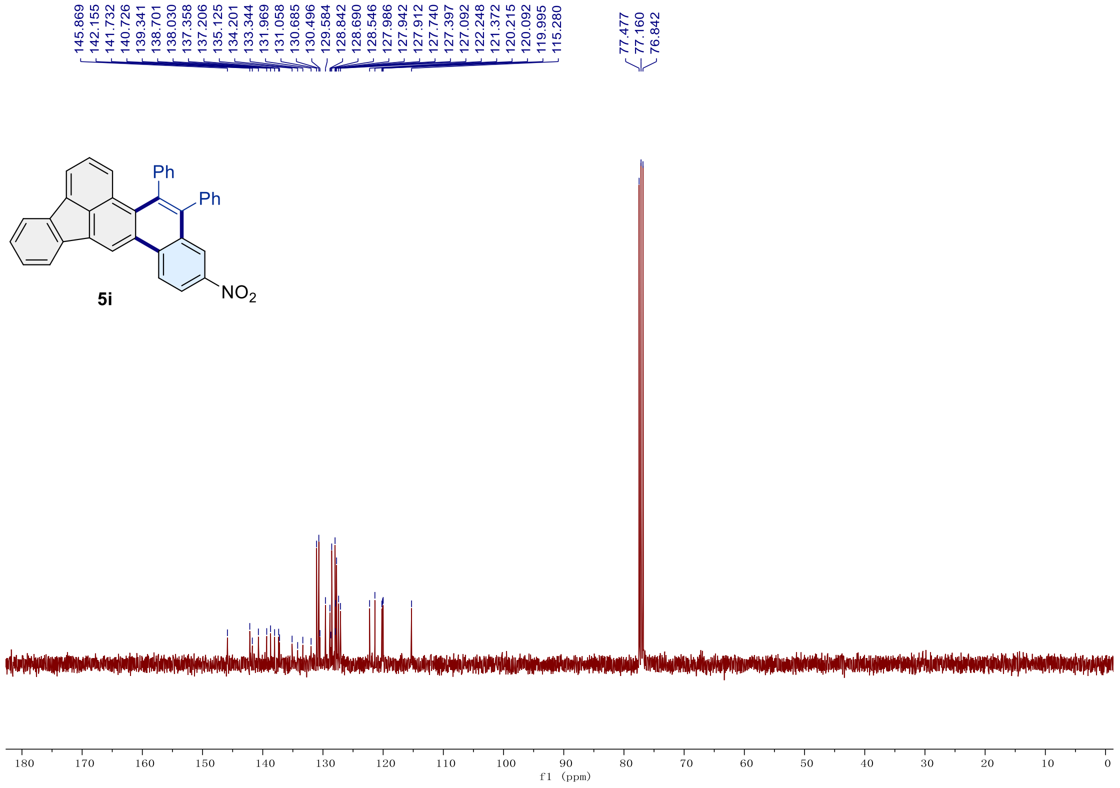
145.869
142.155
141.732
140.726
139.341
138.701
138.030
137.358
137.206
135.125
134.201
133.344
131.969
131.058
130.685
130.496
129.584
128.842
128.690
128.546
127.986
127.942
127.912
127.740
127.397
127.092
122.248
121.372
120.215
120.092
119.995
115.280
77.477
77.160
76.842
Ph
Ph
NO2
5i
f1 (ppm)

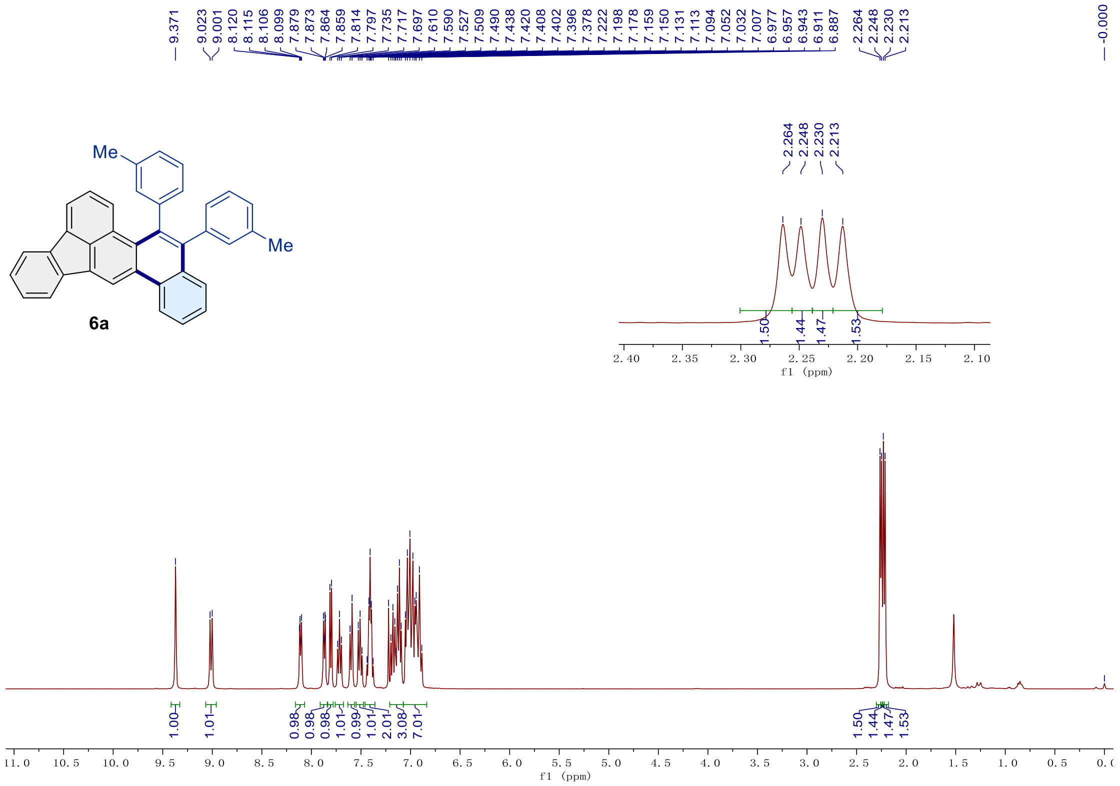

9.371
9.023
9.001
8.120
8.115
8.106
8.099
7.879
7.873
7.864
7.859
7.814
7.797
7.735
7.717
7.697
7.610
7.590
7.527
7.509
7.490
7.438
7.420
7.408
7.402
7.396
7.378
7.222
7.198
7.178
7.159
7.150
7.131
7.113
7.094
7.052
7.032
7.007
6.977
6.957
6.943
6.911
6.887
2.264
2.248
2.230
2.213
-0.000
Me
Me
6a
f1 (ppm)

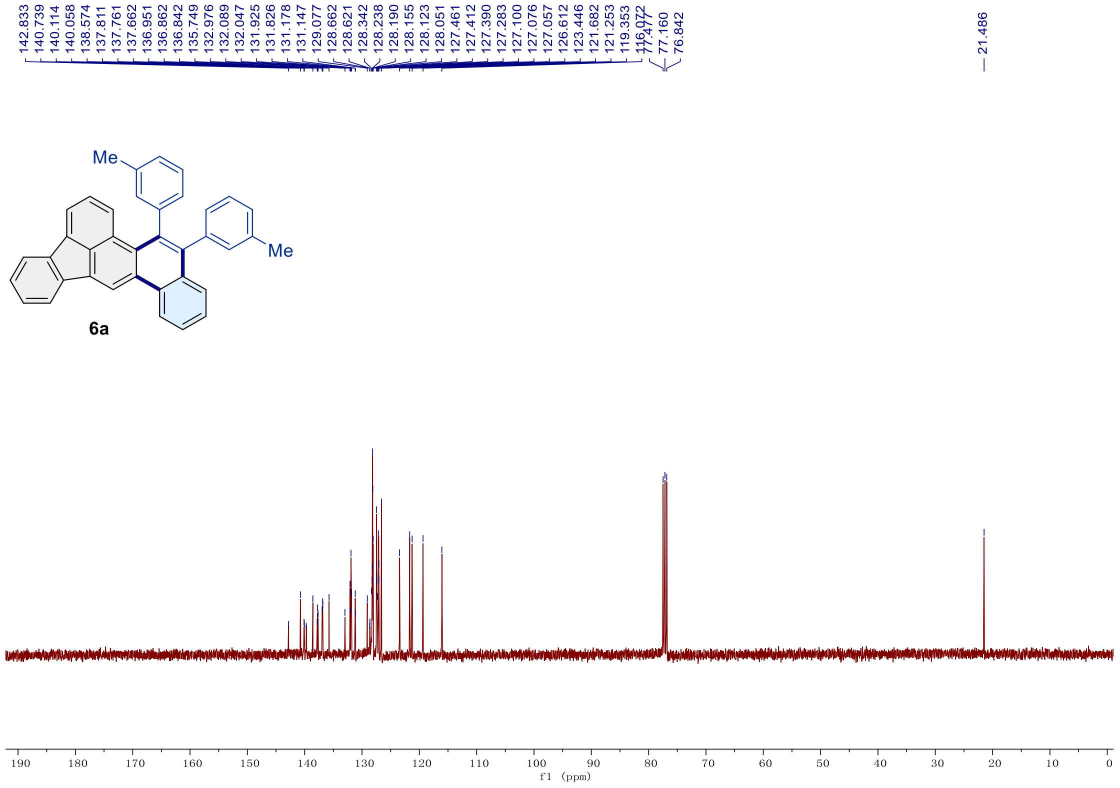

142.833
140.739
140.114
140.058
138.574
137.811
137.761
137.662
136.951
136.862
136.842
135.749
132.976
132.089
132.047
131.925
131.826
131.178
131.147
129.077
128.662
128.621
128.342
128.238
128.190
128.155
128.123
128.051
127.461
127.412
127.390
127.283
127.100
127.076
127.057
126.612
123.446
121.682
121.253
119.353
116.072
77.477
77.160
76.842
21.486
Me
Me
6a
f1 (ppm)

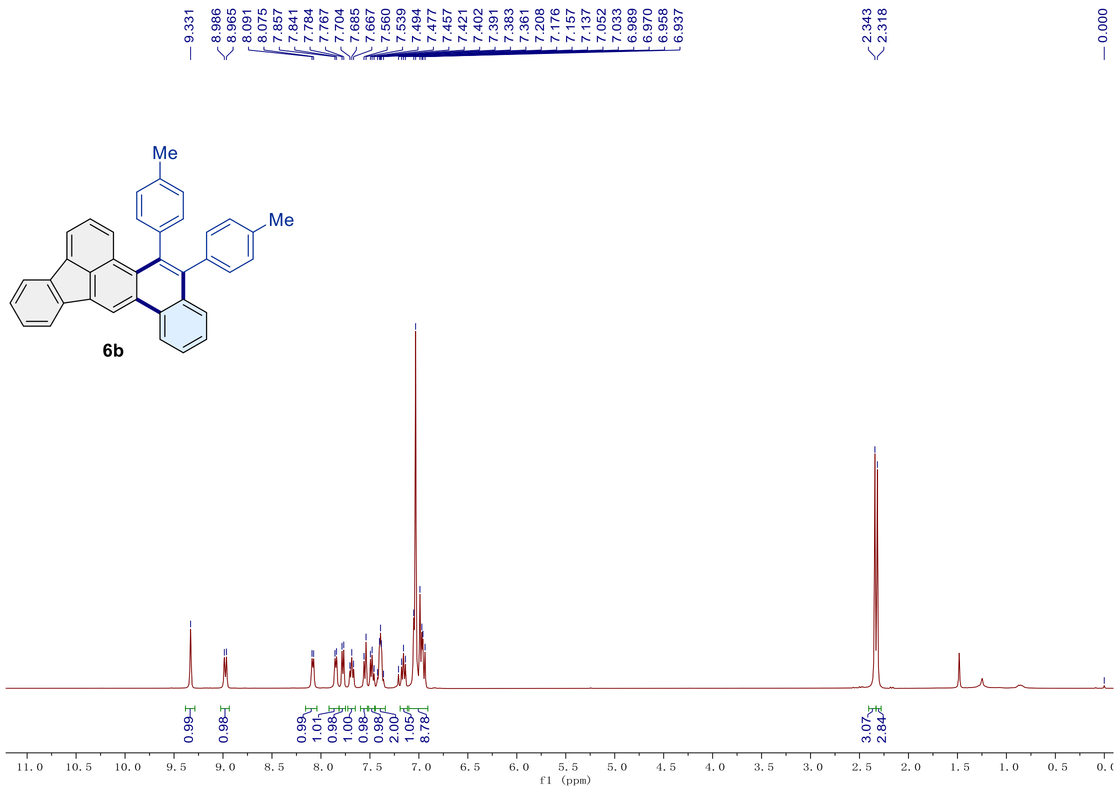
9.331
8.986
8.965
8.091
8.075
7.857
7.841
7.784
7.767
7.704
7.685
7.667
7.560
7.539
7.494
7.477
7.457
7.421
7.402
7.391
7.383
7.361
7.208
7.176
7.157
7.137
7.052
7.033
6.989
6.970
6.958
6.937
2.343
2.318
0.000
Me
Me
6b
0.99
0.98
0.99
1.01
0.98
1.00
0.98
0.98
2.00
1.05
8.78
3.07
2.84
f1 (ppm)

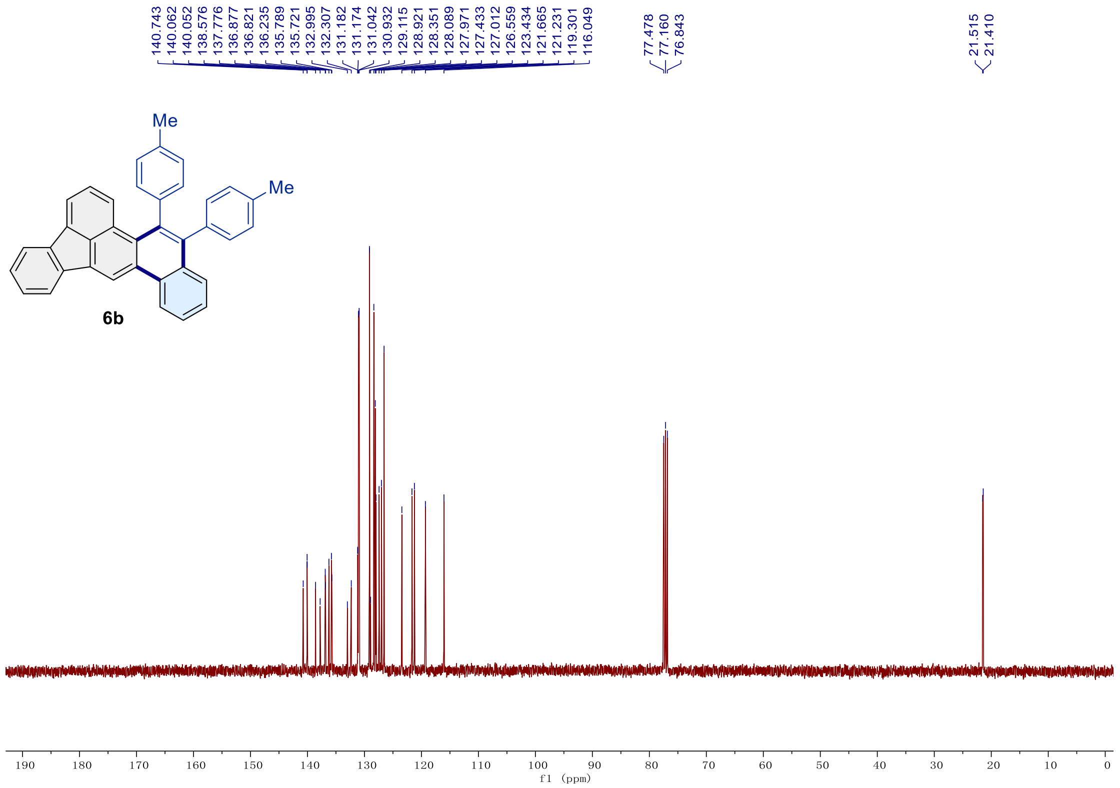
140.743
140.062
140.052
138.576
137.776
136.877
136.821
136.235
135.789
135.721
132.995
132.307
131.182
131.174
131.042
130.932
129.115
128.921
128.351
128.089
127.971
127.433
127.012
126.559
123.434
121.665
121.231
119.301
116.049
77.478
77.160
76.843
21.515
21.410
Me
Me
6b
f1 (ppm)

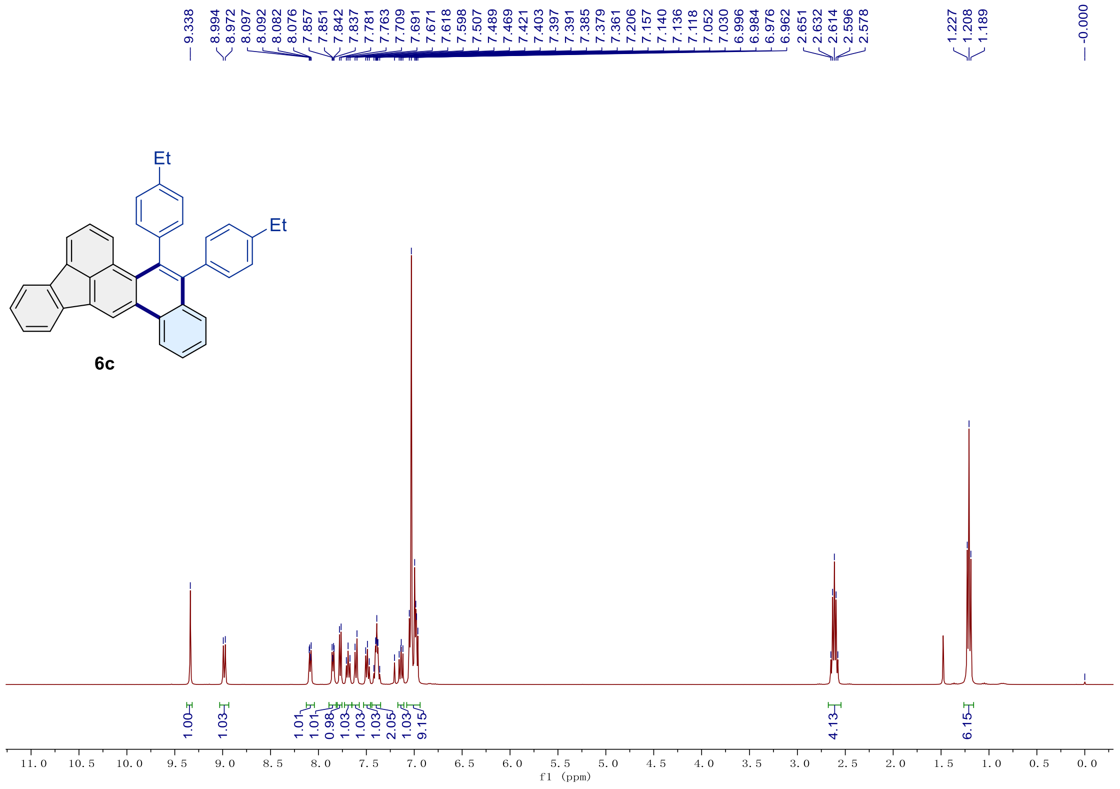
9.338
8.994
8.972
8.097
8.092
8.082
8.076
7.857
7.851
7.842
7.837
7.781
7.763
7.709
7.691
7.671
7.618
7.598
7.507
7.489
7.469
7.421
7.403
7.397
7.391
7.385
7.379
7.361
7.206
7.157
7.140
7.136
7.118
7.052
7.030
6.996
6.984
6.976
6.962
2.651
2.632
2.614
2.596
2.578
1.227
1.208
1.189
-0.000
Et
Et
6c
1.00
1.03
1.01
1.01
0.98
1.03
1.03
1.03
2.05
1.03
9.15
4.13
6.15
f1 (ppm)

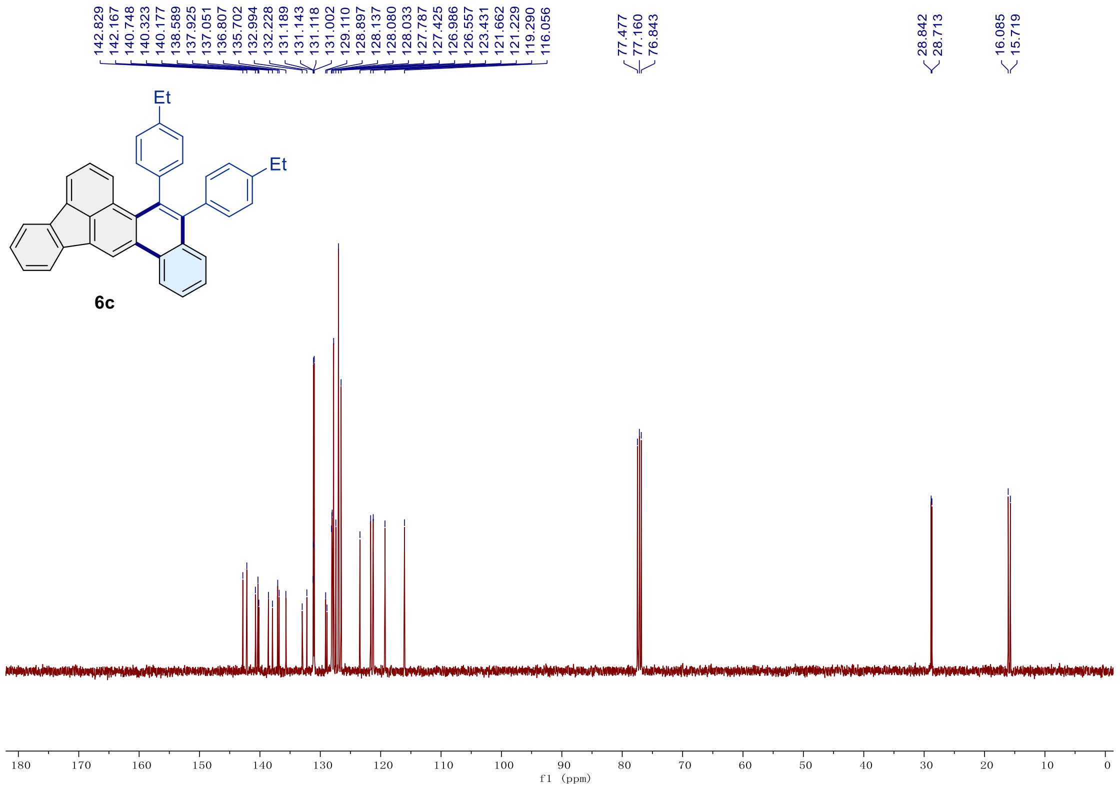
142.829
142.167
140.748
140.323
140.177
138.589
137.925
137.051
136.807
135.702
132.994
132.228
131.189
131.143
131.118
131.002
129.110
128.897
128.137
128.080
128.033
127.787
127.425
126.986
126.557
123.431
121.662
121.229
119.290
116.056
77.477
77.160
76.843
28.842
28.713
16.085
15.719
Et
Et
6c
f1 (ppm)

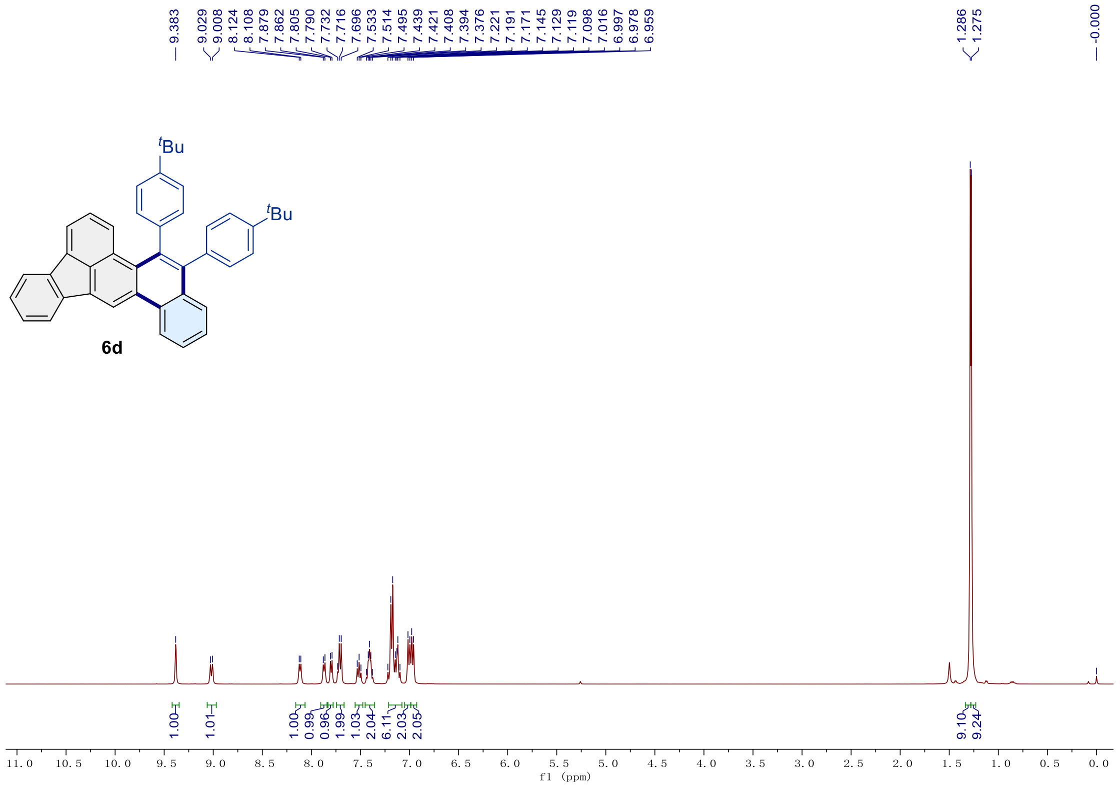

9.383
9.029
9.008
8.124
8.108
7.879
7.862
7.805
7.790
7.732
7.716
7.696
7.533
7.514
7.495
7.439
7.421
7.408
7.394
7.376
7.221
7.191
7.171
7.145
7.129
7.119
7.098
7.016
6.997
6.978
6.959
1.286
1.275
-0.000
tBu
tBu
1.00
1.01
1.00
0.99
0.96
1.99
1.03
2.04
6.11
2.03
2.05
9.10
9.24
f1 (ppm)

6d

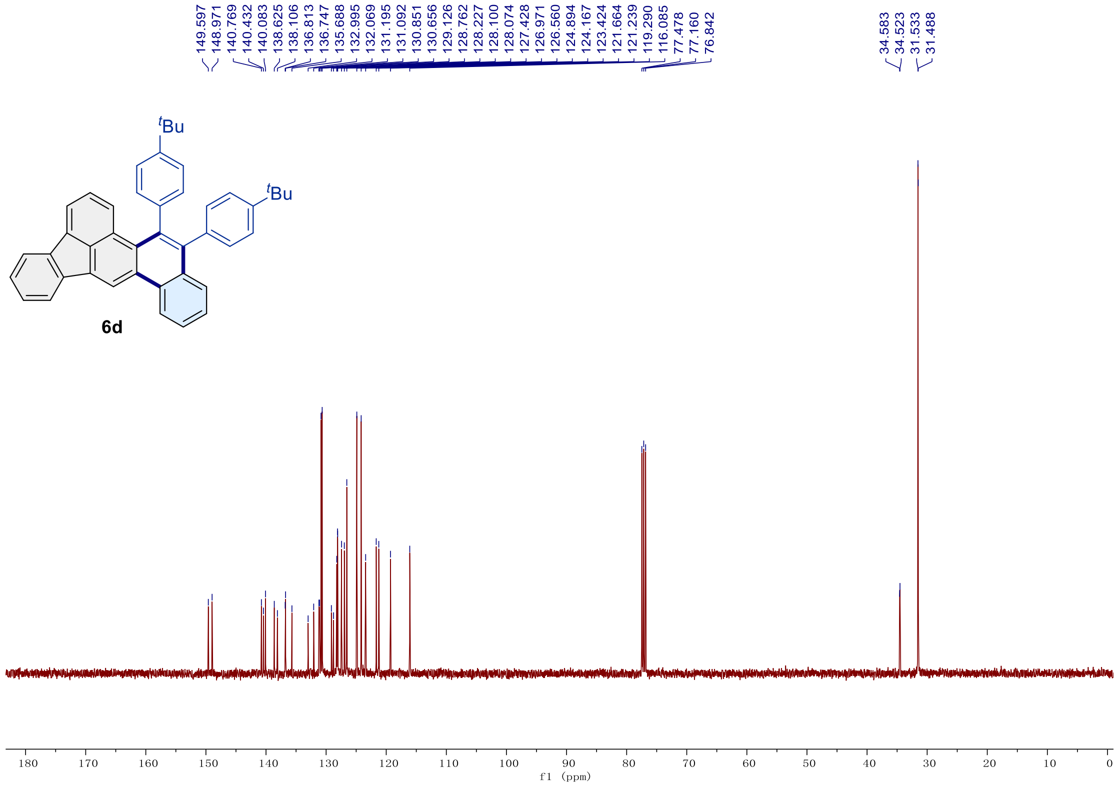

149.597
148.971
140.769
140.432
140.083
138.625
138.106
136.813
136.747
135.688
132.995
132.069
131.195
131.092
130.851
130.656
129.126
128.762
128.227
128.100
128.074
127.428
126.971
126.560
124.894
124.167
123.424
121.664
121.239
119.290
116.085
77.478
77.160
76.842
34.583
34.523
31.533
31.488
tBu
tBu
f1 (ppm)

6d

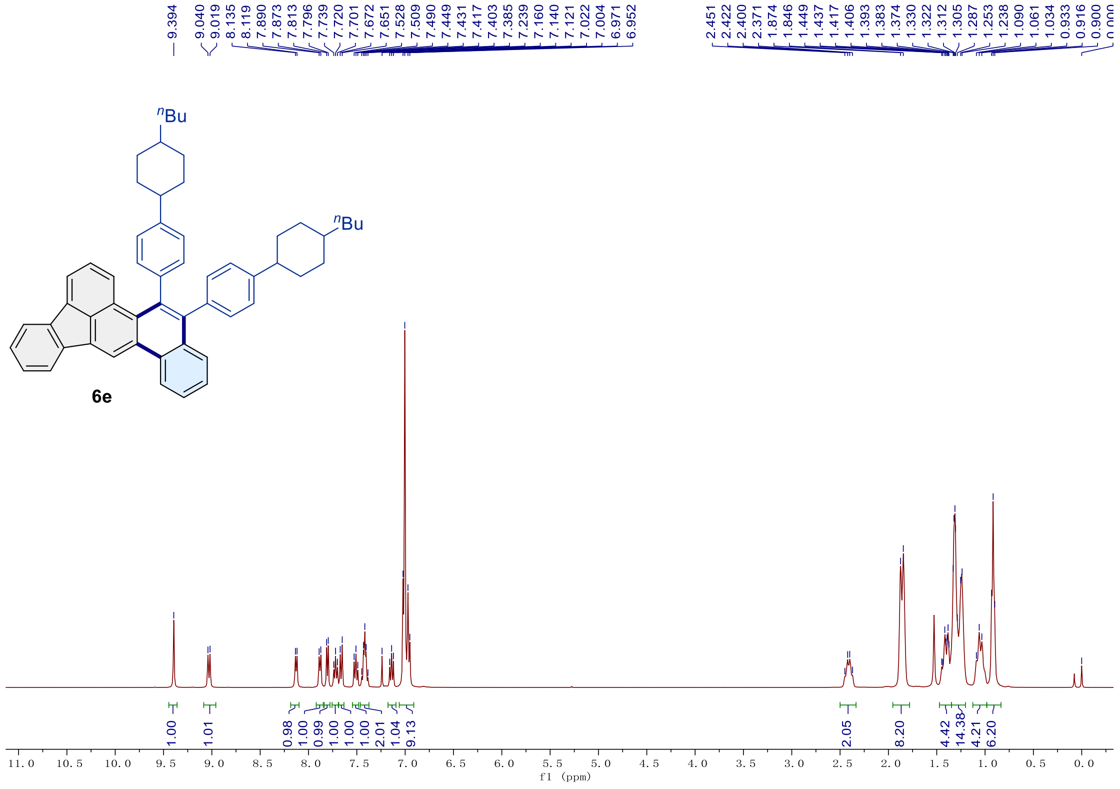
9.394
9.040
9.019
8.135
8.119
7.890
7.873
7.813
7.796
7.739
7.720
7.701
7.672
7.651
7.528
7.509
7.490
7.449
7.431
7.417
7.403
7.385
7.239
7.160
7.140
7.121
7.022
7.004
6.971
6.952
2.451
2.422
2.400
2.371
1.874
1.846
1.449
1.437
1.417
1.406
1.393
1.383
1.374
1.330
1.322
1.312
1.305
1.287
1.253
1.238
1.090
1.061
1.034
0.933
0.916
0.900
0.000
nBu
nBu
6e
1.00
1.01
0.98
1.00
0.99
1.00
1.00
2.01
1.04
9.13
2.05
8.20
4.42
14.38
4.21
6.20
f1 (ppm)

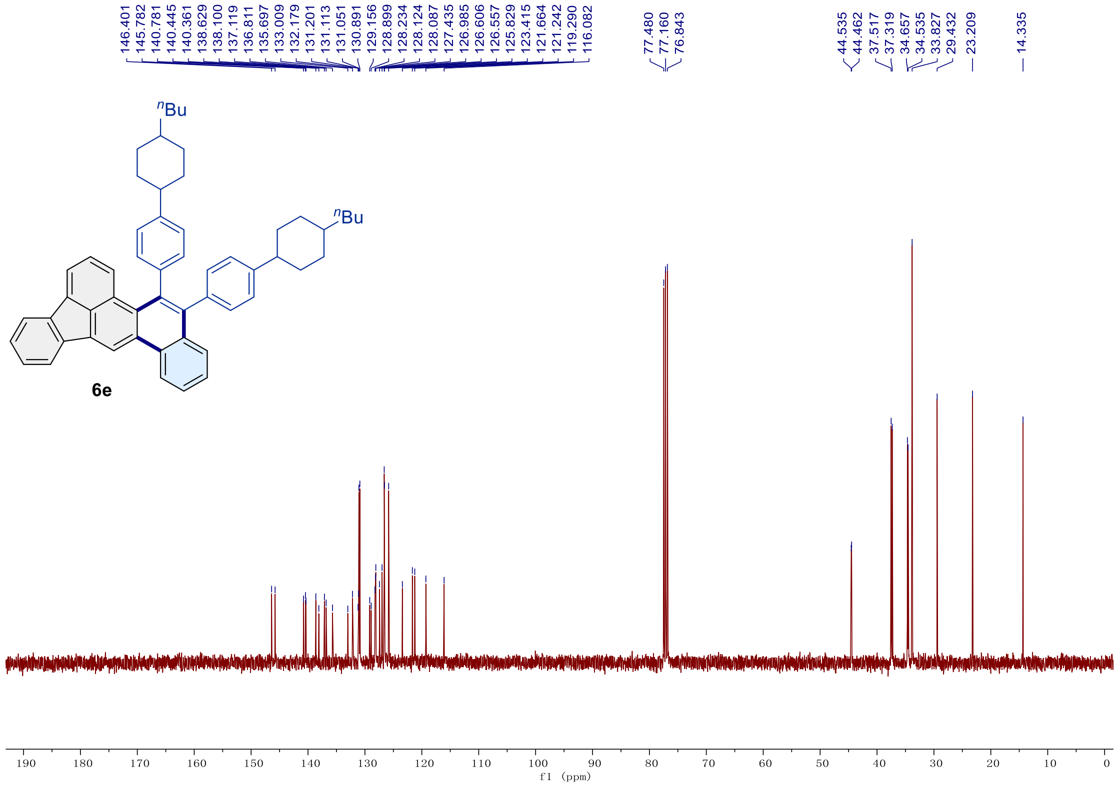
146.401
145.782
140.781
140.445
140.361
138.629
138.100
137.119
136.811
135.697
133.009
132.179
131.201
131.113
131.051
130.891
129.156
128.899
128.234
128.124
128.087
127.435
126.985
126.606
126.557
125.829
123.415
121.664
121.242
119.290
116.082
77.480
77.160
76.843
44.535
44.462
37.517
37.319
34.657
34.535
33.827
29.432
23.209
14.335
nBu
nBu
6e
f1 (ppm)

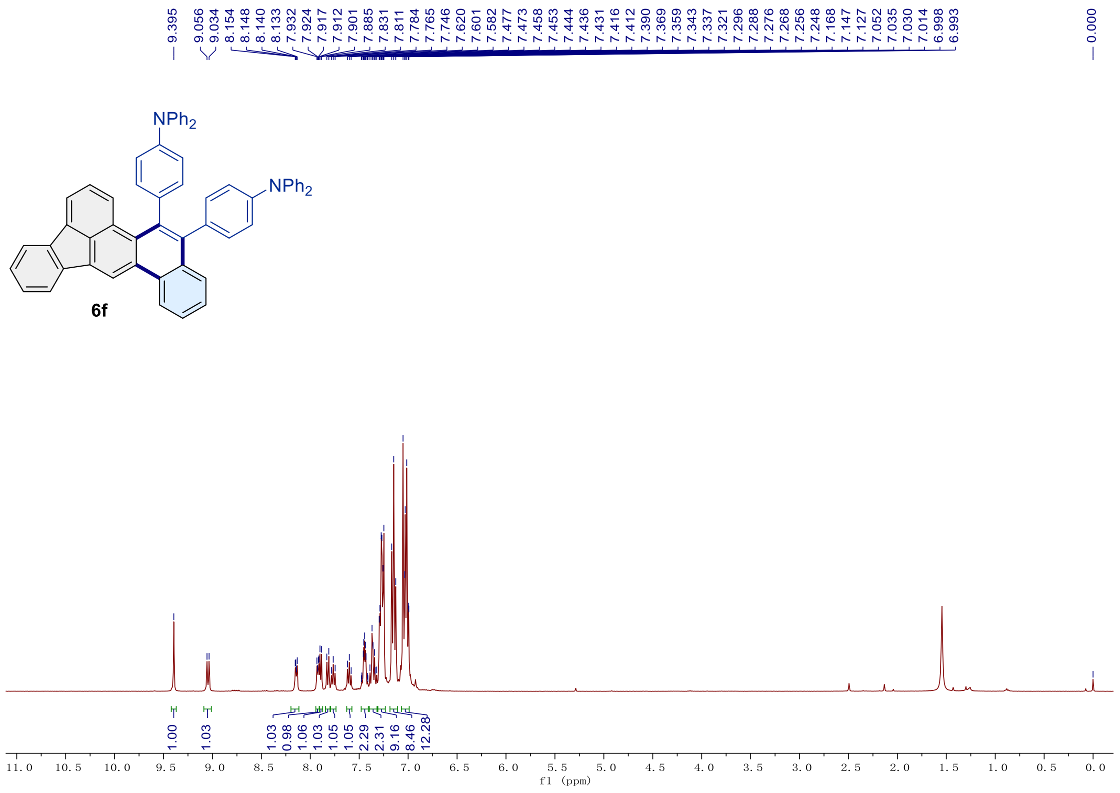
9.395
9.056
9.034
8.154
8.148
8.140
8.133
7.932
7.924
7.917
7.912
7.901
7.885
7.831
7.811
7.784
7.765
7.746
7.620
7.601
7.582
7.477
7.473
7.458
7.453
7.444
7.436
7.431
7.416
7.412
7.390
7.369
7.359
7.343
7.337
7.321
7.296
7.288
7.276
7.268
7.256
7.248
7.168
7.147
7.127
7.052
7.035
7.030
7.014
6.998
6.993
0.000
NPh2
NPh2
6f
1.00
1.03
1.03
0.98
1.06
1.03
1.05
1.05
2.29
2.31
9.16
8.46
12.28
f1 (ppm)

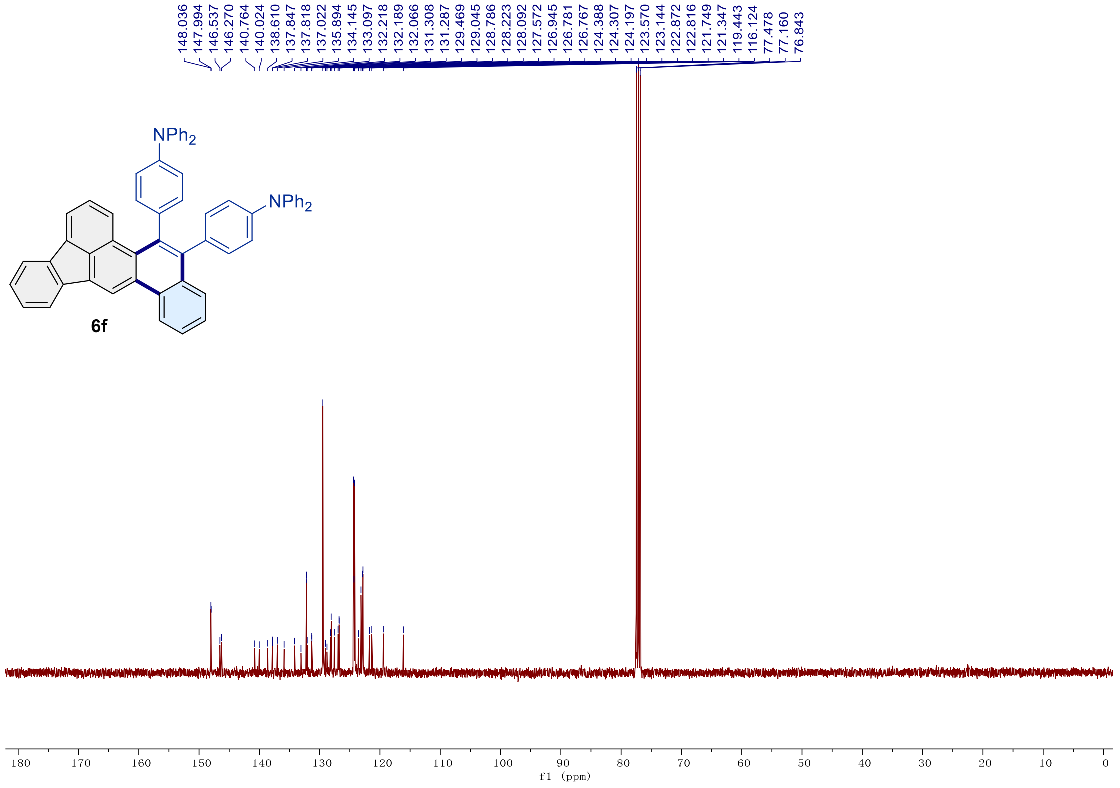
148.036
147.994
146.537
146.270
140.764
140.024
138.610
137.847
137.818
137.022
135.894
134.145
133.097
132.218
132.189
132.066
131.308
131.287
129.469
129.045
128.786
128.223
128.092
127.572
126.945
126.781
126.767
124.388
124.307
124.197
123.570
123.144
122.872
122.816
121.749
121.347
119.443
116.124
77.478
77.160
76.843
NPh2
NPh2
6f
f1 (ppm)

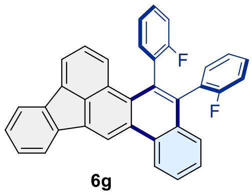
F
F

6g

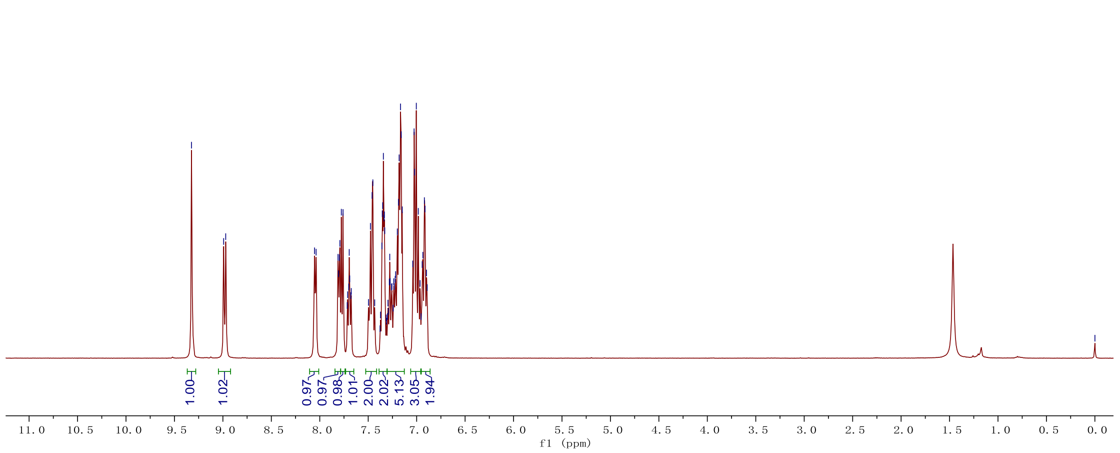
9.324 8.992 8.971 8.054 8.038 7.812 7.806 7.798 7.792 7.777 7.760 7.717 7.712 7.701 7.696 7.691 7.680 7.675 7.497 7.477 7.460 7.452 7.433 7.378 7.373 7.359 7.355 7.351 7.344 7.337 7.333 7.329 7.314 7.310 7.301 7.296 7.292 7.282 7.278 7.273 7.259 7.255 7.242 7.236 7.232 7.222 7.217 7.200 7.188 7.181 7.167 7.159 7.149 7.040 7.026 7.021 7.004 6.983 6.964 6.954 6.943 6.936 6.920 6.914 6.899 6.891 0.000
1.00 1.02 0.97 0.97 0.98 1.01 2.00 2.02 5.13 3.05 1.94
f1 (ppm)

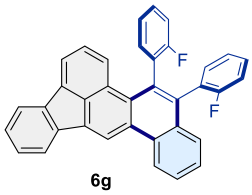
F
F

6g

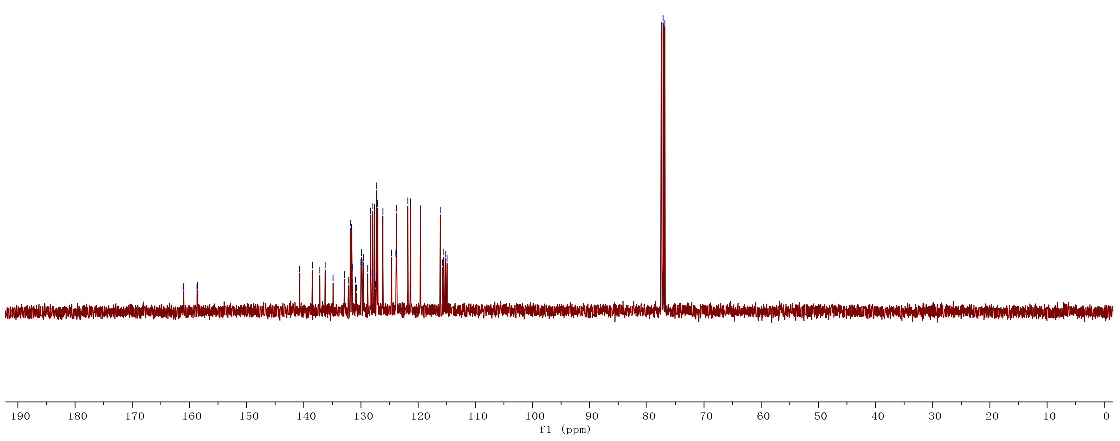
161.106 160.996 158.675 158.566 140.729 138.505 137.192 136.267 134.883 132.882 132.224 131.871 131.642 131.534 130.979 130.805 130.005 129.928 129.661 129.583 128.813 128.317 128.253 127.918 127.605 127.462 127.285 127.243 127.220 127.083 126.161 124.659 124.625 123.889 123.853 123.780 121.796 121.344 119.627 116.142 115.721 115.503 115.161 114.941 77.477 77.160 76.842
f1 (ppm)

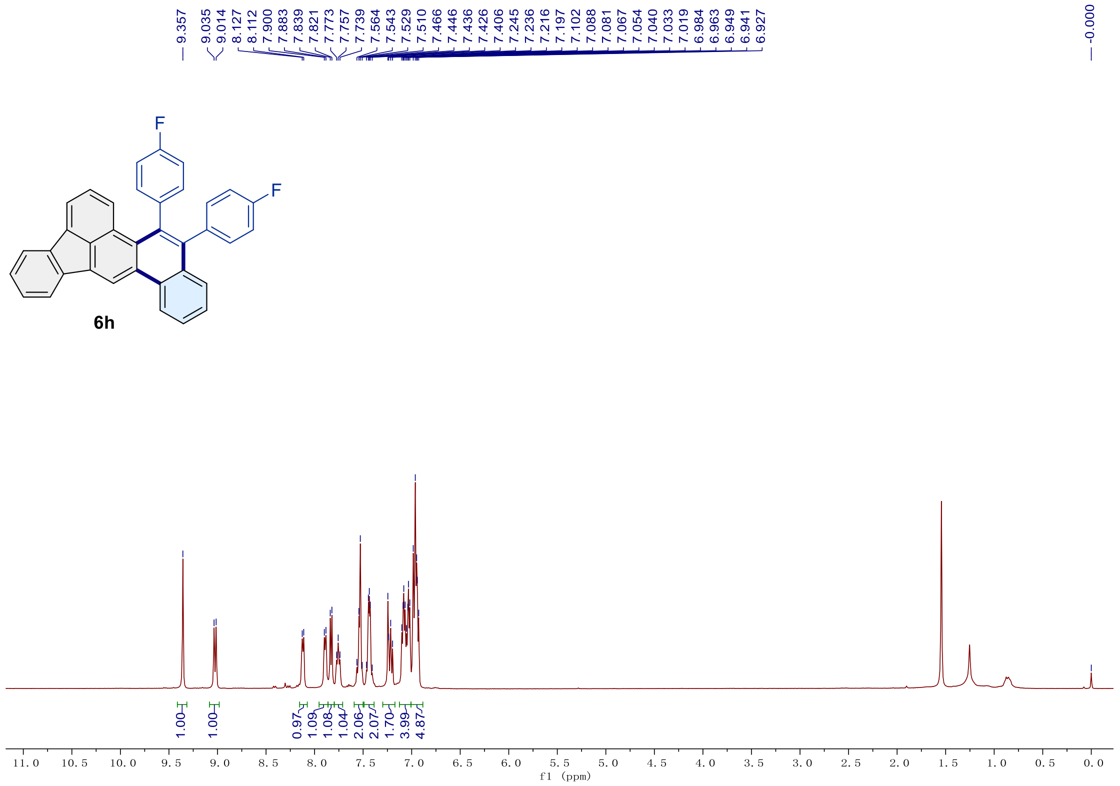
9.357
9.035
9.014
8.127
8.112
7.900
7.883
7.839
7.821
7.773
7.757
7.739
7.564
7.543
7.529
7.510
7.466
7.446
7.436
7.426
7.406
7.245
7.236
7.216
7.197
7.102
7.088
7.081
7.067
7.054
7.040
7.033
7.019
6.984
6.963
6.949
6.941
6.927
-0.000
F
F
6h
1.00
1.00
0.97
1.09
1.08
1.04
2.06
2.07
1.70
3.99
4.87
f1 (ppm)

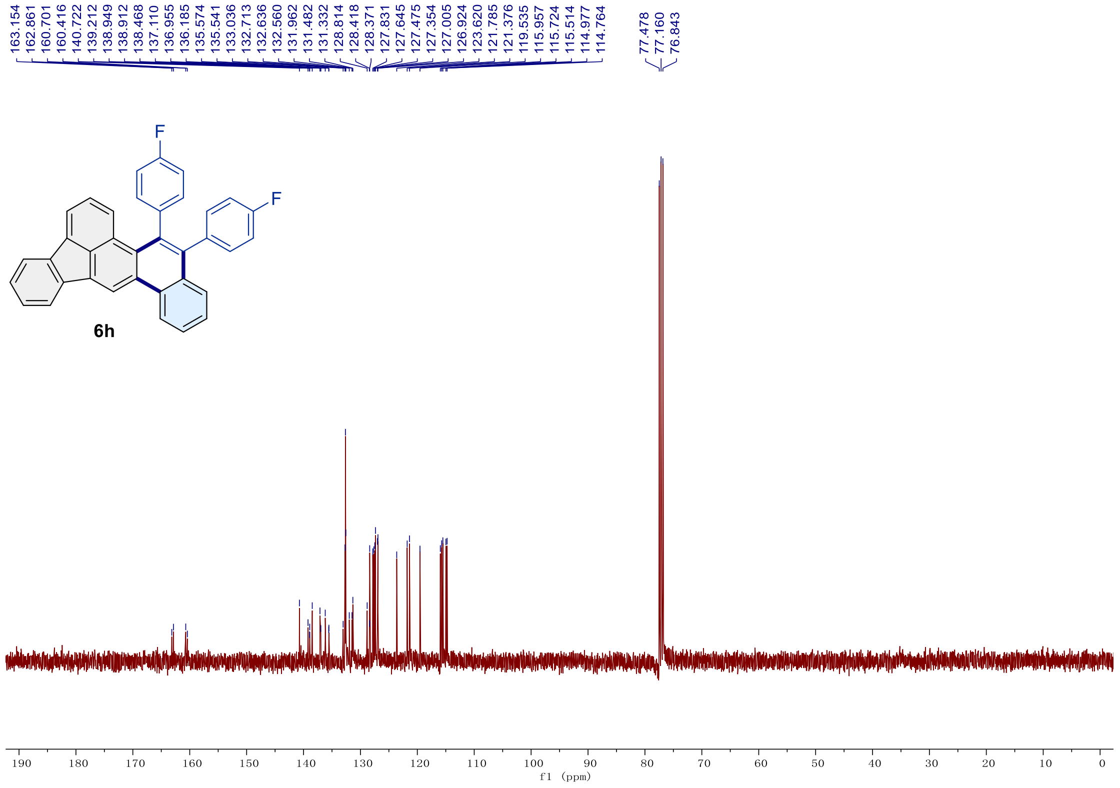
163.154
162.861
160.701
160.416
140.722
139.212
138.949
138.912
138.468
137.110
136.955
136.185
135.574
135.541
133.036
132.713
132.636
132.560
131.962
131.482
131.332
128.814
128.418
128.371
127.831
127.645
127.475
127.354
127.005
126.924
123.620
121.785
121.376
119.535
115.957
115.724
115.514
114.977
114.764
77.478
77.160
76.843
F
F
6h
f1 (ppm)

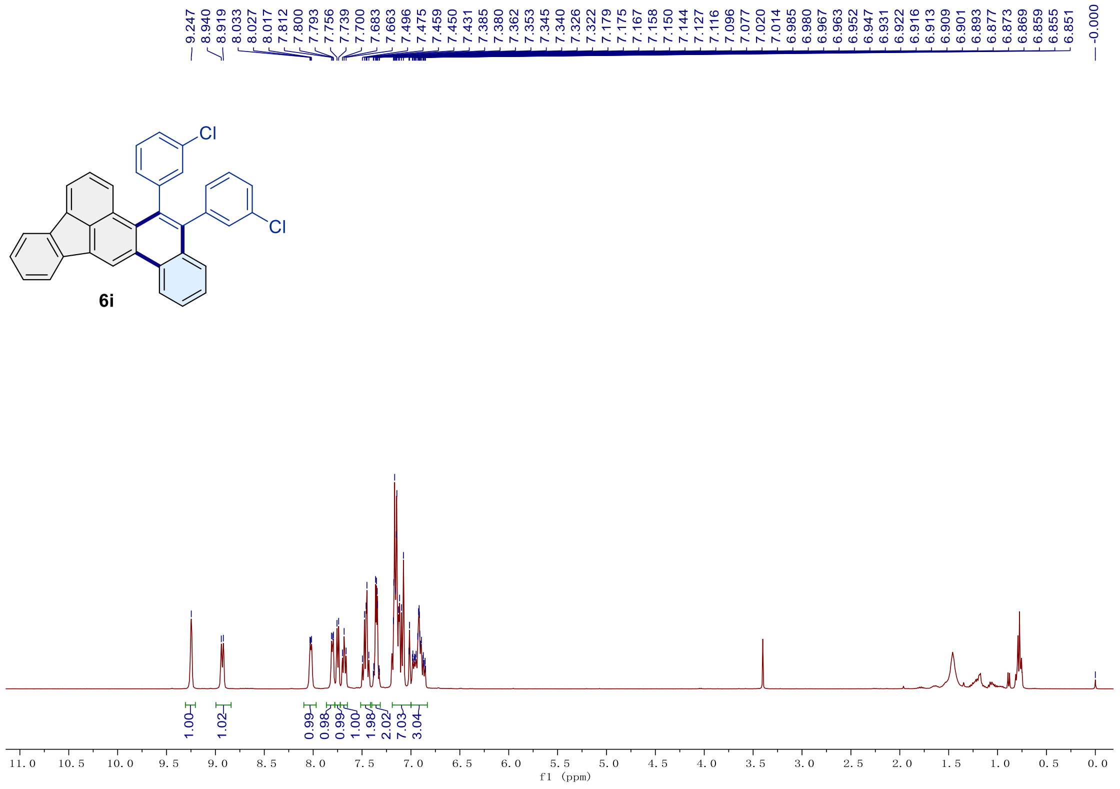
9.247
8.940
8.919
8.033
8.027
8.017
7.812
7.800
7.793
7.756
7.739
7.700
7.683
7.663
7.496
7.475
7.459
7.450
7.431
7.385
7.380
7.362
7.353
7.345
7.340
7.326
7.322
7.179
7.175
7.167
7.158
7.150
7.144
7.127
7.116
7.096
7.077
7.020
7.014
6.985
6.980
6.967
6.963
6.952
6.947
6.931
6.922
6.916
6.913
6.909
6.901
6.893
6.877
6.873
6.869
6.859
6.855
6.851
-0.000
Cl
Cl
6i
1.00
1.02
0.99
0.98
0.99
1.00
1.98
2.02
7.03
3.04
11.0 10.5 10.0 9.5 9.0 8.5 8.0 7.5 7.0 6.5 6.0 5.5 5.0 4.5 4.0 3.5 3.0 2.5 2.0 1.5 1.0 0.5 0.0
f1 (ppm)

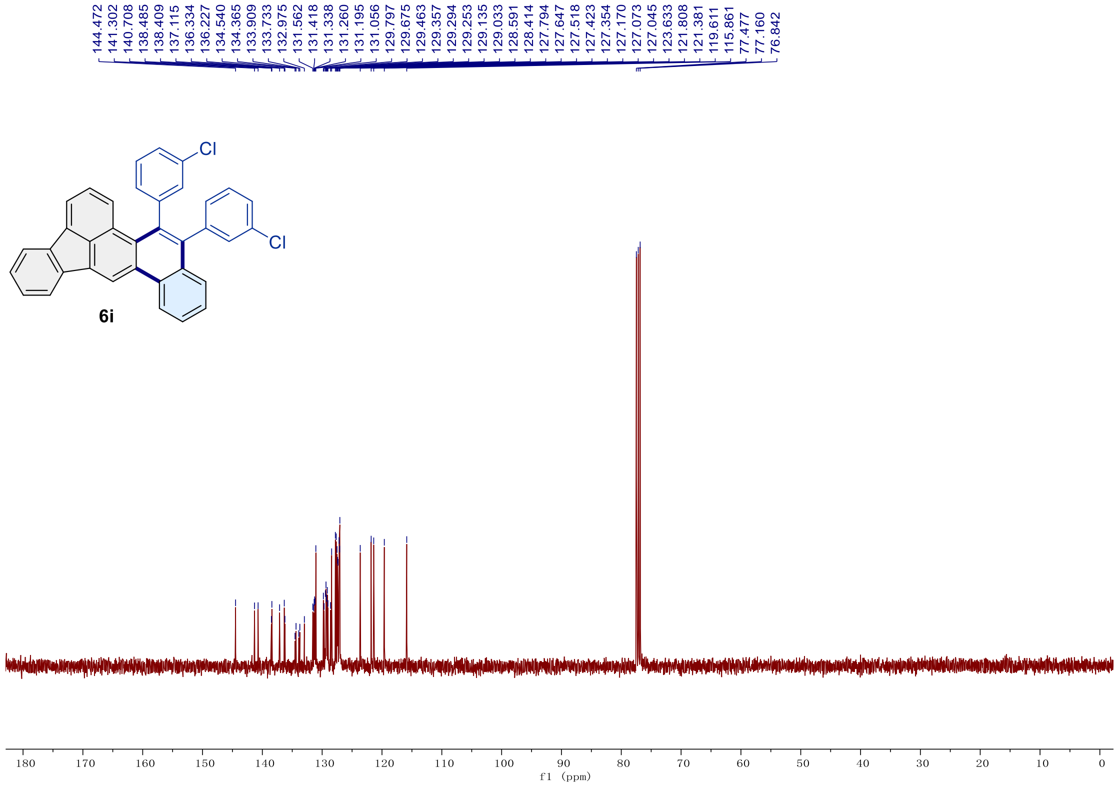
144.472
141.302
140.708
138.485
138.409
137.115
136.334
136.227
134.540
134.365
133.909
133.733
132.975
131.562
131.418
131.338
131.260
131.195
131.056
129.797
129.675
129.463
129.357
129.294
129.253
129.135
129.033
128.591
128.414
127.794
127.647
127.518
127.423
127.354
127.170
127.073
127.045
123.633
121.808
121.381
119.611
115.861
77.477
77.160
76.842
Cl
Cl
6i
180 170 160 150 140 130 120 110 100 90 80 70 60 50 40 30 20 10 0
f1 (ppm)

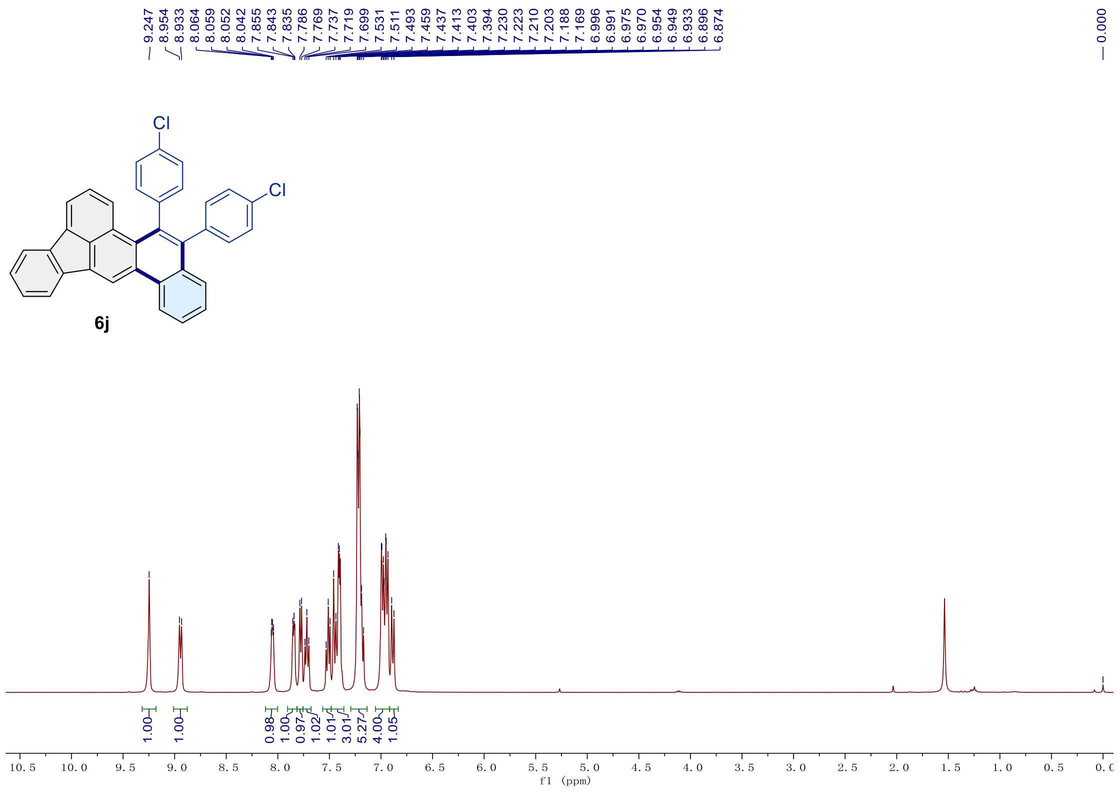
9.247
8.954
8.933
8.064
8.059
8.052
8.042
7.855
7.843
7.835
7.786
7.769
7.737
7.719
7.699
7.531
7.511
7.493
7.459
7.437
7.413
7.403
7.394
7.230
7.223
7.210
7.203
7.188
7.169
6.996
6.991
6.975
6.970
6.954
6.949
6.933
6.896
6.874
0.000
Cl
Cl
6j
1.00
1.00
0.98
1.00
0.97
1.02
1.01
3.01
5.27
4.00
1.05
f1 (ppm)

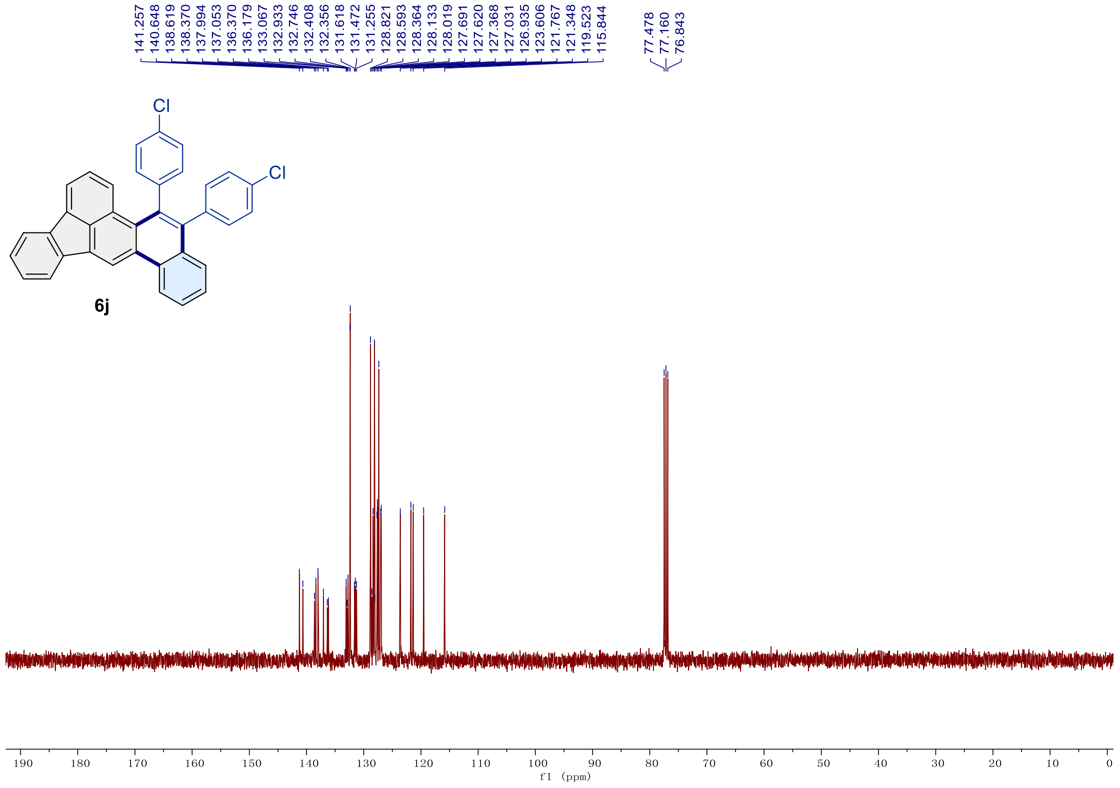
141.257
140.648
138.619
138.370
137.994
137.053
136.370
136.179
133.067
132.933
132.746
132.408
132.356
131.618
131.472
131.255
128.821
128.593
128.364
128.133
128.019
127.691
127.620
127.368
127.031
126.935
123.606
121.767
121.348
119.523
115.844
77.478
77.160
76.843
Cl
Cl
6j
f1 (ppm)

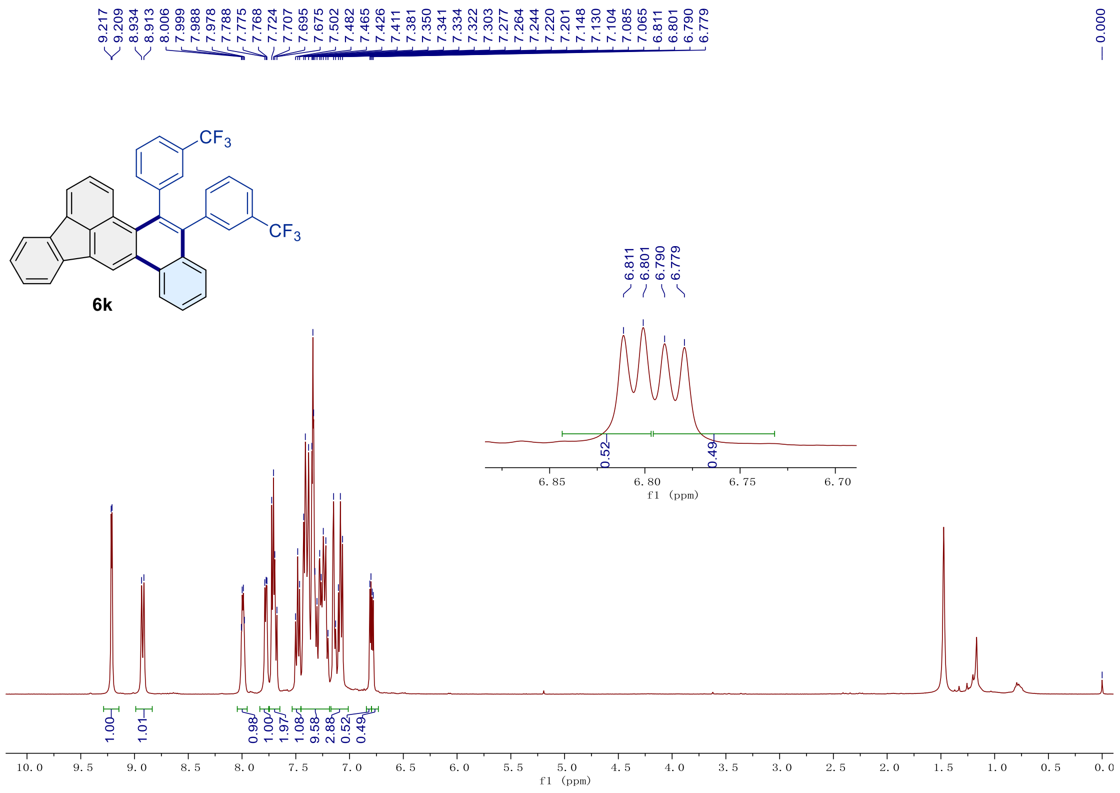

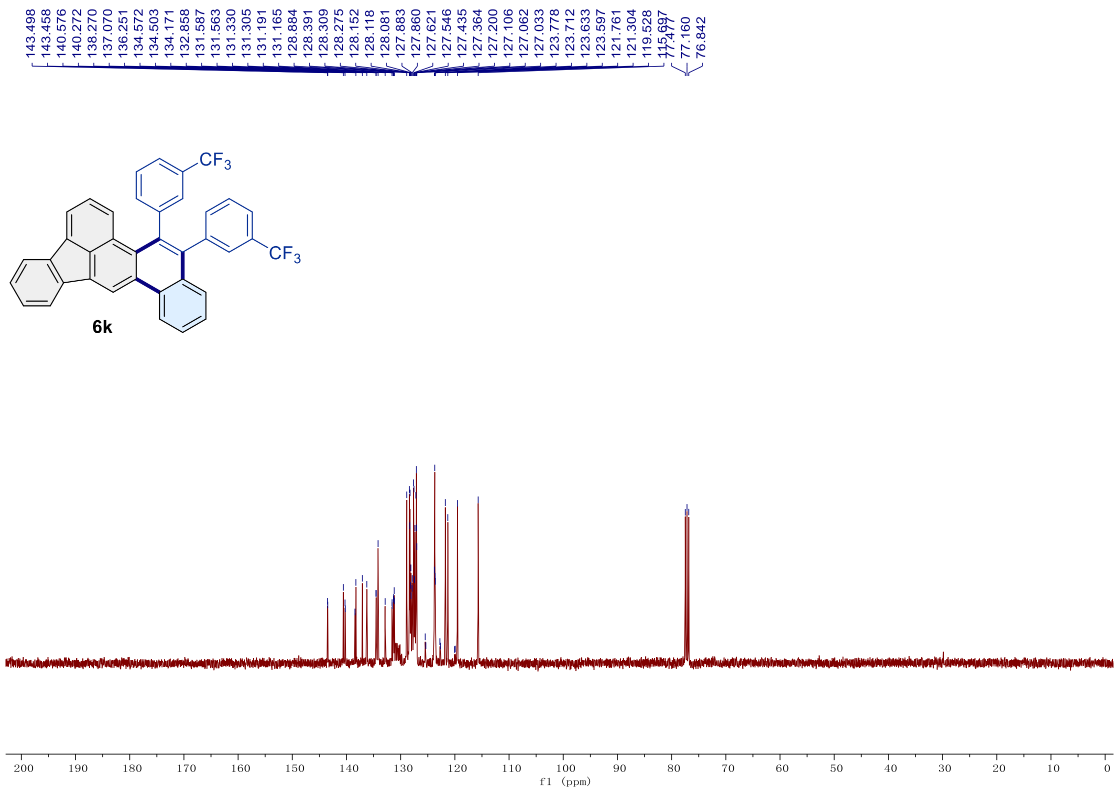

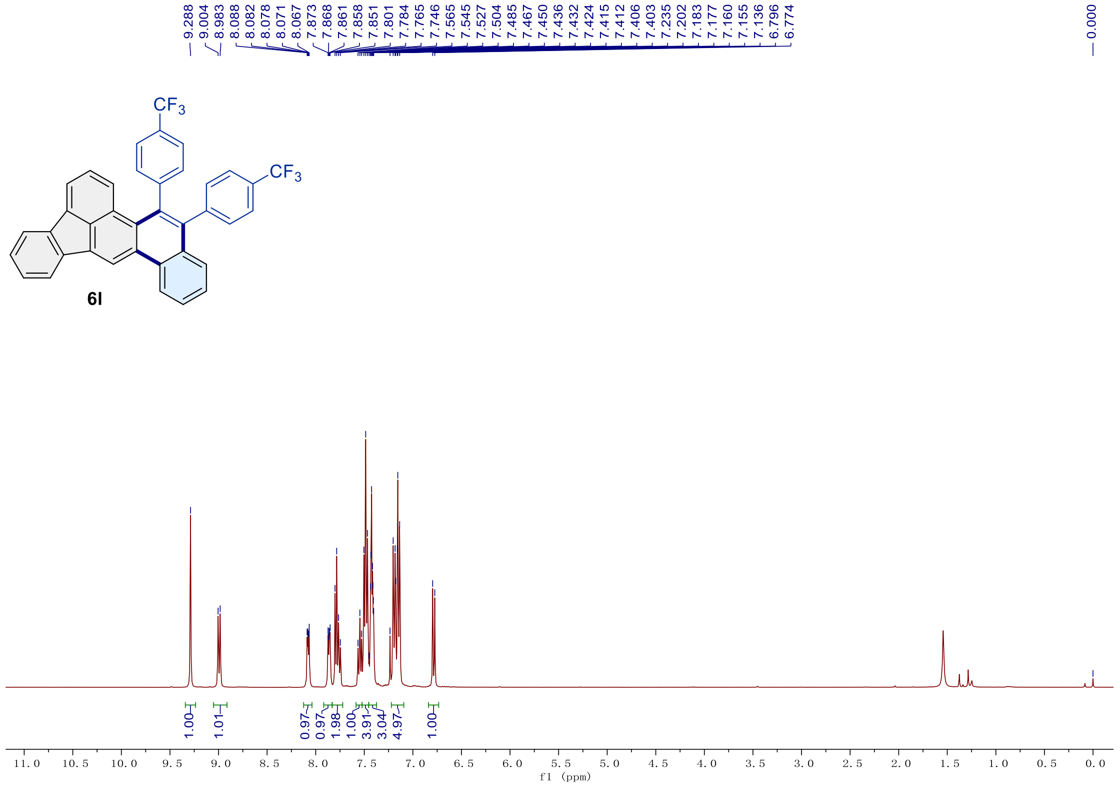

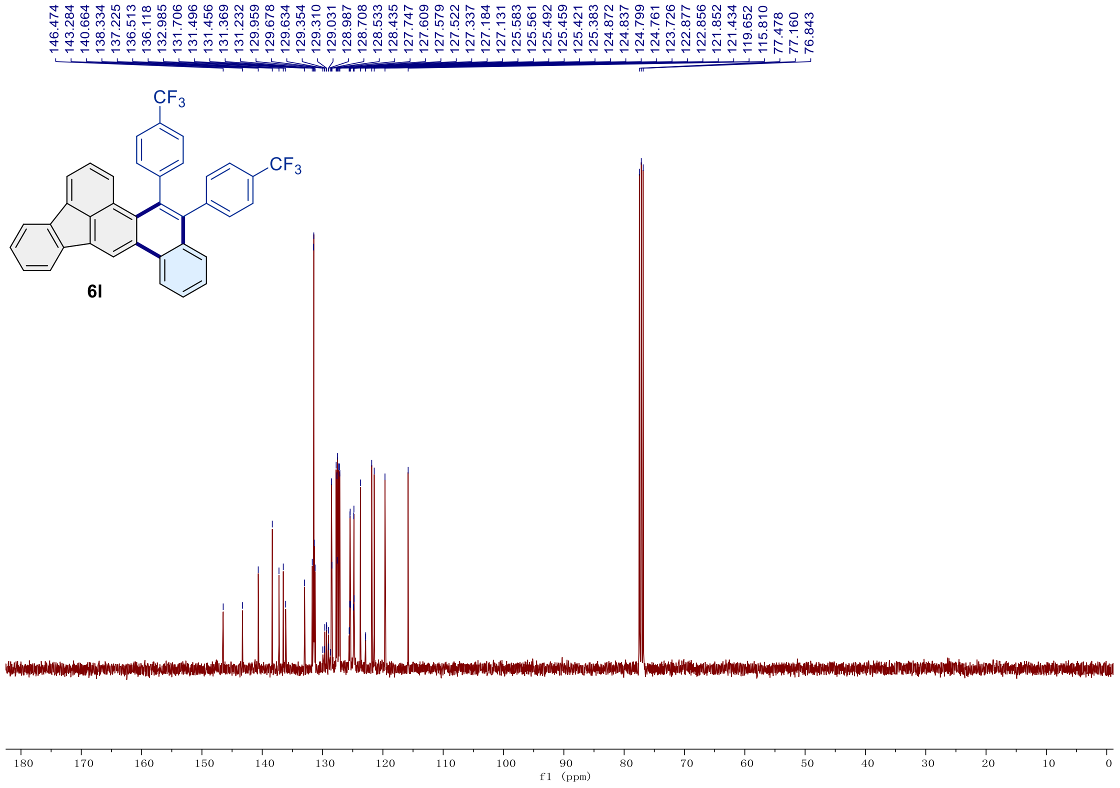

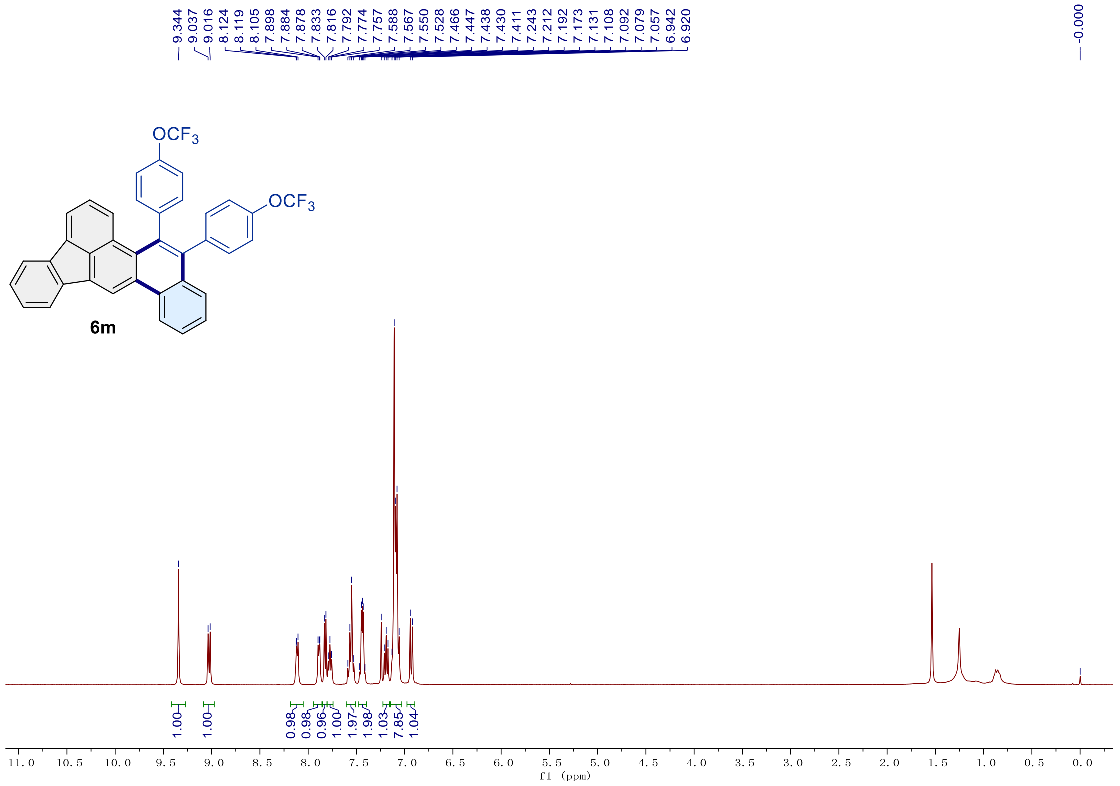
9.344
9.037
9.016
8.124
8.119
8.105
7.898
7.884
7.878
7.833
7.816
7.792
7.774
7.757
7.588
7.567
7.550
7.528
7.466
7.447
7.438
7.430
7.411
7.243
7.212
7.192
7.173
7.131
7.108
7.092
7.079
7.057
6.942
6.920
-0.000
OCF_3
OCF_3
6m
1.00
1.00
0.98
0.98
0.96
1.00
1.97
1.98
1.03
7.85
1.04
f1 (ppm)

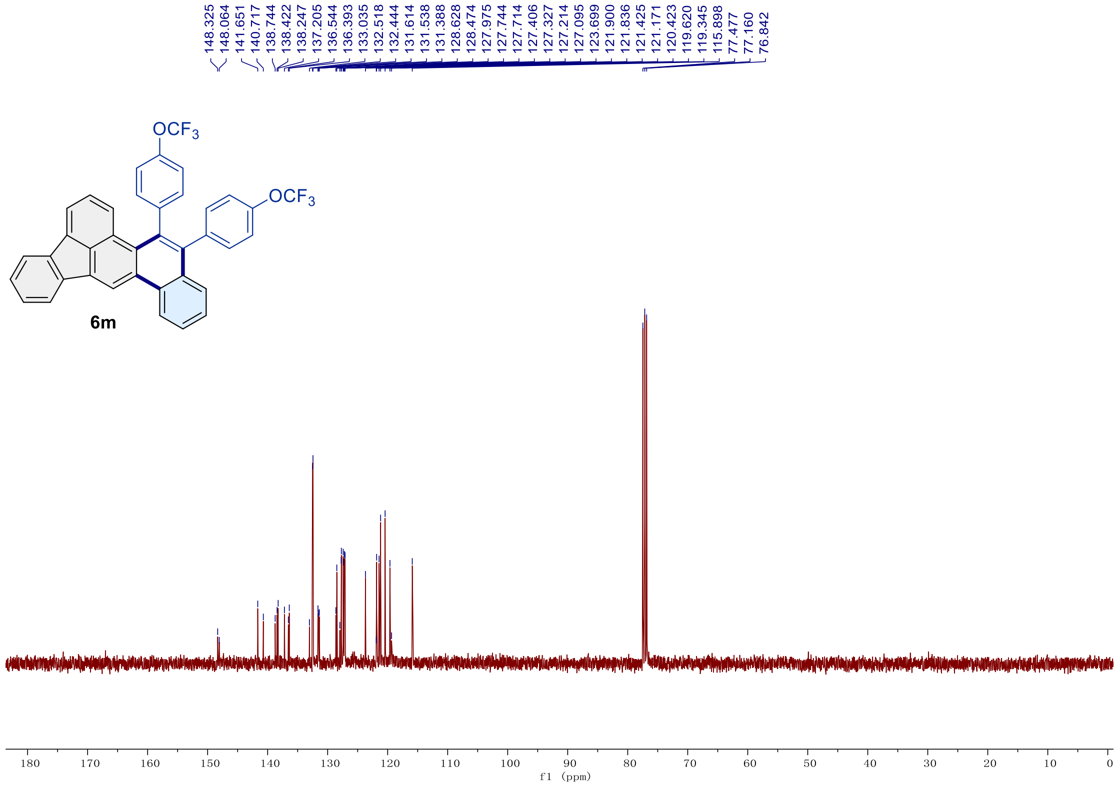
148.325
148.064
141.651
140.717
138.744
138.422
138.247
137.205
136.544
136.393
133.035
132.518
132.444
131.614
131.538
131.388
128.628
128.474
127.975
127.744
127.714
127.406
127.327
127.214
127.095
123.699
121.900
121.836
121.425
121.171
120.423
119.620
119.345
115.898
77.477
77.160
76.842
OCF_3
OCF_3
6m
f1 (ppm)

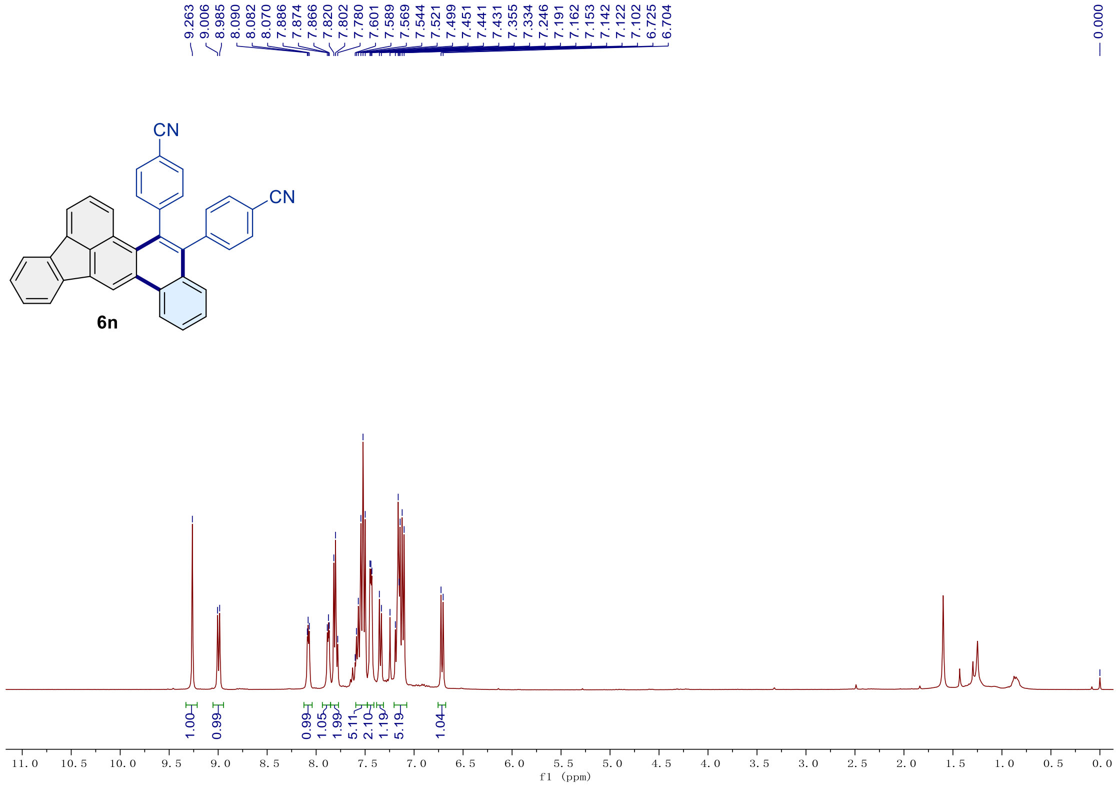
9.263
9.006
8.985
8.090
8.082
8.070
7.886
7.874
7.866
7.820
7.802
7.780
7.601
7.589
7.569
7.544
7.521
7.499
7.451
7.441
7.431
7.355
7.334
7.246
7.191
7.162
7.153
7.142
7.122
7.102
6.725
6.704
0.000
CN
CN
6n
1.00
0.99
0.99
1.05
1.99
5.11
2.10
1.19
5.19
1.04
f1 (ppm)

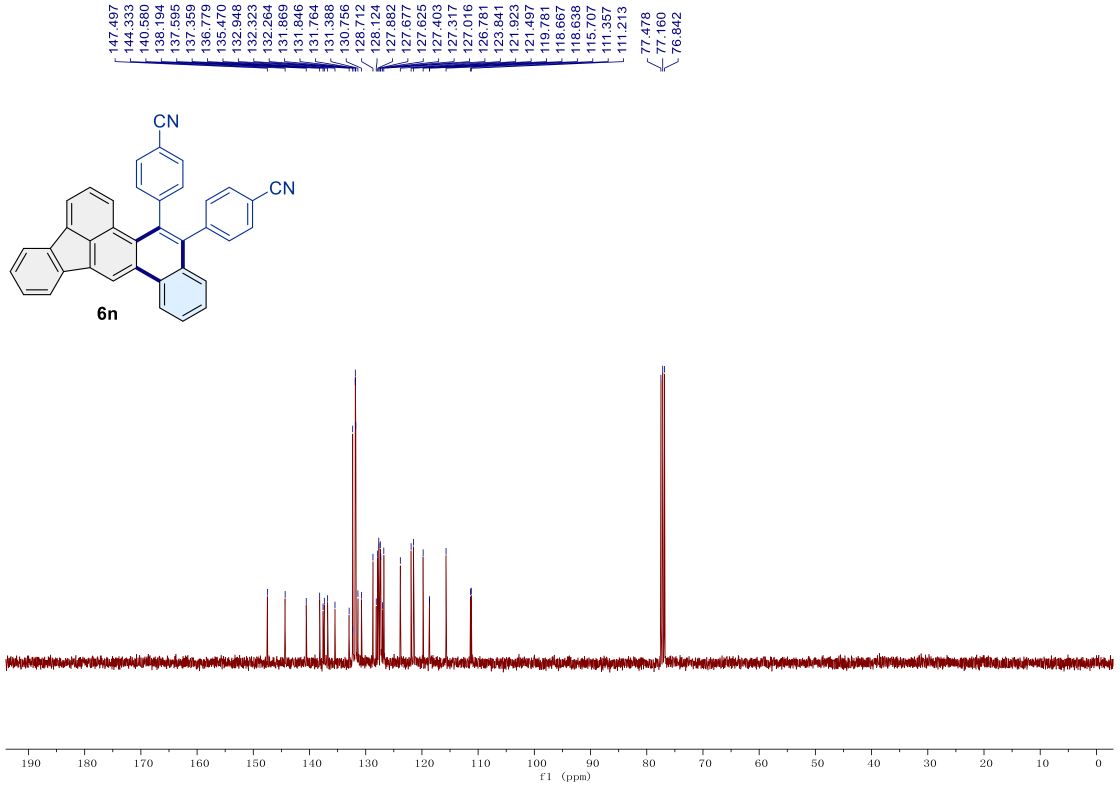
147.497
144.333
140.580
138.194
137.595
137.359
136.779
135.470
132.948
132.323
132.264
131.869
131.846
131.764
131.388
130.756
128.712
128.124
127.882
127.677
127.625
127.403
127.317
127.016
126.781
123.841
121.923
121.497
119.781
118.667
118.638
115.707
111.357
111.213
77.478
77.160
76.842
CN
CN
6n
f1 (ppm)

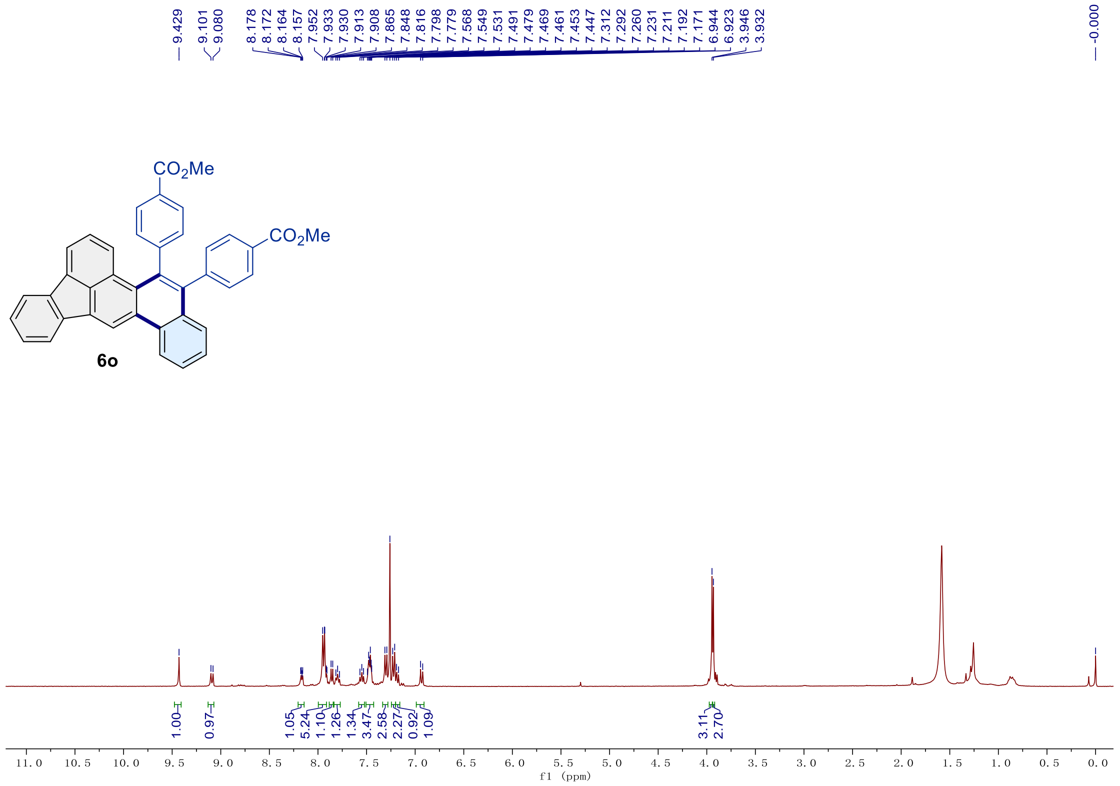
9.429
9.101
9.080
8.178
8.172
8.164
8.157
7.952
7.933
7.930
7.913
7.908
7.865
7.848
7.816
7.798
7.779
7.568
7.549
7.531
7.491
7.479
7.469
7.461
7.453
7.447
7.312
7.292
7.260
7.231
7.211
7.192
7.171
6.944
6.923
3.946
3.932
-0.000
CO_2Me
CO_2Me
6o
1.00
0.97
1.05
5.24
1.10
1.26
1.34
3.47
2.58
2.27
0.92
1.09
3.11
2.70
f1 (ppm)

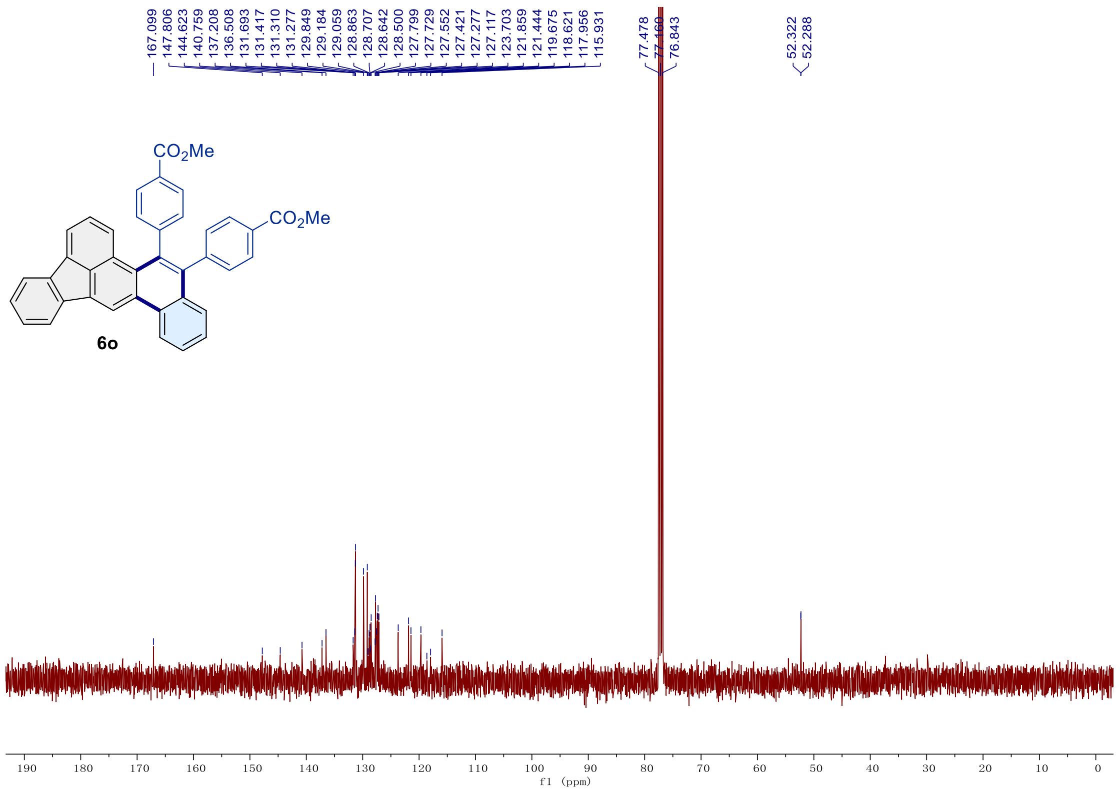
167.099
147.806
144.623
140.759
137.208
136.508
131.693
131.417
131.310
131.277
129.849
129.184
129.059
128.863
128.707
128.642
128.500
127.799
127.729
127.552
127.421
127.277
127.117
123.703
121.859
121.444
119.675
118.621
117.956
115.931
77.478
77.160
76.843
52.322
52.288
CO_2Me
CO_2Me
6o
f1 (ppm)

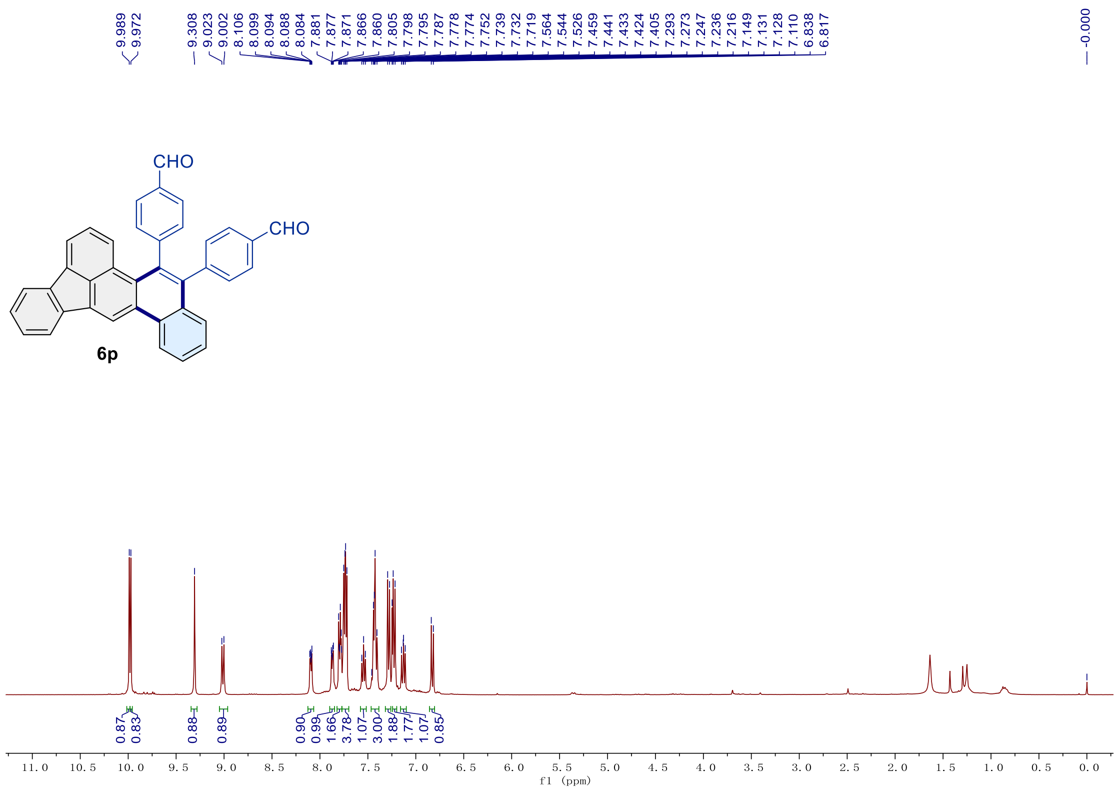

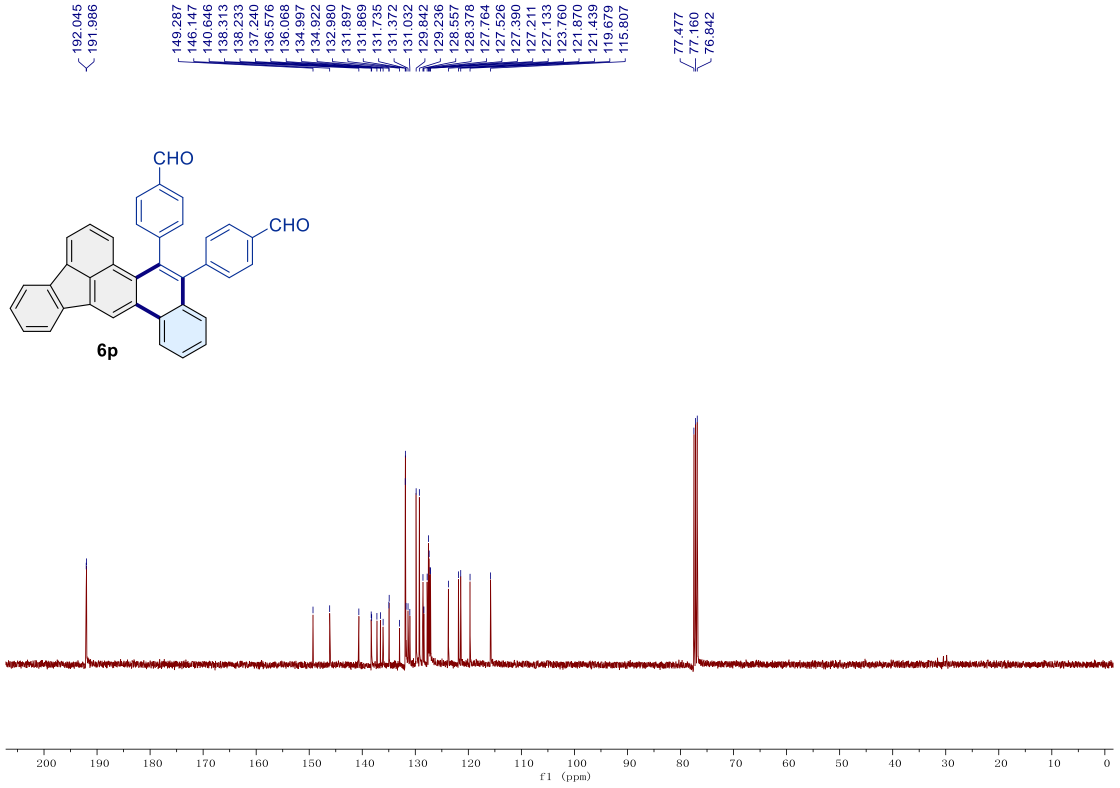

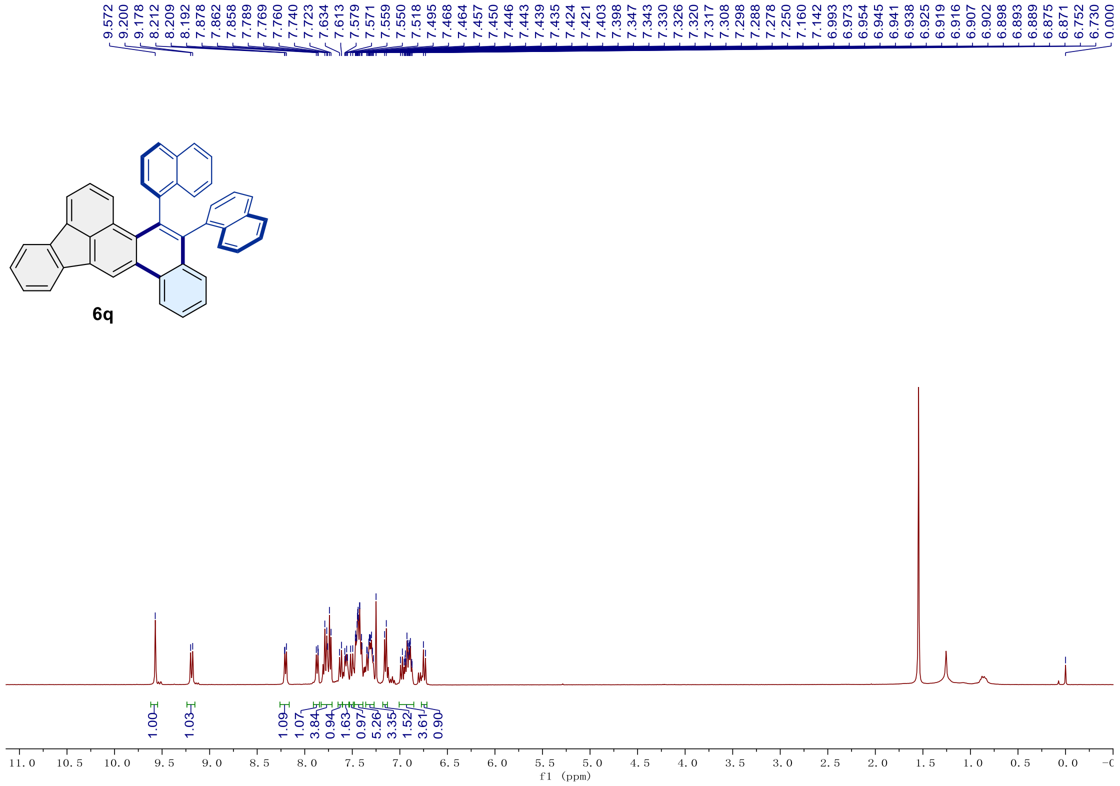
9.572
9.200
9.178
8.212
8.209
8.192
7.878
7.862
7.858
7.789
7.769
7.760
7.740
7.723
7.634
7.613
7.579
7.571
7.559
7.550
7.518
7.495
7.468
7.464
7.457
7.450
7.446
7.443
7.439
7.435
7.424
7.421
7.403
7.398
7.347
7.343
7.330
7.326
7.320
7.317
7.308
7.298
7.288
7.278
7.250
7.160
7.142
6.993
6.973
6.954
6.945
6.941
6.938
6.925
6.919
6.916
6.907
6.902
6.898
6.893
6.889
6.875
6.871
6.752
6.730
0.000
1.00
1.03
1.09
1.07
3.84
0.94
1.63
0.97
5.26
3.35
1.52
3.61
0.90
11.0 10.5 10.0 9.5 9.0 8.5 8.0 7.5 7.0 6.5 6.0 5.5 5.0 4.5 4.0 3.5 3.0 2.5 2.0 1.5 1.0 0.5 0.0
f1 (ppm)

6q

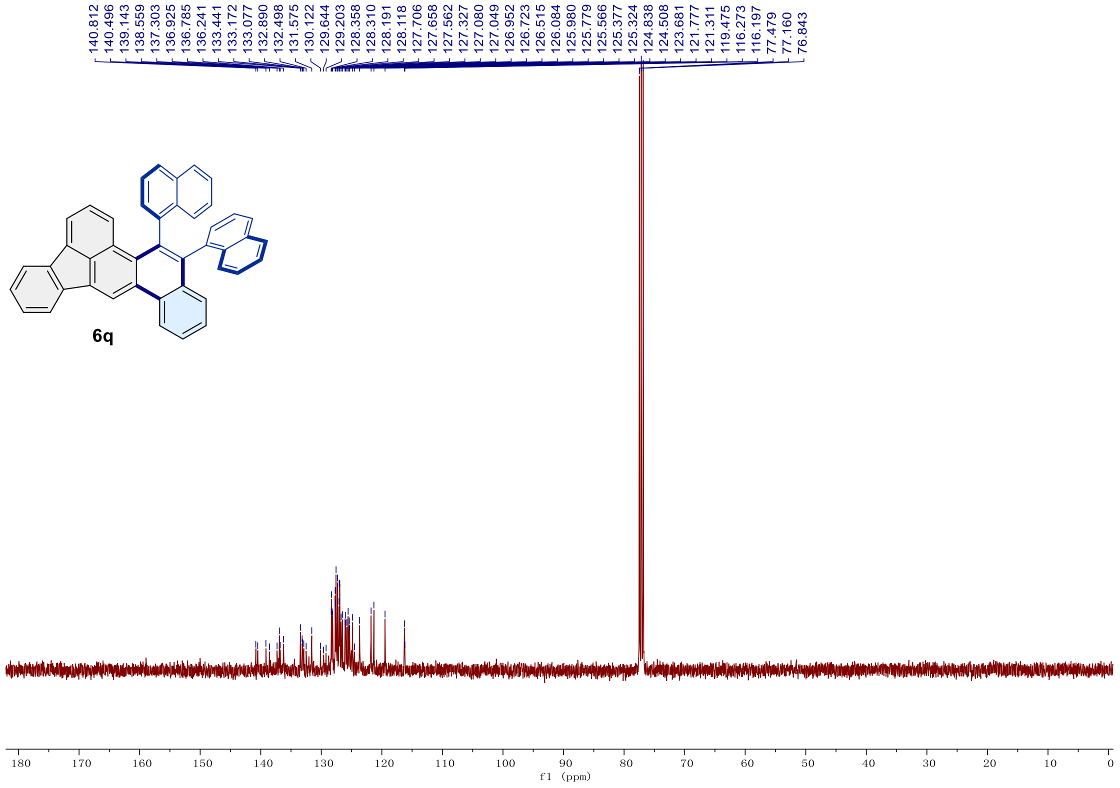
140.812
140.496
139.143
138.559
137.303
136.925
136.785
136.241
133.441
133.172
133.077
132.890
132.498
131.575
130.122
129.644
129.203
128.358
128.310
128.191
128.118
127.706
127.658
127.562
127.327
127.080
127.049
126.952
126.723
126.515
126.084
125.980
125.779
125.566
125.377
125.324
124.838
124.508
123.681
121.777
121.311
119.475
116.273
116.197
77.479
77.160
76.843
180 170 160 150 140 130 120 110 100 90 80 70 60 50 40 30 20 10 0
f1 (ppm)

6q

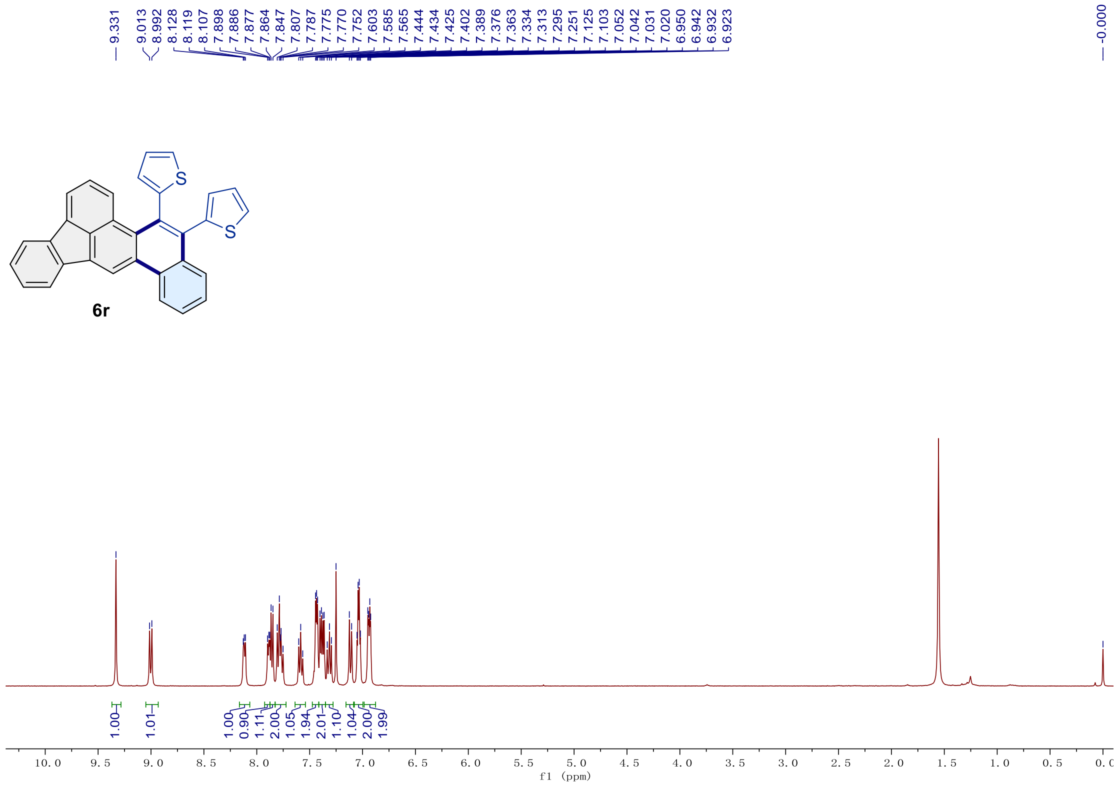
9.331
9.013
8.992
8.128
8.119
8.107
7.898
7.886
7.877
7.864
7.847
7.807
7.787
7.775
7.770
7.752
7.603
7.585
7.565
7.444
7.434
7.425
7.402
7.389
7.376
7.363
7.334
7.313
7.295
7.251
7.125
7.103
7.052
7.042
7.031
7.020
6.950
6.942
6.932
6.923
-0.000
6r
1.00
1.01
1.00
0.90
1.11
2.00
1.05
1.94
2.01
1.10
1.04
2.00
1.99
10.0 9.5 9.0 8.5 8.0 7.5 7.0 6.5 6.0 5.5 5.0 4.5 4.0 3.5 3.0 2.5 2.0 1.5 1.0 0.5 0.0
f1 (ppm)

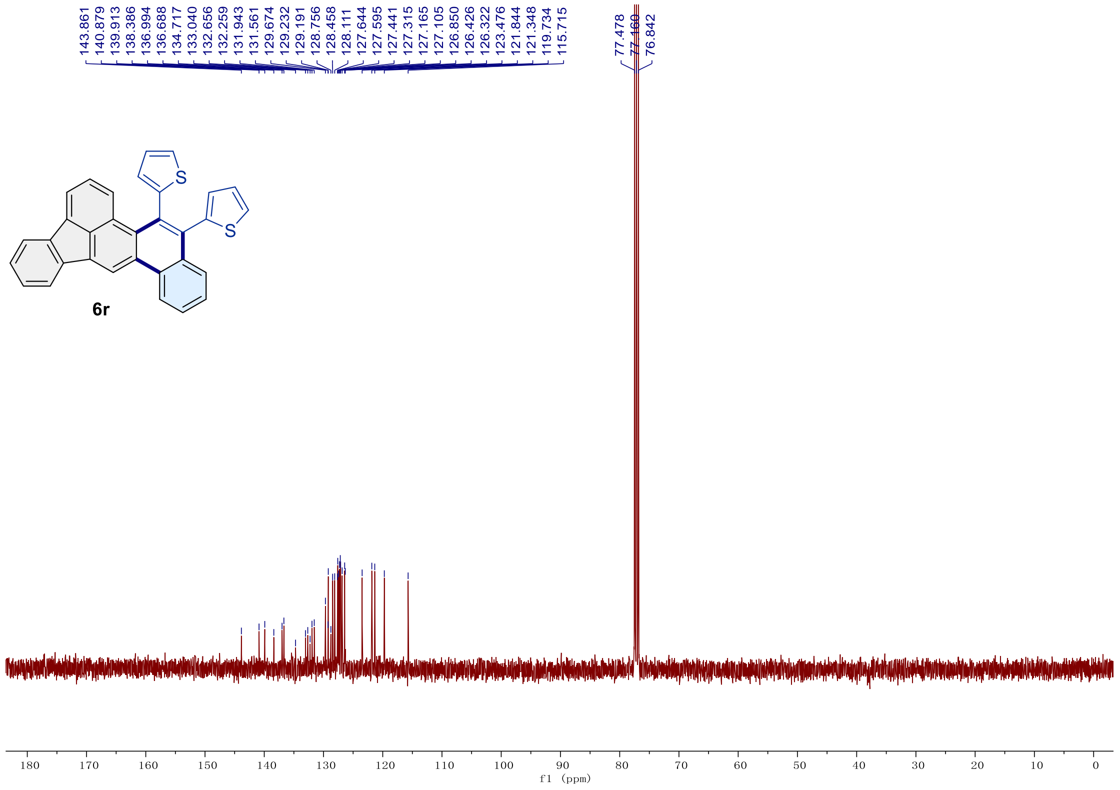
143.861
140.879
139.913
138.386
136.994
136.688
134.717
133.040
132.656
132.259
131.943
131.561
129.674
129.232
129.191
128.756
128.458
128.111
127.644
127.595
127.441
127.315
127.165
127.105
126.850
126.426
126.322
123.476
121.844
121.348
119.734
115.715
77.478
77.160
76.842
6r
180 170 160 150 140 130 120 110 100 90 80 70 60 50 40 30 20 10 0
f1 (ppm)

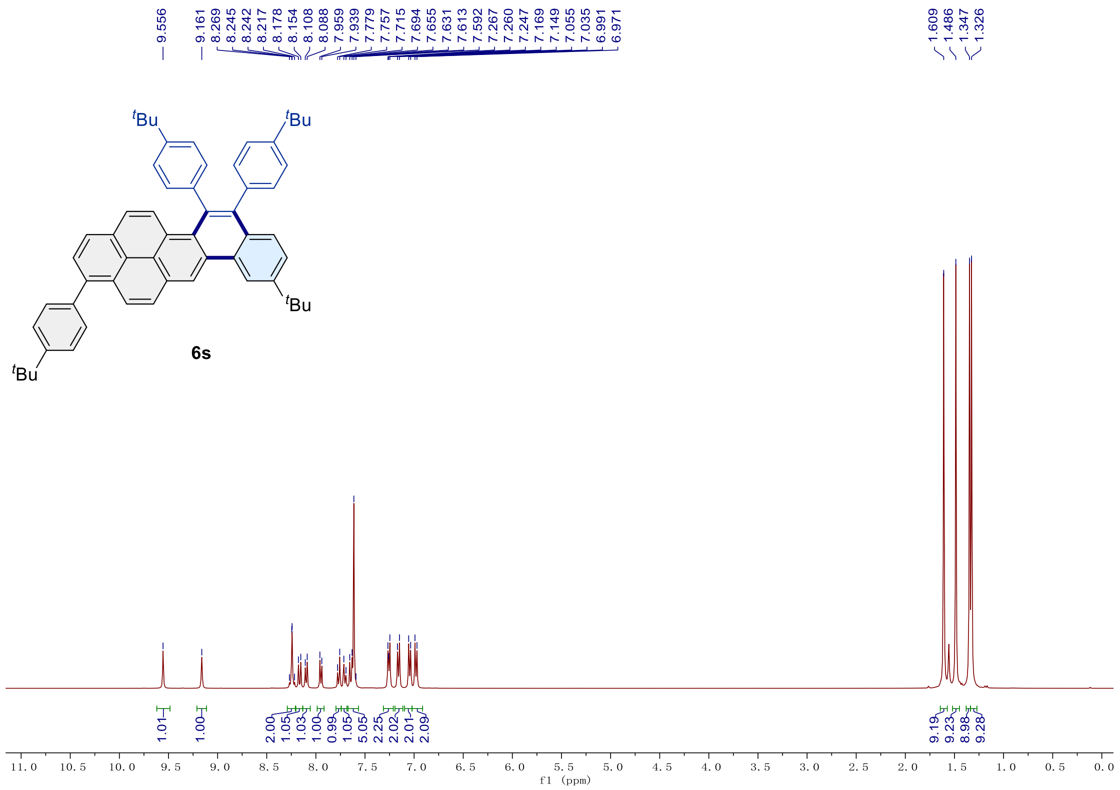
9.556
9.161
8.269
8.245
8.242
8.217
8.178
8.154
8.108
8.088
7.959
7.939
7.779
7.757
7.715
7.694
7.655
7.631
7.613
7.592
7.267
7.260
7.247
7.169
7.149
7.055
7.035
6.991
6.971
1.609
1.486
1.347
1.326
tBu
tBu
tBu
tBu
6s
1.01
1.00
2.00
1.05
1.03
1.00
0.99
1.05
5.05
2.25
2.02
2.01
2.09
9.19
9.23
8.98
9.28
f1 (ppm)

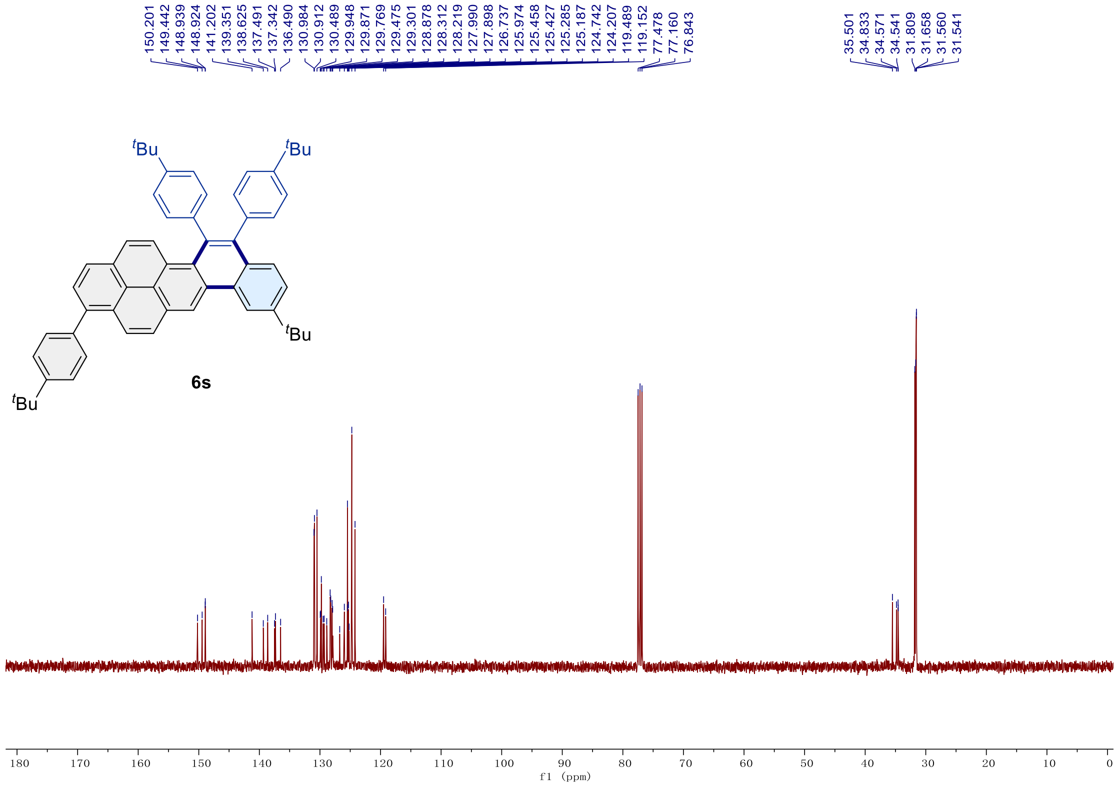
150.201
149.442
148.939
148.924
141.202
139.351
138.625
137.491
137.342
136.490
130.984
130.912
130.489
129.948
129.871
129.769
129.475
129.301
128.878
128.312
128.219
127.990
127.898
126.737
125.974
125.458
125.427
125.285
125.187
124.742
124.207
119.489
119.152
77.478
77.160
76.843
35.501
34.833
34.571
34.541
31.809
31.658
31.560
31.541
tBu
tBu
tBu
tBu
6s
f1 (ppm)

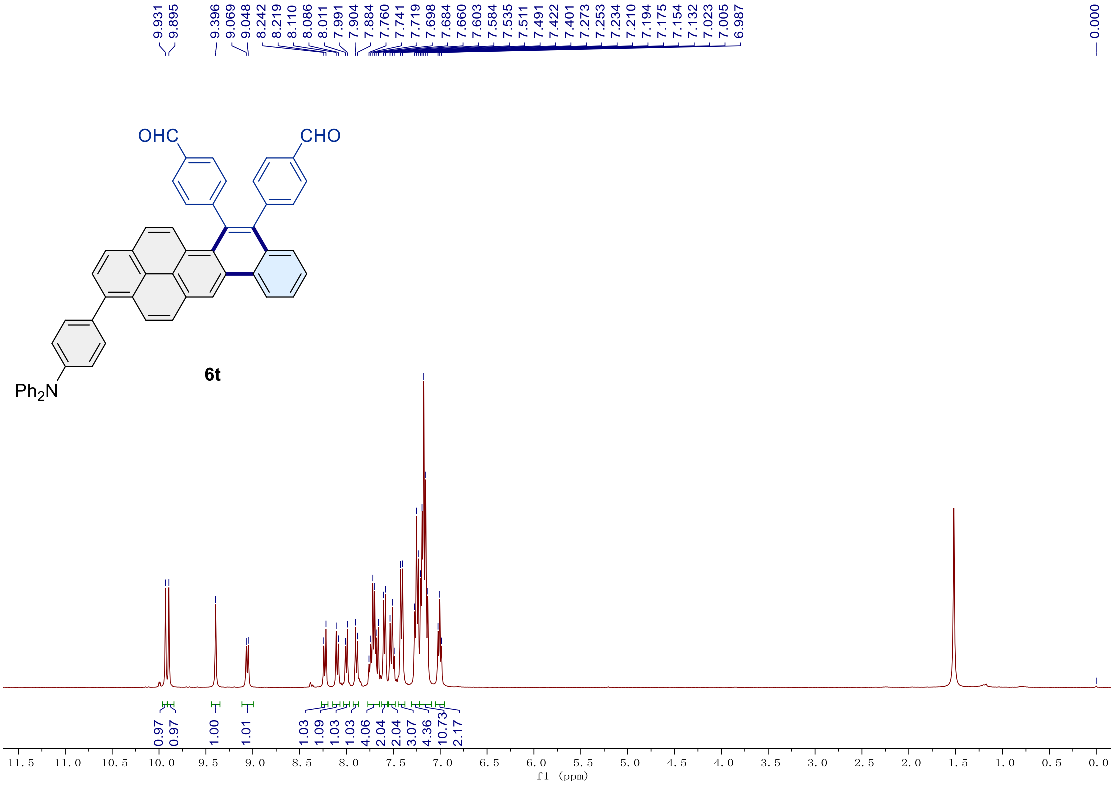
9.931
9.895
9.396
9.069
9.048
8.242
8.219
8.110
8.086
8.011
7.991
7.904
7.884
7.760
7.741
7.719
7.698
7.684
7.660
7.603
7.584
7.535
7.511
7.491
7.422
7.401
7.273
7.253
7.234
7.210
7.194
7.175
7.154
7.132
7.023
7.005
6.987
0.000
OHC
CHO
Ph2N
6t
0.97
0.97
1.00
1.01
1.03
1.09
1.03
1.03
4.06
2.04
2.04
3.07
4.36
10.73
2.17
f1 (ppm)

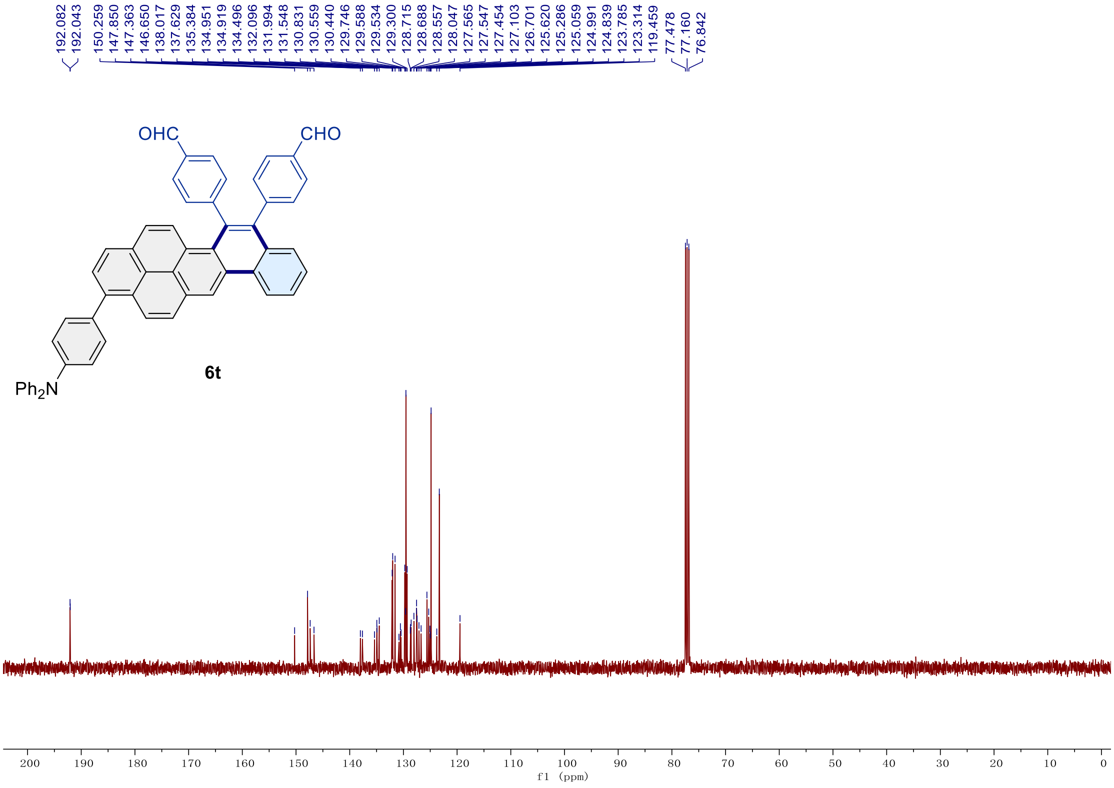
192.082
192.043
150.259
147.850
147.363
146.650
138.017
137.629
135.384
134.951
134.919
134.496
132.096
131.994
131.548
130.831
130.559
130.440
129.746
129.588
129.534
129.300
128.715
128.688
128.557
128.047
127.565
127.547
127.454
127.103
126.701
125.620
125.286
125.059
124.991
124.839
123.785
123.314
119.459
77.478
77.160
76.842
OHC
CHO
Ph2N
6t
f1 (ppm)

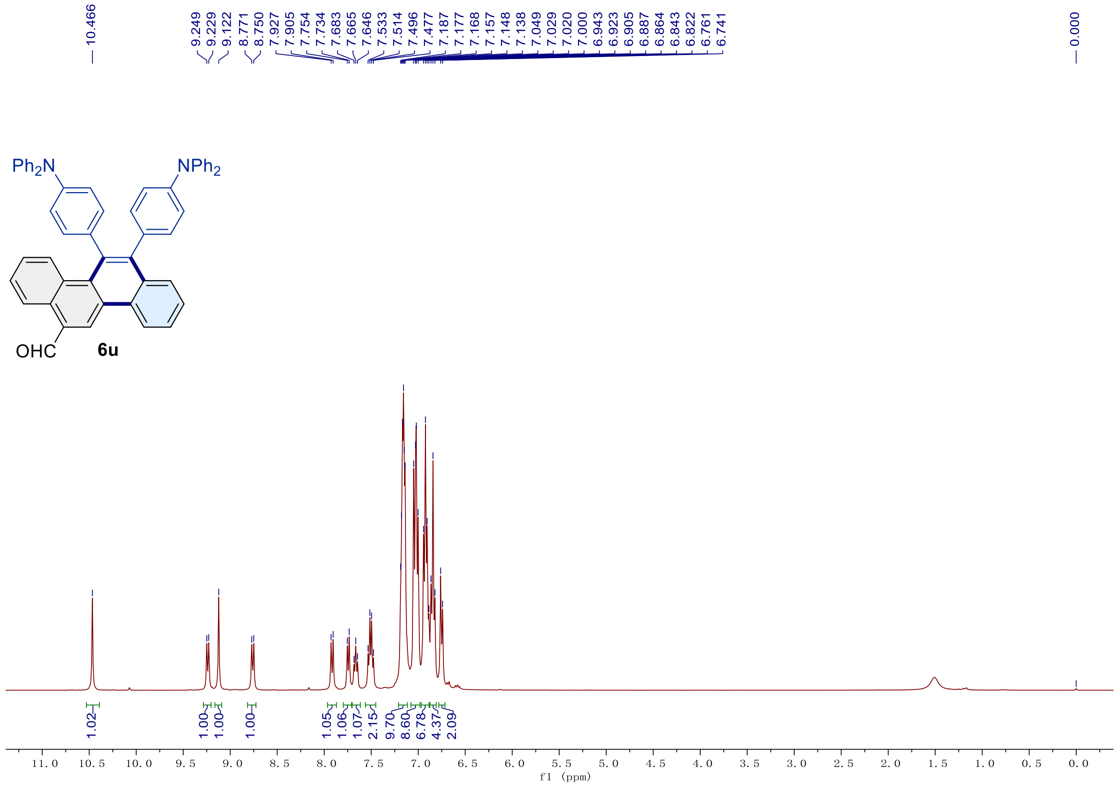

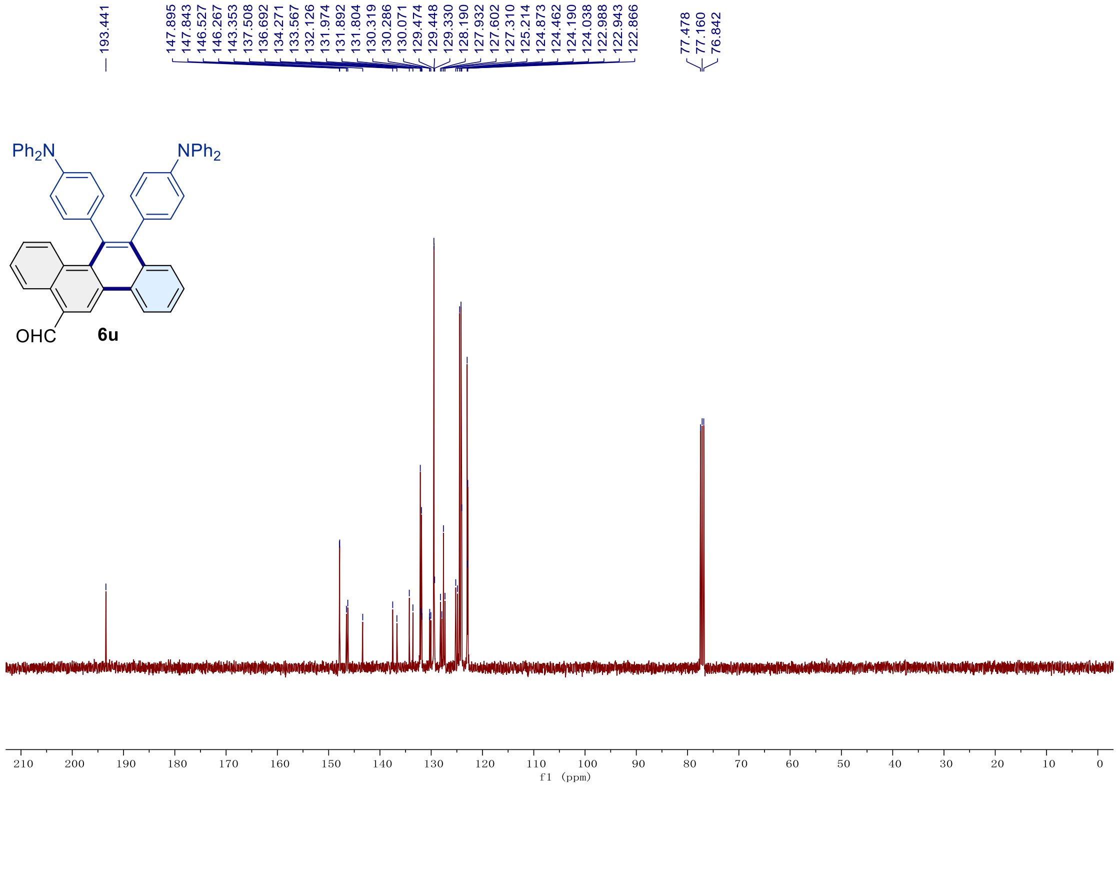

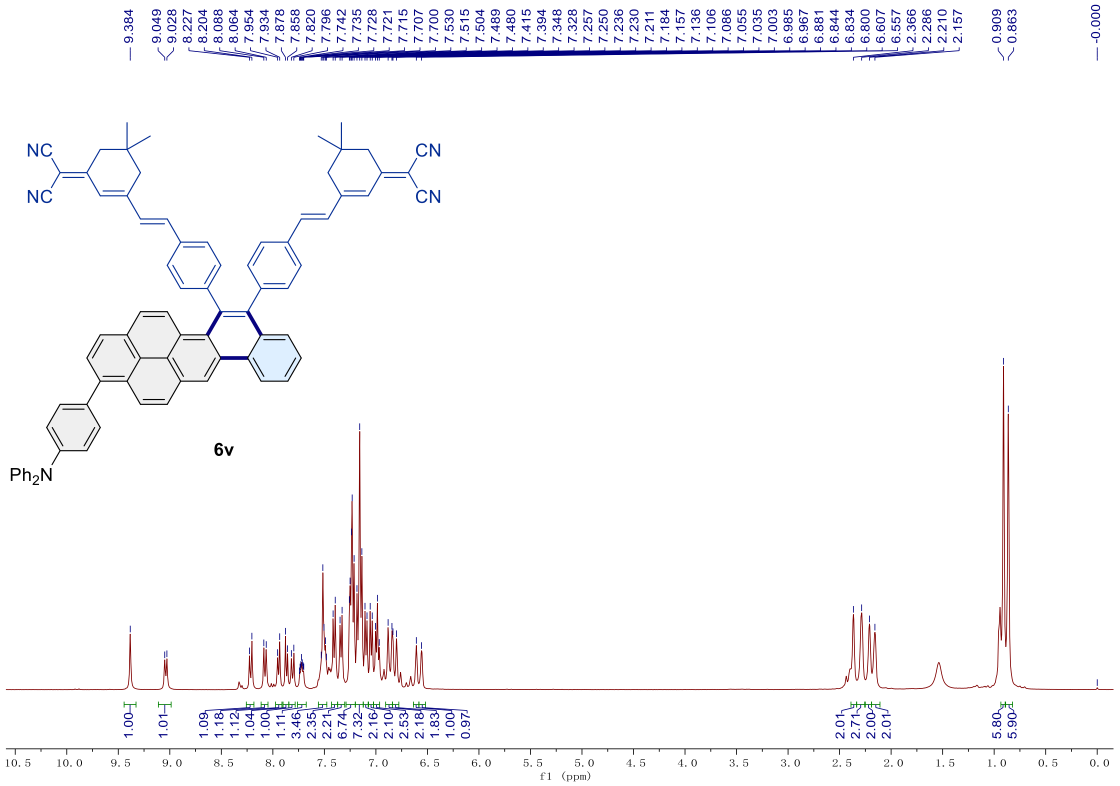
6v
Ph2N
NC
CN
f1 (ppm)

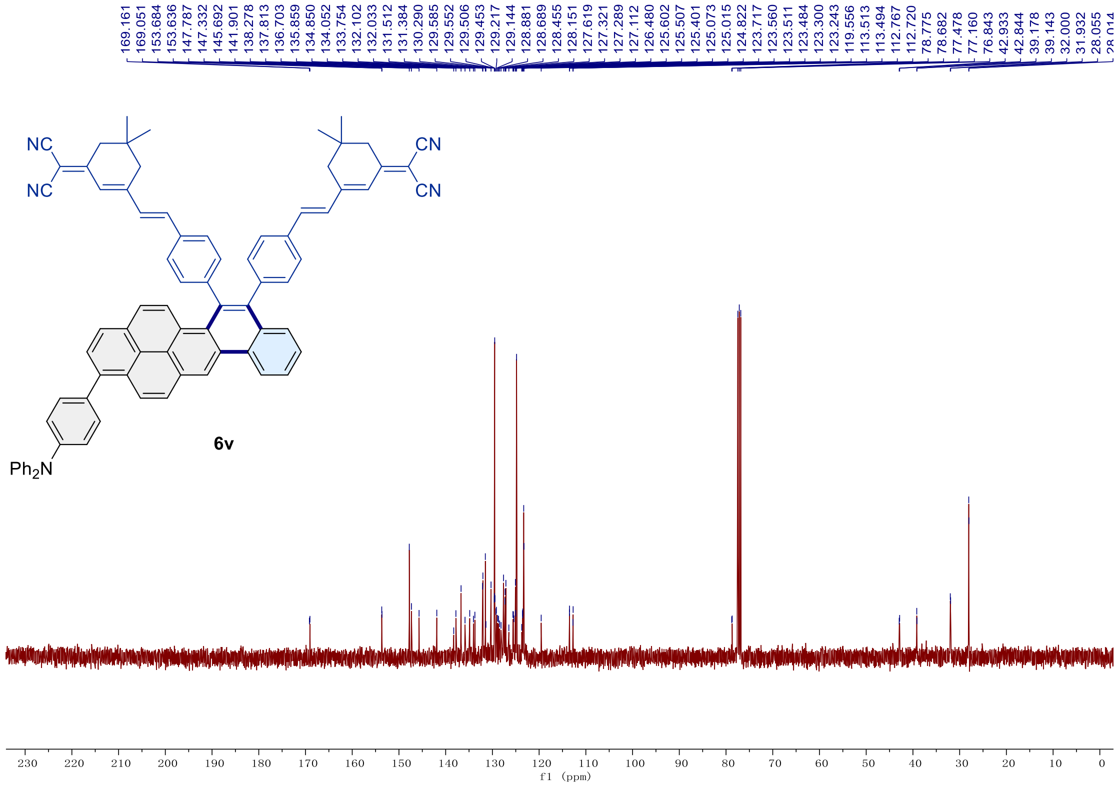
6v
Ph2N
NC
CN
f1 (ppm)

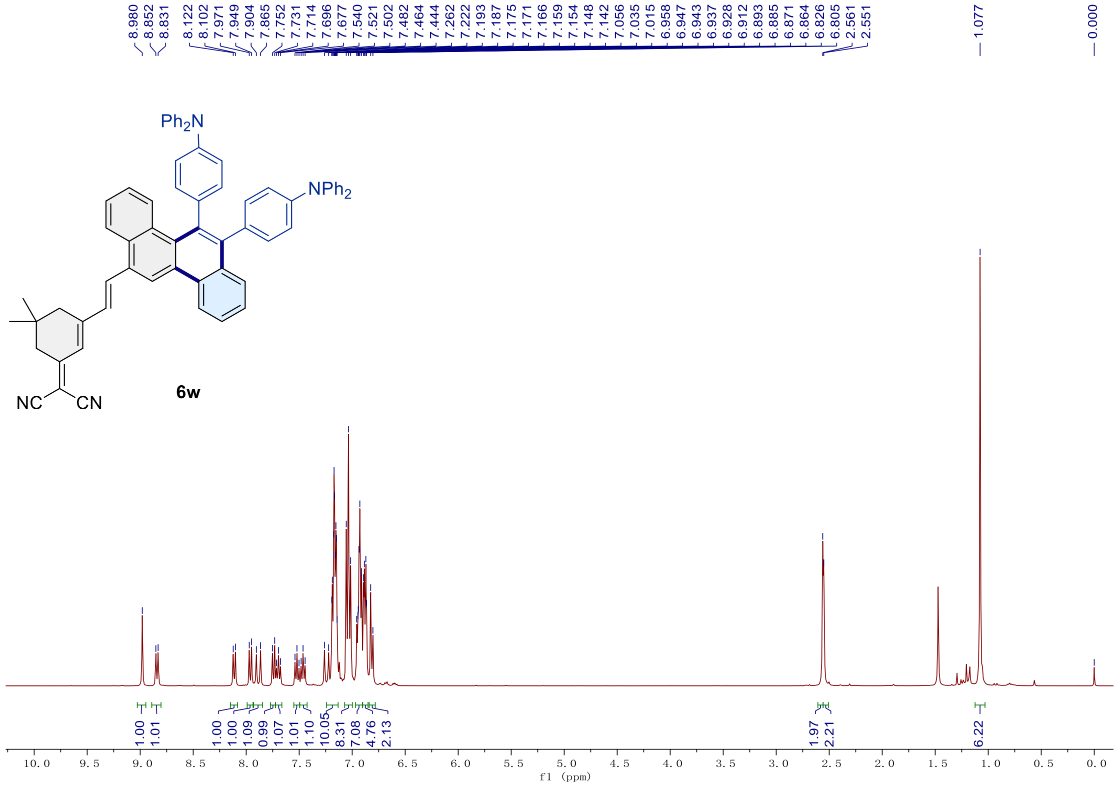
8.980
8.852
8.831
8.122
8.102
7.971
7.949
7.904
7.865
7.752
7.731
7.714
7.696
7.677
7.540
7.521
7.502
7.482
7.464
7.444
7.262
7.222
7.193
7.187
7.175
7.171
7.166
7.159
7.154
7.148
7.142
7.056
7.035
7.015
6.958
6.947
6.943
6.937
6.928
6.912
6.893
6.885
6.871
6.864
6.826
6.805
2.561
2.551
1.077
0.000
Ph2N
NPh2
NC
CN
6w
1.00
1.01
1.00
1.00
1.09
0.99
1.07
1.01
1.10
10.05
8.31
7.08
4.76
2.13
1.97
2.21
6.22
10.0 9.5 9.0 8.5 8.0 7.5 7.0 6.5 6.0 5.5 5.0 4.5 4.0 3.5 3.0 2.5 2.0 1.5 1.0 0.5 0.0
f1 (ppm)

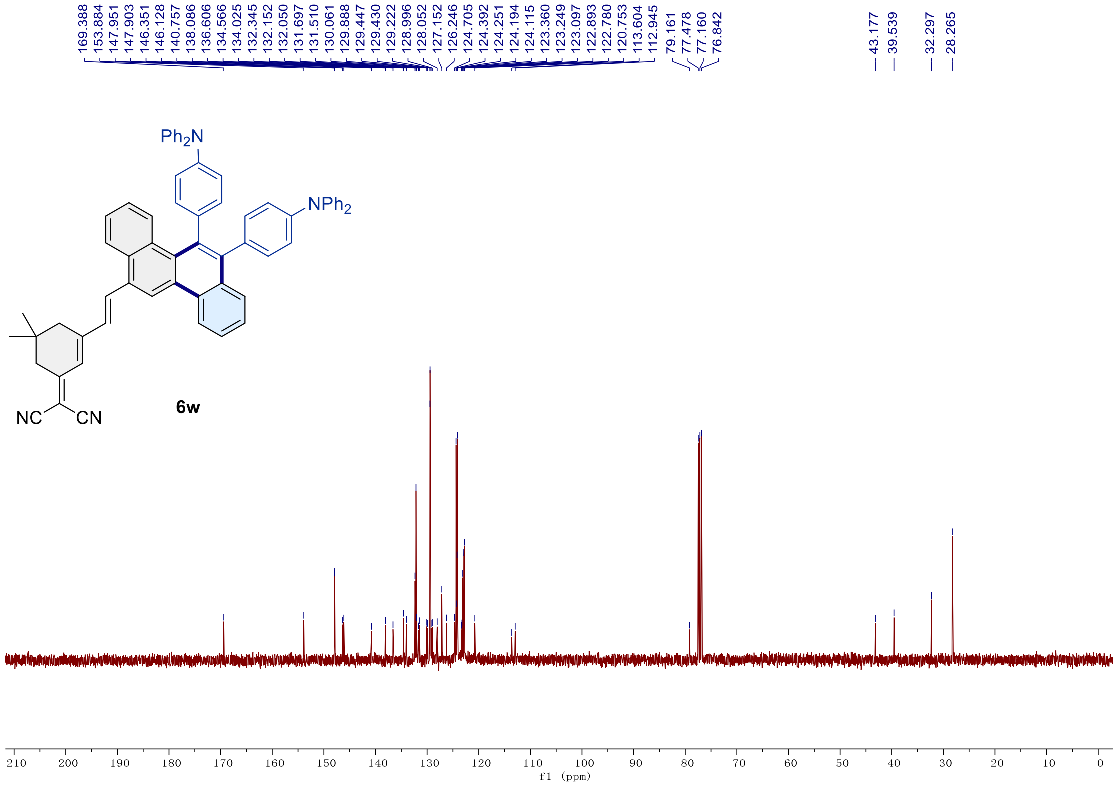
169.388
153.884
147.951
147.903
146.351
146.128
140.757
138.086
136.606
134.566
134.025
132.345
132.152
132.050
131.697
131.510
130.061
129.888
129.447
129.430
129.222
128.996
128.052
127.152
126.246
124.705
124.392
124.251
124.194
124.115
123.360
123.249
123.097
122.893
122.780
120.753
113.604
112.945
79.161
77.478
77.160
76.842
43.177
39.539
32.297
28.265
Ph2N
NPh2
NC
CN
6w
210 200 190 180 170 160 150 140 130 120 110 100 90 80 70 60 50 40 30 20 10 0
f1 (ppm)

NMR spectrum of substrates 1t – u:

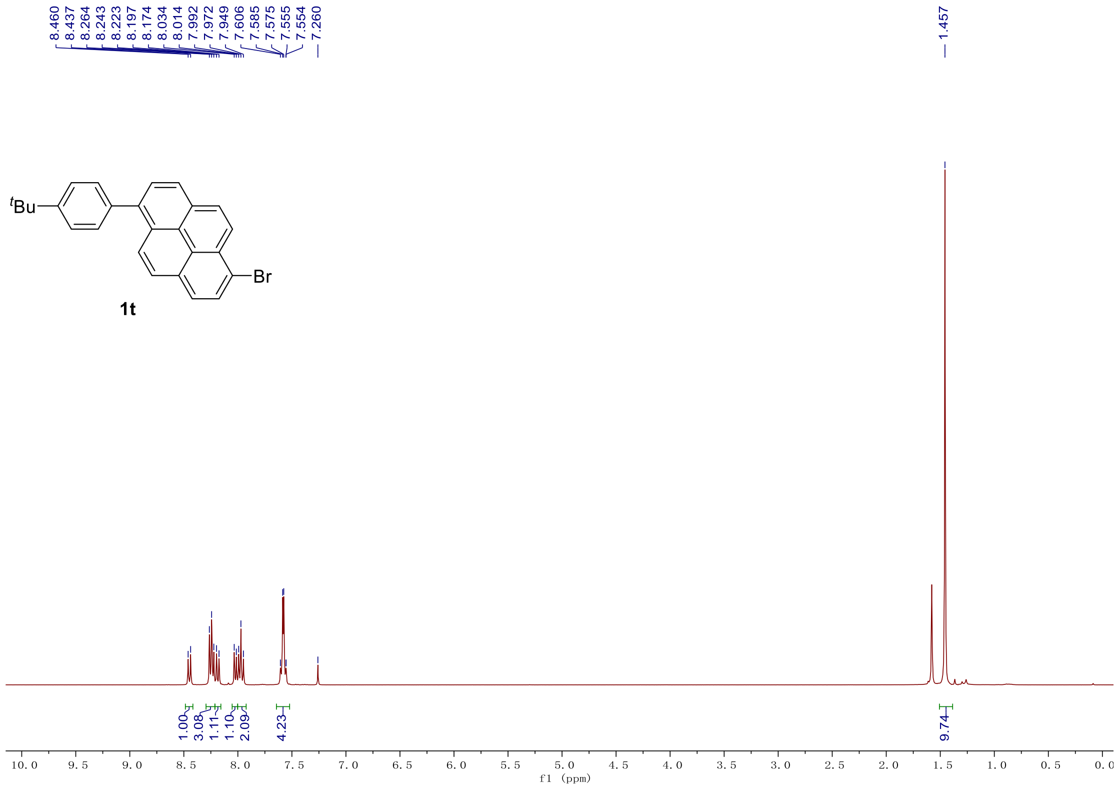

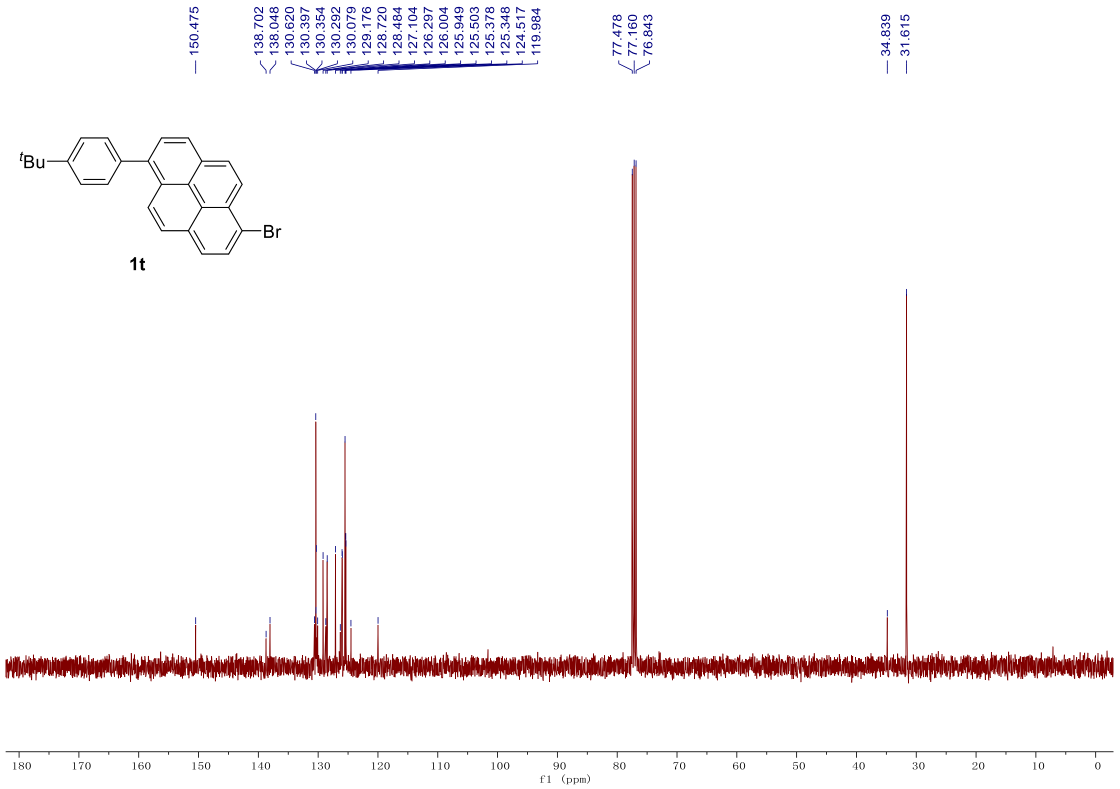

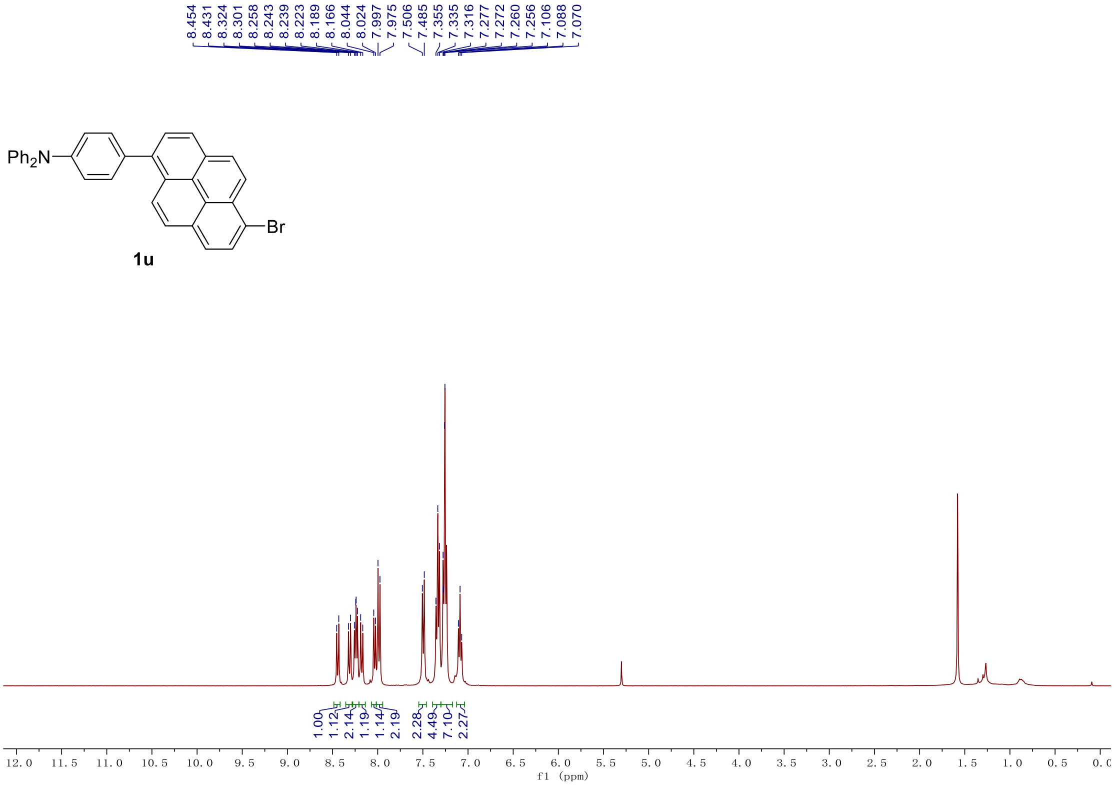
Ph2N
Br
1u
8.454
8.431
8.324
8.301
8.258
8.243
8.239
8.223
8.189
8.166
8.044
8.024
7.997
7.975
7.506
7.485
7.355
7.335
7.316
7.277
7.272
7.260
7.256
7.106
7.088
7.070
1.00
1.12
2.14
1.19
1.14
2.19
2.28
4.49
7.10
2.27
f1 (ppm)

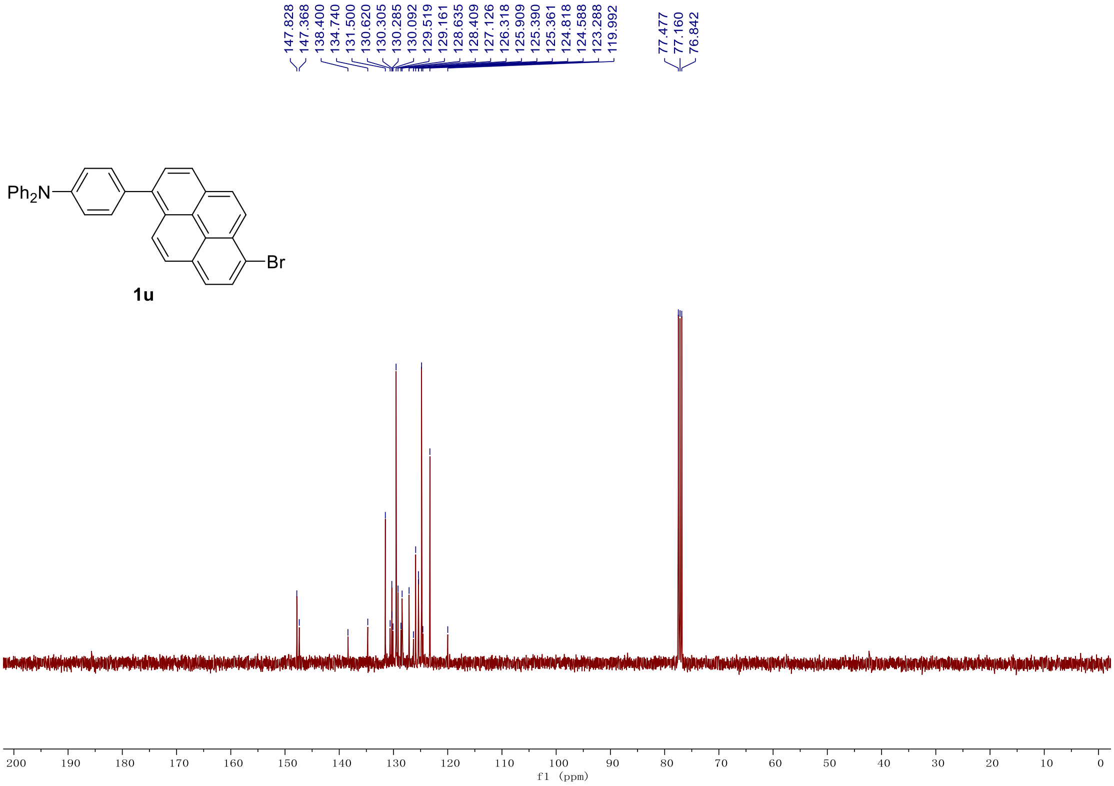
Ph2N
Br
1u
147.828
147.368
138.400
134.740
131.500
130.620
130.305
130.285
130.092
129.519
129.161
128.635
128.409
127.126
126.318
125.909
125.390
125.361
124.818
124.588
123.288
119.992
77.477
77.160
76.842
f1 (ppm)
